# Paeoniflorin ameliorates cognitive impairment in Parkinson’s disease via JNK/p53 signaling

**DOI:** 10.1007/s11011-022-00937-2

**Published:** 2022-03-01

**Authors:** Zhu-qing He, Peng-fei Huan, Li Wang, Jian-cheng He

**Affiliations:** 1 Department of Diagnostics of Traditional Chinese Medicine, Shanghai University of Traditional Chinese Medicine, Shanghai, 201203 China; 2 Shanghai Municipal Hospital of Traditional Chinese Medicine, Shanghai University of Traditional Chinese Medicine, Shanghai, 200071 China; 3 Shanghai Key Laboratory of Health Identification and Assessment, School of Basic Medicine, Shanghai University of Traditional Chinese Medicine, Shanghai, 201203 China

**Keywords:** Parkinson’s disease, Paeoniflorin, Cognitive impairment, JNK/p53 pathway, Network pharmacology

## Abstract

Paeoniflorin (PF) has numerous benefits, including anti-inflammatory and anti-apoptosis effects. However, it is not clear if it has neuroprotective effects against cognitive impairment (CI) in Parkinson’s disease (PD). Through network pharmacology, we identified probable targets as well as signal pathways through which PF might affect CI in PD. Then, we experimentally validated our findings. The core genes of the protein-protein interactions (PPI) network include MAPK8 (JNK), TP53, CASP3 (caspase-3), postsynaptic density protein-95 (PSD-95) and synaptophysin (SYN). Pathway enrichment analysis revealed that genes involved in apoptosis and mitogen-activated protein kinase (MAPK) signaling were significantly enriched. Because JNK is a key mediator of p53-induced apoptosis, we wondered if JNK/p53 pathway influences the effects of PF against apoptosis in mouse model of PD. Molecular docking analysis showed that PF had good affinity for JNK/p53. The results of the experiments indicated that PF ameliorated behavioral impairments and upregulated the expression of the dopamine (DA) neurons, suppressed cell apoptosis in substantia nigra pars compacta (SNpc) of PD. Additionally, PF improved 1-methyl-4-phenyl-1,2,3,6-tetrahydropyridine (MPTP)-induced neuronal injury by inhibiting apoptosis in hippocampal neurons of the CA1 and CA3, and upregulating PSD-95 as well as SYN protein levels. Similar protective effects were observed upon JNK/p53 pathway inhibition using SP600125. Overall, PF improved CI in PD by inhibiting JNK/p53 pathway.

**Supplementary Information:**

The online version contains supplementary material available at 10.1007/s11011-022-00937-2.

## Introduction

Parkinson’s disease (PD) is the 2nd most prevalent neurodegenerative disorder after Alzheimer's disease (AD) (Xicoy et al. 2020). cognitive impairment (CI) is a prevalent non-motor symptom of PD (Santangelo et al. 2015). CI affects >80% of PD patients and can cause dementia (Macchi et al. 2021), thereby affecting many aspects of life and increasing the risk of premature death (Baiano et al. 2020). Thus, elucidation of the mechanisms involved in CI among PD patients is urgently needed for better outcomes. Dopaminergic neurons in substantia nigra and ventral tegmental area project to the hippocampal region through midbrain and cortex dopamine system pathway (Espadas et al. 2021). Dopaminergic projection to hippocampus participates in cognitive function in PD (Jokinen et al. 2009). The CA1 region of the hippocampus is involved in cognitive processes, particularly learning, and memory (Li et al. 2020), and which along with CA3, forms the hippocampal Schaffer collateral pathway, is one of the most studied hippocampal synaptic pathway (Zamora-Moratalla and Martín 2021). Dopamine (DA) released from dopaminergic neurons plays important roles in synaptic plasticity processes. When dopaminergic neurons in the ventral tegmental areas and substantia nigra are damaged, causes a reduction of the direct dopaminergic projections. This could eventually result in reduced levels of neurogenesis and synaptic plasticity in hippocampus.

The JNK pathway plays a key role in cell differentiation, apoptosis and in synaptic plasticity processes (Ji et al. 2020). Studies of JNK knockout mice or mice treated with the JNK inhibitor SP600125 have shown that JNK is involved in various aspects of neuronal excitation, learning, and memory formation (Gong et al. 2020). It has been shown that the expression of phosphorylated JNK (p-JNK) is increased in post-mortem brain samples from AD patients, and it is positive for colocalization with Aβ (Killick et al. 2014). And a number of studies have shown that the JNK signaling pathway is involved in Aβ-induced neuronal apoptosis (Chen et al. 2018; Olivera Santa-Catalina et al. 2017). And in our previous study, the apoptosis of the nigrostriatal pathway of PD is mediated by JNK signaling (Wang et al. 2021). Therefore, JNK pathway may be a potential target for the prevention or treatment in CI of PD.

Paeoniflorin (PF), a monoterpene glycoside, is the main active ingredient of herbaceous peony, which is used in traditional Chinese medicine (TCM). PF has anti-oxidative (Li et al. 2017), anti-inflammatory (Wen et al. 2019) and anti-apoptosis (Wei et al. 2021) effects, and has attracted increasing attention due to its neuroprotective properties. Treatment with PF is reported to attenuate amyloid-beta deposition in hippocampus and restore amyloid-beta induced memory dysfunction (Kong et al. 2020). However, the role and mechanism by which PF improves PD-associated CI is unclear.

Network pharmacology, an emerging discipline, is based on systems biology (Yang et al. 2021) and is often used to uncover molecular mechanisms underlying chronic and complex diseases like PD (Wang et al. 2021). Here, we sought to elucidate the mechanisms underlying the effects of PF in PD-related CI using network pharmacology and experimental validation. Integrated GO (Gene Ontology), KEGG (Kyoto Encyclopedia of Genes and Genomes) and Molecular docking analyses indicated that PF may exert its anti-PD-related cognitive effects via JNK/p53 signaling. Additionally, experimental results showed that PF improved MPTP-induced neuronal injury by inhibiting apoptosis in hippocampal neurons of the CA1 and CA3, upregulated postsynaptic density PSD95 as well as SYN protein levels, and decreased the hippocampal levels of p-JNK/JNK, p-c-Jun/c-Jun, and p-p53/p53. Overall, PF improved cognitive impairment in PD by inhibiting JNK/p53 signaling pathway.

## Materials and methods

Sixty male C57BL/6J mice weighing 16-18 g and aged 5-6 weeks were obtained from the Animal Experimental Animal Center of Shanghai University of TCM, China (No. SYXK (Hu) 2020-0009). Paeoniflorin (PF) and 1-Methyl-4-phenyl-1,2,3,6-tetrahydropyridine (MPTP) were procured from Sigma Chemicals. The antibodies against p-JNK (Thr183/Tyr185), JNK, p-p53, p53, SYN, Bcl-2, PSD95, Bax and cleaved Caspase3 were purchased from Cell Signaling Technology. The antibodies against p-c-Jun, c-Jun (Ser73) and tyrosine hydroxylase (TH) were purchased from Abcam. Antibody against beta amyloid antibody (MOAB-2) was purchased from Novus Biologicals. TUNEL assay kit was obtained from Roche. SP600125 (S1066) was procured from Selleck.

### Network pharmacology analysis

#### Prediction of potential PF targets

Information on PF was obtained from https://old.tcmsp-e.com/tcmsp.php, http://www.lilab-ecust.cn/pharmmapper/ and http://www.swisstargetprediction.ch/databases. Disease-related genes were obtained from Therapeutic Target Database (TTD, http://db.idrblab.net/ttd/), Online Mendelian Inheritance in Man (OMIM, https://omim.org/), Genecards (https://www.genecards.org/), PharmGBK (https://www.pharmgkb.org/), and DrugBank (https://go.drugbank.com/).

#### Construction of the drug-target-disease network

Functional interactions of PF were elucidated using STRING (https://string-db.org/) and network visualization done using Cytoscape.

#### Establishment of the PPI network and core PPI network extraction

The core PPI network was build using STRING and Cytoscape.

#### Pathway and functional enrichment analysis

KEGG and GO analyses were used to predict associated targets and signaling pathways.

#### Molecular docking

Accuracy and stability were verified by molecular docking. Autodock Vina and optimal models were used and visualized using PyMOL (2.0).

### Experimental validation

#### Ethical statement

All animal experiment protocols received ethical approval from the Animal Experimental Animal Center of Shanghai University of TCM, China (license No. SYXK (Hu) 2020-0009). Animals were maintained at a controlled temperature and humidity (23±2°C and 60±65%, respectively) with free access to standard laboratory diet.

#### MPTP-induced mouse model of PD

To induce subacute PD, mice were intraperitoneally (i.p.) injected with MPTP (30mg/kg) for 5 days (Liu et al. 2021).

#### Group and drug treatments

Mice were randomly split into 5 groups, as follows, with 10 mice in each group: Control (Con) group mice were administered with saline (1mL/100g daily, i.p.). Mice in the model group received MPTP (30mg/kg, i.p.) from day 1-5. Mice in the PF group received MPTP (30mg/kg for 5 days) and then PF (30mg/kg/d, i.p.) for 7 days. Mice in the SP600125 group received MPTP (30mg/kg for 5 days) and then SP600125 (30mg/kg, i.p.) for 7 days. After 5 days of MPTP injections, Mice in the PF+SP600125 group received 30mg/kg SP600125 (i.p.) combined PF (30mg/kg/d, i.p.). After administration, all mice were trained for 3 days prior to the behavioral tests. And each behavioral test was tested for three times.

#### Neurobehavioral observations

##### Pole test

The pole test is commonly used to provide motor-symptomatic relief in PD patients (Matsuura et al. 1997). The test was done by wrapping a 50cm long and 1cm wide pipe with gauze and fixing a wooden ball to the top. Subsequently, mice were placed on the wooden ball after which the time taken by the mouse to move from the top to bottom of the tube documented.

##### Open field test

The open field test is frequently used to assess activity after MPTP injury (Sedelis et al. 2001). Before the experiment, four mice from each group were placed in the observation box (25×25×25 cm) and allowed to adapt for 30 minutes. They were then videotaped for 30 min and automated video analysis used to assess the movement trajectory of the mice and the total distance covered in 30 min noted as an indicator of horizontal movement.

#### *Morris water maze test (*MWMT*)*

The standard 6-day MWMT was used to assess cognitive function (Si et al. 2016) and data recorded and analyzed using tracking software.

#### Western blot analysis

Protein extraction was done using a T-PERTM tissue protein extraction reagent (Thermo Scientific, USA) and protein levels determined using a BCA kit (Beyotime, Shanghai, China). Overnight incubation of the membranes at 4°C was done with primary antibodies against TH (1:2000), Bax (1:1000), Bcl-2(1:1000), cleaved Caspase 3 (1:1000), PSD95 (1:1000), p53 (1:1000), SYN (1:1000), JNK (1:500), c-Jun (1:1000), p-JNK (1:500), p-c-Jun (1:1000), as well as p-p53(1:1000) after which they were incubated with secondary antibodies. A LI-COR Odyssey scanner was used to detect fluorescence signal (Biosciences, USA) and image J used to measure the strip optical density.

#### Immunohistochemistry (IHC)

Brain samples were sectioned at 30μm and blocked for 1 h in 5% BSA at room temperature (RT). They were then incubated with mouse anti-TH antibody (1:1000). Next, incubation of the sections with HRP-conjugated secondary antibodies was done for 1 h after which they were incubated for 3 min with 3,3′-diaminobenzidine (DAB). Positive cells were counted on Image J Pro Plus.

#### Immunofluorescence (IF) staining

Brain tissue were cryosectioned at 30μm. The sections were then washed three times using PBS, 5 minutes/wash, and blocked for 30 min using 0.3% BSA in 5% Triton X-100. Then, they were incubated in the presence of antibodies against Aβ (1:1000; Novus Biologicals, USA), followed by incubation with secondary antibodies for 1h and imaged on a confocal microscope and positive cells counted using Image J.

#### TUNEL assay

TUNEL staining was performed on 30μm cryosections using an *in situ* cell death detection kit (Roche, Switzerland Basel, Germany) and imaged by confocal fluorescence microscopy (Solms, Germany). Apoptotic cells stained green, while nuclei were stained blue. Image-Pro Plus 6.0 was used to count positive cells.

#### Nissl’s staining

Brain sections were subjected to Nissl staining using the conventional method. Paraffin embedded tissues were sectioned at 5μm, dewaxed in xylene and rehydrated in graded ethanol. Sections were then stained with 1% toluidine blue for 5 min and then soaked in 70 and 95% ethanol for 5 minutes each, respectively. After that, the nuclei turned blue and the background were colorless, followed by 100%, 90%, 80%, and 70% ethanol at room temperature. Surviving neurons in hippocampal CA1 and CA3 were observed under a microscope (Nikon, Tokyo, Japan).

#### Statistical analysis

Data were presented as Mean ± SEM. Differences between two groups were assessed paired/unpaired t-test. Differences among groups were evaluated by one- or two-way ANOVA, followed by Tukey’s post hoc test. *P=*<0.05 was the cut-off for significance.

## Results

### Network pharmacology screening

#### Construction of a “PF-Target-PD” Network

**Fig. 1:**
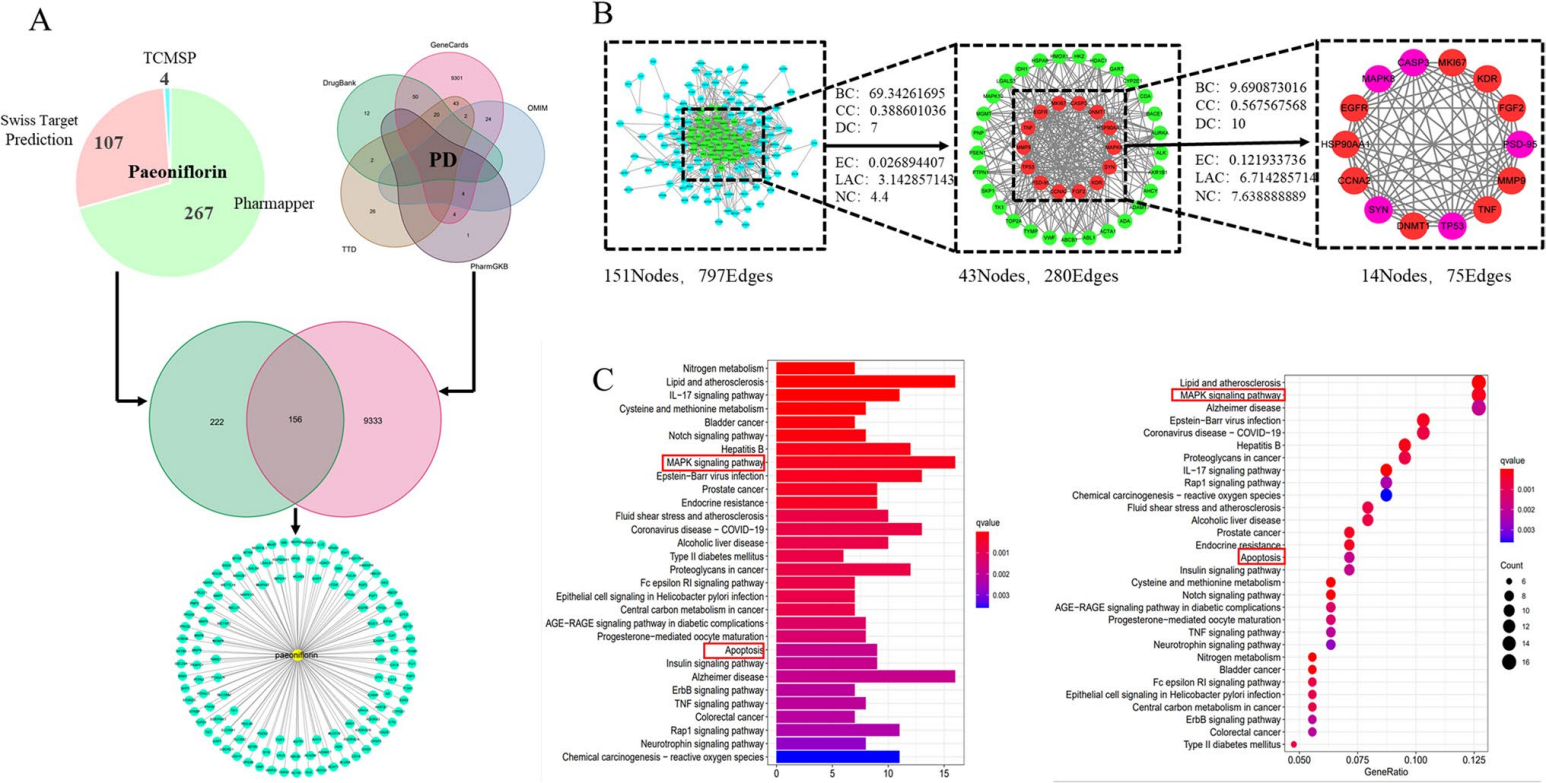
**Network pharmacology screening.** (**A**) The construction of the ingredient-target network of PF. (**B**) Identification of core candidate targets of PF against PD, BC, betweenness centrality; CC, closeness centrality; DC, degree sentrality; EC, eigenventor centrality; LAC, local average connectivity-based method; NC, network centrality. (**C**) Gene ontology terms and KEGG pathway enrichment of core candidate targets of PF against PD. The top-10 terms in each GO category with p Adjust Value < 0.05 were selected. BP, biological process; CC, cellular components; MF, molecular function. KEGG pathway enrichment of core candidate targets of PF against PD. The top-30 pathways that had significant changes of P Adjust Value < 0.05 were identified.

Retrieved from TCMSP, Pharmapper and Sisstarget data repositories, there were 378 related PF targets (Figure 1A) (Supplementary File S1). A total of 9489 PD-associated genes were identified from the online disease database (Figure 1A, Supplementary File S2). A total of 156 genes overlapped between the two datasets (Figure 1A). The “PF-Targets-PD” network was build using STRING and visualized on Cytoscape (Figure 1A and Supplementary File S3).

#### PPI network construction

The largest component of the network had 797 edges and 151 nodes (Figure 1B). Cytoscape was used to compute topological parameters and to obtain the core PPI network from the PPI network. The core PPI network had 14 nodes and 75 edges, including CASP3 (caspase-3), TP53, PSD95, MAPK8 (JNK) and SYN (Figure 1B, Supplementary File S4).

#### KEGG and GO enrichment analyses

Figure 1C shows the top-10 enriched GO terms. Regulation and execution of apoptosis were found to be enriched (Supplementary File S5). KEGG pathway analysis of the 156 genes identified 93 abundant signaling pathways, including MAPK and apoptosis signaling pathways (Supplementary File S6). Figure1C shows the most enriched of the top-30 functional terms.

#### Molecular docking

**Fig. 2:**
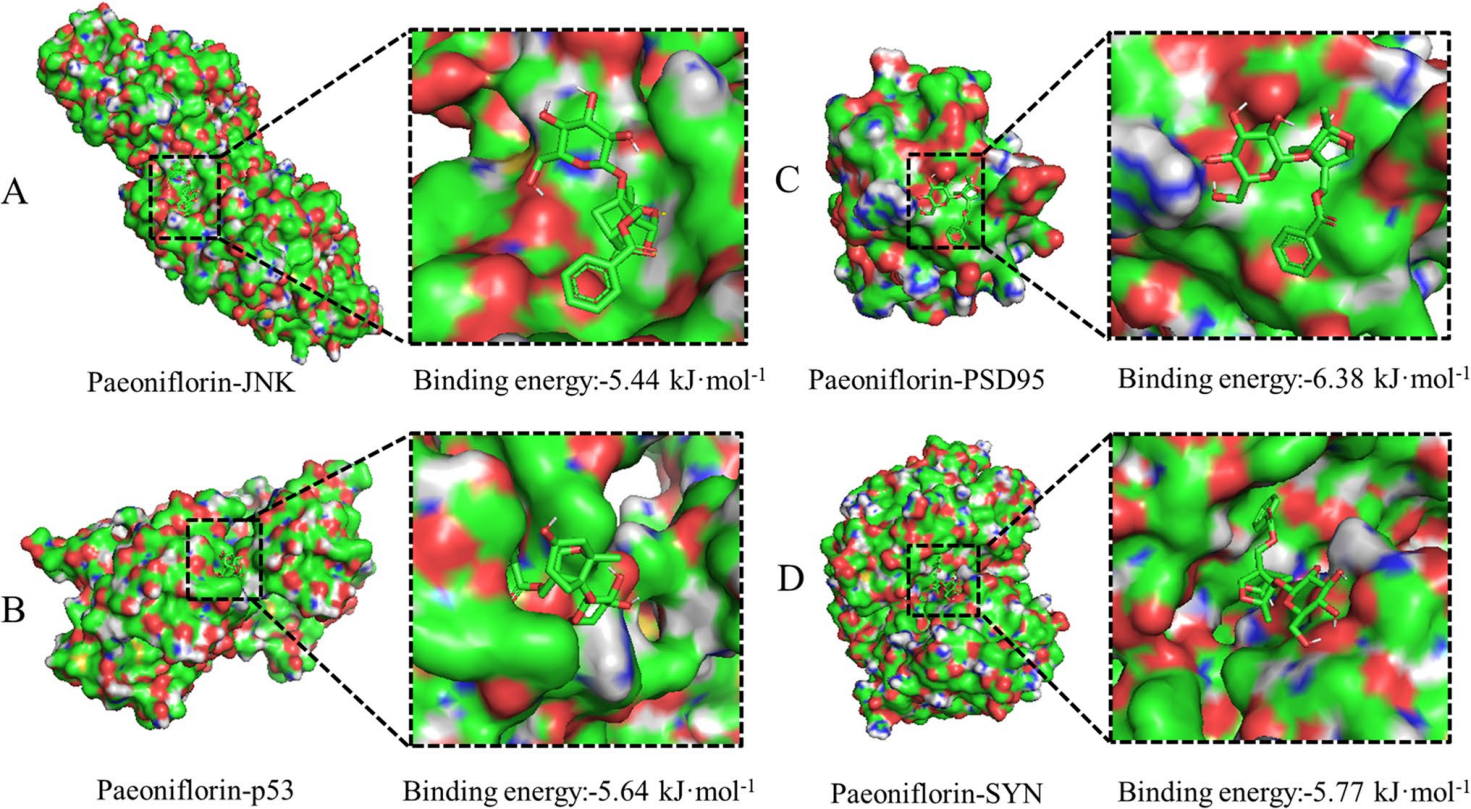
**Molecular docking.** To investigate the binding affinity of PF with JNK/p53, and the synapse associated proteins. The ligands (ingredient molecules) are shown in stick mode. The receptor is shown in surface model.

Molecular docking analysis was used to evaluate the binding affinity of PF to JNK/p53 and synapse-associated proteins (Figure 2). Respectively, the affinities of JNK, p53, PSD95, and SYN for PF were -5.44, -5.64, -6.38, and - 5.77 kcal/mol, suggesting good binding affinities. These findings show that PF exerts neuroprotective effects by inhibiting apoptosis and highlight MAPK signaling as a potential therapeutic target. p53, a target of MAPK signaling, is a crucial mediator of normal cell differentiation and survival. Thus, we assessed if the JNK/p53 pathway contributes to PF’s effects on PD-related cognitive decline.

### Experimental validation

#### PF ameliorated behavioral symptoms, learning, and memory in MPTP-induced PD Mice

**Fig. 3:**
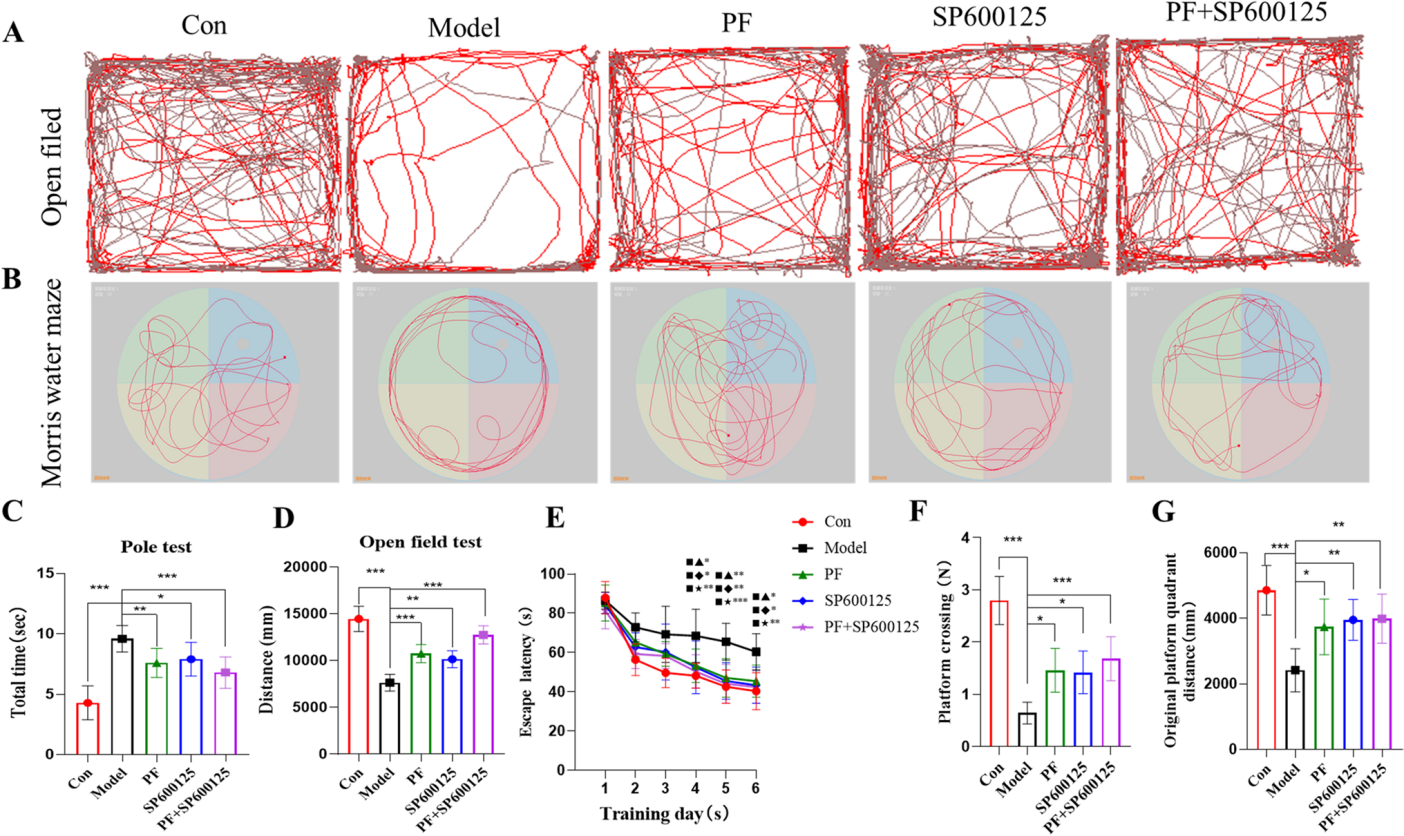
**Evaluation of behavioral symptoms and learning and memory in PD mice.** (**A**) The autonomous trajectory map of mice in the Open field test was recorded by the device. (**B**) Behavioral assays of climbing time in 13. (**C**) The total distance travelled in 5 min is shown. (**D**) Swim paths obtained during the track maps of the MWMT probe. (**E**) Escape latency in the MWMT plotted against the training days. (**F**) The platform crossing times during a 120 s probe trial of the MWMT. (**G**) The swimming distance in the original platform quadrant. Statistical analysis was performed with One-Way ANOVA or Two-Way ANOVA, n=6, Significant differences were indicated by * *P* < 0.05; ** *P* < 0.01; *** *P* < 0.001.

Behavior was assessed 5 days after the final MPTP injection (Figure 3). Analysis of the distance traveled in 14 as gauge of behavioral ability showed that the control group exhibited high values than the model group (*p=*<0.001). Total distances covered by PD mice treated with PF, SP600125 (a specific JNK inhibitor), and PF+SP600125 was significantly higher relative to untreated PD mice (*p*=<0.001, *p*=<0.01, *p=*<0.001, Figure 3A, D). The pole climbing time of PD mice was significantly lower, compared to non-PD control mice (*p=*<0.001). Treatment of PD-mice with PF, SP600125, and PF+SP600125 for 7 days, significantly enhanced their climbing time compared with untreated PD mice (*p=*<0.001, Figure 3C). Implying that PF, SP600125, and PF+SP600125 improve the behavioral ability of PD mice.

Spatial learning and memory function were assessed using MWMT in mice from day 13 (Figure 3B). The acquisition trials of MWMT on day 4-6 of the model group revealed an escape latency that was lengthier compared to that of the control group (*p=*<0.001). Moreover, PD mice treatment with PF, SP600125, and PF+SP600125 had markedly shorter latency on days 4-6, when compared to untreated PD mice (day 4: *p =* <0.05, <0.05, <0.01; day 5: *p =* <0.01, <0.01, and <0.001; day 6: *p =* <0.05, <0.05, and <0.01, respectively; Figure 3E). There were no marked differences between intervention groups (*p*=>0.05). Similar observations were made upon analysis of platform crossing time and original platform quadrant distance (Figure 3F-G). MWMT data indicated that PF could maintain learning and memory abilities that weakened upon MPTP exposure.

#### PF attenuated MPTP induced loss of DA neurons in PD mice

**Fig. 4:**
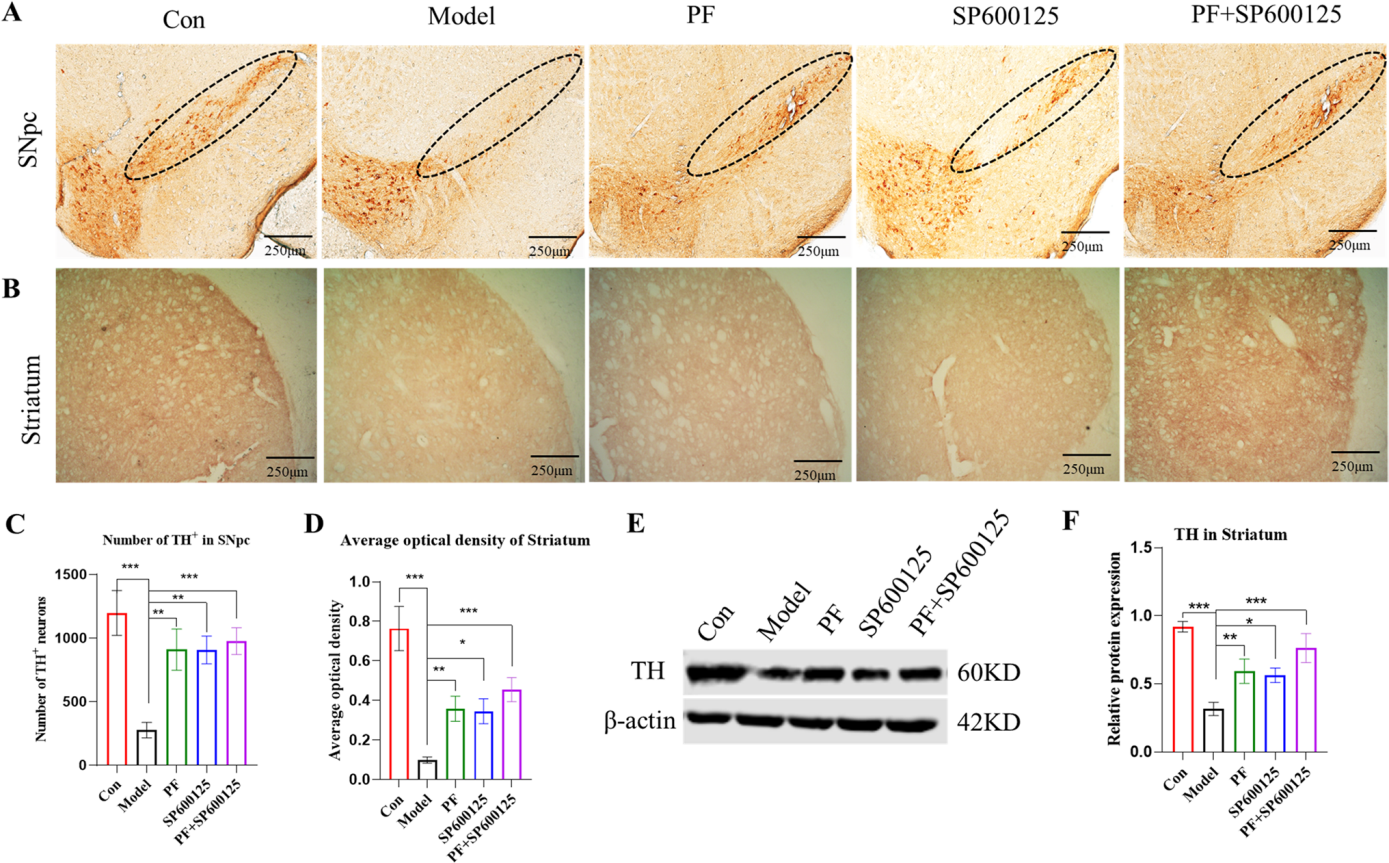
**PF attenuated MPTP induced loss of nigrostriatal DA neurons in the PD mice. (A-B)** DAB staining of TH onnigrostriatal of each group (Scale bar: 250 μm). (**C**)The counts of TH-positive cells of the SNpc. (**D**) Average optical density of the striatum of each group. (**E-F**) The expression level of TH proteins was detected with Western Blot in the Striatum, β-actin served as control. Statistical analysis was performed with One-Way ANOVA or Two-Way ANOVA, n = 3. Significant differences were indicated by * *P* < 0.05; ** *P* < 0.01; *** *P* < 0.001.

IHC examination of DA neuronal damage in substantia nigra (Figure 4A-B) revealed significantly fewer DA neurons in the PD model relative to non-PD controls (*p=*<0.001). Relative to untreated PD mice, PD mice treated with PF, SP600125, and PF+SP600125 had significantly higher number of DA neurons (*p =* <0.01*,* <0.01, and <0.001 respectively). Additionally, PD mice treated with PF, SP600125, and PF+SP600125 significantly improved the optical density of TH-positive fibers when compared to untreated controls (*p =* <0.01, <0.05, and <0.001, respectively, Figure 4C-D).

Western blot showed markedly suppressed levels of TH in the striata of PD mice, compared to control group (*p=*<0.001), which were significantly promoted upon treatment with PF, SP600125, and PF+SP600125, for 7 days (*p =* <0.01, <0.05, and <0.001, respectively (Figure 4E-F). These results indicate that PF protects from MPTP-induced neuronal damage and loss of dopaminergic neurons.

#### PF reduced MPTP-mediated apoptosis in nigrostriatal of PD mice

**Fig. 5:**
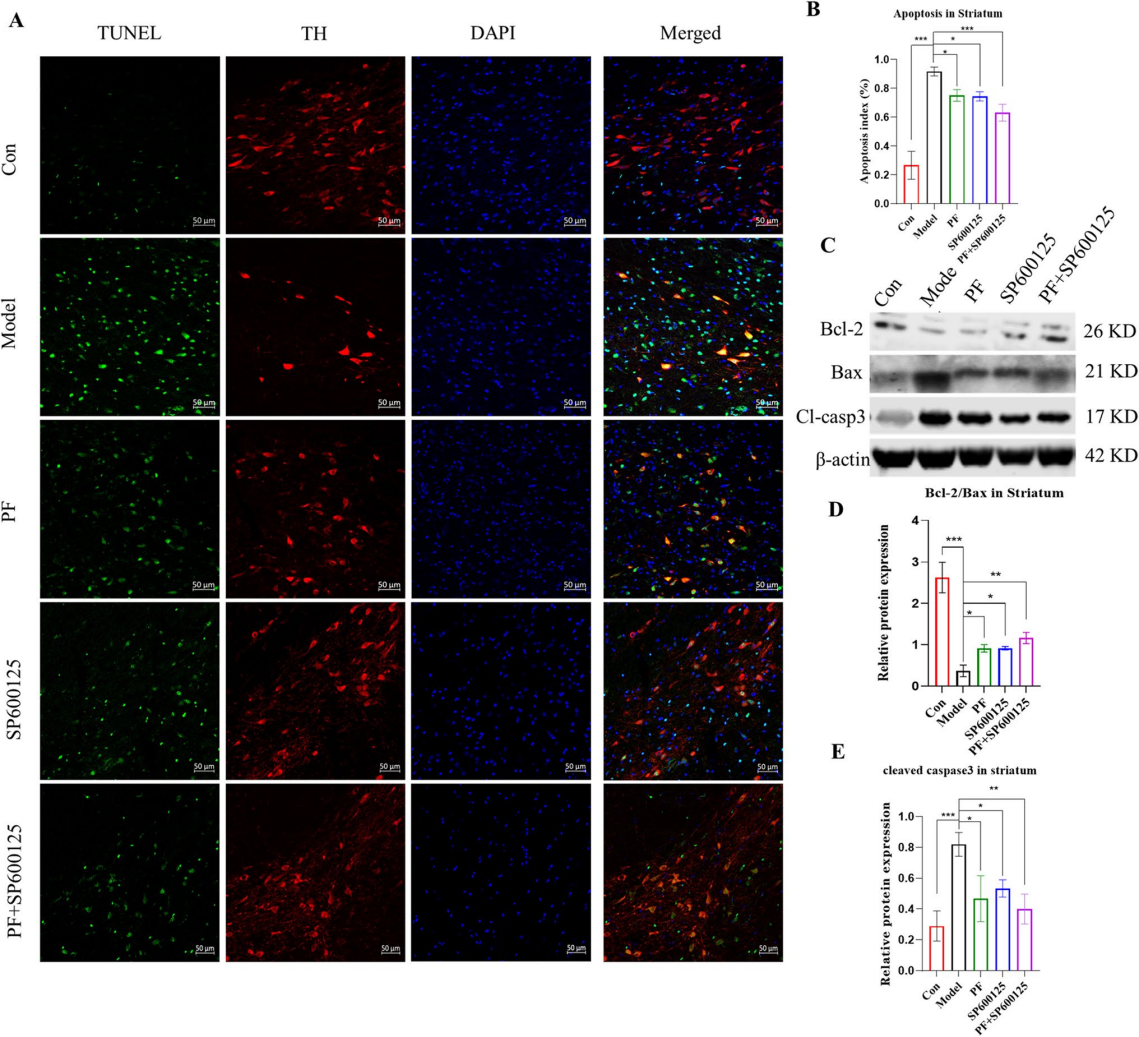
**PF attenuated MPTP induced cell apoptosis in the PD mice.** (**A**) TUNEL assay of the apoptotic neurons in the SNpc of mice. TUNEL (green), TH (red) and DAPI (blue), (Scale bar: 50 μm). (**B**) Apoptosis index of the SNpc in each group. (**C-D**) The expression level of the Bcl-2/Bax, Cl-casp3 protein were detected with Western Blot in the Striatum. β-actin served as control. Statistical analysis was performed with One-Way ANOVA, Turkey’s multiple comparison test post hoc, *n* = 3. Statistical analysis was performed with Two-Way ANOVA, *n* = 3. Significant differences were indicated by * *P* < 0.05, ** *P* < 0.01, *** *P* < 0.001.

TUNEL assay in the SNpc (TH: red, TUNEL: green, Figure 5A) revealed that the counts of apoptotic neurons in PD mice markedly decreased relative to control group after MPTP treatment (*p*=<0.001), while treatment with PF, SP600125, and PF+SP600125, reversed the percentage of apoptotic cells (*p* = <0.05, <0.05, and <0.001, respectively, Figure 5B).

Western blot revealed that the ratio of Bcl2/Bax protein was markedly reduced, while the levels of cleaved Caspase3 was significantly elevated in the striata of PD mice (*p* = <0.001 and <0.001, respectively; Figure 5C). Conversely, treatment with PF, SP600125, and PF+SP600125 enhanced the Bcl2/Bax protein ratio and reduced the level of cleaved Caspase3 (Bcl2/Bax: *p*=<0.05, <0.05, and <0.01, respectively; cleaved caspase3: *p* = <0.05, <0.05, and <0.01, respectively; Figure 5D-E). These results imply that PF suppresses MPTP-mediated apoptosis of substantia nigra neurons in PD mice.

#### PF improves hippocampal neuronal damage in the MPTP-induced PD mice brain

**Fig. 6:**
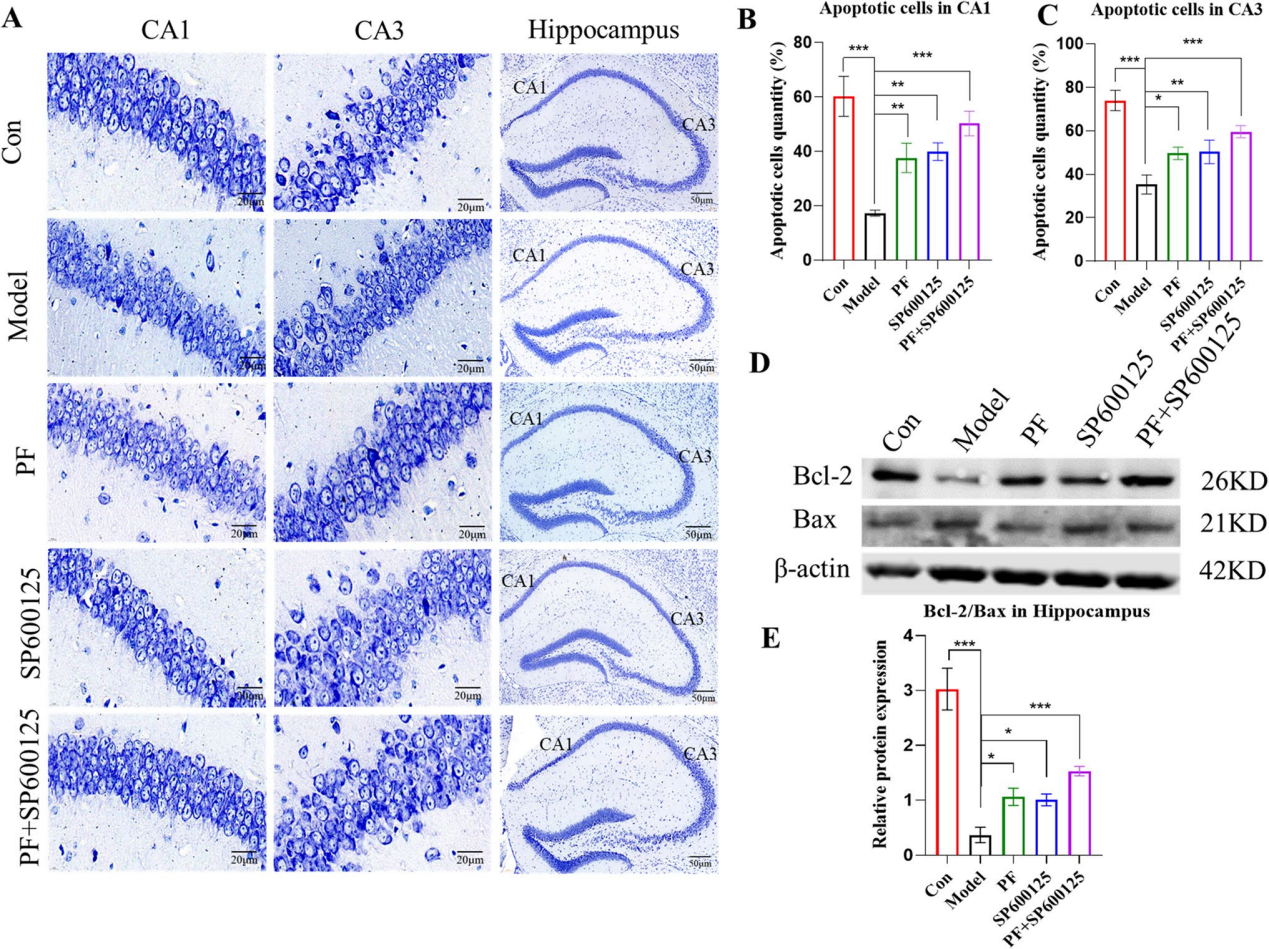
**Nissl staining was performed on sections from the hippocampus to determine neuronal survival.** (**A**) Typical photomicrographs of Nissl staining of the hippocampal CA1 and CA3 from the Control group, Model group and PF group. (**B**) The apoptotic cells quantity was calculated in the CA1 region of the hippocampus. (**C**) The apoptotic cells quantity was calculated in the CA3 region of the hippocampus. (**D-E**) The expression level of the Bcl-2/Bax protein was detected with Western Blot in the Striatum. β-actin served as control. Statistical analysis was performed with One-Way ANOVA, Turkey’s multiple comparison test post hoc, *n* = 3. Significant differences were indicated by * *P* < 0.05, ** *P* < 0.01, *** *P* < 0.001.

Nissl staining analysis of neuronal apoptosis in CA1 as well as CA3 hippocampal regions of PD mice revealed that PF enhanced neuronal survival in MPTP-treated mice (Figure 6A) and that hippocampal CA1 and CA3 levels in PD mice was markedly high, relative to control group (*p*<0.001, Figure 6B-C). However, apoptosis rates were markedly low in PD mice treated with PF, SP600125, and PF+SP600125 relative to untreated PD mice (CA1: *P* = <0.01, <0.01, and <0.001, respectively; CA3: *p* = <0.05, < 0.01, and <0.001, respectively).

Western blot analysis of Bax and Bcl-2 levels in the hippocampus revealed that the Bcl2/Bax ratio in PD mice was markedly high, relative to control group (*p* = <0.001), while treatment of PD mice with PF, SP600125, and PF+SP600125 markedly increased Bcl2/Bax levels relative to untreated PD mice (*p* = <0.05, <0.05, and <0.01, respectively, Figure 6D-E). Taken together, these data show that PF might suppress apoptosis in the hippocampal neurons of MPTP-induced PD mice.

#### PF attenuates Aβ formation and elevates synapse-related proteins in the hippocampus of MPTP-induced PD mice

**Fig. 7:**
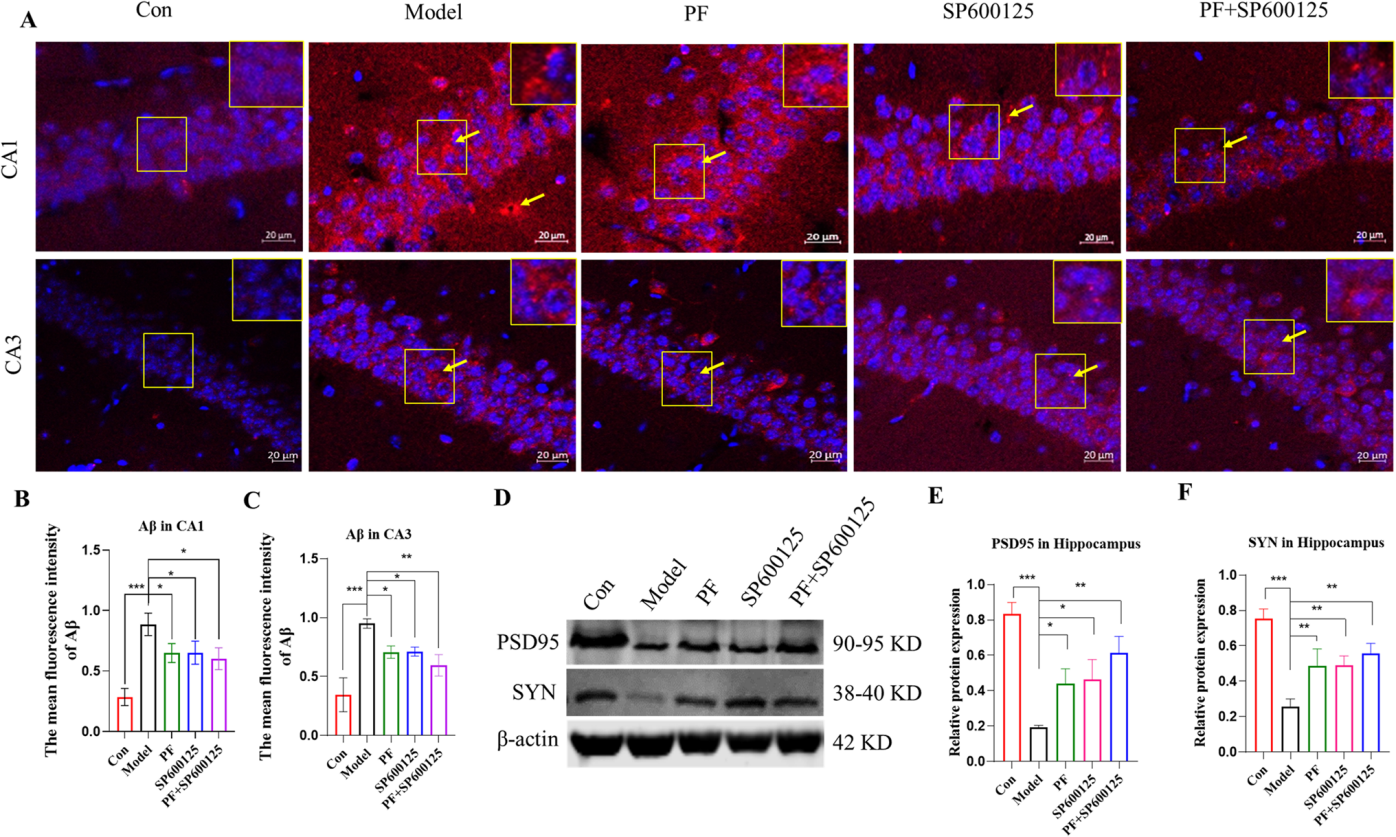
**Detection of the accumulation of Aβ and the expression of synaptic-related proteins.** (**A**) Immunofluorescent staining of Aβ (Red) and the DAPI (blue) in the CA1 and CA3 (scale bar = 20 μm). (**B-C**) Mean fluorescence intensity analysis for Aβ (*n* = 3, per group). (**D-F**) Expression of PSD-95, SYN were assesses by Western blot analysis. β-actin served as control. Statistical analysis was performed with One-Way ANOVA, Turkey’s multiple comparison test post hoc, *n* = 3. Significant diffrences were indicated by * *P* < 0.05, ** *P* < 0.01, *** *P* < 0.001.

Next, we used immunofluorescence (IF) to examine the effect of PF on hippocampal amyloid β (Aβ) levels (Figure 7A-C) and found that in the PD group, Aβ-positive puncta were mainly located in the CA1 and CA3, and that the Aβ protein signal was distributed in the cytoplasm. Moreover, the mean fluorescence intensity of Aβ signal in the CA1 and CA3 of PD mice was markedly high, relative to control group (*P*<0.001, *P*<0.001). Treating PD mice with PF, SP600125, and PF+SP600125 significantly reduced Aβ signal intensity in CA1 and CA3 when compared with untreated PD mice (CA1: all *p* = <0.05; CA3: *p* = <0.05, <0.05, and <0.01, respectively).

Next, western blotting was used to assess PSD95 and SYN levels (Figure 7D-F). Relative to the control group, levels of these proteins were markedly reduced in the hippocampus of PD mice (*P*<0.001, *P*<0.001). Treating PD mice with PF, SP600125, and PF+SP600125, significantly enhanced the protein levels of PSD95 and SYN, relative to untreated PD mice (PSD95: *p* = <0.05, <0.05, <0.01; SYN: all *p* = <0.01). Overall, the results suggest that the mean fluorescence intensity of Aβ was significantly decreased, while the level of synapse-associated proteins was significantly enhanced upon treating MPTP-induced PD mice with PF.

#### PF inhibited phosphorylation in the JNK/p53 signaling pathway, rescuing MPTP-induced apoptosis

**Fig. 8:**
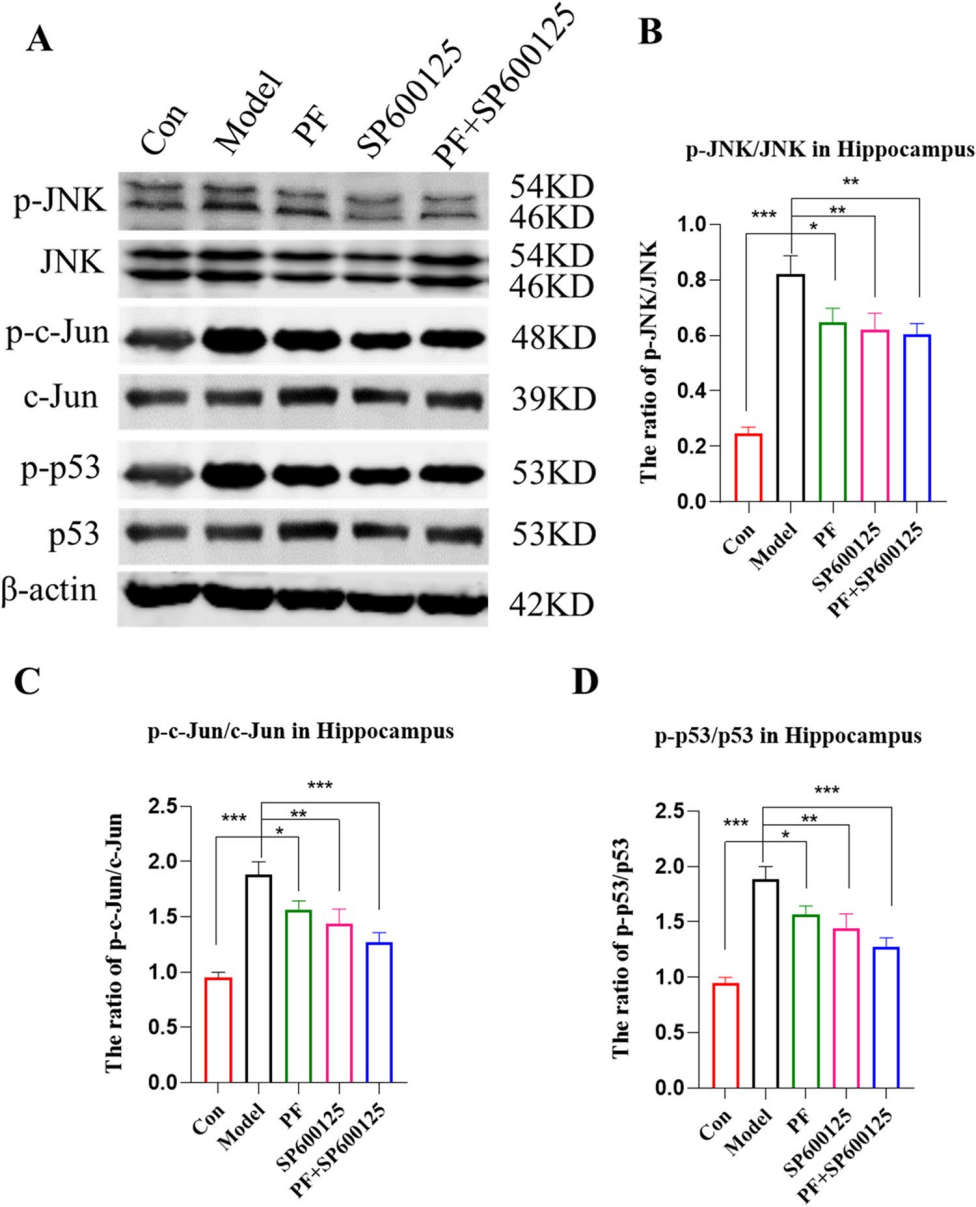
**Impact of PF on the phosphorylation of JNK/p53 pathway in MPTP-induced PD mice.** (**A-D**) Expression of p-JNK/JNK, p-cJun/c-Jun and p-p53/p53 proteins were assessed by Western blot analysis. β-actin served as control. Statistical analysis was performed with One-Way ANOVA, Turkey’s multiple comparison test post hoc, *n* = 3. Significant differences were indicated by * *P* < 0.05, ** *P* < 0.01, *** *P* < 0.001.

We used western blotting to assess the levels of p-c-Jun, JNK, Jun, p-p53, p-JNK, and p53 proteins in the hippocampus (*P*< 0.001, Figure 8A-D). PF treatment resulted in significantly increased p-c-Jun/Jun, p-JNK/JNK, as well as p-p53/p53 levels (all *p=*<0.05). Comparable findings were obtained after treatment with SP600125 and PF+SP600125 (p-JNK/JNK: *P*<0.01, *P*<0.01; p-c-Jun/Jun: *P*<0.01, *P*<0.001; p-p53/p53: *P*<0.01, *P*<0.001). These data strongly indicate that the JNK/p53 pathway influences apoptosis in the hippocampus of PD mice.

## Discussion

PD is a form of neurodegenerative disease that is associated with motor as well as non-motor symptoms (NMS). Clinically, CI is a common sign of NMS of PD (Baiano et al. 2020) and currently, there are no effective treatments. TCM appears to be a good treatment choice. PF, a biologically active compound extracted from *Paeonia lactiflora* Pallas, is commonly used in TCM. Using network analysis and experimental validation, we have uncovered the multi-target mechanisms underlying the effects of PF against PD. Using the databases TCMSP, Pharmapper and Sisstarget, we identified potential interaction targets. PD associated genes were identified on TTD, OMIM, Genecards, PharmGBK and DrugBank. CASP3 (caspase-3), TP53, MAPK8 (JNK), PSD95, and SYN were identified as core PPI network components. Integrated GO and KEGG analyses indicated that PF may exert its anti-PD-related cognitive effects via JNK signaling.

PF has numerous biological effects, including anti-inflammatory and immunoregulatory effects (Zhang and Wei 2020), anti-oxidation effects (Yuan et al. 2020) and anti-apoptosis effects (Wei et al. 2021). Moreover, PF is also reported to attenuate cognitive dysfunction in diabetic rats (Sun et al. 2017) and to delay neurodegeneration by reducing neuroinflammation, inhibiting internal, and external cell apoptosis, and improving motor and NMS via regulation of neurotransmitter levels in PD (Du et al. 2020). A previous study confirmed that PF exerts its effects against Aβ-induced neuroinflammation by inhibiting NF-κB signaling (Cho et al. 2020). PF is also reported to influence the development of neurodegeneration in AD by inhibiting neuroinflammation (Zhang et al. 2015). However, no studies have evaluated the therapeutic potential of targeting PF in the treatment of CI in PD. Here, we used molecular docking to identify proteins associated with CI in PD and found that JNK, p53, PSD95, and SYN had good binding affinity for PF, indicating that JNK is a target of PF. Our findings suggest that PF might have synergistic effects on the CI of PD. To evaluate this possibility, we assessed the effects of PF on a mouse model of PD established by intraperitoneally injecting MPTP, which is widely used to establish Parkinsonian mouse models (Rai et al. 2017; Rui et al. 2020). We employed well-recognized methods like the pole and open field test to evaluate the neurobehavioral functioning of PD mice. Neurodegenerative diseases that are attributed to aging, including PD, can lead to impaired neurological functions, particularly spatial memory (Lithfous et al. 2013). The MWMT is used to assess spatial memory formation and to evaluate spatial learning abilities (Fu et al. 2017; Vorhees and Williams 2006). However, the impact of the motor function of PD mice on the performance in MWMT is somewhat controversial. The learning and memory ability of PD rats were tested using the MWMT at 4 and 8 weeks after transplantation (Gu et al. 2012). McNamara’s study indicated that the advantage of the Y-maze test is that it is not as dependent on motor function as compared to other tests of memory (McNamara and Skelton 1993). In our study, we used MWMT to assess learning and memory in the PD mice. And the results revealed that PF improved learning and memory capacity in MPTP-induced PD mice. Loss of dopaminergic neurons in nigrostriatal pathway and regional or global brain volume are important trigger of CI in PD (Jokinen et al. 2009). Research indicated that motor deficits predicted CI in PD (Rochester et al. 2017). CI that are common in PD may limit the ability to compensate for gait disturbances, leading to further exacerbated gait impairments (Intzandt et al. 2018). Our analysis found that the climbing time in PF-treated mice was significantly shorter. The spontaneous exercise ability of the mice was also significantly enhanced. Additionally, PF enhanced the protein levels of TH and the number of DA neurons in nigrostriatal pathways of PD mice. Previous research had uncovered that JNK specific inhibitor SP600125 improved behavioral impairment, inhibited apoptosis of dopaminergic neurons, improved dopaminergic synaptic function in PD mice (Wang et al. 2004). These were validated in the present study. Nevertheless, our study went a step further by exploring investigating the effects of SP600125 on the CI in MPTP-induced PD. Comparable findings were obtained by inhibiting SP600125 with JNK, implying that PF exerts its protective effects against CI of PD via JNK pathway inhibition.

In recent years, apoptosis has emerged as a crucial factor in PD and AD pathogeneses (Mirzayans and Murray 2020; Yu et al. 2020; Paquet et al. 2018). Bcl-2 and Bax genes are apoptotic factors, with Bcl-2 inhibiting apoptosis and Bax promoting it (Narita et al. 1998). Caspase-3 is an effector of apoptosis (Wang et al. 2021). Assessment of the mechanism involved in DA neuronal increase, improved learning, and enhanced memory in PD mice revealed that PF reduced TUNEL positive cells in SNpc and increased the Bcl-2/Bax protein ratio in the striatum. Moreover, PF enhanced neuronal survival in hippocampal CA1 as well as CA3 regions, and increased the ratio of hippocampal Bcl-2/Bax proteins, similar observation upon JNK inhibition. The results of the present study revealed that the occurrence of neuronal apoptosis in the hippocampus, which was alleviated by the inhibition of JNK expression, suggested that PF inhibits apoptosis in the hippocampus of PD mice by inhibiting JNK signaling.

Based on the aforementioned results, we will explore the molecular mechanisms involved in the effects of PF on PD-associated CI. There is strong evidence that the degree of amyloid plaque pathology is an important cause of dementia in PD patients (Luo et al. 2020). A past study has shown that synaptic plasticity also plays a vital role in hippocampal learning and memory functions (Kurioka et al. 2021). Synaptogenesis is considered necessary in learning and memory. During neural development, PSD-95 is crucial for synaptic plasticity, glutamate transmission and dendritic spine morphogenesis (Coley and Gao 2018; Park et al. 2020). Thus, during development, PSD-95 dysfunction may lead to synaptic malformations associated with nervous system disorders. SYN is an important marker of synaptogenesis. Mounting evidence indicates that SYN is important in hippocampal-dependent cognition, anxiety and depression-related behaviors (Dandi et al. 2018). Through CA1 activity, the hippocampus is prone to degenerative lesions (Benito et al. 2018). The hippocampal CA3 neurons that drive cognitive function mainly affect spatial and associative learning, but they also affect working and reference memory (Xiao et al. 2021). Here, we find that PF exerts it protective effects in PD mice by reducing the levels of Aβ in CA1 and CA3 regions, and increasing hippocampal levels of SYN and PSD95.

JNK significantly influences behavior, cognition, and synaptic plasticity (Biggi et al. 2017). However, the role of JNK in resistance to CI in PD is rarely reported. In this study, we predict and analyze the potential mechanisms of CI in PD from the perspective of systematic network pharmacology method. GO and KEGG analyses indicated that JNK signaling may serve an important role in CI in PD. p53 has critical roles in senescence and apoptosis (Rufini et al. 2013) and its disruption affects cell proliferation and migration, which are involved in neurodegenerative diseases (Steffens Reinhardt et al. 2020). p53 is reported to regulate neurite growth and axon regeneration (Di Giovanni 2006). JNK signaling activates p53 signaling (Zyuz’kov et al. 2015, Akhter et al. 2015). It is reported that JNK/p53 signaling is involved in AD pathogenesis (Shi et al. 2020). Furthermore, p53 phosphorylation is needed for apoptosis activation by JNK signaling (Hua et al. 2019). Our data show that PF decreased the hippocampal levels of p-JNK/JNK, p-c-Jun/c-Jun, and p-p53/p53. We also show that combined treatment with PF+SP600125 markedly suppressed JNK, indicating potential synergistic effects between PF and SP600125.

## Conclusion

Based on network pharmacology and experimental validation, we show that protective effects of PF against CI in PD are mediated by JNK/p53 inhibition. This study has potential limitation. Further studies are needed to characterize anti-inflammatory effects of PF in CI of PD. For instance, we use LPS-induced PD model was used as a positive control. Further detection of serum inflammatory factors and the expression of microglia and astrocytes, such as GFAP and Iba1 in striatum and substantia nigra in our future study. Nevertheless, Our data offer insights into novel potential therapeutic strategies against CI.

### Supplementary Information

Below is the link to the electronic supplementary material.

Supplementary file1 (PDF 1598 KB)

## Declarations

### Data availability statement

All data underlying this study are available from the corresponding author upon request.

Animal protocols were permitted by the Experimental Animal Ethics Committee of Shanghai University of Traditional Chinese Medicine.
