# Correction to: Paeoniforin ameliorates cognitive impairment in Parkinson’s disease via JNK/p53 signaling

**DOI:** 10.1007/s11011-023-01300-9

**Published:** 2023-10-07

**Authors:** Zhu-qing He, Peng-fei Huan, Li Wang, Jian-cheng He

**Affiliations:** 1 Department of Diagnostics of Traditional Chinese Medicine, Shanghai University of Traditional Chinese Medicine, Shanghai, 201203 China; 2 Shanghai Municipal Hospital of Traditional Chinese Medicine, Shanghai University of Traditional Chinese Medicine, Shanghai, 200071 China; 3 Shanghai Key Laboratory of Health Identifcation and Assessment, School of Basic Medicine, Shanghai University of Traditional Chinese Medicine, Shanghai, 201203 China

**Correction to: Metabolic Brain Disease (2022) 37:1057–1070**

10.1007/s11011-022-00937-2

The Fig. 4, Fig. 5, Fig. 6, Fig.7 and Fig. 8 in the article were not the final version, the final version Fig. 4, Fig. 5, Fig. 6, Fig.7 and Fig. 8 are shown below.

**Fig. 4:**
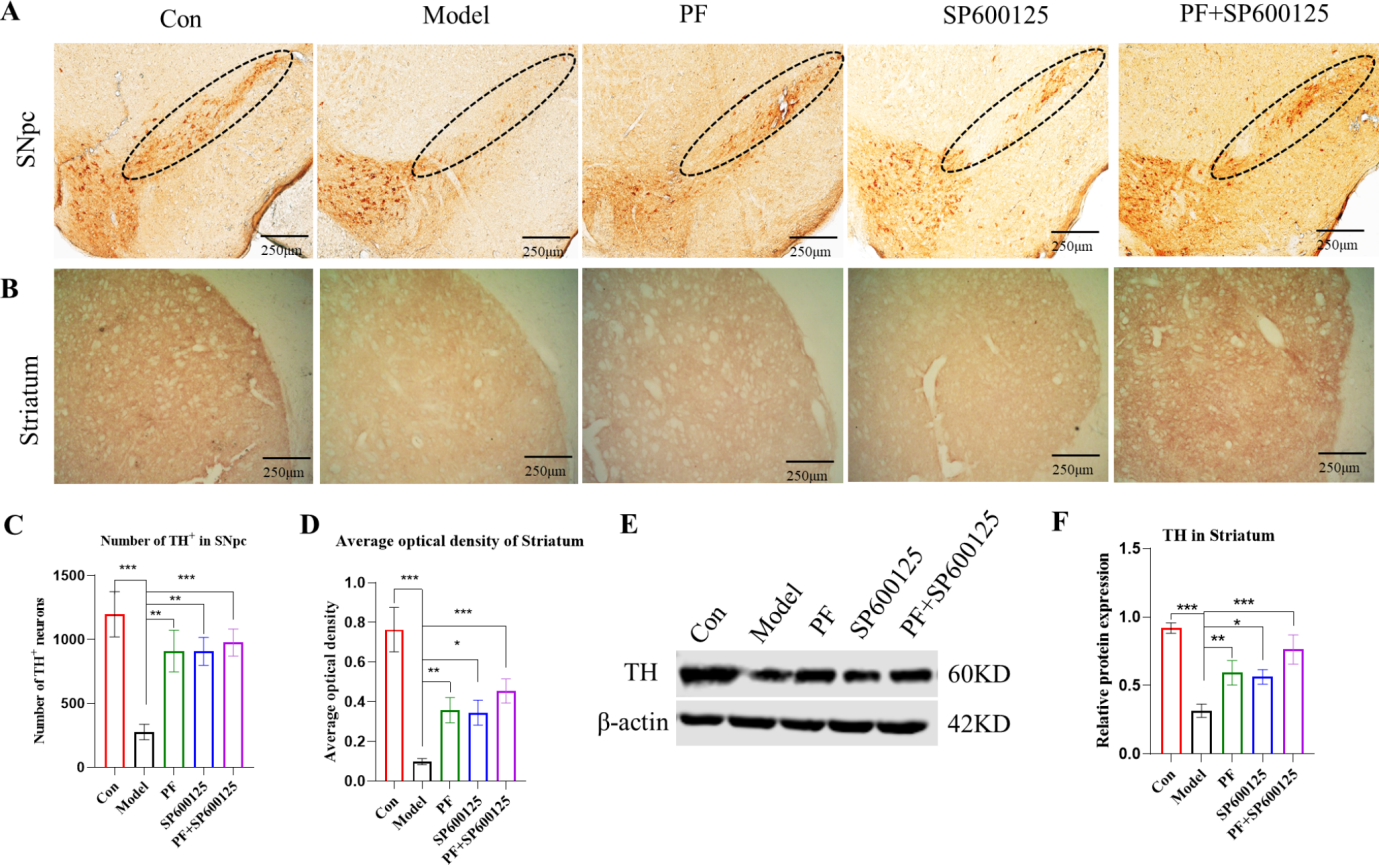
**PF attenuated MPTP induced loss of nigrostriatal DA neurons in the PD mice. (A-B)** DAB staining of TH on nigrostriatal of each group (Scale bar: 250 μm). **(C)** The counts of TH-positive cells of the SNpc. **(D)** Average optical density of the striatum of each group. **(E-F)** The expression level of TH proteins was detected with Western Blot in the Striatum, β-actin served as control. Statistical analysis was performed with One-Way ANOVA or Two-Way ANOVA, n = 3. Significant differences were indicated by * *P* < 0.05; ** *P* < 0.01; *** *P* < 0.001

**Fig. 5:**
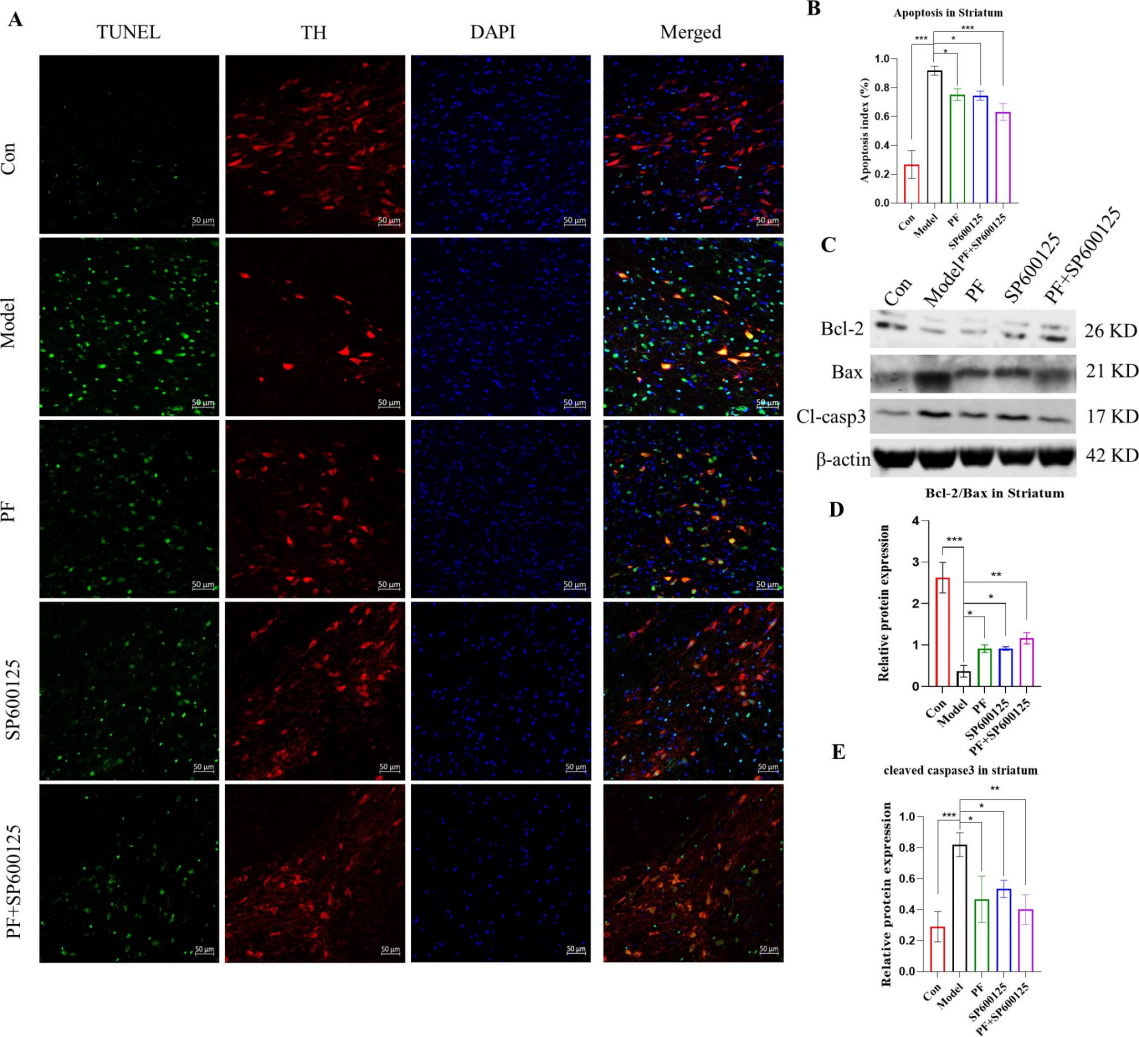
**PF attenuated MPTP induced cell apoptosis in the PD mice. (A)** TUNEL assay of the apoptotic neurons in the SNpc of mice. TUNEL (green), TH (red) and DAPI (blue), (Scale bar: 50 μm). **(B)** Apoptosis index of the SNpc in each group. **(C-E)** The expression level of the Bcl-2/Bax, Cl-casp3 protein were detected with Western Blot in the Striatum. β-actin served as control. Statistical analysis was performed with Two-Way ANOVA, n = 3. Significant differences were indicated by * *P* < 0.05, ** *P* < 0.01, *** *P* < 0.001

**Fig. 6:**
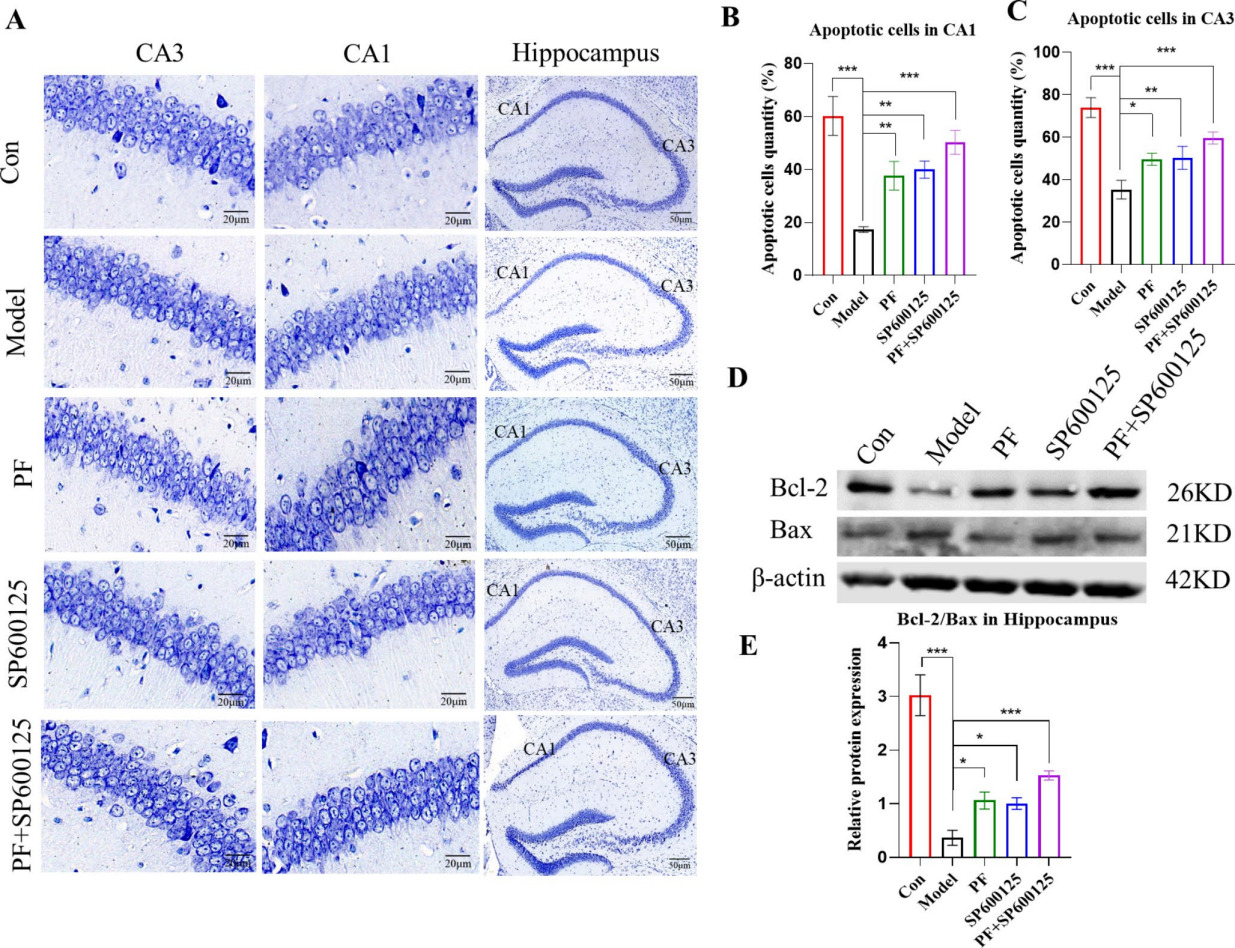
**Nissl staining was performed on sections from the hippocampus to determine neuronal survival. (A)** Typical photomicrographs of Nissl staining of the hippocampal CA1 and CA3 in each group. **(B)** The apoptotic cells quantity was calculated in the CA1 region of the hippocampus. **(C)** The apoptotic cells quantity was calculated in the CA3 region of the hippocampus. **(D-E)** The expression level of the Bcl-2/Bax protein was detected with Western Blot in the Striatum. β-actin served as control. Statistical analysis was performed with One-Way ANOVA, Turkey’s multiple comparison test post hoc, n = 3. Significant differences were indicated by * *P* < 0.05, ** *P* < 0.01, *** *P* < 0.001

**Fig. 7:**
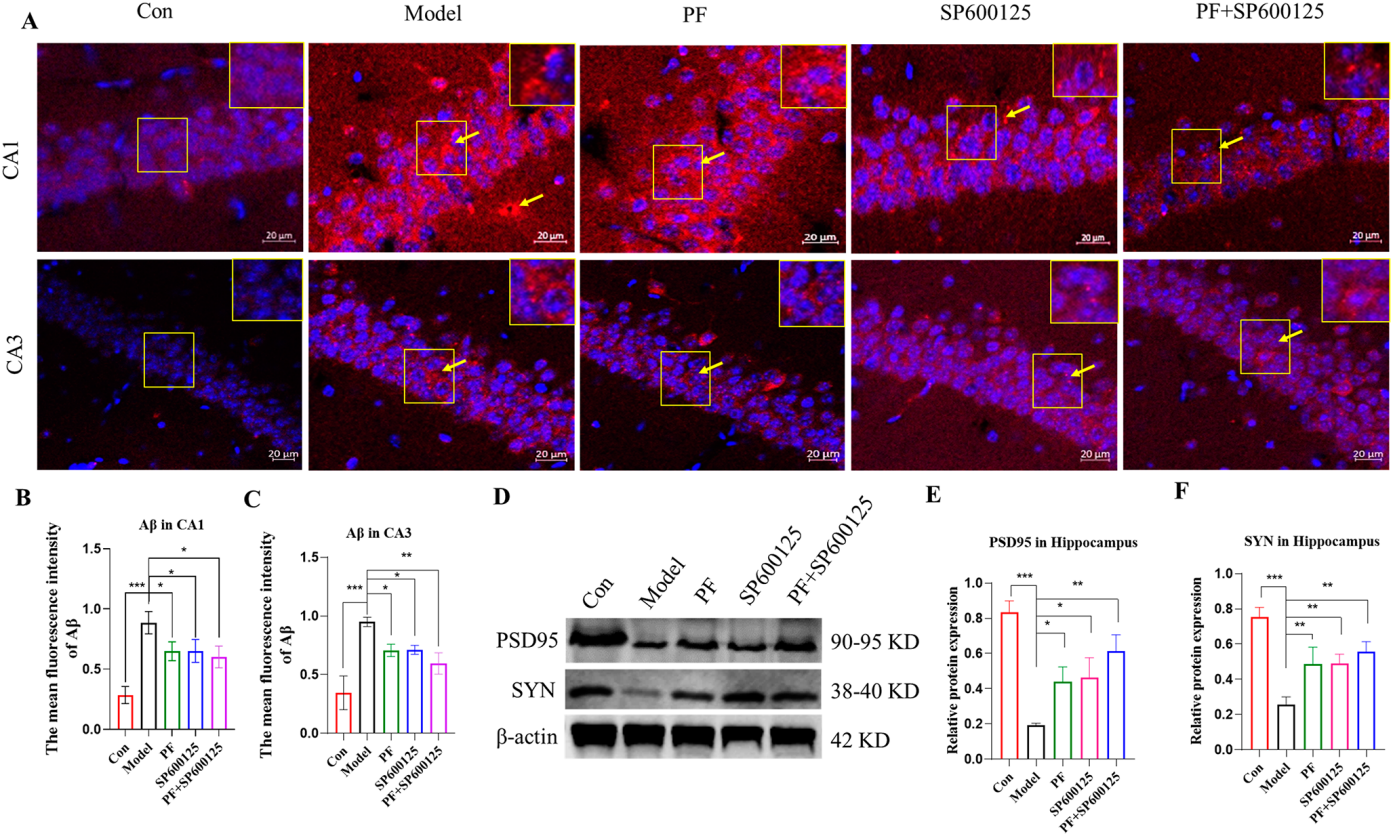
**Detection of the accumulation of Aβ and the expression of synaptic-related proteins. (A)** Immunofluorescent staining of Aβ (Red) and the DAPI (blue) in the CA1 and CA3 (scale bar = 20 μm). **(B-C)** Mean fluorescence intensity analysis for Aβ (n = 3, per group). **(D-F)** Expression of PSD-95, SYN were assessed by Western blot analysis. β-actin served as control. Statistical analysis was performed with One-Way ANOVA, Turkey’s multiple comparison test post hoc, n = 3. Significant differences were indicated by * *P* < 0.05, ** *P* < 0.01, *** *P* < 0.001

**Fig. 8:**
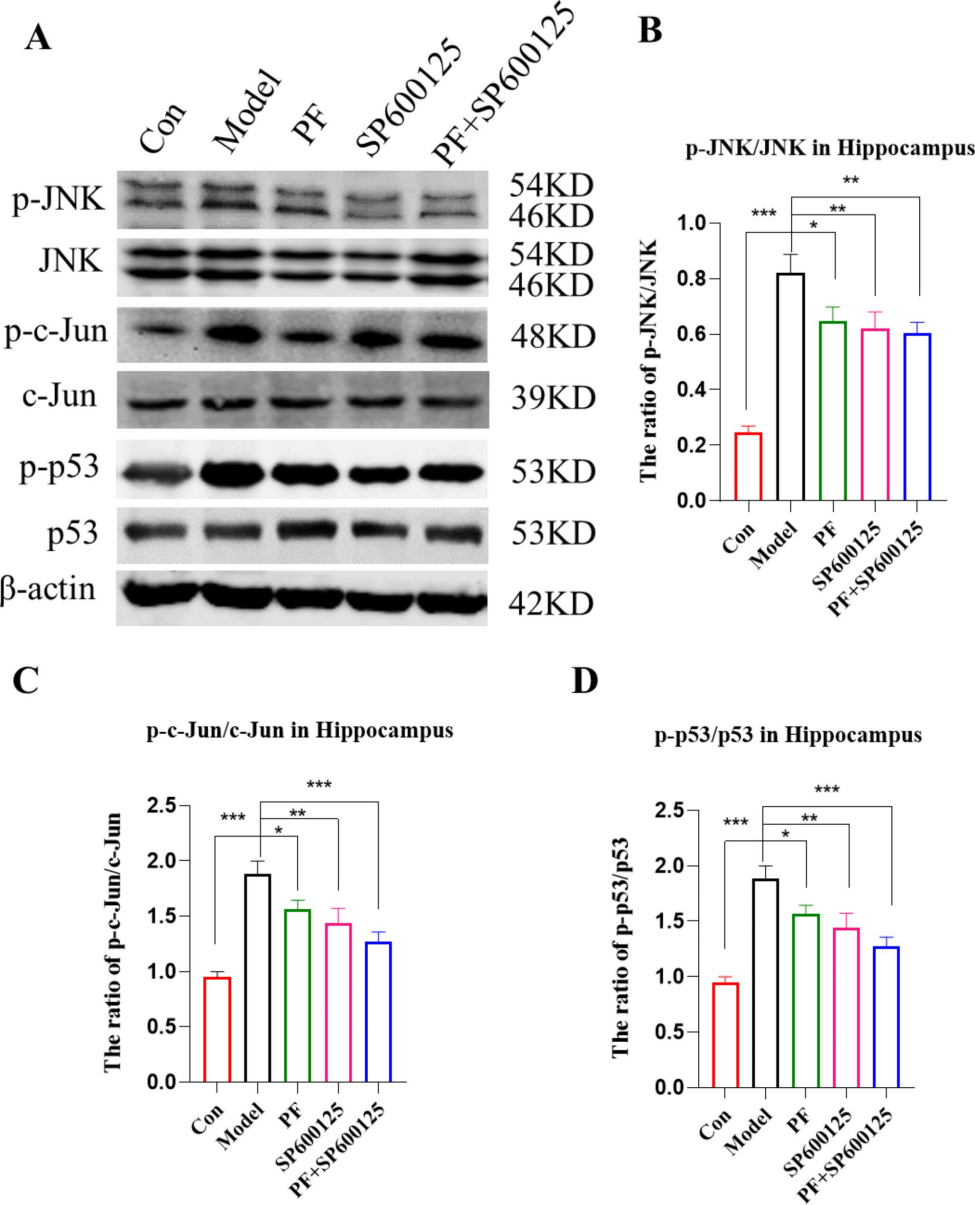
**Impact of PF on the phosphorylation of JNK/p53 pathway in MPTP-induced PD mice. (A-D)** Expression of p-JNK/JNK, p-c-Jun/c-Jun and p-p53/p53 proteins were assessed by Western blot analysis. β-actin served as control. Statistical analysis was performed with One-Way ANOVA, Turkey’s multiple comparison test post hoc, n = 3. Significant differences were indicated by * *P* < 0.05, ** *P* < 0.01, *** *P* < 0.001

