# Supplementary material for: Paeoniflorin ameliorates cognitive impairment in Parkinson’s disease via JNK/p53 signaling
Source: Metab Brain Dis. 2022 Mar 1;37(4):1057–70. doi: 10.1007/s11011-022-00937-2 (PMC9042992; doi:10.1007/s11011-022-00937-2)
Supplement: Supplementary file 1 — Supplementary file1 (PDF 1598 KB) [file 11011_2022_937_MOESM1_ESM.pdf]

Ligand:
10200440
4

Pharma Mc	Num Featu	Fit	Norm Fit	Num Hydrc	Num HB Ac	Num HB Dc	Num Positi	Num Negat
3bhq_B_ca	5	4.966	0.9931	3	2	0	0	0
2et1_A_cav	5	4.898	0.9795	4	1	0	0	0
1x66_A_ca	5	4.887	0.9773	3	1	1	0	0
2dk2_A_ca	5	4.875	0.9751	4	1	0	0	0
1buc_B_ca	5	4.874	0.9749	3	1	1	0	0
2hjs_A_cav	5	4.853	0.9706	4	1	0	0	0
1aei_D_cav	5	4.834	0.9668	3	0	2	0	0
1y79_1_ca	5	4.825	0.965	3	1	1	0	0
3eg9_B_ca	5	4.813	0.9625	3	1	1	0	0
1odh_A_ca	5	4.809	0.9619	4	1	0	0	0
2h63_A_ca	5	4.806	0.9611	4	1	0	0	0
2i4c_A_cav	5	4.805	0.961	3	1	1	0	0
1sqp_D_ca	5	4.802	0.9603	3	0	2	0	0
2wa0_A_ca	5	4.801	0.9602	4	0	1	0	0
1uf2_E_cav	5	4.798	0.9596	3	1	1	0	0
1z4z_A_cav	5	4.792	0.9584	3	1	1	0	0
1ggt_A_cav	5	4.777	0.9555	4	1	0	0	0
1kpi_A_cav	5	4.761	0.9521	3	1	1	0	0
1l0v_P_cav	5	4.744	0.9488	5	0	0	0	0
2g18_C_ca	5	4.741	0.9483	4	1	0	0	0
1ivx_B_cav	5	4.741	0.9482	4	1	0	0	0
2fw2_C_ca	6	5.682	0.947	4	0	2	0	0
1wp1_B_ca	5	4.735	0.947	4	1	0	0	0
2bty_A_cav	5	4.729	0.9458	5	0	0	0	0
2f5j_A_cav	5	4.726	0.9451	4	0	1	0	0
1v9s_A_cav	5	4.72	0.944	4	1	0	0	0
1ykd_A_ca	5	4.714	0.9427	3	1	1	0	0
1bcp_B_ca	6	5.545	0.9242	4	1	1	0	0
1nvm_A_ca	6	5.514	0.9189	4	1	1	0	0
3epm_B_ca	6	5.365	0.8941	4	1	1	0	0
2cpt_A_cav	6	5.241	0.8735	4	1	1	0	0
1wi5_A_ca	6	5.105	0.8508	4	1	1	0	0
1y64_B_ca	8	6.776	0.847	6	1	0	0	1
3g3o_A_ca	6	5.064	0.8441	4	1	1	0	0
1gk9_B_cav	7	5.878	0.8397	4	1	1	0	1
2jgd_A_cav	7	5.855	0.8364	4	0	2	0	1
1d7c_A_ca	6	4.996	0.8326	4	2	0	0	0
1x65_A_ca	6	4.995	0.8325	4	1	0	0	1
2dmy_A_ca	6	4.99	0.8316	3	3	0	0	0
2ex8_A_ca	6	4.985	0.8308	3	2	0	0	1
1ovq_A_ca	6	4.98	0.8299	4	2	0	0	0
1jv1_A_cav	6	4.967	0.8278	3	1	1	1	0

1fwi_C_cav	6	4.96	0.8266	3	0	2	1	0
1puj_A_cav	6	4.96	0.8266	3	1	1	0	1
2qvv_A_ca	7	5.783	0.8261	4	1	1	0	1
3e7o_B_ca	6	4.956	0.8259	3	1	1	0	1
1k44_E_cav	6	4.948	0.8246	4	1	0	0	1
1tz7_A_cav	6	4.943	0.8238	3	1	1	1	0
2f2f_C_cav	6	4.94	0.8233	4	1	1	0	0
2gjr_A_cav	6	4.924	0.8206	3	1	1	1	0
1d5b_B_ca	6	4.922	0.8204	5	1	0	0	0
1q1h_A_ca	6	4.911	0.8185	3	1	1	1	0
1tu9_A_cav	6	4.907	0.8178	4	1	0	0	1
2k3r_A_cav	6	4.907	0.8178	4	1	1	0	0
2e2e_A_ca	6	4.898	0.8164	4	0	2	0	0
3g73_B_ca	6	4.888	0.8147	5	0	1	0	0
1lpj_A_cav	6	4.887	0.8145	3	0	2	1	0
1pvn_B_ca	6	4.881	0.8135	4	1	1	0	0
3etu_A_cav	6	4.88	0.8134	4	1	1	0	0
1jw2_A_ca	6	4.878	0.813	5	0	1	0	0
1k9k_A_ca	6	4.878	0.8129	4	0	1	1	0
2gu0_B_ca	6	4.872	0.812	5	1	0	0	0
2enj_A_cav	6	4.871	0.8118	4	2	0	0	0
2oay_A_ca	6	4.869	0.8115	4	0	1	1	0
2bed_A_ca	6	4.866	0.8109	3	2	1	0	0
2a7w_C_ca	6	4.863	0.8105	5	1	0	0	0
1jx1_C_cav	6	4.862	0.8104	4	2	0	0	0
2k3s_A_cav	6	4.862	0.8103	4	0	2	0	0
2nmp_C_ca	6	4.86	0.81	4	1	1	0	0
1s6i_A_cav	6	4.857	0.8095	5	0	1	0	0
1qwo_A_ca	6	4.856	0.8094	3	2	0	0	1
2rkj_J_cavi	6	4.854	0.809	5	1	0	0	0
2o3j_A_cav	6	4.845	0.8075	4	1	1	0	0
1omo_B_ca	6	4.845	0.8075	4	0	2	0	0
2hma_A_ca	6	4.843	0.8071	3	2	1	0	0
1ahs_C_cav	6	4.842	0.807	3	2	1	0	0
1zag_D_cav	6	4.841	0.8068	4	1	0	1	0
1uld_D_cav	6	4.839	0.8065	4	1	1	0	0
2ejr_A_cav	6	4.838	0.8064	3	2	1	0	0
2ys5_A_cav	6	4.837	0.8061	4	1	1	0	0
3euh_E_ca	6	4.835	0.8059	4	1	1	0	0
1zpu_E_cav	6	4.83	0.8049	3	1	1	1	0
2aff_A_cav	6	4.829	0.8048	4	1	1	0	0
3gbx_B_cav	6	4.827	0.8046	4	0	2	0	0
3dwl_H_ca	6	4.827	0.8046	3	1	2	0	0
1spi_B_cav	6	4.827	0.8045	4	0	2	0	0
1f5z_A_cav	6	4.826	0.8044	4	2	0	0	0
1j5w_A_ca	6	4.82	0.8034	3	2	1	0	0
2qxx_A_cav	6	4.819	0.8032	4	1	1	0	0

1asq_A_cav	6	4.818	0.803	4	1	1	0	0
2ql6_E_cav	6	4.815	0.8025	4	1	1	0	0
2iuk_B_cav	7	5.614	0.802	5	1	0	1	0
1n07_B_ca	6	4.812	0.802	4	0	1	0	1
1oxy_A_ca	6	4.81	0.8017	4	1	1	0	0
1srp_A_cav	6	4.807	0.8012	4	1	1	0	0
1sp3_A_ca	6	4.807	0.8011	4	1	1	0	0
1yf6_L_cav	6	4.804	0.8006	4	1	0	1	0
1w6j_A_ca	6	4.8	0.8	3	2	1	0	0
3bxw_A_ca	6	4.796	0.7994	4	1	1	0	0
2qi2_A_cav	6	4.796	0.7993	4	1	1	0	0
2v4j_E_cav	6	4.795	0.7992	4	1	1	0	0
2vr2_A_cav	6	4.793	0.7988	3	2	1	0	0
2gb5_B_ca	6	4.791	0.7985	3	2	1	0	0
1ibh_A_cav	6	4.791	0.7984	3	3	0	0	0
1ken_T_cav	6	4.789	0.7981	4	0	2	0	0
2aya_A_ca	6	4.788	0.7979	3	2	1	0	0
2z3x_A_cav	6	4.787	0.7978	5	1	0	0	0
2a5m_A_ca	6	4.786	0.7977	3	0	2	0	1
1ako_A_ca	6	4.786	0.7977	4	2	0	0	0
1s99_B_cav	6	4.786	0.7977	4	0	2	0	0
1l6s_A_cav	6	4.786	0.7976	2	2	1	1	0
1s68_A_ca	6	4.781	0.7969	3	2	1	0	0
1d0l_A_cav	6	4.779	0.7965	4	1	1	0	0
1u2c_A_ca	6	4.774	0.7957	4	1	1	0	0
2gsq_A_cav	6	4.773	0.7955	5	0	1	0	0
1s4f_C_cav	6	4.769	0.7948	3	1	1	1	0
1sgm_B_ca	6	4.768	0.7947	4	1	0	1	0
1ajr_B_cav	6	4.762	0.7937	3	1	1	1	0
2j6h_A_cav	6	4.762	0.7937	3	2	1	0	0
1zxn_B_cav	6	4.762	0.7937	3	2	1	0	0
2coa_A_ca	6	4.76	0.7934	4	1	1	0	0
2rcv_G_cav	6	4.759	0.7931	4	0	1	1	0
1igw_B_cav	6	4.753	0.7922	5	1	0	0	0
3cdk_B_cav	6	4.753	0.7922	4	1	0	1	0
2d2m_A_ca	6	4.752	0.7919	4	0	1	0	1
1iqc_C_cav	6	4.75	0.7916	4	1	1	0	0
1x5n_A_ca	6	4.749	0.7915	5	1	0	0	0
2pff_A_cav	6	4.749	0.7914	3	2	1	0	0
3a0h_B_ca	6	4.748	0.7914	5	0	1	0	0
1s9c_F_cav	6	4.748	0.7913	5	0	0	1	0
1bq4_C_ca	6	4.748	0.7913	3	1	1	1	0
1fe8_J_cav	6	4.744	0.7907	3	2	1	0	0
2eje_A_cav	6	4.739	0.7898	3	2	1	0	0
1aii_A_cavi	6	4.738	0.7896	4	1	1	0	0
2cyc_A_cav	7	5.526	0.7894	5	0	1	1	0
3d47_B_ca	6	4.735	0.7891	4	1	1	0	0

1p4t_A_cav	6	4.734	0.789	5	1	0	0	0
2okq_B_ca	6	4.734	0.789	4	0	1	1	0
1wuk_L_ca	6	4.733	0.7888	4	0	2	0	0
3e4e_A_ca	6	4.726	0.7877	5	0	1	0	0
2qbv_A_ca	6	4.722	0.7871	5	0	1	0	0
2jpe_A_cav	6	4.721	0.7869	5	1	0	0	0
1hyh_C_ca	6	4.721	0.7868	3	2	0	0	1
3c7g_A_cav	6	4.721	0.7868	4	1	0	1	0
2ak4_I_cav	6	4.719	0.7866	3	2	1	0	0
1ddi_A_cav	6	4.719	0.7865	3	2	1	0	0
2uzf_A_cav	6	4.717	0.7862	4	2	0	0	0
1qgk_A_ca	6	4.715	0.7859	4	1	1	0	0
3dh4_D_ca	6	4.714	0.7857	6	0	0	0	0
3ibv_B_cav	6	4.714	0.7856	5	1	0	0	0
1spg_A_cav	9	7	0.7778	7	0	1	0	1
1lrz_A_cav	7	5.389	0.7699	4	0	2	1	0
1lln_A_cav	7	5.37	0.7672	4	1	1	1	0
2ron_A_cav	7	5.309	0.7584	4	1	1	0	1
2ffa_A_cav	7	5.309	0.7584	4	0	2	1	0
1wfi_A_cav	8	6.058	0.7572	4	2	1	0	1
1yi5_I_cavi	7	5.296	0.7565	5	0	1	1	0
1j7x_A_cav	7	5.294	0.7563	4	1	1	1	0
2e74_A_ca	7	5.281	0.7545	5	1	0	0	1
2ee4_A_ca	8	5.986	0.7482	5	2	0	0	1
2rmp_A_ca	7	5.228	0.7468	5	0	1	1	0
1inz_A_cav	7	5.224	0.7462	4	1	1	1	0
2c2c_A_cav	9	6.696	0.744	6	1	1	0	1
1mg5_B_ca	8	5.941	0.7427	5	1	1	1	0
1pkq_G_ca	7	5.195	0.7421	5	0	1	0	1
1wi3_A_ca	7	5.193	0.7419	4	1	1	0	1
3brp_A_cav	7	5.165	0.7378	4	0	2	1	0
1mje_A_ca	7	5.15	0.7357	5	1	1	0	0
1sjw_A_cav	8	5.879	0.7349	5	0	2	1	0
1xup_O_ca	7	5.14	0.7342	4	1	1	0	1
2vas_A_cav	7	5.138	0.734	4	1	1	0	1
1jqx_A_cav	7	5.107	0.7295	5	2	0	0	0
1kmh_A_ca	8	5.83	0.7287	6	0	2	0	0
1u4j_A_cav	7	5.09	0.7272	4	1	1	1	0
1s6d_A_cav	8	5.803	0.7254	5	0	2	0	1
1wxr_A_ca	8	5.767	0.7209	4	1	1	2	0
2ve7_C_cav	7	5.026	0.718	5	1	1	0	0
1vmi_A_ca	7	5.015	0.7165	4	1	1	0	1
3ecp_A_ca	7	5.005	0.715	6	0	1	0	0
1xk0_A_cav	7	5	0.7143	5	0	1	0	1
1u63_C_ca	7	5	0.7143	5	0	1	1	0
1xdt_T_cav	7	5	0.7143	5	0	1	1	0
2daf_A_cav	7	5	0.7143	5	1	0	0	1

1vmd_B_ca	7	4.998	0.714	4	1	1	0	1
2dpy_B_ca	7	4.993	0.7133	4	0	2	1	0
1u3r_B_cav	7	4.991	0.713	4	1	1	1	0
2i7x_A_cav	7	4.991	0.713	4	1	1	1	0
2r55_A_cav	7	4.99	0.7129	4	1	1	1	0
1v5w_B_ca	7	4.988	0.7126	4	0	1	2	0
2dlu_A_cav	7	4.982	0.7117	4	0	2	1	0
3e1k_I_cav	7	4.978	0.7112	4	1	1	1	0
1ntg_D_cav	7	4.977	0.7111	4	0	2	1	0
1onq_C_ca	7	4.976	0.7109	4	1	1	0	1
1ega_B_ca	7	4.976	0.7108	4	1	1	0	1
2w7n_B_ca	7	4.974	0.7106	4	0	2	0	1
1lqy_A_cav	7	4.972	0.7103	4	0	2	0	1
2cxq_A_cav	7	4.971	0.7102	4	1	1	1	0
1k1d_A_ca	7	4.971	0.7101	3	1	1	1	1
2vdg_A_ca	7	4.97	0.71	4	2	0	0	1
1uvx_A_ca	7	4.968	0.7097	5	1	0	0	1
1aih_C_cav	7	4.967	0.7095	4	1	1	1	0
2cog_B_cav	7	4.966	0.7094	3	0	3	0	1
1nj8_D_cav	7	4.963	0.709	4	1	1	1	0
1q8b_A_ca	7	4.963	0.709	4	0	1	1	1
1tu8_A_cav	11	7.798	0.7089	6	1	1	0	3
1m3s_A_ca	7	4.958	0.7083	4	1	1	0	1
1gyt_A_cav	7	4.957	0.7082	3	0	3	0	1
3glb_C_cav	7	4.951	0.7073	4	1	1	1	0
2eql_A_cav	7	4.951	0.7072	3	1	2	1	0
2qnc_B_ca	7	4.948	0.7068	6	1	0	0	0
2ch5_A_ca	7	4.947	0.7067	4	0	2	1	0
1r6u_A_cav	7	4.946	0.7065	4	1	1	0	1
1ugr_B_cav	7	4.944	0.7063	5	1	1	0	0
2hqm_A_ca	7	4.939	0.7055	4	0	2	0	1
2gvc_E_cav	7	4.935	0.705	5	2	0	0	0
2rf4_A_cav	7	4.927	0.7039	5	1	1	0	0
1t6l_A_cav	7	4.927	0.7039	5	2	0	0	0
1ch4_A_ca	9	6.326	0.7029	7	0	1	0	1
1rp8_A_cav	8	5.62	0.7025	4	1	1	2	0
3bxj_A_cav	7	4.905	0.7008	4	2	0	1	0
3eay_A_ca	7	4.9	0.7	3	1	1	0	2
2csj_A_cav	7	4.898	0.6997	4	1	1	1	0
1rhd_A_cav	8	5.596	0.6995	3	2	1	1	1
1emy_A_ca	10	6.994	0.6994	6	1	1	0	2
2hcb_B_ca	7	4.896	0.6994	4	0	2	0	1
2nzt_B_cav	7	4.896	0.6994	5	2	0	0	0
1tw2_B_ca	7	4.895	0.6993	3	0	3	1	0
2f66_E_cav	7	4.893	0.699	4	0	2	1	0
2ziv_B_cav	7	4.892	0.6988	5	0	2	0	0
2ju0_A_cav	7	4.89	0.6986	6	0	1	0	0

1nhy_A_ca	7	4.89	0.6986	5	2	0	0	0
3blv_A_cav	9	6.285	0.6983	6	1	1	1	0
2eng_A_ca	7	4.884	0.6978	3	1	2	1	0
3epy_B_ca	7	4.881	0.6973	6	1	0	0	0
6req_D_ca	7	4.88	0.6971	4	2	0	0	1
2vwt_A_ca	9	6.271	0.6968	6	2	0	0	1
3gan_A_ca	7	4.876	0.6966	5	0	2	0	0
2vyc_I_cav	7	4.875	0.6965	3	1	2	0	1
1n60_B_ca	7	4.873	0.6961	5	2	0	0	0
2oeq_C_ca	7	4.873	0.6961	4	1	1	1	0
1mqv_B_ca	7	4.872	0.696	5	2	0	0	0
1w36_F_ca	7	4.869	0.6955	3	1	1	0	2
1hi6_B_cav	8	5.564	0.6954	4	1	2	1	0
1b0n_B_ca	7	4.866	0.6951	6	1	0	0	0
3czm_A_ca	7	4.865	0.695	4	1	1	0	1
1nex_C_ca	7	4.864	0.6949	5	1	1	0	0
2qtz_A_cav	7	4.863	0.6947	5	1	1	0	0
2k8y_A_ca	7	4.863	0.6947	6	1	0	0	0
2qqh_A_ca	7	4.862	0.6946	4	0	2	0	1
2hzv_C_cav	7	4.857	0.6939	5	2	0	0	0
1cz2_A_cav	7	4.851	0.6931	6	0	1	0	0
1prx_A_cav	7	4.851	0.693	6	1	0	0	0
1orr_A_cav	7	4.847	0.6924	4	1	1	1	0
1yiv_A_cav	8	5.538	0.6923	4	2	0	2	0
2v91_A_ca	7	4.844	0.692	4	0	2	1	0
1f0x_A_cav	7	4.839	0.6912	3	2	0	1	1
2an7_A_ca	7	4.838	0.6912	4	0	2	0	1
1d5y_D_ca	8	5.528	0.691	4	2	0	1	1
1r19_A_cav	7	4.837	0.691	5	1	1	0	0
1e6b_A_ca	7	4.835	0.6907	5	1	1	0	0
1bj1_H_cav	7	4.834	0.6906	3	3	0	0	1
1xqv_A_ca	7	4.834	0.6906	5	0	2	0	0
1wch_A_ca	7	4.833	0.6904	5	1	1	0	0
1o7d_E_ca	7	4.832	0.6904	3	2	1	1	0
1i11_A_cav	7	4.832	0.6903	5	1	1	0	0
1eyr_A_cav	7	4.83	0.69	6	0	1	0	0
2qp4_A_ca	7	4.828	0.6898	4	0	2	0	1
1s5a_A_cav	7	4.824	0.6892	3	1	1	2	0
2gmj_B_ca	7	4.823	0.6891	4	1	2	0	0
1o8v_A_ca	7	4.822	0.6889	5	1	1	0	0
1t9g_S_cav	7	4.822	0.6889	3	0	2	1	1
1qbi_A_cav	7	4.821	0.6887	5	1	1	0	0
2dlz_A_cav	7	4.819	0.6884	3	2	0	2	0
1sq1_A_ca	7	4.818	0.6882	4	1	1	1	0
1lar_A_cav	7	4.817	0.6882	5	1	1	0	0
1nsl_B_cav	7	4.815	0.6878	5	1	1	0	0
1zdq_C_ca	7	4.812	0.6875	4	1	1	1	0

1kfi_B_cav	7	4.806	0.6865	4	2	1	0	0
1v8b_C_ca	7	4.803	0.6862	5	1	1	0	0
2h00_C_ca	7	4.802	0.6861	5	1	1	0	0
1bco_A_ca	8	5.482	0.6852	5	2	1	0	0
2nv4_A_ca	7	4.793	0.6846	5	1	1	0	0
1je5_A_cav	7	4.791	0.6844	4	3	0	0	0
1tzp_A_cav	7	4.79	0.6843	2	2	1	1	1
1peo_A_ca	8	5.47	0.6837	4	2	1	1	0
1mvf_D_ca	7	4.785	0.6836	5	0	2	0	0
1j9a_A_cav	7	4.784	0.6834	5	1	1	0	0
2o4c_A_ca	7	4.782	0.6832	3	3	1	0	0
3b68_A_ca	7	4.782	0.6831	5	0	1	0	1
1wth_D_ca	7	4.778	0.6825	5	1	1	0	0
1veg_A_ca	7	4.777	0.6825	5	1	1	0	0
1wgw_A_c	7	4.772	0.6818	6	1	0	0	0
2qtv_A_cav	7	4.771	0.6816	3	1	1	0	2
1rkh_A_cav	10	6.812	0.6812	6	1	1	0	2
1uaa_B_ca	7	4.766	0.6809	4	0	2	1	0
1vlu_B_cav	7	4.764	0.6805	6	1	0	0	0
3el6_A_cav	7	4.763	0.6804	5	2	0	0	0
2afj_A_cav	7	4.759	0.6799	4	1	1	1	0
2goy_G_ca	7	4.759	0.6798	5	2	0	0	0

Num Arom	Name	Class	Uniplot
0	NONE	NONE	NONE
0	Oxalate oxi	NONE	P45850
0	Friend leuk	SIGNALING	FLI1_HUMAN
0	Heterogen	NONE	O43390
0	Acyl-CoA d	NONE	Q06319
0	USG-1 prot	NONE	O87014
0	Annexin-B1	NONE	P26256
0	Peptidyl-di	NONE	P24171
0	Protein tra	NONE	Q15436
0	Chorion-sp	NONE	P70348
0	Biliverdin r	NONE	P53004
0	Bicarbonat	NONE	Q55460
0	Cytochrom	NONE	P31800
0	Melanoma	NONE	P43358
0	Major 114	NONE	P22472
0	Hemagglut	NONE	P04850
0	Coagulatio	BLOOD CO	F13A_HUMAN
0	Cyclopropa	NONE	P0A5P0
0	Fumarate r	OXIDORED	FRDA_ECOLI
0	Phycocyan	NONE	Q93TN0
0	Phenylethy	OXIDORED	PAOX_ARTGO
0	Testis-spec	NONE	Q9Y6F7
0	Outer mem	MEMBRAN	OPRM_PSEAE
0	Acetylgluta	NONE	Q9X2A4
0	Mortality f	NONE	Q9UBU8
0	Uracil phos	NONE	Q72J35
0	Adenylate	NONE	P94182
0	Pertussis to	NONE	P04977
0	4-hydroxy-	NONE	P51016
0	Thiamine b	NONE	Q9A6Q5
0	Vacuolar p	NONE	O75351
0	Protein RRI	NONE	Q14690
0	Actin, alph	NONE	P68135
0	Vacuolar tr	NONE	P43585
0	Penicillin G	NONE	P06875
0	2-oxogluta	NONE	P0AFG3
0	Cellobiose	NONE	Q01738
0	Cold shock	NONE	O75534
0	Spermatid	NONE	Q96SI9
0	D-alanyl-D-	NONE	P24228
0	Putative H	NONE	P0A8I1
0	UDP-N-ace	NONE	Q16222

0	Urease sub	NONE	P18316
0	Ribosome l	NONE	O31743
0	Fructose-1,	NONE	P00636
0	Mitogen-ac	TRANSFERA	MK09_HUMAN
0	Nucleoside	NONE	P84284
0	4-alpha-glu	NONE	O66937
0	Cytolethal	NONE	O87120
0	Glucan 1,4-	NONE	P19571
0	Ig kappa ch	NONE	P01834
0	Transcripti	NONE	Q980M5
0	Hypothetic	NONE	Q9HX49
0	Ribonuclea	NONE	Q8U0H6
0	Formate-de	NONE	Q8X5S3
0	Forkhead b	NONE	Q08050
0	Retinoid-bi	NONE	Q96R05
0	Inosine-5-n	NONE	P50097
0	Protein tra	NONE	P53847
0	Hemolysin	NONE	P0ACE3
0	Protein S10	SIGNALING	S10A6_HUMAN
0	Non-struct	NONE	Q9PY93
0	Protein kin	TRANSFERA	KPCT_HUMAN
0	Plasma pro	IMMUNE S	IC1_HUMAN
0	Protein far	NONE	Q04631
0	Phosphorib	NONE	Q7P0E6
0	Chalcone--	NONE	P28012
0	Smoothelin	NONE	Q99LM3
0	Cystathioni	NONE	P32929
0	Calcium-de	NONE	P28583
0	3-phytase A	NONE	O00092
0	Tyrosyl-tRN	NONE	P12063
0	UDP-glucos	NONE	Q19905
0	Ornithine c	NONE	O28608
0	tRNA-speci	NONE	Q97T38
0	Core protei	NONE	P36325
0	Zinc-alpha-	LIPID MOBI	ZA2G_HUMAN
0	Galectin-2	NONE	Q9P4R8
0	Lysine-spec	OXIDOREDU	LSD1_HUMAN
0	ALK tyrosin	SIGNALING	ALK_HUMAN
0	Chromosor	NONE	P60293
0	Iron transp	NONE	P38993
0	Antigen KI-	NONE	P46013
0	Serine hydr	NONE	P0A2E1
0	Actin-relate	NONE	P32390
0	Fructose-1,	NONE	P22418
0	N-acetylne	NONE	P44539
0	Glycyl-tRNA	NONE	Q9WY59
0	Deoxycytid	NONE	O07247

0	L-ascorbat	NONE	P37064
0	Nicotinami	SIGNALING	NRK1_HUMAN
0	Seed lipoxy	NONE	P24095
0	Riboflavin I	NONE	O74866
0	Hemocyani	NONE	P04253
0	Serralysin	NONE	P07268
0	Cytochrom	NONE	Q8E9W8
0	Reaction ce	NONE	P0C0Y7
0	Lanosterol	ISOMERASI	ERG7_HUMAN
0	Chitinase d	NONE	Q9BWS9
0	Protein pel	NONE	Q9HJ74
0	Sulfite redu	NONE	P45574
0	Dihydropyr	NONE	Q14117
0	NADH pyro	NONE	P32664
0	Superoxide	NONE	P00446
0	Hemagglut	NONE	P03438
0	DNA polym	NONE	P06710
0	Small, acid-	NONE	P02958
0	Beta-crysta	NONE	O35486
0	Exodeoxyri	NONE	P09030
0	Putative HI	NONE	O34911
0	Delta-amin	NONE	P0ACB2
0	RNA ligase	NONE	P32277
0	Membrane	NONE	P41052
0	Dystroglyca	NONE	Q62165
0	Glutathion	NONE	P46088
0	Genome pc	NONE	P19711
0	Uncharacte	NONE	P42105
0	Aspartate a	NONE	P00503
0	Glucosamir	NONE	P17169
0	DNA topois	ISOMERASI	TOP2A_HUMAN
0	Serine/thre	NONE	Q9BZL6
0	Superoxide	NONE	P54375
0	Isocitrate ly	NONE	P0A9G6
0	Probable su	NONE	P42315
0	Extracellula	NONE	Q7M419
0	Cytochrom	NONE	P55929
0	Harmonin	NONE	Q9Y6N9
0	Fatty acid s	NONE	P19097
0	Photosyste	NONE	P51765
0	Peroxisoma	NONE	P51659
0	Phosphogly	NONE	P00950
0	von Willebı	IMMUNE S	VWF_HUMAN
0	General tra	NONE	P78347
0	Annexin A3	CALCIUM/F	ANXA3_HUMAN
0	Tyrosyl-tRN	NONE	O58739
0	L-rhamnon	NONE	Q8ZNF9

0	Surface prc	NONE	Q9RP17
0	Uncharacte	NONE	P0AAQ9
0	Periplasmic	NONE	P21852
0	Cytochrom	OXIDOREDI	CP2E1_HUMAN
0	Uncharacte	NONE	P64767
0	Nuclear inh	NONE	Q8R3G1
0	L-2-hydroxy	NONE	P14295
0	Arabinoxyla	NONE	Q45071
0	HLA class I	IMMUNE S	1B35_HUMAN
0	Sulfite redu	NONE	P38038
0	Naphthoate	NONE	Q5HH38
0	Importin su	NONE	Q14974
0	Sodium/glu	TRANSPOR	SGLT_VIBPA
0	Exportin-T	NONE	O94258
0	Hemoglobi	NONE	P56250
0	Aminoacylt	NONE	P0A0A5
0	Antiviral pr	NONE	Q40772
0	Surfactin sy	NONE	Q08788
0	Alpha-hem	NONE	P08716
0	Nuclear mi	NONE	O35685
0	Acetylcholi	NONE	P58154
0	Retinol-bin	NONE	Q7SZI7
0	Cytochrom	NONE	P83791
0	Rho GTPase	NONE	Q13017
0	Mucorpeps	NONE	P00799
0	Epsin-1	NONE	Q9Y6I3
0	Cytochrom	NONE	P0C189
0	Alcohol del	NONE	P00334
0	Myelin-olig	NONE	Q63345
0	DNA-bindir	DNA BINDI	SATB2_HUMAN
0	C-phycocya	NONE	P00306
0	26S protea	NONE	P60896
0	Nogalonic a	NONE	Q9RN59
0	Glycerol kir	NONE	O34153
0	Myosin-VI	NONE	Q29122
0	Dihydroorc	NONE	Q53ZE5
0	ATP syntha	NONE	P06450
0	Phospholip	NONE	Q6SLM1
0	Albumin-8	NONE	P23110
0	Hemoglobi	NONE	O88093
0	Kinetochor	NONE	O14777
0	Ethanolami	NONE	P77218
0	Transposas	NONE	Q46731
0	Heme oxyg	OXIDOREDI	HMOX1_HUMAN
0	50S ribosor	NONE	P54050
0	Proheparin	COMPLEX (	HBEGF_HUMAN
0	IQ and ubic	NONE	Q8NA54

0	Methylglyo	NONE	Q9X0R7
0	Flagellum-s	NONE	P26465
0	Estrogen re	TRANSCRIP	ESR2_HUMAN
0	Cleavage ar	RNA BINDII	CPSF3_HUMAN
0	StAR-relate	NONE	Q9NSY2
0	Meiotic rec	NONE	Q14565
0	InaD-like pı	NONE	Q8NI35
0	Galactose/I	NONE	Q06433
0	Tyrosyl-tRN	NONE	P54577
0	T-cell surfa	IMMUNE S	CD1A_HUMAN
0	GTP-bindin	NONE	P06616
0	TrfB transc	NONE	P03052
0	Peptide de	NONE	O31410
0	Glucose-6-ı	NONE	P06745
0	D-hydantoi	NONE	Q45515
0	Aldose red	NONE	P23901
0	Globin LI63	NONE	Q08753
0	Integrase	NONE	P21442
0	Branched-c	TRANSFERA	BCAT1_HUMAN
0	Prolyl-tRNA	NONE	Q58635
0	Uncharacte	NONE	O31641
0	Glutathion	NONE	P46427
0	3-hexulose	NONE	P42404
0	Cytosol am	NONE	P68767
0	HTH-type t	NONE	P07774
0	Lysozyme (	NONE	P11376
0	Recombina	NONE	P13340
0	N-acetyl-D-	NONE	Q9UJ70
0	Tryptophar	NONE	P23381
0	Cobalt-con	NONE	Q7SID2
0	Glutathion	NONE	P41921
0	Thiol-speci	NONE	Q9HFE4
0	DNA-direct	NONE	P46669
0	DNA polym	NONE	P16790
0	Hemoglobi	OXYGEN TF	HBB_HUMAN
0	Alpha-amyl	NONE	P00693
0	Ras GTPase	NONE	Q9QUH6
0	Sentrin-spe	HYDROLAS	SENP7_HUMAN
0	Tight juncti	NONE	Q9Z0U1
0	Thiosulfate	NONE	P00586
0	Myoglobin	NONE	P02186
0	Chromosor	NONE	O66659
0	Hexokinase	TRANSFERA	HXK2_HUMAN
0	Carminomy	NONE	Q06528
0	Suppressor	NONE	P25604
0	Crossover j	NONE	Q7SXA9
0	Calcium-bir	NONE	Q06389

0	Elongation	NONE	P29547
0	Isocitrate d	NONE	P28834
0	Endoglucar	NONE	P43316
0	Acyl-CoA-b	NONE	Q8N6N7
0	Methylmal	NONE	P11653
0	2-keto-3-de	NONE	P76469
0	Uncharacte	NONE	Q9LUJ3
0	Biodegrada	NONE	P28629
0	Carbon mo	NONE	P19921
0	UPF0342 p	NONE	Q5L2A5
0	Cytochrom	NONE	P00149
0	Exodeoxyri	NONE	P07648
0	Ig gamma-2	NONE	P01864
0	HTH-type t	NONE	P06533
0	L-lactate de	NONE	Q27797
0	Suppressor	NONE	P52286
0	Methionine	NONE	Q9UBK8
0	Uncharacte	NONE	Q57646
0	Compleme	NONE	P07357
0	Nickel-resp	NONE	P0A6Z6
0	Non-specifi	NONE	P24296
0	Peroxiredo	NONE	P30041
0	CDP-parato	NONE	P14169
0	Myelin P2 p	NONE	P0C6G6
0	Strictosidin	NONE	P68175
0	D-lactate d	OXIDOREDU	DLD_ECOLI
0	Protein par	NONE	P22995
0	Right origir	NONE	P0ACI0
0	Serine-aspa	NONE	Q9KI13
0	Glutathione	NONE	Q9ZVQ3
0	Vascular er	COMPLEX (	VEGFA_HUMAN
0	Proline imi	NONE	P96084
0	Tyrosine-pr	NONE	Q12923
0	Lysosomal	NONE	Q29451
0	Transcriptio	NONE	P35710
0	N-acylneur	NONE	P0A0Z8
0	Bile salt sul	NONE	Q06520
0	Hypothetic	NONE	O31511
0	Electron tra	NONE	P55931
0	Fatty acid-b	NONE	Q02970
0	Medium-ch	OXIDOREDU	ACADM_HUMAN
0	Quinoprote	NONE	P13650
0	Guanine nu	NONE	P52735
0	Chorismate	NONE	Q9PM41
0	Receptor-ty	NONE	P10586
0	Putative rib	NONE	P96579
0	Copper-cor	NONE	P38501

0	Phosphoglu	NONE	P47244
0	Adenosylho	NONE	P50250
0	Putative m	NONE	Q86W50
0	Transposas	NONE	P07636
0	UPF0066 p	NONE	O29998
0	Helix-desta	NONE	P03696
0	Penicillin-ir	NONE	P0C0T5
0	Ribonucleo	NONE	Q08698
0	PemI-like p	NONE	P0AE73
0	Oligoribonu	NONE	P45340
0	Erythronat	NONE	Q9I3W9
0	Androgen r	TRANSCRIP	ANDR_HUMAN
0	Tail-associa	NONE	P16009
0	NEDD8 ulti	NONE	P54729
0	Signal reco	NONE	P14576
0	Protein tra	NONE	P15303
0	Vitamin D3	NONE	P13053
0	ATP-depen	NONE	P09980
0	Gamma-glu	NONE	P54885
0	Erythronoli	NONE	Q03132
0	SPRY doma	NONE	O88838
0	Phosphoad	NONE	O05927

FLI1
HNRNPR
usg
ANXB12
dcp
SEC23A
Gcm1
BLVRA
cmpA
UQCRC1
MAGEA4
HN
F13A1
cmaA2
frdA
pcyA
CDY2A
CDY2B
oprM
argB
MORF4L1
upp
cyaB2
ptxA
dmpG
thiC
VPS4B
PDCD11
ACTA1
VTC2
pac
sucA
CDH-1
CDH-2
CSDE1
STRBP
dacB
yqgF
UAP1
ureA
rbgA
FBP1
MAPK9
ndkA
malQ
cdtA
IGKC

tfe
PA3967
rnp4
nrfG
FOXM1
RBP7
IMPDH
DSL1
hha
S100A6
PRKCQ
SERPING1
Fnta
hisE
CHI1
Smtnl1
CTH
phyA
cyt-18
sqv-4
ala
mnmA
Segment-7
AZGP1
Cgl2
ALK
mukF
FET3
MKI67
glyA
act2
nanA
glyQ
dcd
NMRK1
LOX1.4
fmn1
otr
puhA
CHID1
pelA
dsvA
DPYS
nudC
sodC
HA
dnaX

sspC
Crygs
xthA
ykoF
hemB
Y10A
mltB
Dag1
yxaF
GOT1
glmS
TOP2A
PRKD2
sodA
aceA
scoA
ghbA1
ccp
USH1C
FAS2
psbA
HSD17B4
GPM1
VWF
GTF2I
ANXA3
tyrS
rhmD
nspA
ybaA
hydB
CYP2E1
MT0975
Rv0948c
Ppp1r8
xynD
cysJ
menB
KPNB1
los1
hba
femA
PAP2
srfAD
hlyB
rbp3
petB

ARHGAP5
EPN1
cycA
Adh
Mog
SATB2
cpcA
SEM1
snoaL
glpK
MYO6
pyrDA
atpA
hbp
NDC80
eutD
tnpA
HMOX1
rpl1
HBEGF
IQUB
mgsA
fliI
ESR2
CPSF3
STARD5
DMC1
PATJ
GAL80
YARS1
CD1A
era
trfB
Gpi
Ll637
int
BCAT1
proS
yjcS
GST2
hxlB
pepA
catM
LYZ
49
NAGK
WARS1

GLR1
fmo1
RPA43
UL44
HBB
AMY1.1
Syngap1
SENP7
Tjp2
TST
MB
dnaA
dnrK
STP22
mus81
FRQ1
CAM1
IDH1
ACBD7
mutB
rhmA
RDM1
adiA
coxS
GK0640
recC
sinR
SKP1
MTRR
cgi121
C8A
nikR
PRDX6
rfbE
PMP2
STR1
dld
parD
rob
sdrG
GSTZ1
pip
PTPN13
MAN2B1
Sox5
neuA
SULT2A1

yesE
ETFDH
FABP1
ACADM
gdhB
VAV2
aroC
PTPRF
ydaF
nirK
pp63-1
PFE1050w
METTL16
A
AF_0241
2.5
mepA
nrdE
mazE
orn
pdxB
AR
5
Nub1
Srp54
SEC23
Vdr
rep
PRO2
eryA
Spsb2
cysH

Tumor nec	DrugBank
Interleukin	DrugBank
Monocyte	N/A
Lipopolysaccharide-binding protein	

UGT1A9
UGT1A1
SLCO3A1
SLCO2B1
SLCO1B3
SLCO1B1
SLC7A8
SLC7A5
SLC6A2
SLC47A2
SLC47A1
SLC22A1
SLC18A2
SLC16A10
SLC15A1
POU2F2
PAH
ORM2
ORM1
MAOB
MAOA
HTR7
HTR6
HTR3A
HTR2C
HTR2B
HTR2A
HTR1E
HTR1D
HTR1B
HTR1A
HRH1
DRD5
DRD4
DRD3
DRD2
DRD1
DDC
CYP3A7
CYP3A5
CYP3A4
CYP3A
CYP2D6
CYP2C9
CYP2C8
CYP2C19
CYP2C18

CYP2B6
CYP2A6
CYP1A2
CYP1A1
COMT
CHRM5
CHRM4
CHRM3
CHRM2
CHRM1
BCL2
ALB
ADRB3
ADRB2
ADRB1
ADRA2C
ADRA2B
ADRA2A
ADRA1D
ADRA1B
ADRA1A
ADORA1
ABCG2
ABCB1
SULT1E1
SULT1B1
SULT1A3
SULT1A2
SULT1A1
SLCO1B3
SLCO1B1
SLC22A6
SLC22A2
M
HTR2C
HTR2B
HTR2A
HTR1B
HTR1A
GRIN3A
DRD5
DRD4
DRD2
DRD1
DDC
CYP3A5
CYP3A4

CYP2C8
CYP2B6
COMT
CHRNA7
CHRNA4
CHRNA3
ALB
ADRA2C
ADRA2A
ADORA2A
ABCG2

LRRK2
SNCA
PRKN
PARK7
PINK1
SYNJ1
MAPT
GBA
UCHL1
SLC6A3
SNCAIP
PINK1-AS
VPS35
DNAJC6
HTRA2
VPS13C
LOC106627981
PLA2G6
DNAJC13
GIGYF2
ATP13A2
FBXO7
PARK16
PARK10
PRKAG2
NR4A2
PACRG
PARK21
PARK12
ATXN2
TH
TAF1
EIF4G1
MT-ND1
TBP
PODXL
MT-TK
GCH1
ADH1C
PARK3
ATXN8OS
GLUD2
MIR433
MIR133B
CHCHD2
ATP1A3
RAB39B
POLG
SNCB
PSEN1

TRPM7
SLC18A2
GRN
GDF6
APOE
DCTN1
MAOB
TBC1D24
ATP6AP2
COMT
CHMP2B
MT-ND5
NODAL
TBX20
VCP
COQ2
SQSTM1
C9orf72
TREM2
PRKRA
TARDBP
APP
DRD2
SOD1
MT-TT
NDUFS4
KMT2B
TNNI3
BDNF
PANK2
SLC39A14
UQCRC1
ATXN3
C19orf12
SLC30A10
FGF20
MT-ND6
CYP2D6
TMEM230
RPS27A
NDUFV2
PSAP
PRNP
FUS
GDNF
WDR45
GSK3B
HFE
FMR1
CP

CHAT
DDC
DRD4
STUB1
GPR37
SNCG
MAOA
MIR659
MT-TP
GAK
LOC108663996
DRD3
CHCHD10
PDGFRB
CAT
SYT11
ADORA2A
DDOST
TWNK
COASY
CASP3
LOC108663987
SERPINA3
PSEN2
LAMP2
PON1
NFE2L2
BMP2
UBE2L3
CDK5
SLC6A4
CACNA1A
CHROMR
TOR1A
SORL1
RAB29
CYCS
FTL
DRD1
KLK6
HTT
HCRT
PITX3
ACHE
MT-ND2
DNAAF3
HTR2A
PDGFB
TFAM
LMX1B

LINGO1
SOD2
STH
SPR
NAT2
SPG11
MT-CYB
BST1
GAD1
BCHE
MT-ATP6
AUP1
RNF19A
LRRK1
MYH7
TMEM175
GFAP
DBH
HLA-DRA
NEFL
MT-ND3
TBK1
CASP9
KIF5A
MAP2
VPS26A
MAPK14
JAM2
NOS1
DNM1L
XPR1
FRAXA
PPP2R2B
SIAH1
NQO1
BAX
MAPK8
AKT1
CCDC62
STX1B
MBP
MIR132
RIT2
NRTN
PARL
TK2
EPHA3
MYORG
HSPA4
HSP90AA1

ACO1
TNK2
HMOX1
UBB
HSPA8
NGF
CTSD
HSPA9
PARP1
AIMP2
ALB
BECN1
CSF1R
MCCC1
GAPDH
GSR
ACTC1
TTR
SLC41A1
GSTO1
LMX1A
MT-ND4
DGKQ
MGC32805
CALB1
GFRA1
MAPT-AS1
RAB3A
TMEM106B
NEDD4
SNX27
STK39
VDAC1
HTR1A
PRKAG2-AS1
DDIT4
NTRK2
GSTO2
PVALB
GRIN2B
MIR6084
MT-CO1
PTEN
CLN3
ATXN1
CNR1
PTRHD1
UBE2E3
PDYN
NEK1

MAPK10
TAF1L
IREB2
FIG4
HLA-DRB5
NQO2
SLC2A1
CDCP2
FAM47E
RNF11
PSNP2
GPNMB
HIP1R
ACMSD
INPP5F
NNMT
OPTN
LOC109504728
TUBA1B
TSPO
BAP1
GATD1
RAB10
MSN
SLC11A2
NEAT1
MFN2
GJD2-DT
SLC20A2
SFPQ
TUBA4A
PAH
USP24
GRM5
TRAP1
HNRNPA1
NUDT1
SH3GL2
RAB8A
CASP8
PRKCD
TPH1
TNNT2
ATP13A3
GRIN2A
FBXO48
DYRK1A
NTS
PPARGC1A
RAB7A

MIR34C
PM20D1
STBD1
CCL5
SEPTIN4
RAB5A
UBQLN2
AFG3L2
PRODH
WASHC5
AAK1
RHOT1
GAP43
CHM
MFN1
SLC6A2
ATXN7
JUN
UBE2D2
USP40
CNTF
MT-CO2
MAPK3
VPS29
DAPK1
NTF3
UBA1
SIPA1L2
ALDH1A1
CDC42
UBE2N
ADORA1
PSNP3
ARTN
SCN1A
TOMM20
UBE2E2
OGDH
OTX2
TOMM40
MIR212
FGF8
NEFH
MALAT1
CUL1
DYNC1H1
HRH3
MIR24-1
MIR29B1
NDUFV1

MNDA
TSC2
EN1
ATXN10
BACE1
EIF4EBP1
H2AC18
LAMP1
ATXN8
QDPR
FAF1
ELAVL4
MARK2
MTFMT
TGM2
POU5F1
FA2H
ABCA7
SOX2
DNAJC12
NDUFA1
SACM1L
MIR26A2
MT-TL1
RIC3
LINC01262
MIR124-1
MAF
HOTAIR
SCN5A
DNAJB6
MYBPC3
GRIA2
ADCYAP1
SNHG1
GRIA1
MSX1
FOXO1
GRM1
PCSK1N
NDUFS7
NFKBIA
EN2
PSPN
BAG2
PENK
NDUFS2
NEDD8
NTF4
ACTB

A2M
RAB12
SORT1
MYH6
IGF2R
PGAM5
TNNT1
TPPP3
PFN1
TAC1
KLF4
VPS13A
RAB5B
MAP3K13
CLU
MYC
MATR3
SLC25A4
MCU
WASHC1
RAB11A
PPP1R1B
CCNF
UNC13A
ATG12
SEPTIN5
GRIK2
TBC1D15
AIF1
MT-CO3
SNX3
EIF4E
JUP
LIN28A
GLIS1
H19
ARIH1
NDUFB3
MIR22
NDUFS8
MPO
PCA3
DPY19L2P2
RNU1-1
SCOC-AS1
MIR4422HG
LINC01734
SGCE
MSRA
ORC6

KCNQ1
ACTG1
NDUFS3
IAPP
RET
NDUFAF2
EEF1A1
PIK3C3
DLG4
P4HB
MT-ATP8
NDUFB9
IFNG
ABCC9
PDE10A
NDUFB11
UBE3C
HPCA
MIR9-1
RING1
SFXN5
UBE2S
ANG
FIS1
CAMKK2
NOS3
RAB7B
PON2
CASQ2
NOTCH2NLC
CACNA1D
SERPINI1
SNX1
ARIH2
NCSTN
UBE2A
MT-TQ
SORCS1
TTBK2
AMBRA1
HSPBP1
TTN
ANKRD50
SORCS3
MFHAS1
ENTR1
PCDH19
THAP1
PSENEN
HM13

VAPB
TTN-AS1
AP5Z1
KCNC3
APH1A
APLP2
EEA1
NDUFS1
SNX6
ZSCAN21
WASHC4
TJP1
SNX2
ERBB4
LOC107032825
SNX5
VPS26B
TBC1D17
POTEF
MIR21
UCHL1-DT
HARS1
UNC5C
SKP1
CRH
DENR
ASPA
RNF144A
RANBP17
GMEB1
FUNDC1
SYT12
EFCAB6
TVP23A
RAB40AL
MIR331
UCH1LAS
PHB
MTHFR
COL5A1
MT-TN
IGLON5
LOC102724058
GSTP1
SST
MECP2
TNF
NDUFS6
NDUFA6
GSTM1

PDE4A
NDUFAF3
STXBP1
KEAP1
NDUFA11
NDUFA13
SURF1
HPRT1
NDUFAF1
TMEM126B
CBS
JPH3
SNCA-AS1
SLC26A4
SLC26A4-AS1
MHRT
IL1A
MIR29A
PRKAA1
LOXL3
NPPB
SELENOT
S100B
VIPR1
MIR34A
PRKAA2
DNAJC5
MT-TI
INPP5K
GLE1
EPHA4
EIF2AK2
CYLD
IL1B
TMEM240
LDHB
ALS2
ATN1
MT-TF
HFE-AS1
TIMMDC1
APOA1
UBC
DSC3
MT-TS1
MT-TH
MT-TS2
PDE5A
IMMT
TCAP

NR1H4
IL10
REST
JAG1
PON3
MIR146A
ABCB1
PTPRC
SCARB2
ATP5PF
NKX2-5
NDUFB10
PRDX1
SIGMAR1
RRM2B
CFAP410
PSMF1
PRKAG3
PANK1
PNKD
PRPH
NDUFAF4
FOXRED1
NDUFAF5
NDUFB8
ERCC8
MAP1LC3B
DCAF17
PLAU
SPG7
GAA
NUBPL
NDUFAF8
FBXO42
LDHA
NDUFA10
YWHAQ
CRYAB
OPA1
SCN2A
SREK1
POLG2
COX4I1
NPC1
CDH1
NOTCH3
IGF1
PIN1
TF
DIO2

NPY
SCYL1
MIR107
UBQLN1
G6PD
IL6
PICALM
TEX15
FATE1
CHL1
NDUFA2
SYP
SDHAF2
CYP27A1
JMJD8
RD3
SCN9A
RYR2
BCL2
THAP3
GCLM
NOS2
TYROBP
ZFYVE26
DAO
PDE8B
TAF15
SI
PSMC4
NDUFA12
NDUFA9
PIP4K2C
UBA2
SETX
ANXA11
MRE11
TSC1
MIR106B
MIR328
MIR298
AD5
AD16
AD17
AD6
AD10
AD11
AD12
AD13
AD14
AD7

AD8
TP53
MMP9
DHX9
HNRNPA2B1
NANOG
ERCC6
NPEPPS
CTNNB1
UBQLN4
GNAL
TPPP
ETM2
APAF1
GLT8D1
RNASE4
ALS7
ALS3
LOC108903148
AARS2
MGAM
PTS
RANGAP1
PANK4
CACNA1C
AMIGO2
GSTT1
HSPA5
CDK1
GJC3
SNORD56B
PAWR
FBXL18
CHGB
DSC2
TAF13
ITGB5
PI4K2A
GJB2
ATG7
APOL1
EPRS1
SLC25A41
INPP5E
PI4K2B
MTMR7
CAMK2G
ACE
SERPINA1
SNAI3

ZBTB1
DESI1
UQCC3
CYP1A1
GOLPH3
HP
CNBP
MC4R
SPINK1
VDR
TNR
LRP10
NSF
BCS1L
NDUFAF6
MOBP
TFRC
PRKCG
SNAP25
TSEN54
VCL
ANO3
PCBP2
GNE
SUMO1
SYN1
MAPK1
SIRT1
KDM4C
SEMA5A
CRP
APOC1
ZNF23
ATCAY
RBMX
NDUFC2
CHRNA4
SCN1B
SMPD1
UBE2G2
MRPS14
CIZ1
CRHR1
ETM3
MIR17
MIR20A
SDHA
MIR19A
MTOR
ZNF746

CASP1
TUBB
PCDH10
UBTF
GNPDA2
NOP53
DPP6
PCBD1
TTBK1
COX8A
CHI3L1
ATF6
LMNA
ATP13A1
MIR18A
DCC
PQBP1
SCG2
DRD5
U2AF2
CCK
MEF2C
INA
MT-TL2
TOR1B
GYPE
EIF2S1
PSMC1
SRSF7
GRIA3
SLC1A2
ESR1
FTH1
HNMT
MT-RNR1
SEZ6
SP1
ANK2
WNT2
COX6B1
GLUD1
STMN2
VEGFA
TUBB2A
CYP2E1
MTIF3
RHBDF1
TIA1
TNK1
BIN1

NRGN
KANSL1
FYN
MEF2D
CAST
GTPBP3
MAPK9
PSMD4
FAS
CASP6
BCL2L1
CASP7
NFKB1
GPRIN3
CPT2
BICD2
NGFR
CPLX1
SDHD
PMPCA
COX10
MT-TE
PTGS2
SMC1A
CRAT
SRSF9
RAB38
MT-TV
MT-TW
DMPK
CYP1A2
TLR2
RBM8A
HLA-DRB1
ERLIN1
SCO1
COA3
CDKL5
MTR
COQ4
PRKAR1B
REPS1
PRL
TDP2
VPS41
GLUL
CTSB
SLC1A3
NUCKS1
PLCG2

LYST
USH2A
INS
GRIN1
RELN
TXN
NEFM
H1-2
PRDM16
RIF1
HIF1A
VAMP2
ATM
KCNJ10
MT-TA
ATXN7L3-AS1
PTBP1
LOC110806262
CST3
ATP5F1C
BAG3
SRSF6
UQCRB
CALM1
PFAS
ELP1
UBE2L6
ADRB2
PPIF
TNFRSF1A
HRH2
FXN
TGM6
XBP1
TMCO6
NDUFV2-AS1
CHIT1
CELF4
MIR324
CRYAA
PABPN1
PSMC6
TLR4
GAD2
MC1R
GABRA1
GABRG2
ESR2
GRK5
KCNH2

IL2
UBE2J2
DDIT3
SLC25A6
FGF2
WNT3
CAPN2
PNMT
ADNP
ERBB3
TUBB4A
NDUFC2-KCTD14
ATF4
IFT74
MFSD8
VDAC2
UBE2J1
ADCY5
CDK5R1
SYNE1
AQP4
TUBG1
CACNA1G
IL4
SLC18A3
DLG2
HSPB1
TFEB
AKR1B1
NPTX2
SRSF4
HTR2C
KCNN3
MAP3K5
MS4A4E
EPHX1
SREBF1
KCNN2
FASTKD2
CCNE2
CHMP4B
NES
ARID1B
PHF1
TRA2B
LAMP3
CFL1
MBNL2
DLST
DCTN2

SSBP1
MIR34B
TYR
CCNE1
NOTCH2
RAD51
FLNC
MASP2
POU1F1
TM2D3
CLIP1
LMOD1
VDAC3
MAPK12
BAD
UQCRC2
CXCL12
RPL10AP7
HDAC6
HCFC1
TMEM163
PAICS
PARK7P1
PARK7P2
TACO1
SLC40A1
APC
MAPK11
ITPR1
EIF2AK3
SCN3A
ALDH2
ULK1
GSTM3
CYC1
MAPRE3
SLC2A13
LINGO2
CALB2
ATPAF2
MIR30B
RGS2
MAPK13
NOD2
CAMK2A
POLR2A
CHRNA7
ATP13A4
HLA-DQB1
PSMB5

ACTR3C
FHIP1B
PRRT2
ATP5F1B
STX6
HSPB2
RALGDS
MIRLET7I
SLC33A1
TUBA1A
HSD17B10
DLG1
GSS
LRRC37A
PSMA1
MYH11
GRIN2C
PSMA6
MIR92A1
MIR4697
VSNL1
CCKAR
NEBL
HS1BP3
NDUFB6
TBX3
TPP1
PLEKHG5
LOC109029536
SLC25A5
HSP90B1
PSMB1
PSMB6
MIR19B1
APOD
CAPN1
CYP1B1
ATP5F1A
BMP4
CCL2
MB
MT-ND4L
TGFBR2
TFR2
DKK1
MRC1
PSMB3
NDE1
CHGA
CDC37

MT-TG
YWHAB
RAD50
HLA-DQA1
GRK2
PIK3CA
CTNNA3
LGALS4
PMM2
LRRC37A3
CALCA
PTPA
COQ8A
MIR26B
MIR128-1
MIR363
NEUROG2
UQCRFS1
MAP1B
SLC9A7
CHST7
KRBOX4
SNORD52
PSMA7
FADD
SLC17A6
IL1RN
DICER1
SOX9
ASCL1
MIRLET7G
MMRN1
UBE2G1
ADAM10
BCKDK
LRP6
OGG1
RPS6
SATB2
KIF1C
PEX6
CLN8
SEMA6B
CEP104
INCA1
C11orf65
TH2LCRR
TBX3-AS1
LOC101927078
SCA18

DYT17
SCA25
SCA30
FLOT1
HMGCR
KLC1
ADAM17
MIR26A1
MIR195
CCKBR
PSMC2
CDR1
PSMA4
IDE
HDAC4
SIRT2
LAMA4
FNIP1
GSTA4
DNM1
PGK1
PSMC5
GART
GAL
PRKACA
SIRT3
ERBB2
HRAS
MT-RNR2
COX5A
ATIC
DVL2
FAM47E-STBD1
BMP5
APEX1
RAB3GAP1
NARS1
DSP
HSPA1A
IGF2
NOP56
SCN11A
PER1
USP25
LRPPRC
TRIM9
ENO1
MBNL1
PSMB2
MAP2K6

YWHAZ
SMN1
PSMD3
MAP2K4
HNRNPH2
RAB25
DUSP11
PTCH1
BRCA2
SMO
PTCH2
NTN1
CLCN6
SUFU
DNAL4
NEB
MYF5
SDHAF1
CFAP43
MILR1
PET100
COA8
MIR125A
MIR326
MIR199B
MIR30D
LOC100507346
SPG41
SPG19
SPG29
SPG36
SPG37
SPG38
PSMA3
TPM1
ABL1
ARSA
U2AF1
AKAP9
TTC39A
CDKN2A
CA4
PPP3CA
TRPV1
NR1H2
LRP5
H1-1
LMNB1
TUBB3
GNAI3

ADSL
NOX4
CSNK2A1
PSMA2
WNT1
CD200R1
PRDX3
CS
HSPA1B
TUBB4B
MAPK8IP1
FOS
PSMD2
NTRK1
STAP1
ALX4
PJVK
LINC02210
LGALS3
UBR5
HNRNPC
CSNK1D
MAP2K3
HNRNPDL
NAGLU
GSK3A
ATG5
BGLAP
FGF10
HSD3B7
TLR9
RPS12
CYP19A1
CYBB
SPP1
PHOX2B
HTR3A
DDRGK1
DSG3
AP1S1
SYNCRIP
ADH1A
IL17A
TUBB6
MANF
VIP
UBE2V1
AQP1
PSMD1
SDHB

OCA2
SV2C
SLC45A3
EP400P1
SRSF2
GPX1
KIF11
BLVRB
NRG1
PDXK
YWHAH
EGF
TPI1
ACTN2
DGUOK
CSRP3
ERN1
CREB1
ATG14
SET
PSMB4
PSMA5
DPYSL2
NUB1
CACNA1H
MDH2
NCL
GJA5
TBX5
RAI1
PRRG4
QSER1
PITX2
TUBA3D
MAP4K4
CSNK1A1
LAMC1
NONO
AXIN1
MDM2
BYSL
SPAST
SCN8A
MX1
SLC1A1
USP30
PANK3
PEBP1
ADD2
CXCR4

GDF15
ABCA1
NRP1
SGK1
DISC1
REN
CALML3
GALC
TPT1
YWHAE
AGER
CYP17A1
ITGA8
CNNM2
PLEKHM1
UNC13B
SFXN2
TAS1R2
ZNF646
CNKSR3
ISM1
PRDM15
PRSS53
DNAJB1
SUCLG1
ATP5PD
EGFR
RPS6KB1
WDR45B
GABARAPL2
RPS16
IRS1
PLD1
SLC39A13
SLC30A9
HNRNPA3
HEPH
TFB1M
PSMD6
AHSA1
TMOD2
PPID
CTSH
MIR17HG
AKT3
TUFM
SHH
ADH5
HTR1B
PIEZO1

CYP2C9
BLOC1S1
CNTN1
EPG5
MT3
RAC1
GDI1
GRM4
KIF5C
RPS2
DNMT3B
MDH1
COX7A2
PIK3C2A
FRMD4A
TUBA1C
RYR3
ITPR3
KCNJ6
LEP
INPP5B
FASLG
PRDX5
RPTOR
TPK1
DUSP1
WARS2
KCNIP3
MSTO1
TIAM1
MAP2K1
GABARAPL1
YWHAG
PLCG1
NDUFA5
MAP2K7
RAF1
NCKAP1
RAI14
TIMM13
SEPTIN7
CHRNA3
MIR126
TOMM70
MTHFD1
PRKG1
PPARA
RPS11
MOGS
POMC

NEDD9
PUF60
HOMER1
RICTOR
CYP2B6
IL1R1
KAT2A
SPPL2C
ATP5PO
ADRA2A
MIR103A1
EIF4A2
CYFIP1
LRPAP1
RPS15
FGFR1
BAG5
UBE2D3
XIAP
OTUB1
PRPF6
DNM3
CSF1
ADRM1
UBE2K
GBAP1
PFKM
MECOM
PRSS1
RPL18
VPS16
RPS20
GPX3
ISL1
ANXA5
UCHL3
AIFM1
LOC109461478
RPL7A
UBA52
PSMD8
RBFOX1
NUDT6
FKBP1A
MGAT5
KIF5B
NR2F2
CR1
DKK2
LRP8

NOX1
SLC17A7
DKK4
CSNK1A1L
TET1
UBA7
EPHX2
M6PR
TPH2
MTRR
GSTZ1
TAP2
SYT4
BDNF-AS
PRSS3
PRDX2
CD4
ADAMTSL1
TMOD1
MMP13
IGFBP2
MIR376A1
ENO2
ATG13
UCP2
TYMP
NBN
CDNF
ARFGAP1
PNKP
GLRX
RPL13
MIR148B
MIR3605
NDUFA8
NDUFS5
LIF
GLA
CCR5
OPRK1
CCR2
PSMB9
COQ7
HUWE1
HSP90AB1
RNF144B
RCSD1
LMOD2
NTPCR
MED13

MTX1
TBC1D5
SLC25A28
LGI1
HSPD1
SUCLA2
KCNQ2
CNTNAP2
MGRN1
CRK
RPL26
CCAR2
WNT5A
NCAM1
BLZF1
GGT1
HAX1
C4A
APH1B
SNX13
ADRA1A
POLD1
WDR6
PSMB8
TAP1
GBF1
GFPT2
ESD
SKIV2L
IGF2BP3
RPS27L
NOL6
HPF1
CASP2
PPARG
KLK11
BTNL2
TMEM59
TOX3
SPPL2B
TMEM229B
TGFB2
PIK3CG
FGGY
FGF1
CXCL8
PLK1
SPG21
ATP6V0A2
RBMS3

NUP42
FRA6E
GABARAP
CCR6
NIPA1
IKBKB
CSF3
PAK4
SRRM2
ADH4
SNX14
WIPI1
OTUD5
TECPR2
SPART
GRK6
GH1
HNRNPH1
CTSL
FDFT1
PAX7
MAP3K14
KDM6B
PRICKLE1
DAAM1
ASRGL1
COL13A1
STAB1
ADAMTS16
HLA-DQA2
ARHGAP27
TOR2A
CTC1
LHFPL2
SLC50A1
BRINP1
CLRN3
DBF4B
PAQR6
NEURL4
ZC2HC1C
CCDC82
TMC3
BORCS7
ODAPH
TCIM
CYP17A1-AS1
TMC3-AS1
LOC100996842
LINC00693

NLRP3
FLNA
GPD1L
PKM
ADCY10
CELF1
RPS18
RPL34
IL2RB
SDHC
AKR1A1
SERPINF1
HEXA
RTN4
SLC5A7
PRKACB
CREBBP
MIR106A
MIR92A2
MIR19B2
PSMC3
TCP1
PLK2
KCNJ3
CHP2
LSM14A
CAPN3
KMT2D
MRPS27
TACR1
IRS2
FBXW7
AHR
AURKB
WFS1
CACNA1I
PTN
PLXNC1
SLC2A9
RPL10A
KCNJ2
CACNA1E
TRPM4
SNTA1
WWP1
RBM20
FLNC-AS1
LOC111188154
HSPA1L
COPS5

AOC1
FMNL1
ERLIN2
ATXN2L
VIM
NTRK3
DDX3X
CTRL
MAP1LC3A
PCBP1
ITPR2
EPHB2
RPSA
OPRM1
TRIB3
WASHC2C
PLCB1
WIPI2
TUBGCP2
TUBA3E
RUVBL2
UQCR10
CD40LG
CDH2
UBE2E1
MPV17
VPS54
BCL2L2
SYT10
PET117
FRAXE
RBX1
FGF14
LARS2
RTN2
REEP1
RPS3
RPS27
RPL14
AVP
RUVBL1
DNAJA1
TCIRG1
CYP3A4
HNRNPU
ISG15
TRAF2
SH3GLB1
PABPC3
NR1H3

ITGAX
IDUA
IL15
SESN2
C3
CFH
ARNTL
ACO2
STIP1
MSRB1
FOLH1
MCM5
SLC39A8
MIR30E
MIR10A
MIR370
MIR128-2
MIR136
MIR127
MIR30A
MIR375
MIR431
MIR497
MIR485
MIR1224
MIR16-2
MIR409
MIR503
MIR873
MIR338
MIR18B
MIR20B
MIR497HG
MIR5193
MIR1294
MIR4448
TGFB1
TOMM22
RPL26L1
MMP1
FKBP4
STOML2
HERPUD1
PABPC1
CYP2C19
CNTN2
PRORP
GATA2
EPHB1
RRAS2

XRCC1
TFB2M
PHACTR2
ABCB7
UBE3A
SACS
RNF41
SLC25A13
SLC25A3
FANCI
PAK6
EP300
NHLRC1
AR
RHEB
URI1
ABT1
AFDN
MAG
GLI3
WAS
PPP3R1
FOXG1
IFNB1
PPCDC
ATG2A
RBFOX3
WDR41
RAB27A
SLC25A1
MAPRE2
HNRNPF
HNRNPM
ATP2A2
RHOA
ATAD3A
C1QA
ATP10B
RPL23
EPO
COL4A1
TMPRSS6
TRAF6
COX4I2
LRSAM1
ACTL6A
ITGB2
IL6R
SAMHD1
TBPL1

PPP1R17
COQ9
MCL1
RPL6
HMGB1
PURA
ATL1
PLEKHG4
PLD2
SAG
CDCA7L
CLSTN1
ZFYVE27
APBA1
ANO5
GPT
CAMK2D
TUBG2
CLTC
CCT4
CCT6A
RCAN1
MRRF
SFN
LTF
AP3B1
HK1
CHRNB2
SLC25A24
ADPRH
NPPA
USP35
MMP3
PSMB7
HADH
RPL30
CYP27B1
SCN4A
SCN2B
SCN4B
SCN3B
TNFAIP8L1
EZH2
KNG1
NMT1
MAT1A
DECR1
IPO9
ALDH9A1
FMR1-AS1

SMARCB1
CSNK2A2
GLRX2
PHF5GP
ECHS1
CHRM1
RAB1B
RPL12
TUBB1
ABCG2
TP73
PLN
ESRRG
GUSB
FGF21
H3-3B
CD33
MARS1
SRI
PTRH2
GPD2
IRS4
NOVA1
TRAPPC4
PPP1R8
MRPL11
RBM39
RACK1
FASN
TKT
EEF1D
WBP11
APBB1
PSMD7
PSMD14
HMOX2
CNR2
ZDHHC8
USP36
UBE2D1
IARS2
G3BP1
RAG1
GRID2
ADAMTS4
KCNT1
IGHMBP2
ARHGEF9
SLC25A22
SMCR8

PDIA3
RB1CC1
MSH6
SRM
RNASEH2B
PAX6
CX3CR1
MIR223
RAC2
F2
HRH1
CD14
GBA2
CSTB
XDH
COX5B
GLP1R
ADH1B
ATP1A1
RPL28
CTTN
ALOX5
LYN
USP2
ACAD9
PAK1
PIK3R4
CAPRIN1
LIMK1
SLC8A1
CAPN5
PDYN-AS1
PIKFYVE
RPS13
NUP133
PLG
B2M
NELL1
MYCBP2
PNOC
SKAP2
CLOCK
ARNT
PRICKLE2
PPHLN1
RANBP2
IDH2
SLC25A11
CRYZ
RPN1

SFXN1
SEPTIN2
RELA
MME
PLA2G4A
ITM2B
MPP2
NDUFA4
VAPA
ATP6V1G1
FOXA2
FOXO3
RB1
GLI1
CENPJ
SFRP1
POLRMT
PRDM2
MS4A6A
SRRM4
PML
FBXO40
TOP1
PLA2G7
DVL1
DVL3
MLST8
COX7C
SPTBN2
IFNA1
CAD
PAFAH1B1
COPB2
DDB1
GHRL
PLXNA2
DHX30
PHB2
RPLP2
SRP14
TARS1
NMD3
UTRN
NOTCH1
DNM2
CUL4B
FLOT2
NAE1
RTN3
SF3B6

APEH
PPCS
RPL35
FBL
FARSA
GNL3
CACYBP
MRPS9
CA2
MARK3
ACTA1
IL18
FARP1
TPRG1
MIR135B
ESRRB
DAXX
CCT3
EXOSC3
S100A6
LSM4
RSU1
VTA1
COX6C
BRAF
MYH14
RPL10
WASF1
RPL21
PRPF31
RPS15A
DLG3
PRPF8
DNAJA3
RPS23
SF3B3
CDKN1B
KAT8
VPS11
NEK11
CDR2
GPHA2
NCS1
PREP
STK11
DEPTOR
NR4A1
YME1L1
CAV3
UQCRQ

WNT3A
CHRNA5
MUTYH
GRIK3
GABRG3
SLIT3
APCS
MKKS
RAB3IP
TEF
PSME3
RAB8B
BRCA1
CCNB1
PTGDS
PLD3
NPM1
SRSF1
SRSF3
CIRBP
RPS25
HAMP
SERBP1
H1-0
VLDLR
LPL
ATP7B
PHYH
DGCR8
HAPLN1
OPN4
MARK1
CIB1
REG1A
DDHD2
BHLHB9
GSAP
MIR210
ATP13A5
TRIT1
DPYSL5
IFNA17
MIR6070
BAK1
HLA-DMA
CTNND1
ALAD
CCT8
PCNA
RPS19

EEF1A2
MCM7
AMOT
DDX17
MMP14
SLC11A1
ABCC8
GPX2
VTN
OSM
LIN7A
GEMIN8
MIR144
PRDX6
MRAS
CR2
AOX1
FCER2
EFNA5
FOXO4
MAP3K10
MDK
RPS24
SRGAP3
NTNG1
LY6E
CHP1
FIGN
PDCD2
HIVEP3
RPS4Y1
MIDN
MIR133A2
MIR133A1
LINC01387
P4HA3-AS1
LOC111258525
KCTD11
TNFRSF1B
ETFB
HLA-B
TOM1L2
KCNA1
BSCL2
KCNMA1
CDKN2B-AS1
SAE1
TRIM28
SEC16A
NACA

CALR
C1QBP
ARHGEF7
UBE2R2
HSF1
UBE2M
ATF2
GJD2
TIAL1
LDLR
MIR7-1
ZNF512B
LOC108663985
PRKDC
IKBKG
SATB1
TRPM2
KCNIP4
KIF1A
HNRNPK
HADHB
AIMP1
PDIA4
IARS1
SCAMP3
OGT
RHOT2
MOG
RTN4R
E2F1
KCNN1
NDUFB1
HGS
SUMF1
PCCA
PCCB
IGF2BP1
RPLP0
MYL12A
TRIM44
MRPS23
TCEAL2
EIF2B5
ACLY
CBX3
DAP3
MRPS31
SMN2
HTR6
GOSR2

BCL7C
HOOK1
BCL2L13
LOX
PNMA2
CYP51A1
NDN
NAAA
ELOVL7
HTR7
INSR
ITGB1
DMD
CLPX
CASK
KCNJ5
USP15
CD163
SYN3
LAG3
PPP2CA
CSNK1E
DDX5
RPL23A
RPL7
SNRNP200
RPL4
MRPS28
RPL8
RFC4
HNRNPR
YBX3
MARCKSL1
PTCD3
SHC3
TNFSF12
PSMD11
PDIA2
RHOU
ALDOA
CSNK2B
MTA1
OMA1
CHUK
PADI2
HK2
CDKN3
PSMD5
CYP46A1
AFF2

PDGFRL
TIMM17A
GFI1B
ERAL1
LCOR
ATXN3L
PRKCA
ATG16L1
CNOT7
ARL8B
ADRA2C
TFPI
DNAJB2
AMBP
SYVN1
DNAH8
NDFIP1
MT1F
PMEL
VWA5A
DNMT1
CD40
VPS13D
STK33
ZNF483
C1orf112
TENM4
MLX
CXADR
PIGO
MCTP2
AGAP1
PIGG
FAM3B
TRAPPC2L
PHF7
FBXL5
SYT17
TRIM40
RUSC1
FAM184A
FAM214B
KRTCAP2
ZMAT4
GTF3C6
TCEANC2
POMZP3
ZGRF1
CWF19L2
MARCHF3

CCDC190
SPTLC1
PLA2G1B
APTX
LRP12
SLC25A37
TMEM119
SNORA49
LOC642361
MIR4432
CD36
HADHA
SHMT2
SSB
BAG1
RAB1A
LRRC59
FUBP1
PNPLA6
TMEM70
TUBB7P
SCFD1
MIR137
BNIP3
PRRC2C
PHGDH
SHC1
RAB11B
ASPH
KIFC3
GLOD4
AP1M1
CHORDC1
KLC2
ATP5F1E
GLS
HNF1A
RMDN3
MCM2
APRT
GALK1
EIF5
KCNAB1
PLS3
EIF1AX
TAGLN2
MYBBP1A
RPL36
STK40
DDX54

MRPL19
SUB1
RPAP3
LEPR
TSFM
DBN1
PITRM1
MIR15A
CTSK
CDC34
DFFA
AOC3
GRP
PRSS2
C1orf115
MIR27B
LRP1
MIR574
MIR424
NCAPD2
DNAJC1
PCMT1
GLB1
KLC4
BCL2L11
SMARCA4
ELP3
FGFR2
PFKL
MAPRE1
RPS10
PGM3
TLE3
CAMK1D
LARP7
RPS26
RPS9
HMGB2
MPG
GGA2
OSTF1
TIMM50
RPL3L
RPS21
CHMP3
ANP32B
DDX10
ORC3
MRPS2
STAC

RPL37A
NOC2L
USP39
DRAP1
DIMT1
POLR2K
SNRNP40
NUDT16L1
TAF9B
CHCHD6
GPKOW
OARD1
MOB3A
SEPTIN6
H2AZ2
SPTAN1
TJP2
CHD1
GFM1
NR2C2
CYFIP2
CNOT3
CCS
RPLP1
C11orf58
SCAF4
FGR
CNTFR
SRF
RABAC1
PSME1
ITSN2
TNPO2
BAG6
HYI
ARFGAP3
MITF
PIAS2
TCN2
SLC7A1
HBEGF
SH3GL1
MEST
VRK3
AGFG1
SLC25A27
UXT
POLDIP2
S100G
MIR27A

MIR205
MIR15B
MIR7-3
MIR7-2
SIRT6
SIAH2
FGF11
EEF2
CAV1
PPP2R1A
HSPG2
TMED10
GYS1
MAIP1
OPRD1
PDHA1
AGT
GPHN
LEF1
DHCR24
AGTR2
MRPL44
HAP1
NECTIN2
EMC3
MIR93
MIR320A
BACE1-AS
SULT1A3
ROCK2
TIMP1
POLR3A
PRDX4
COMMD1
PLA2G2A
PARP4
RREB1
SNX10
RGMA
OTOG
TYW1B
TYRL
RPS2P34
CKB
HYOU1
RPN2
EIF3I
STRAP
MYBPC1
CYP2A6

ITIH4
ECE1
UCP3
EPHB4
MAP3K1
PIM1
PDE4D
NUMB
ROR1
SPHK1
CAMK1
HMGCS2
UGT1A9
IFNGR2
MAK
GPAM
PADI4
QKI
RGS10
USF1
IL7
TRH
ZFPM2
DNAJC19
COIL
PTP4A1
MLLT3
CADPS2
CCDC88C
ANGPTL7
COLEC12
MPP3
GORAB
GNLY
PCBD2
TSLP
RGS1
ZFAND5
LETMD1
S100A16
ZWINT
CILK1
PHLDA1
HMG20B
GADD45GIP1
OLFML2A
MRPL27
ODF2L
SUSD3
SPSB2

RUFY3
VASH2
LARP4
NDUFAF7
SPATA4
PDCD7
ZMYM5
ATAT1
RAB39A
THAP7
ZNF574
ZNF684
CCDC60
C1orf105
MOB3C
PRRC2B
PUSL1
DNAH14
MRM3
H2BC3
MTURN
FAM47B
INKA2
TCP10L3
DPY19L2P1
ANKRD36BP1
TBC1D3P2
MAP2K2
OCLN
CLPB
CXCL1
GM2A
SLC6A20
SERPINE2
CNOT8
DEGS1
DNAJC14
TIMM23
SRC
HDAC9
ADK
OLR1
TFCP2
SYK
THRB
PCSK9
MBL2
IRF8
KMO
TIMP2

SREBF2
PTPRT
IL1RL2
FOSL2
ULK2
PTPRR
NEGR1
RNF114
EPS8L3
HIVEP2
FBXO3
PMEPA1
GPR156
FRMPD4
ZNF385B
MACROD2
B3GALT2
TBC1D22A
SGCZ
RILPL1
KBTBD11
UNC13C
IGSF5
SPATA8
EXD2
TMEM128
INS-IGF2
CDC14C
MIR135A1
MIR520D
OR9G9
ZDHHC8P1
DPY19L2P3
CYMP
SNORD50B
DHFRP2
OR7E28P
BANF1P1
EIF3FP3
NASPP1
RPS3P7
MRPS36P2
RPL26P19
EIF4A1P6
NPM1P10
PAICSP3
MAPRE1P1
RPL9P21
RPS3P6
RPL23AP28

RPS23P3
LOC111365141
PDPK1
CCL26
BNIP1
COX6A2
CSMD1
HDAC2
GPX4
TXNRD2
MYO1C
GDI2
TMX1
TIMM44
ST13
ERP29
OCIAD1
RPS6KA1
GAB2
KCNAB2
ANXA7
BPTF
CH25H
TUBA3C
NDUFB4
L3MBTL3
ACTN1
MIF
AK2
NME1
PDHB
MCM3
XRCC5
CANX
RBBP4
RANBP1
PGAM1
HDLBP
PABPC4
NUDC
DNAJA2
CAP1
GSTK1
PPA1
EIF3B
SUMO2
HNRNPAB
AGTR1
FZD5
P2RX7

NR4A3
PDE7A
TGFA
CHRNA6
PTX3
MUL1
SELENOP
MIR221
MIR153-1
DES
ATP5MG
CDKN1A
MRPS22
SCO2
RETREG1
PIK3R1
GNAQ
ADAM12
MARK4
RAN
ACSS2
BLMH
DROSHA
KHDRBS1
MTHFD1L
XPNPEP1
COG2
THOP1
GTPBP4
SERPINH1
VPS53
COPE
OXT
NDUFAB1
NDUFB7
NDUFB5
MT-TC
ACACA
PPIB
RPL11
PC
RPL5
SLC25A12
USP9X
CBR1
ADORA3
CRKL
STK3
BID
ALDH1B1

PPP2R2A
MYL9
ENDOG
EIF5A
CAPZA1
MPST
MAP4
MED1
SUCLG2
CLIC1
CSE1L
DBNL
PA2G4
PPA2
RFC5
MYO1D
MAGT1
ABCF2
ENOPH1
GRPEL1
ILF3
MYO1B
SF1
TBL3
DHX15
CHML
MRPS17
RABIF
UGGT1
MED27
RAB21
PHPT1
NASP
DNAJB12
UFD1
YOD1
BZW1
TMEM33
ATP1B4
NME1-NME2
MTREX
H3C6
TEC
IDH3A
MTHFD2
ACE2
GK
SYT1
TALDO1
GBE1

CUL2
NAGK
KIFAP3
CDC27
PLS1
NUP85
TRIP4
FAU
NSMCE2
SUPT5H
TBCE
TBL1X
ZFAND6
MOB2
CHRM3
ARG1
TNFAIP3
CACNA2D1
SLC19A1
MATK
ADIPOQ
CHRNB4
GRIK5
MAPKAP1
CHRNB3
BNIP3L
CREM
ZBTB17
DPP7
NACC1
CD200
GHRH
NUS1
TCF25
SLC25A46
PLXNA4
SLC17A9
ATRNL1
SLIRP
CFAP20
WDR75
GALNT17
SEPTIN14
TARDBPP3
ATP1A2
FBXW5
AKT1S1
PRR5
TUBA8
RPS6KA3

CYP2C8
ARHGDIA
PRMT1
RPA1
ABCD3
TECR
NSUN2
NUP107
TPR
RPL13A
RPA3
COPA
EIF3A
OLA1
RCC1
PITPNB
SMARCA5
SRP72
EIF3D
EIF3E
NOP58
ILF2
DDX21
DNAJC9
SRP68
AARS1
MRPL14
MRPL2
RRP12
NOC3L
H1-10
CDK4
MRPL12
ANAPC2
ANXA1
FDPS
ENG
KPNA1
ATP1B2
MRPS30
FAHD2A
NISCH
UCHL5
CASR
GRIN2D
SPTLC2
SCN10A
EPHA8
DLL1
FPR2

TIMP3
BLVRA
COL17A1
GNA14
HCRTR2
TNFRSF21
CYP2U1
HCRTR1
RBP4
DNTT
ANK3
GPC1
GPR50
FXR1
GALR2
MADD
APBA2
KLK8
KEL
JAG2
NRXN3
NR2E3
MEOX2
WNT8B
DOCK3
ADARB2
ASAH2
ANO10
ATG4B
APLP1
HOXA2
MAZ
KMT2C
ACSS1
COL25A1
XK
VSX2
BACE2
CD68
CD207
CWF19L1
APBB2
FRAT1
FXR2
GATA5
LHX8
SEMA4G
SPPL2A
VPS13B
GPR3

NRBF2
CAPN8
CLDND1
OSR2
LRRTM3
VMA21
PCDH11X
RALGPS2
ZNF526
CALHM1
EXOC3L2
MAP1LC3C
TM2D1
TSTD1
ANKRD24
AP5B1
AP5S1
C10orf55
JCAD
MIAT
SOX2-OT
MIR181C
HAR1A
SCARNA6
MIR197
SNHG3
BCYRN1
HAR1B
SNORA54
MIR511
LINC01194
LINC01080
LRP1-AS
LINC01772
LINC01616
LOC106694315
AD9
NPTXR
BLK
STAT6
MSH2
FGG
STIM1
RRM1
VCAN
ACAT2
PPP1CB
AP2M1
HERC2
GPC4

S100A10
SHMT1
UROD
AHCYL1
NANS
POLR3B
IGF2BP2
TRIM25
CBR3
ABI1
CNDP2
CDH13
IGFBP1
EFTUD2
PAFAH1B2
RPS17
PTK7
WASF2
CLTA
ENAH
LGMN
PPIL1
MACF1
SUMO3
RPS3A
SEL1L
RPL3
SMARCD1
CNOT1
AP1G1
FMOD
DEK
GNPDA1
GOLGA2
IFT88
RABGGTA
RPL27A
TSN
SNRPA
AATF
AP2A1
CDC5L
RABGGTB
POP1
NUP160
MAGED2
SUMO4
SON
SRPK3
SENP3

CAND1
CPNE3
ILKAP
DDX3Y
EIF3L
KHDRBS3
KPNA5
RBM28
POLDIP3
PIR
NAA15
LYAR
TBCB
SF3A1
TTC37
SRRM1
WDR77
YBX1
DHX37
ACOT8
HTATSF1
FTSJ3
OSBPL8
RPL17
RNF20
RSL24D1
DPH5
ABCF3
CEBPZ
LAS1L
MRPL39
MED22
RBM14
PWP2
SART3
UNC45A
BPNT1
DDX27
INTS7
RBM23
U2SURP
TXNDC17
TRIM3
PPP6R3
WDR3
NOP2
XRN1
ANP32E
CUEDC1
ERMP1

KCTD12
RPL10L
SRCIN1
PDE12
ZBTB8OS
WDR18
CSN1S1
ELOB
MRTO4
UBFD1
H3-4
MRGBP
SFR1
TTC27
BUD23
CMTR2
MCMBP
LRRC47
PCLAF
SEPTIN8
HDGFL2
GCN1
MIR16-1
LEPQTL1
GNAI2
NDUFA3
NDUFA7
TACR3
SERPINE1
GRIA4
WNT7A
LRP2
SLC22A5
BIRC2
IRF3
FGB
GRIK1
HTR4
OCRL
MFGE8
TET2
SLC7A11
CBX5
COL6A3
ACSL5
GRIK4
FOXA1
LBP
SDC3
RSPO2

CCL7
IL16
NTSR1
MGST1
MAGED1
DAD1
DKK3
LGALS3BP
MOV10
SLC8A2
SMG1
CCL3
ATG9A
IKZF2
LRRC32
SLC30A3
FBXO2
IL27
OMP
MRPS34
NPFF
STARD3NL
AMOTL2
CNTNAP4
CPQ
C15orf48
PCNP
TNFAIP8L2
MYDGF
FABP12
MIR185
MIR29C
MIR494
MIR505
IGH
MIR153-2
MIR384
CYCSP42
MARCKS
EPM2A
GCNA
PSMD13
WWOX
DCAF7
BCR
FN1
GAMT
EFNB1
CLEC7A
CFD

CARD9
IL10RB
IL17RA
TNKS
CD209
CCL20
CD82
GPX7
HIPK3
MSMO1
MYO7A
PIGQ
CDH4
EPHA6
FRS2
TRIM21
UTS2R
CAMP
ASCC1
CDH17
ADH6
CCNC
LHX3
LRBA
IL17F
NAIP
RFX2
ALG11
FAR2
HFM1
SYNJ2
AGBL5
CCRL2
AOC2
IL34
GZMM
PCBP4
PEG3
PRPH2
TTL
GLG1
PIGW
ZNF521
MED7
IL17C
PLB1
SUGCT
SPO11
PLEKHB1
CLEC4E

CLEC6A
TTLL1
PPP6R1
RBM45
MYRF
MTHFSD
SYBU
TADA2B
STX10
MED31
RNF112
VEZT
ZNF569
CYTL1
CAMK2N2
P3H3
PALS1
ZNF470
WAPL
DEFB103B
RIPPLY3
RNASE13
SERTAD3
PGA4
DEFB103A
SLF1
DOP1B
TIMM23B
RTL10
MIRLET7D
MIR196A1
MIR142
MIR151A
SNORD95
PRSS3P2
SNORD97
TROAP-AS1
ENSG00000202498
SLC6A1
NDUFV3
COQ6
CALM3
ATP5PB
CREB3L4
DENND4B
CA8
PKP2
PTPRD
C4B
SLC38A1

KTN1
LAMTOR2
ERCC6L2
MX2
PPRC1
LACTB
SQOR
LRRC37A2
MIR190A
ARX
GNAI1
PSMD12
IGF1R
AURKA
ACVR1
TLR3
BUB1B
ACVR2B
BUB1
EXT1
GABBR2
PBX1
ROR2
FZD2
LIMK2
MMP8
PRKCB
GRM7
FZD6
MTNR1B
PLCB4
SPRY2
RORB
EEF2K
CFI
HRH4
GRM2
FBN1
LIFR
REL
CDK10
ENPEP
EPHB3
KCNA3
NFIA
MTNR1A
PIM2
TAC3
SEMA4D
SNRPB

VCAM1
WNT11
WIF1
AKAP13
DGKD
GPC6
HAAO
FZD3
FZD7
FUT8
NPHP1
TRAF3IP2
BRCC3
B3GAT1
IDI1
IL12A
KCNH7
FGF13
GC
P2RY6
NPY5R
KCNA4
TACR2
RSPO1
SLC8A3
PTPRZ1
SFRP4
VANGL1
XRCC3
IGFBP6
HAS2
FOSB
NPAS2
KRIT1
IGFBP4
TEAD4
PTGES
SMOC2
SALL2
TRIM27
SDCCAG8
RYK
PLEKHA1
SFRP2
USH1C
CCBE1
CDK14
CXCL11
KIR3DL1
CELSR1

CELSR2
FRZB
LGALS8
RAB11FIP2
SLC6A15
SFXN4
NFIC
ZNF462
DHRS2
AK8
CD99
HES6
MPZL1
MYOG
TGIF2
UXS1
ARNTL2
ATG10
AOAH
CD93
CELSR3
CXCL9
GLCE
GNPTG
NVL
MICA
RPS28
SCARA5
RCVRN
STX8
SFRP5
PRAME
ACSM3
ALKBH3
BANK1
ARHGEF19
APOBEC3C
IRX2
KRT6C
DBP
GEMIN2
HCAR2
HMG20A
GBP2
KLRG1
NCEH1
PMF1
MAL
LRRFIP1
RASD2

PKDCC
PGLYRP2
PGLYRP4
MAFK
TECRL
SIPA1L1
SLC25A26
UCN
TRAPPC12
SEC16B
RNF43
RSPO3
STARD4
TGOLN2
ZNF212
TMPRSS5
ATF5
CPEB4
FAM120A
KRT23
GLIPR2
FAM107A
NSL1
PAK5
PCDH18
MTUS1
PXMP2
MBD5
S100A13
THTPA
TMEM237
SRP9
RSPO4
REEP3
YPEL5
ZNF536
AKAP8L
CKAP2
CLIP3
STYX
TAS2R38
SLC35B3
RPL36A
TTC39B
SRSF10
RFX4
TFAP2D
REEP4
ZNRF3
DHX40

DNAJC30
APOBEC4
CD24
KIF12
ENKUR
PDZRN4
MRPL21
LRRC8E
PNRC2
PIGY
TC2N
TBKBP1
SLAIN1
SPDYA
RGS22
NKD2
ZBTB44
ZFYVE21
YTHDF3
XIRP2
AKIRIN2
CEP112
FKBP15
KIAA1586
FYTTD1
LRRC20
OR2J3
TMEM120A
SDR39U1
SNX33
RPP25L
PPP1R21
ZNF282
CASD1
C17orf80
GET4
MIEF1
OBP2A
RPS19BP1
RPS4Y2
RWDD4
ZNF83
AKAP14
ASPHD1
JADE2
MIS18BP1
RBM33
TMEM139
SS18L2
RNF121

SLC7A6OS
ZKSCAN8
ZNF688
WDR24
CCDC43
CSTL1
C11orf16
C2orf88
KCTD14
RERGL
TEX29
ZMAT5
CYB5RL
H1-3
SAGE1
CCDC186
CLHC1
DBNDD2
C22orf31
CCNQ
ZNF783
ATP6AP1L
CFAP126
OXLD1
C8orf86
CFAP300
ERICH2
RBIS
EXOC3-AS1
TSBP1
TENT5D
SLX9
XIST
PGAP4
MCRIP2
RIOX1
MIR141
MIR150
NKAPD1
MIR196A2
MIR214
MIR100
DGCR5
MIR146B
TP53TG3C
IGKV1-5
KIRREL3-AS3
MIR155
OR14K1
GMCL2

MIR193A
MIR29B2
MIR24-2
MIR425
MIR486-1
LINC-PINT
MIR302B
TXLNGY
DUX3
GGTA1
MIR1-1HG-AS1
LOC728392
LOC100287944
BAALC-AS1
HSP90AA4P
HAGLROS
MIR599
VTRNA2-1
LDLRAD4-AS1
MIR626
GUSBP3
LINC01483
TEKT4P2
CFAP97D2
MIR548AT
RPL23AP5
CYCSP38
SORD2P
RNR2
LOC100506725
LOC643327
RPS12P4
LOC101927967
RPS3AP46
RPL34P11
RPS12P5
LOC102723370
LOC109461479
LOC107832851
LOC109609705
NDUFB2
TRAK1
SRRT
FABP3
SMAD4
RYR1
MSI1
CALM2
PSMD9
UQCRH

PTPN22
CIITA
SLC22A4
CD244
NFKBIL1
SERPINC1
SLC17A8
PNPO
IGBP1
ADI1
ATOX1
NAV1
NOL11
CDKN2B
PTH
SYMPK
CDK9
ACBD3
DHX8
EIF3C
POLR3D
UBAP2L
ATP7A
HTR1D
NPC2
PDSS2
TTPA
DPH6
PRKCZ
PDGFRA
CHEK1
VAC14
CHKA
SF3B2
MACROH2A1
ACADS
EIF2B4
EIF2B3
COQ3
COQ5
MED13L
H2AC7
MUC1
EHMT2
BRD2
HLA-C
EIF4A1
REV3L
PMVK
STK10

CADM1
TGFB1I1
ABCB9
SETD1A
WRAP53
UBA6
SEC23IP
RIMS1
AACS
CCNT2
RBBP5
PSMC3IP
CHD9
HEXIM1
NUP54
LSM7
MAEA
PYGO2
PLSCR3
DCUN1D1
CAB39L
MED12L
SMU1
DXO
RPAP1
VPS37B
EIF2D
MACROH2A2
CYRIB
VPS26C
KHDC4
HES1
PLP1
PYGM
KCNQ3
KCNA2
TXN2
COX7A2L
NDUFC1
ATP5MC2
GNB1
LARS1
RBPJ
DAB1
HBG2
PDE6H
MSMB
STX11
HEYL
TBX22

PRND
CDC42SE1
MT-TM
MT-TR
CDK2
RAB9A
TBCA
SLC44A1
SSTR2
RAD51C
SPINT2
POLI
OPA3
POGK
ACTL7A
ACTL7B
NNAT
MTRFR
PRKD2
FARS2
GAS1
SLC25A10
GABRR1
RTN4IP1
GABRR2
TGM4
KCNS2
GABRE
POU6F2
NEXN
BRAT1
GABRQ
FHL5
RNF220
AKAP4
MKX
RRP8
DACT3
ANKRD12
DALRD3
GPR151
MRPL36
ZNF528
DNHD1
STK32B
LINGO4
CCDC183
TOMM20L
CCDC183-AS1
LOC112552175

LOC112081413
NFKB2
ITGA2B
CACNA1B
CD19
MYLK2
BRIP1
TYRO3
ADCY7
COL3A1
FCGR2A
EXTL3
TRPS1
BAAT
ALAS1
CHRNB1
IL1R2
CD38
ITPKB
SEMA4A
HLA-DPB1
NEU1
RIPK4
SLCO1B3
EFNA1
HSD17B1
FGD4
FPGS
PAM
NR1D2
MLH3
MTHFS
SULT1A2
CBFA2T3
AGPAT1
ADAM15
CLCN3
CNTNAP1
COL5A2
B3GALNT1
HLA-DMB
GALNT14
FGFRL1
ITPKA
DSTYK
PARD3
LTK
SULT1C2
THBS3
RERE

VAMP4
WNT9B
WNT9A
ZP3
DPM3
ABCD2
CLK2
ASH1L
AS3MT
KLHL7
KCNS3
HLA-DOA
HLA-DOB
HLA-DPA1
GUCY1A2
KCNIP2
ITIH3
NLGN2
MICB
SLC44A4
SLC26A9
PTPRE
CAPN13
CDC42BPA
DACT1
KCNK10
ITIH1
DTX2
NPFFR1
SLC2A8
SORBS3
STK19
SRCAP
CRLS1
BICD1
ATP8B2
GPR65
IP6K2
PARP9
NEIL2
LDB1
PSD
MCM9
SLC26A1
ST6GALNAC6
NGEF
ZDHHC2
ZNF79
A1BG
CLEC11A

CERS6
HLA-DQB2
IGSF11
PDLIM2
SUPV3L1
ST6GALNAC4
DNAH1
DNAH17
AQP10
ANO2
CNTROB
GPR19
ELK4
GPS2
IPCEF1
KCTD13
FAM126A
NCKIPSD
MEX3C
MDGA2
NFX1
THSD4
STARD5
RUSC2
ZNF184
ZNF668
BIN3
ADAMTS20
DEPDC1B
SAMD4A
ZNF608
ATXN7L3
CENPC
KIF24
GON4L
GPATCH8
FCRLB
GXYLT1
OLFML2B
PCGF3
MIPOL1
LCORL
OGFOD2
SNX20
SLC25A44
SLC25A35
SCAF11
RPS6KL1
TRIM46
RNF141

UVSSA
ZNF165
EFCAB3
B3GNT4
CRISPLD1
CREBL2
APOLD1
ASXL3
GDPD3
GPR32
INO80C
KLHDC1
GPSM3
GSDMC
KIAA0753
ORAI3
LONRF1
RABEP2
PTRH1
SNX29
ZNF141
TMPRSS9
ZBTB4
ZNF764
C19orf48
C2CD2
CCN6
KLHL29
FAM171A1
FAM189B
L3HYPDH
OR9Q1
MEX3A
FOXI2
TMEM132C
SPATA19
WBP1L
UBAP2
ZNF747
FAM171A2
FBRSL1
MALSU1
TMEM158
SPTSSB
DCAF12
ATAD2B
C5orf24
C9orf24
IGSF9B
NXPE4

MICU3
MUC19
LZTS3
PROX2
VWA7
ZNF718
CYB5D1
C12orf40
NIBAN2
SSC4D
ZNF396
DENND2B
ARL17A
PABPN1L
NXPE2
LY6G5B
SCGB1D4
UPK3B
ZNF678
LDLRAD1
ARL17B
C8orf58
H2BC13
SMIM15
RETREG3
PRAG1
FAM200B
MFSD4A
SLC35G6
PLPPR1
RUSC1-AS1
DIRC1
C5orf52
RESF1
ZNF286B
CLEC19A
SLC49A3
STUM
ASB16-AS1
MINAR1
LINC02693
CFAP20DC
SNORC
LINC00324
LINC00479
LINC00476
OIP5-AS1
CYP21A1P
LINC01549
SNORA65

TMEM72-AS1
TUSC7
CTBP1-DT
HLA-DRB6
POU5F1P4
SNORA48
CASC16
CCNT2-AS1
HCG23
LINC00112
LINC02210-CRHR1
MAP3K14-AS1
SNORD10
SNORD88C
HTR2A-AS1
ITPKB-IT1
KANSL1-AS1
MSTO2P
LRRC37A4P
ZSCAN16-AS1
TRPM2-AS
LOC100505841
CHL1-AS1
DCST1-AS1
HLA-DQB1-AS1
MIR663AHG
LINC00456
LINC01307
LOC100129917
LOC100133091
LINC01500
MROH3P
MCCC1-AS1
UBBP4
C10orf95-AS1
GPR32P1
HLA-DQB3
ITPKB-AS1
LINC02067
LINC01869
LINC02471
LINC02527
LINC02582
LINC01127
KLHL7-DT
POLRMTP1
SMG1P5
LOC101927237
LOC102723566
LOC101928881

CDC37P1
HSD17B1-AS1
MIR4308
LINC01012
LINC02885
LINC02555
PWRN4
TDH-AS1
TSBP1-AS1
TPM1-AS
RERE-AS1
SMG1P2
LOC101929290
ENSG00000265749
EHMT2-AS1
HSD17B1P1
LINC02224
OR5AZ1P
LINC02400
LINC02451
LRRK2-DT
PSMC1P7
SIRLNT
TRI-AAT4-1
RPS3AP34
TRS-AGA2-6
TRT-AGT1-2
ENSG00000270361
ENSG00000271725
ENSG00000224505
ENSG00000251095
ENSG00000253194
ENSG00000253939
ENSG00000251456
LOC105370802
ENSG00000255439
ENSG00000260267
ENSG00000260947
ENSG00000259495
ENSG00000259672
ENSG00000262089
ENSG00000255046
ENSG00000260911
CICP26
GPR89P
GYG2P2
OSTCP8
LRRC37A17P
MTCO3P1
RNU6-835P

TRD-GTC2-11
TRG-GCC1-5
TRG-TCC3-1
RPS7P11
RPL7AP20
RPL7P17
RNU6-1169P
RNU6-418P
TRW-CCA1-1
ENSG00000271380
ENSG00000224361
TRP-CGG1-3
LOC100131289
LOC102725019
ENSG00000260304
ENSG00000260793
ENSG00000255495
ENSG00000228352
ENSG00000234181
ENSG00000236545
EEF1GP4
AKR1B1P5
GM2AP1
LINC01709
LINC02331
OR5BD1P
PABPC1P11
LINC02629
MTND1P24
PSMA6P4
RNU7-97P
RNU7-181P
THBS3-AS1
RNU1-138P
RNU6-1000P
RSL24D1P1
RNU6-566P
RN7SL813P
RNA5SP443
LOC339902
ENSG00000269947
ENSG00000287401
ENSG00000285800
ENSG00000226816
ENSG00000228318
ENSG00000228675
ENSG00000235497
ENSG00000257004
ENSG00000262372
ENSG00000254263

ENSG00000266929
ENSG00000201944
HMGB1P13
HNRNPCP3
HMGN2P18
FRG1CP
RAD1P1
RPL9P22
SRSF10P1
RN7SKP114
RN7SL474P
RPA2P1
RNU4-66P
ENSG00000207123
ENSG00000271267
ENSG00000273920
ENSG00000283321
ENSG00000285521
ENSG00000286074
ENSG00000273133
lnc-KCNJ6-6
lnc-CDC42BPA-5
lnc-FAM129B-2
lnc-LTK-1
RF00017-4451
lnc-TTC3-6
ENSG00000236654
lnc-RFX8-3
LARP7P4
NPM1P35
PHBP21
RNU7-137P
ENSG00000274238
ENSG00000277695
ENSG00000270892
ENSG00000288630
CYP17A1-AS1-001
HSALNG0035299
ENSG00000273734
ENSG00000276542
ENSG00000287463
ENSG00000288520
ENSG00000253790
ENSG00000250892
lnc-CCAR2-5
lnc-ELOVL3-2
lnc-GBA-1
lnc-ITPA-2
lnc-KANSL1-4
lnc-LMNB1-1

lnc-NMD3-2
lnc-NUP54-2
L13712-011
lnc-ADAM2-3
lnc-B3GALNT1-3
lnc-FAM3B-3
lnc-HLA-DQB1-1
lnc-MFSD4A-1
lnc-ASAP1-6
lnc-CERS6-1
lnc-CHD9-4
lnc-CTC1-2
lnc-DEFB136-2
lnc-IGF2BP3-1
lnc-OAZ1-3
lnc-PARP1-5
lnc-PRSS53-1
L13705-001
lnc-ATAD2B-6
lnc-BNC2-5
lnc-BTNL2-2
lnc-C2CD2-3
lnc-HLA-DQA1-9
lnc-HLA-DRB1-7
lnc-LSM7-3
lnc-MAPT-2
lnc-PITX1-6
HSALNG0102039
HSALNG0110820
HSALNG0049404
HSALNG0081496-001
HSALNG0103019
HSALNG0111460
HSALNG0081057
HSALNG0081496-002
HSALNG0114404
HSALNG0067898
HSALNG0081495
HSALNG0106460
HSALNG0114409
lnc-SNX13-3
lnc-TIAL1-6
lnc-WISP3-8
MG828730-042
NONHSAG010911.2
NONHSAG045793.2
lnc-WNT3-1
MG828730-058
NONHSAG022015.2
lnc-WDHD1-3

lnc-ZNF296-6
MN309207
NONHSAG019425.2
NONHSAG038251.2
piR-35530
piR-38051-158
piR-61955
RF00017-5002
lnc-RPS27L-8
MG828730-053
MN309549
NONHSAG017121.2
RF00017-4457
RF00017-5006
piR-51401
RF00017-3888
GS1-124K5.11
ENSG00000257852
ENSG00000236942
lnc-RAB29-1
RF00017-5948
RF00017-5950
RF00017-7420
RF00994-1086
RF00017-7425
LOC107984669
LOC390618
LOC283172
AB372660-002
HSALNG0009976
HSALNG0011100
HSALNG0036510
HSALNG0036512
HSALNG0044670
HSALNG0016950
HSALNG0026464-002
HSALNG0032204
HSALNG0033063
HSALNG0036511
HSALNG0007472
HSALNG0013542
HSALNG0036503
AB330774-009
HSALNG0032205
HSALNG0034883
lnc-ATXN7L3-2
lnc-FDFT1-1
lnc-ESD-2
lnc-NEK1-1
lnc-NOS1AP-2

HSALNG0133206
lnc-EGR3-4
lnc-ELOVL3-1
lnc-IPCEF1-6
lnc-ARL3-2
lnc-BST1-1
lnc-IGSF9B-13
lnc-NUP54-1
lnc-PLEKHM1-10
HSALNG0110544
HSALNG0049423
HSALNG0049424
HSALNG0068604
HSALNG0105360
HSALNG0107648
HSALNG0063462
HSALNG0094684
HSALNG0106455
HSALNG0123161
lnc-SCGB1D4-4
lnc-SPTSSB-2
lnc-ZNF668-1
piR-32325-169
piR-36037-004
piR-38580-304
lnc-UBAP1-6
piR-39703
MG604298
NONHSAG040481.2
piR-35179
piR-37106
piR-37170-008
piR-37213
piR-46381-087
piR-59412-001
piR-60985-091
RF00017-5320
lnc-TOM1L2-7
piR-36441-001
RF00017-3971
piR-57801-025
LOC105370052
LOC105377286
LOC105376281
RF00017-7019
LOC105373117
LOC105371265
LOC105378347
LOC107985959
LOC107984642

HSALNG0030267
HSALNG0007556
HSALNG0007557
HSALNG0009970
HSALNG0031133
1EI2_A
DQ866751
HSALNG0019031
HSALNG0023066
HSALNG0034884
HSALNG0035796
LOC105371449
LOC105371702
HSALNG0141624
lnc-CCDC73-1
lnc-CNOT7-1
lnc-DCAF12-1
lnc-CPLX1-12
lnc-FANCM-11
lnc-FGD4-1
lnc-NEU2-2
lnc-C12orf40-2
lnc-C19orf48-4
lnc-DEFB136-3
lnc-FAM189B-3
lnc-NOL4-3
HSALNG0129429
hsa-miR-5095-131
hsa-miR-5095-397
lnc-CHD9-9
lnc-DNM1L-3
lnc-GPRIN3-4
lnc-PGF-1
HSALNG0052803
HSALNG0089398
HSALNG0105667
HSALNG0106243
HSALNG0053116
HSALNG0064748
HSALNG0049343
HSALNG0114313
HSALNG0114981
HSALNG0114983
HSALNG0116733
HSALNG0070819
HSALNG0078606
HSALNG0116912
piR-35674-050
piR-30963-002
piR-31199-012

piR-32246
lnc-SCGB1D4-5
MN298528
piR-32606
piR-33614-160
piR-42219
piR-42694-036
piR-61945-390
RF00017-1479
RF00017-2222
piR-43401
piR-43864
piR-45471-470
piR-47062
piR-50357-077
piR-57845-019
piR-59109
piR-60516
MG828668-121
MK280073-211
MN296910-006
piR-45438
piR-52261-023
piR-55809-038
piR-57133-264
piR-57133-539
RF00017-2219
RF00017-317
piR-43106-096
piR-43134
piR-51036
piR-54316
piR-56480-026
piR-61157-011
LOC105369180
LOC105369506
LOC105377329
piR-41828
RF00017-7024
RF00994-103
RF00017-7037
5MWI_A-028
GU228580
lnc-ARID2-7
HSALNG0087326
HSALNG0066252
HSALNG0083491
NONHSAG026010.2
piR-37895-114
piR-32214-089

RF00017-7235
ADCY1
RIPK1
IL1RAPL2
C1QB
NAT1
TRPM8
APOH
GOPC
NPPC
XCR1
FBXO41
SLC35G1
COX6A1
COX7B
PPP2R5D
F2R
SOD3
IGKV2D-29
ODC1
USP7
LGALS1
SH3KBP1
COTL1
SEPHS2
TIMM10
ELK1
ANKK1
STAT3
MT2A
TRADD
SELENOW
GYG1
TIMM9
GNAS
TUBB2B
TUBGCP6
PSMA8
TUBGCP3
ATP5F1D
EZR
SLC2A4
FZD1
PTPN5
PPP2R2C
JUND
SOCS6
OXA1L
SCYL3
RBP1

SELENOS
PABPC4L
NORAD
HNF4A
PNP
LOC108660405
ABAT
MAPKAPK2
KCND3
CD8A
SYNGAP1
FKRP
RAP1GAP
TNS1
SOX18
SESN1
ATP6V1E2
ICOSLG
RABEP1
GANC
PCDH17
IMMP1L
PRELID1
METAP1D
NOA1
MIR548AW
CAMK2B
PRKACG
SLC18A1
KLC3
SLC25A31
UBD
TUBB8
COX7A1
TUBE1
UQCR11
CALML5
NDUFA4L2
TUBAL3
CALML4
CALML6
COX7B2
COX8C
TUBGCP5
COX6B2
SEM1
ATP5MC3
ATP5MC1
UQCRHL
HDAC1

PLEC
ANXA2
HSPA2
ELAVL1
ITGAL
KIF1B
HTRA1
YIF1A
SCAANT1
IDH1
YAP1
TIMM8A
FBP1
GALT
TXNIP
MIR410
CHCHD3
GLO1
AVPR2
AQP2
AMPH
PNPLA2
MPZ
PNPLA8
GJC2
DDHD1
GTPBP2
TMEM67
CHERP
DHFR
GCG
RNF31
TOMM7
MMP2
PRKAB1
GCLC
ARPP19
STAT1
STXBP2
USP14
YARS2
BMP6
COX15
MMACHC
GARS1
TMED2
STMN1
CYP7B1
PDSS1
TRAK2

FTMT
CCT7
PDIA6
GNAO1
KDM1A
LASP1
MGME1
H3C1
RPS14
SART1
ENSA
RPS8
DDX47
CDK6
TNFRSF10B
GCDH
LRG1
IBGC1
LMAN1
THBD
CHRNA2
MCOLN1
ACP2
ATP6V0C
FDX1
CYBRD1
KLHL1
CLN6
FAF2
SPATA13
TPM3
GFRA2
JAK2
HGF
CCND2
GLRB
IL4R
CD44
F2RL1
PROM1
CNP
PLXNA1
CARS2
CA5B
PECAM1
ATOH1
GAN
ABI3
CA13
SFMBT1

TOR1AIP2
DCHS2
NBEAL2
DCLK1
RBCK1
SEC13
GABPA
TIGAR
SPTBN1
IMPDH2
XRCC6
FAM98B
ARRB1
PEMT
BAG4
RNASE2
STRBP
NECAB1
SIAH3
MCCC2
DBT
AGPS
PRKCSH
ABCE1
RALY
ATAD3B
HSDL2
ATP5MF
VRK1
KCNB1
ARHGEF2
NR1I2
PPT1
CHD2
COL4A2
MYO5A
TDP1
UNC13D
ELAC2
DARS2
KDM2B
PEX19
TPP2
EIF3J
RBM17
NIF3L1
MRPL18
KLHDC4
STK31
RBM19

LIX1
BEAN1
SPAG8
KDR
CTPS1
NUP62
ELP2
INTS2
FKBP5
PIAS4
SEC31A
IFI30
DMTN
ADAP2
LAMB4
SIN3A
ATP2B1
HNRNPD
CYB5R1
CALU
HDGF
RCN2
CKAP4
CNDP1
UGCG
SETBP1
CDKL2
NBPF3
USPL1
MUCL3
HTR2B
SCD
PPP1CA
EEF1B2
TOM1
TPM4
HNRNPH3
PRPF19
MRPS18B
CCND1
KIT
ALK
MYH9
MGMT
NF1
BRD4
BIRC5
NKX2-1
WEE1
ARID1A

FOXM1
DSG1
AHSG
DGKG
MID1
RNASEH2A
ROBO3
CDK13
RNASEH2C
ELP4
NEURL1
TMSB10
DNAJC17
MRPL45
MRPS26
ELP6
PPIA
ATP2B3
CASP14
SEC24C
GRB10
RPL22
EIF4B
PSME2
CBX1
NSFL1C
MRPS7
VAT1
ZC3H15
RGS6
DUSP19
ACTBL2
ZRANB2
DIABLO
NEDD4L
SND1
RAB5C
GABBR1
GABRB3
ITGA4
GABRB2
WNT10B
CETP
PDE6A
SLC12A5
PDCD1
DLL4
CACNB4
AMPD1
CLCN2

CTSF
CFLAR
F13A1
ELOVL4
HCN1
GABRD
GABRA2
TYRP1
CTNS
CSF2RA
KCND2
HAND2
GABRB1
TBX2
SLC1A6
PRF1
STEAP3
SGSH
NOTCH4
ATP6V0A4
CTSZ
MGAT1
SLC46A1
CYP7A1
HEY1
DCT
GZMA
FGF4
RARS2
PEX3
TBX1
SEPSECS
SLC13A5
DLL3
CD63
HPS1
MLC1
MTO1
TBR1
TBX15
TBX18
TBX4
SLC35A2
VTI1B
EFHC1
CLN5
CEP290
FLVCR1
GPR55
MOCS1

PEX26
TBX19
SCN7A
STEAP2
RIMS2
DEPDC5
HEY2
PCDH12
KIF7
TBX6
SLC25A17
FBXL4
HPX
HPS6
KCTD7
JPH1
MADCAM1
STEAP1
STAMBPL1
STRADB
TRMU
IFNK
CDK5RAP1
PNPLA4
PEX11A
HJV
GDF1
JOSD1
OSGEPL1
POU3F1
TBX10
SNX4
JOSD2
NCDN
ZNF346
GPR22
IFITM5
MOB3B
MTERF1
TMCO5A
ARRDC4
ATXN1L
JPH4
OR4L1
TMEM199
SLC10A6
SPP2
TRMT61B
ABTB2
CSRNP3

NKAIN3
SPRN
SCNM1
YRDC
MRM2
CDIN1
ATXN7L3B
OR1L6
PROSER1
MEIG1
TMEM252
VRTN
BSPH1
PROB1
C2CD6
LINC02694
MT-TD
TRL-AAG2-3
ENSG00000235775
LOC108663993
LOC109461484
TH2-LCR
LOC108660406
LOC109286556
CASP4
VHL
TGM1
DUSP3
AGRN
SRR
NCALD
FIBP
ACYP2
WSB1
CPLX3
GOLGA4
KIAA1191
IQCD
SEPTIN3
RETREG2
MIR103A2
ACTA2
TRPC1
TPCN2
VGF
NIPSNAP1
HSPA7
STK26
PGM1
ACSL4

ARRB2
NGLY1
PPP1CC
LAP3
FABP7
ETHE1
LIMA1
YKT6
TXNDC5
USP48
ABHD10
UBL4A
H3C14
MET
DNMT3A
TERT
AXIN2
NRAS
VWF
GLI2
LIG4
TRPC3
COL1A2
KDM6A
PAX3
PAX5
PPM1D
CDKN2C
F8
GLRA1
MYCN
TNFSF10
ZIC1
TRPA1
TRPM6
GJA8
HPSE
RASSF1
SLC20A1
WRN
BTD
BMI1
AMD1
KCNH5
NCOR1
RNASEH1
PDGFA
PDGFC
PIGK
SOX17

SOX4
DNA2
CD1A
ELAVL2
HHIP
OTX1
MEGF10
PHOX2A
SPINK5
CSAD
CD1C
IL13RA2
GFI1
CXCL13
GPR161
HIC1
PCGF2
NALCN
MYT1L
ME3
TREM1
PPY
TTC19
DAB2IP
DOCK4
CD1B
CD1E
FLG
DMBT1
HPR
HEPACAM
SHBG
HSPB3
PARD6G
LONP2
POU4F1
SIRPB1
RPH3AL
ARL13B
IL4I1
HCST
MIA2
RAD51B
TREML1
TREML2
DEFB4A
ATRN
COX14
CPXM1
FUT4

SYNM
SGSM1
WDR83
ZNF750
P4HTM
TUT1
PRICKLE4
ANKMY1
ADAT3
CDIP1
CTU1
FSTL5
MPZL3
RBM24
TANC2
VSTM2L
CLEC5A
BARHL1
KNL1
EPGN
ELP5
FRRS1L
KCTD6
JKAMP
MS4A4A
LMLN
MXD3
SNTG1
ZFP3
ZCWPW1
OR4X2
PTPRQ
TANC1
KCTD21
PRR12
SMIM19
TREML4
TMEM245
CYS1
FAM166B
ZSWIM7
SPRR2D
IER5L
CBARP
MYCNOS
MIR219A1
MIR218-1
RPPH1
MIR383
SNORD53

SNORA12
CCAT1
VAC14-AS1
MIR548D1
MIR4274
IBGC2
PCK1
ACAN
ALDH7A1
HSD17B4
PCK2
PTK2
FEN1
SRPK1
PYCR2
DDX1
PTGES3
CYB5B
ARCN1
SAR1A
NUP93
PTPN23
STK25
COPS7B
EIF3G
GCNT2
CLPP
TGM3
AGK
AMY2B
CEP55
FYCO1
LPO
PIP
RNF32
GSDMA
MRPS15
PABPC1L
IGHG1
EMC8
HNRNPCL1
H2AC1
IGHA1
H4C11
DMAC1
EEF1A1P5
IGKV2-30
CALM1P1
PDE4B
MMP12

CCNL1
BMPR1B
MAPK7
PRKCI
STAT5B
ASS1
UBE2C
SETD2
UBE2I
YY1
ACY1
ALDH4A1
UBE2B
ACAA2
ARPC1B
ATP6V1E1
DCXR
HMBS
CPOX
MCFD2
UBE2T
CISD2
ANXA6
S100A8
VPS33B
ECH1
BRD3
AK4
MAT2B
EIF2A
PPP1R9B
GLRX5
NUDT9
SNRNP70
ECI1
AGO1
ABHD11
ATL3
MRPL1
ZW10
CHID1
CYP20A1
ERH
IPO4
EFHD2
JAGN1
UBL5
RALYL
TRIM41
RER1

WDR46
ISOC2
KYAT1
MMGT1
CHTOP
PRRC1
TBC1D9B
GATD3
BTK
MORF4L1
INTS3
SEPTIN9
FANCA
MYD88
PRKCE
CHRM4
CSNK1G2
ADRA2B
PTPN3
SIRT5
APOC3
CSNK1G1
ICAM3
NMUR1
MSRB2
RCBTB1
CDC42EP3
TMX3
RILP
DNAJB8
ANKS4B
SERF1A
ENO3
SMAD3
CTNNA1
PRPS1
EIF2B2
SERPIND1
RPA2
EIF3F
PRPF4
ASPSCR1
HAGH
NLN
ECI2
COPZ1
IK
FIP1L1
NOLC1
PLRG1

EIF3M
TWF1
CKMT1B
COPS8
IFRD2
FUBP3
LUC7L2
RTF1
PFDN2
PRPF38A
INIP
HMCES
QARS1
H2BC1
PYM1
FAM168B
AKT2
CHEK2
PTPN11
KRAS
FLT1
GSN
MAPKAPK3
PIK3CB
PTPN6
CUL3
GPT2
PSAT1
SLC25A20
CDK7
HELLS
ERCC4
PAK2
PKN1
BCAT2
ADARB1
AICDA
ALDOB
COL4A6
CTBP2
CDC7
GALE
KPNA2
PAPSS2
PBRM1
METAP2
LIG3
RAP1B
SRPK2
PPP5C

PPP3CC
PPP2R5C
USP13
ARF6
KIF2C
PARVA
MPI
WDR5
CACNB1
ACTL6B
BAIAP2
ARHGDIB
IRF2
GTF2E2
FERMT3
FGF12
NAGA
SGK2
SERPINB2
WASL
CLK3
CSRP1
CCNG1
KIF3A
ERC1
FTSJ1
KPNA4
ORC4
MRPL3
MARS2
MYL1
RAP1GDS1
SULF1
SMYD3
TBC1D7
RPL31
NLRP2
TARBP2
USP5
DYNLRB2
CHAF1B
CLP1
CIAO1
ARHGAP17
KIF4A
DHX16
EIF6
GYG2
EIF2S2
OXSR1

POLR2F
RAB14
POLA2
POLE3
TAF6
TBCD
STK16
SENP2
RIOK2
PPP2R5E
PLIN3
KIFC1
CDK3
DDX18
EPS8L2
HBS1L
RASA3
TRAPPC3
WAC
DPP3
BRWD1
BTF3
ACAD10
ABCF1
ACTR1B
CIAPIN1
CSTF2
ARFIP2
COPB1
ANXA9
GGCT
DECR2
GORASP2
GTF2E1
GTPBP1
FERMT2
GOSR1
KCNAB3
PELP1
NUP50
NFYA
SUPT4H1
TAF9
RGS3
RPL37
NECAP1
TRMT2A
YTHDF2
USP47
EIF1

DYNLT1
DIDO1
BUD31
AIFM3
ALDH8A1
CLIC3
CMAS
AP2A2
APEX2
CXCL14
DDX19B
EXOSC7
GSPT2
EXOSC1
OXSM
NAPA
MRPL24
MRPL28
MRPS6
NOB1
MOB1B
MED30
POLR3C
NTMT1
MCM10
LYPLA2
SSBP3
SAP30BP
SETD3
SF3A3
ZC3HAV1
WARS1
DCTPP1
ECHDC1
DUSP12
DNAAF2
CALCOCO1
CHRAC1
CNPY2
CFDP1
EXOC3
GNL2
HIRIP3
HP1BP3
FAM50A
MND1
MRPL10
LSM6
MED24
PYROXD1

PDS5B
TCEA1
TCF19
SIVA1
SPAG6
ZMYM3
TRUB1
UBAC1
ZADH2
DIRAS1
FCHO1
DDX55
ERMN
GCC1
MRPL20
PTMS
RAD51AP1
NAA25
SURF2
PATL1
VPS37C
ZCCHC8
UPF3A
DCTN6
DDX43
DYNC1LI2
DNAJB14
DNAJC8
BBX
ARGLU1
ARMC1
BOLA1
IMP4
DHRS7B
FOXJ2
NECAP2
MRPL48
MRPL49
MLLT6
MED8
PWP1
PTBP3
SP140L
TAF1B
RCOR3
SRXN1
SLC4A1AP
ZNF622
DUS3L
C11orf54

CARS1
ANKRD13D
BLOC1S2
ANKZF1
ENY2
NUSAP1
PRPSAP2
SYF2
QPCTL
TIPRL
RRP15
RPF2
PRR16
PDAP1
TCP10L
AIDA
CWC15
C9orf78
HDDC3
INTS12
OGA
MED19
MESD
RASL11B
RBM42
RASL10B
NSRP1
TSSC4
WDR89
TRPT1
COMMD2
METTL2B
MED11
UTP4
TRMT1L
CAAP1
CFAP58
C7orf50
HYPK
CFAP298
MRPL58
MVB12A
TCP11L1
RIOX2
RPUSD2
UTP23
ZC3HAV1L
C2orf49
KIAA1143
METTL16

TRIM52
TMA16
TCEANC
TMA7
SPANXN3
NCBP3
JHY
TRIR
UTP25
FRG1BP
CBL
DDR2
ITGA6
KCNJ11
AK1
GJB1
RASGRP1
TAB2
DCX
NOG
KCNJ8
CDC20
MSTN
SMURF1
NLK
SNAP23
SLCO2B1
EBP
HHAT
PDE1A
PAX1
KLF6
LCN2
PLK3
IL13RA1
DDX20
GLS2
GNA12
PELI1
SLC29A4
SH3GL3
SLC14A2
SLC38A2
UBE2H
PHF8
AGO2
CLDN3
CPLX2
EIF4E2
NMNAT2

RAB35
UBE2Z
UBE2D4
TET3
UBE2O
EPB41L2
MICU1
RAB3D
NUCB1
AUTS2
FN3KRP
HPS4
PARP3
RAB26
SNIP1
EOGT
NBR1
RAB3B
OLFM4
SDF4
STAU1
ABLIM3
GPR26
IL9R
HINT2
FAM20B
SYNJ2BP
SPATA7
RNF146
STARD7
PLAGL2
NKAP
JTB
CMTM3
OCIAD2
MICALL2
RAB43
SH3BGRL3
TMEM97
CCDC14
ARMCX3
C1orf127
IFNL3
ELMOD1
GIMAP8
TOMM5
RIMS4
LEPROT
OSCP1
LRRC46

TTC23
DTWD2
MIS18A
ACSM6
ZBED3
MRGPRE
UBL4B
SELENOI
SEPTIN1
TUT7
OR13C4
TDRD12
FDX2
TSGA13
PRELID3A
SRARP
LINC01104
LINC01432
CCT2
EPHA2
CFTR
ZAP70
PAK3
PIK3R2
TEK
PRKD1
ESRRA
HCK
POLH
IFNGR1
SLC2A2
TRPC6
ETS1
NR1D1
PDK3
NMNAT1
PLCD1
PTGIS
ZEB2
CCND3
ADRA1B
CDC25A
HPGD
FCGR2B
IFIH1
LFNG
KAT5
MEF2A
RASA1
PTGDR

PTK6
PTPN12
TLR1
TPMT
TTK
WNT4
UNG
ABCD1
ADRA1D
HDAC7
GNAT2
DDR1
FHL2
ERG
FUCA1
LATS1
P2RY2
MKNK1
RASGRP2
POLB
PTPRO
PIK3R5
TK1
RPS6KA5
SOX5
TGIF1
CACNG2
CAMKK1
ADM
BMX
IDO1
GNB5
GSTA3
FZD10
GABRA4
FOXP2
POU2F1
MDM4
MAD2L1
SLC17A5
SLC22A6
SEMA3A
TNFSF13B
RLBP1
PRDM1
VIPR2
ABCA5
ARNT2
CXCL10
GNAZ

GADD45A
FCGR3A
LPAR3
PIP5K1A
PEX2
NUAK1
MTMR14
SNAI2
SIK2
SOCS3
SMAD1
DUSP4
DYRK2
BCL11A
CRHR2
BDH1
ASXL1
DFFB
APLNR
FANCM
GMPR
GNA15
IL12B
KLK2
CRADD
CD74
EPB41L1
ETV4
HAVCR2
GSTA1
HBG1
GAPDHS
IFI16
NRIP1
P2RX4
NME3
KAT7
LNX1
POLL
PLA2G4C
PLCD4
TANK
PTPN7
SGK3
TLL1
SLC22A1
SELE
SECISBP2
RPS6KA6
RGS14

SDC1
SENP1
PLSCR1
SGCG
TNFRSF14
CACNG3
CLDN16
CEP57
CERK
AREG
CELF2
APOBEC3G
COL14A1
KISS1
CDIPT
CDCA7
DAPK2
HMMR
HOMER2
HS3ST1
FLCN
GALNT12
MYOT
P2RX6
NRCAM
MS4A2
MEIS2
MEOX1
LIPT1
KLF7
MCEE
MXI1
LCP2
RAB28
RASGRP3
POGZ
PFKFB1
NFKBIB
SOCS5
SIRT7
SHOC2
SPEG
SEMA3C
STK17B
RXFP3
PCNT
VPS37A
NDP
VAMP3
E2F6

DIO3
DYNC1I1
BHMT2
BRF1
ABR
BIK
ABLIM1
ACSL3
AGPAT3
ANKS1B
CDKN2D
CEACAM6
ARAP1
ASGR1
BCL2A1
ARHGAP9
BSND
FGF16
EMG1
CDK16
EME1
HOMER3
HOXB7
GRHL2
GADD45G
GNPTAB
JMJD1C
ITGB3BP
LAIR1
LGALS2
NDEL1
NTM
POLD3
NME6
MAST2
IL1RL1
MCC
LIMS2
PPIL2
PDZK1
NUAK2
PIBF1
MBD1
TBCK
SH3BP5
SKAP1
SIX2
ULK4
TNFSF14
TRIM63

TOB1
UGT1A10
TRAIP
ST3GAL6
RGS5
PCYT1B
RIOK1
PKNOX1
NMT2
USP6
TSPYL1
ZFYVE16
USP6NL
DLGAP5
AGR2
AKAP1
ACSBG1
CDKL3
CLEC3B
CD300LF
ARHGAP15
CPEB1
IFT81
FGFBP1
ING3
ELF2
CDK17
CDK18
ETV5
KIF3B
LIMD1
OAS2
ORMDL3
NME8
OSBPL5
PHLPP1
TAF11
RTKN
SIGIRR
RNF34
RFWD3
RNF138
PPP1R14A
CXXC4
DCDC2
DHX58
BLOC1S6
CAMKV
BCAR3
CRMP1

CEP250
ARHGEF5
ARMC9
ARHGAP24
ART4
C1D
CXCL16
KLK7
CTF1
CD58
DEF6
ELK3
HBZ
HSPB7
GAS2
GCHFR
ITGB1BP1
GRK3
HERC5
HBD
IFNW1
OLFM1
NEIL1
PAEP
NPL
ODF2
PARD6B
MOB1A
LMCD1
PLA1A
PLA2G4D
MAGEA4
MT1X
MAB21L2
SP2
RRAGB
ULK3
TRDMT1
TRAF3IP1
TRAM1
TRIM11
TIPIN
RGL1
RNF10
PRSS23
PPP1R3C
SSH3
SH2D1B
NOS1AP
ZHX2

ZFP36
USP12
DDX42
DEFA6
ACAD11
ACYP1
CASZ1
CBFA2T2
CCL13
ACBD4
ACKR2
CD300LB
AEBP2
ARID3A
BOD1
BDH2
ARID3B
ASXL2
DERL1
ANKS6
AP4S1
AFAP1L2
CFHR2
IER3
IFNA5
IL18RAP
FBXO38
FMNL2
KLF12
KCNK5
CENPT
DNALI1
CDCA3
EPB41L5
GIMAP4
GIMAP5
HOXC6
GPR12
IFI6
NARF
PAIP2
MSL3
KLF8
MXD1
FOXI1
TBC1D20
PSRC1
RP9
SLC35B1
UGGT2

TNIP2
TRIM69
TRIB2
TSPAN5
UBXN6
TRIM39
TMOD4
TIMM17B
RGS18
RETSAT
RNF185
SLX4
PLEKHG2
NMRK2
ZDHHC7
TASP1
TCEA2
ZC3HC1
ZKSCAN1
ZNF365
ZFYVE1
XAF1
DZIP1L
CAPNS2
CCDC40
CCDC28B
CST2
BRMS1
CRIP2
APLN
CCNB3
CCNB1IP1
IRGC
GLB1L
FKBPL
ERGIC2
CENPQ
CDC42EP2
CDC42EP4
CENPB
ENC1
GPR61
GSDMB
GTDC1
HAUS6
HMBOX1
GAL3ST1
KIR2DL3
IFNA8
FAAH2

IFNA21
LAD1
NAALADL2
PCDHGC3
MRPS18A
MT1B
MRPS24
LRRC1
ING2
MYL5
KPTN
RALGPS1
RASSF3
RASSF8
PPFIBP2
POMGNT2
OLFM3
MYOZ1
NELFA
LY86
LYRM7
MT1H
TCL1B
SUN5
SYT6
TBCC
TBC1D10C
SCEL
TOX2
UBL3
STARD10
RBPJL
RPA4
RMND5A
PLEKHF2
PPP6R2
VSIG4
ZNF10
ZRSR2
TSSK6
E2F8
DIXDC1
BEGAIN
ACBD6
CAMKMT
CCDC33
CLUAP1
CLEC2B
BET1L
ANKRA2

IGSF6
ETNPPL
FAM3A
EFS
LIMCH1
LANCL2
OSBPL11
OTUB2
PARP15
RAB2B
RAB40B
PELI3
NSD2
PHLDA3
TCP11
TAF7L
RSPH3
SH3RF2
SERAC1
ROPN1L
TFPT
SMAP1
PLPP3
USE1
VAT1L
EAF1
DSN1
DPCD
ECHDC2
BLOC1S5
CCDC115
CCDC59
AHDC1
AGFG2
AGO4
CINP
CMTM2
COA5
ARL4D
ARL6IP6
ASB6
AVPI1
ASMTL
ANXA8
APLF
IFNL1
IGSF10
FBXL7
GRAMD4
IL26

KCNMB2
KIAA0930
DEDD
ETV3
GNRH2
HIC2
GUCY1B1
FMNL3
FSIP1
GIMAP6
FOXS1
LETM2
ORMDL1
NMRK1
ODAM
NXT2
PARVG
MRAP2
MRPL34
MRPL35
MED9
MYL10
RBM4B
RASL12
RAI2
PUS7L
MT1G
MTG1
SYNE3
TCP11L2
SMG5
SLC41A3
TMCC2
SKA3
SLAIN2
TTLL6
TSSK2
TRIM47
TPD52L3
SPIC
SPZ1
SOX7
RNF115
RNPC3
PLEKHJ1
SLC48A1
STOML1
PLEKHO2
NKIRAS1
ZMAT3

ZNF2
ZNF22
YPEL3
ZNF35
ZNF581
ZNF75A
ZXDC
CYB561D2
DMKN
EBF4
DENND1C
DLK2
CARF
CCIN
ADAD2
CLIP4
ARHGEF40
ARL4C
ARL5A
ARRDC1
ATRAID
C1orf87
C5orf15
C15orf39
CFAP53
FAM114A2
FGFBP2
FNDC3A
FILIP1
KRT36
KIAA0825
CENPI
EPB41L4A
EVI2A
GPR78
HMGB4
GTPBP8
HIGD2A
KIAA0513
KANSL2
EEPD1
FAM151A
HAUS8
DTWD1
LACRT
LARP6
OR2W1
PHF20L1
PPAN
NIM1K

MIER1
LRRC8D
PRR15
RABL3
POP7
PLBD1
PDZD4
PCDHA7
PCDHGC5
MTCP1
MAGEH1
TBC1D21
PRSS33
SASH3
TTLL7
TSPAN13
TPRA1
TMUB1
STOML3
SPATA2
SPATS2
SPC24
STRIP2
PTTG2
RWDD2B
WDR88
NOL7
ZNF410
ZCCHC17
ZDHHC23
ZFC3H1
THAP4
ZNF343
ZNF777
ZNF839
ZSCAN18
ZSCAN5A
BEND5
CAPSL
CCDC106
CCDC107
ADAT2
CDK2AP2
CLDN20
CLASRP
CHAC2
ANKRD39
ASCL3
C10orf88
C3orf20

C9orf43
IQCH
KLK9
IGHM
IKBIP
KCNE5
DCUN1D2
GPANK1
FBF1
DGCR6
FAM32A
FAM126B
FRMD8
IFNA16
L3MBTL4
LARP1B
OCEL1
NXF2
PCDHGA12
MRPL52
MOGAT3
METTL8
LLPH
PRKRIP1
RAPGEFL1
PAGR1
PHYHD1
MZT2A
MTERF3
PRR14
TIGD1
TMEM53
TMEM255A
SNX21
TCEAL8
TP53TG5
TRIM16
TSKS
SERP1
RSRC2
TEX2
PHACTR4
WWC3
NECAB3
NOP16
ZCCHC12
UBXN7
ZNF740
DHRSX
AKNAD1

ANKRD45
ARMC7
C7orf31
KCTD18
ERVFRD-1
GPATCH1
GPN2
HINT3
FSD1L
OR7A5
NOL12
MRPL53
KLHL32
R3HDML
MTERF4
SYCP2L
TRIML1
SMTNL2
SPINK6
SNURF
RNF183
PCIF1
STN1
ZNF706
ZSCAN16
DUSP21
DRAM1
DNAJC4
C14orf93
CCDC15
AAMDC
CHURC1
C11orf53
BEND2
C1orf174
C1orf35
C3orf33
C6orf201
CRACR2B
FAAP24
HCFC1R1
HIKESHI
GUCD1
FAM53A
FAM90A1
FBXW9
FITM1
LENG1
PASD1
MPV17L

LMNTD1
LRRC42
OR4D6
PDZD9
LURAP1L
TICRR
UBXN10
SPX
SNRNP35
SRSF8
RITA1
PPP1R32
ZC2HC1A
TSNAXIP1
DTD2
CARNMT1
CATIP
CCDC144NL
ALKBH7
CCSER2
CGB3
C11orf52
C19orf33
C1orf131
C4orf46
C6orf118
C8orf33
DENND6B
CENPS
ENKD1
FAAP100
FAM131C
FAM200A
FAM133B
METTL25
LSMEM1
PP2D1
TMEM183A
SEPTIN12
RNASE11
RNF151
SLFNL1
ZNF322
ZSCAN1
ZNF720
C11orf71
C18orf21
C19orf18
C3orf62
CCZ1B

HCP5
H4C9
GAGE1
NUP62CL
MROH8
NXPE3
PROSER2
RPL41
PLAAT5
CIAO3
CIART
ODF4
STEEP1
CCDC149
CDPF1
DGLUCY
GOLM2
H2AC21
H2BC14
PIMREG
TAFA4
SMIM11
SPRR4
SERTM1
RSRP1
PLAAT1
PRXL2A
CAVIN4
C11orf45
C3orf36
FHIP2B
ILRUN
PAXX
LSMEM2
MARCHF10
NAA80
TEX37
SPATA46
SANBR
PHAF1
YAE1
DSCR9
BRME1
C22orf46
CSAG1
KRTAP10-7
INKA1
PABIR1
MTRES1
MFSD13A

PNMA8A
TMEM35A
RSPH10B2
RTL8C
TMEM267
RTL8A
TEX44
CFAP299
MIR30C1
SCAND2P
TP53TG1
TEPSIN
TERB2
TSPY2
TPRXL
VCY1B
WASH2P
TARP
WASH3P
AFG3L1P
MIR590
LINC00663
UBE2DNL
PRDM16-DT
BAALC-AS2
DLGAP1-AS2
VLDLR-AS1
OSTCP1
BIN3-IT1
HERC2P9
MIR4306
MYRF-AS1
SLIT1-AS1
LOC403312
DPY19L2P4
IL6-AS1
HLA-V
MT-TY
NUDT16L2P
TENM3-AS1
RNR1
LOC254896
MIR5701-1
CCT5
CYB5R3
CISD1
AP1B1
PICK1
CDK11A
MYOC

CPT1A
USP8
EPS15
ICAM1
PTK2B
CUL9
TPPP2
POR
SLC29A1
ALOX12
SARM1
ZNF219
ABCC1
PRKAR2A
ATP6V1A
SOAT1
LONP1
ARPC4
CYB5A
GJA1
SLC22A2
TOLLIP
BBC3
RAB32
BSN
OAS1
SV2A
PYY
FBXO32
TRIM22
TSC22D3
RBM6
MZF1
MIR148A
MIR6862-1
MIR6862-2
LOC110740340
NEK2
CTH
IMPA1
CARM1
FH
SLC1A5
USP10
FKBP8
PMPCB
RAD23A
LMAN2
SEC61B
ARL6IP5

TXNDC12
SPCS2
DLD
ACTN4
MAT2A
MYO6
GANAB
PGRMC1
ACP1
DYNLL1
SSR4
CAPZB
EEF1G
PSMD10
MLEC
PNN
MAP3K11
ETV6
PTGS1
RIPK2
ICAM2
NCOR2
PDE3B
HIPK1
MCPH1
FAM13A
ZNF202
PPP1R14C
ZCRB1
ZNF831
EMC7
SLC12A2
KYNU
TXNRD1
ACOX1
GOT1
DDX6
IDH3B
OXCT1
HMGA2
GMPS
RAD23B
IMPA2
AKR1B10
GNB4
GATAD2B
TAB1
ACTR3
KHSRP
HCCS

SLC23A2
NIPBL
BCAP31
ATG3
HSD17B12
PPIG
TAX1BP1
SSR1
UPP1
TXNL1
UBAP1
DNAJC10
PGLS
NENF
SNAPIN
WDR82
UBXN1
FOXK1
PAAF1
SBNO1
EMC2
TMEM109
ATP5ME
ATP5IF1
PSME3IP1
HLA-A
MAP3K7
HDAC3
CHRM2
STAT2
FANCC
KCNH1
STX1A
ATRX
GDF5
ALOX15
BCAR1
APOC2
FHIT
HSPA6
ANK1
FANCG
FGF9
GDAP1
EGR1
SLC32A1
CSNK1G3
ATP2C2
TAOK3
ASIC2

GPRC6A
ESYT1
MAB21L1
SRY
METTL21A
RILPL2
MARCHF5
MTARC2
RAB5IF
DMAC2
HTT-AS
TPTEP2-CSNK1E
ADA
ACAT1
AHCY
STK4
BUB3
OAT
SCP2
PYCR1
DLAT
AMPD2
ETFA
SMC3
CHD4
HINT1
SEC23A
STK24
DHRS4
AQP9
IQGAP1
NUMA1
BSG
ACTR2
ACAA1
ARF4
ATP6V0D1
HSPH1
KPNB1
POLR1C
AP1S2
RBBP7
SLC3A2
TPD52
DNAJB11
CAPZA2
HSPE1
MTCH2
PTMA
STX7

UPF1
POLR2H
RRBP1
DSTN
CORO1C
FKBP3
MTA2
TMOD3
RCC2
ACOT9
EIF3K
KARS1
NAP1L1
BASP1
RARS1
CUTA
MTPN
TMED9
LSM12
GHR
ATR
SMARCA2
ATP2A1
IMPDH1
ITGA5
MLH1
SLC16A1
RDX
GNA11
ATP1B1
ATP6V1B2
DDX58
FANCD2
ITGAV
P4HA2
PGD
PPARD
PDHX
PPP2R1B
ACADSB
AMT
ACADM
ABCC3
ALDH6A1
APOB
ITCH
DBI
GOT2
GPI
HMGCL

GRHPR
FLNB
NT5C2
KRT17
TPM2
UGDH
SLC9A3R1
PLOD3
STAMBP
PPP2CB
NME2
ZYX
AASS
AGL
BDKRB2
CUX1
CTSA
ATP2B4
CCNA2
CFL2
FDXR
EWSR1
HLA-G
MAN2B1
MINPP1
RALBP1
TBL1XR1
SLC29A2
TFG
ANXA4
GMDS
PYGB
PDHA2
SEC23B
RPL15
PPP6C
AK3
CMPK1
AP2B1
GIPC1
DDAH2
GMPPB
GNB2
HMGCS1
HIBCH
GDA
KALRN
GCSH
MSRB3
RAB2A

PNPT1
UGP2
RPIA
SLIT1
VASP
VPS4A
ZMPSTE24
UBE2V2
DUT
AFF4
AKAP8
CNOT2
ARPC2
ATP1B3
ATP6V0A1
IDH3G
CETN2
CDC42BPB
HBA2
HIBADH
PARS2
MBD3
MYL6
RANBP9
SARS2
PLOD1
VPS4B
ALDOC
CLIC4
ARF5
ARL2
ARPC5
CRTAP
ERP44
FABP5
GMFB
FLAD1
GNG12
IPO5
GTF2H4
PFKFB2
P4HA1
RAB6A
RAE1
PPIH
PPIC
PACSIN2
SDCBP
SIL1
WDR1

DNPEP
BPHL
BZW2
CDC16
GSPT1
RAB11FIP5
PDLIM5
SERPINB1
STX5
SMC4
ECSIT
DNAJC7
COPS3
COX17
ERGIC1
EXOSC9
HSPA14
HNRNPL
FKBP2
P3H1
NIT2
NRBP1
RBBP6
LYPLA1
SYNE2
TMCO1
SLC15A2
SCAMP2
SEC24A
RCN1
STOM
STRN3
PPME1
VPS28
WDR48
WDR61
ZNF638
CNN2
BOLA3
GRSF1
IPO7
NIT1
LSM5
RBM7
PFDN4
PDIA5
NUCB2
MDN1
MTCH1
RPL38

SRPRB
SSR3
SRSF11
SCG5
SDF2
QTRT1
ACOT13
CORO1B
INO80
EXOSC4
G3BP2
FAHD1
LACTB2
LAMTOR5
PAM16
MRPL23
MON1A
PEF1
SH3BGRL
UBXN4
REXO2
SGTA
VBP1
NIP7
ZNF207
SCCPDH
ACP3
ALDH16A1
H3-3A
HDHD3
HEBP1
NAXE
MAP1S
RBM25
PXDNL
TRIAP1
SEC22B
TTC1
COMMD3
COMMD4
HAUS4
NELFB
MACROD1
COA7
ESF1
SPATA20
PYCR3
DCAF1
CC2D1B
TXLNG

H2AC4
KYAT3
PUDP
CIAO2B
ADPRS
C5orf51
KPRP
SEPTIN11
JPT1
TYK2
COL1A1
IRAK1
SPARC
PTGER2
FAAH
KRT18
BIRC3
KRT8
NRXN1
TRAF3
PLCB2
ACADVL
CHRNA1
CA1
PDP1
OPRL1
NPY1R
ACSL1
AGA
CPT1B
KLK1
S1PR1
SLC1A4
SLC22A3
TNFRSF10A
USP1
CARTPT
HSPB8
PDK2
INHBA
SIX1
BCKDHB
CA6
CPT1C
RXFP2
CAPNS1
ACKR3
INSL3
GPR37L1
CDO1

MGST3
MIP
SPAG9
CYB561
EDAR
GFRA3
ELOVL6
MAPK15
SLC27A1
PCSK6
CALCOCO2
AADAT
RPH3A
BMP2K
OBSCN
LRRTM1
RAB13
CYGB
BBS1
MEX3B
MPRIP
MYL12B
SYNPO
TAAR1
TMEM30A
NDRG2
ACAP3
AVEN
DMRTA1
FAM111B
PPM1H
SPCS3
TRIM17
TMX2
TMX4
ZDHHC6
UPP2
CHCHD4
GPR6
EMC1
OTUD7B
TSPAN14
TPCN1
RHBDD2
GPR21
HACD3
FUNDC2
OSTC
PCDHB10
SCT

RHBDD1
EML3
GFRA4
FAM8A1
PEAK1
PCMTD2
SERF2
WDR5B
GSKIP
WDR83OS
MTERF2
SERF1B
MTARC1
LINC01006
MIR129-1
MIR129-2
MGC16025
LINC01239
DRD5P2
DRD5P1
RPL19P13
GUCY2C
EDN1
WHRN
MORF4
JAK1
NT5E
FZD4
PTPN1
ROCK1
SLC5A1
ADRB1
CXCR2
EPAS1
FLI1
SLC2A3
TNC
SLC6A9
RUNX1
ABCC2
GHSR
PRKAG1
ZEB1
CYBA
ACTG2
CLDN1
CA12
IL6ST
CDH5
CYP24A1

GRM3
FMO3
ITGB6
PAX2
MAP2K5
SLC4A1
TNFSF11
RHO
SLC5A6
SMAD6
PDE2A
BCAT1
ABCB6
ALDH18A1
ABCC4
ATP2A3
AVPR1A
GP1BA
DHCR7
CD59
ELANE
GNPAT
GALNT2
PARP2
NNT
KCNA5
LSS
PTPRB
RALA
PITX1
SLC16A2
SCARB1
SLC27A4
SLC4A4
RIT1
SLC5A5
EHHADH
BCKDHA
CA9
ALOX5AP
COL18A1
CUL5
FADS2
GJB6
FADS1
GLRA2
GNA13
ETFDH
IVD
MGLL

PFKFB3
TAOK1
TLE4
TDO2
SLC2A10
RDH11
SLC7A5
STT3A
SLC6A6
SLIT2
NOD1
TAT
CASP5
BTC
AVPR1B
ATF3
CRY1
ID2
CEBPB
GIT1
GYS2
PAX4
MMP20
ME2
LTBP2
MBTPS2
NSDHL
PIK3R3
TBC1D4
SLC22A12
SOAT2
PPP1R12A
NFE2L1
EDC3
DOK1
ALCAM
ABCC5
CIC
CNGB1
ATP6AP1
AUH
CA5A
CCNA1
CA14
COL11A1
COL6A1
F11R
ID1
KCNMB1
KDM5B

CD34
DIAPH2
DLGAP1
DDAH1
EIF4G2
HSD17B7
GRPR
GUK1
GRK1
FZR1
FMO1
JAM3
GRIP1
LETM1
LAMA2
PDE1B
POSTN
MLF1
LOXL1
LRP4
MIPEP
MBTPS1
PER3
NID1
MBD4
PTGES2
SMURF2
SLC28A2
SLC13A3
TRAF5
SLC5A3
SLC6A13
RIPK3
STX3
PTTG1
SLC7A2
TCOF1
DOCK7
EDEM1
ABCA2
CBX4
AAAS
ALG2
CLDN5
CLK1
ADPGK
CRLF1
BGN
ATP8A1
CX3CL1

AP3B2
ID3
GPRC5A
KDELR2
ESAM
HIP1
GSTM2
GSTM5
ETS2
GCAT
OPLAH
PMS1
MLKL
KLK15
RABGEF1
RAPGEF2
QPRT
NEUROG3
NFU1
MYOF
THY1
RP2
UBIAD1
SLC6A17
STIL
SLC7A8
TST
EDIL3
CACNG4
BCAN
BACH1
AGAP2
CLCF1
ARL1
ATP1A4
B3GALT4
ATAD1
CA3
IL11
CD80
GNG5
HGFAC
HMGN1
GPR17
FKBP10
KCNIP1
L2HGDH
MRC2
LSR
PTP4A2

RBM10
LUM
MAGI1
PRPF4B
PTGER1
TMEM43
SLC25A14
SPTB
REEP5
PRSS12
PPP1R15A
SLCO3A1
S100A1
SGCB
ZNF148
DOCK6
AGMAT
CHMP1A
CNTN4
CEP170
ANAPC11
AZU1
FCGRT
HIF3A
KDELR1
NPTN
KRT15
MCAM
TMEFF2
TBL2
SLC39A6
SLC4A3
SLCO1C1
SESN3
PREB
DAP
BAIAP2L1
ACP6
CLDN12
BAIAP3
ARHGEF16
APOL2
BST2
BBS9
CA7
ASPN
CA10
ISCA2
IL32
INPP4B

CDK11B
CXCL6
GOLGA5
HSPB6
FUZ
DOCK10
LALBA
NRN1
NPY4R
PAFAH2
NMUR2
LRRC4
MAP1A
MFSD2A
RAB27B
SYN2
TDRKH
TJP3
TMEM165
THEM4
SCG3
SLC24A3
SLC38A3
SLC39A1
SLC8B1
SLC9A9
SLC4A8
SLPI
PGRMC2
PPP1R10
PLXNB2
USP33
AKAP11
ACSF2
BTG2
C1RL
GHITM
KCNH4
CREG1
HMGCLL1
CCHCR1
OXER1
PHACTR1
PARP10
MPC2
LRP3
LSAMP
MYO1F
MYOM2
RAD1

RBM3
POMK
PDZD2
OGFR
SUMF2
SYNGR2
SLC39A12
SAMM50
SLC16A12
SLC38A5
TRIM31
RNF170
SLC7A3
SRGAP2
PLXNA3
USP42
AGR3
ACOT1
COA6
ASTN2
ATPAF1
ANXA10
ERGIC3
ELMOD2
HSPA13
FAM136A
KCNK17
KCTD3
GRIN3A
IBSP
FAIM2
OSGIN1
P3H2
MTRF1
RAB15
RAB3C
LYPLAL1
TM9SF3
SAMD12
RHBDL2
RNH1
PCYOX1
THAP11
ZRANB1
CARD16
ARHGAP19
DCTN3
G6PC1
FBXW8
ORM2

NIPSNAP3A
RANGRF
PILRA
SYPL1
SYPL2
SYNGR3
TMEM14C
STK35
SPCS1
RFTN1
PCP4
DUSP18
DNTTIP2
DNAJC11
COL22A1
CSN3
CEP85L
APOO
APOOL
FAM162A
HELB
FKBP9
ERP27
GEMIN7
HSPB11
GRIPAP1
LAMTOR1
PCBP3
MON1B
NIPSNAP3B
MLF2
PALM
NUDT19
NUFIP1
MAGEB2
TIPARP
TBC1D8
SEC11C
SCAMP5
RPP25
UBR3
SPNS1
RHBDL1
RHBDL3
REXO1
SUDS3
YIPF3
ZBTB10
ZNF804A
DSEL

CHRFAM7A
ARMCX2
FNDC5
EPPK1
HAUS3
H1-5
FOXRED2
MLLT11
LONRF3
MELTF
LYRM2
TMEM41A
SHISA6
SLC2A14
STON2
SPICE1
RHBDD3
RCN3
PRB1
VEGFD
TTYH3
CAVIN1
CACUL1
CNOT10
COA4
BPNT2
COQ8B
CCZ1
EMC10
DAOA
H4-16
HAUS5
FERD3L
HAUS2
MPPED1
MGARP
MXRA7
RAB19
NT5DC3
MT4
SVIP
TMEM186
TNMD
TMEM168
TMEM205
SMDT1
UNC80
C10orf90
BPIFB3
EMC4

OR1L8
THUMPD2
TMED7
TAAR8
ZNF782
C17orf75
GLIPR1L2
CEP162
NIPSNAP2
POTEE
TOMM6
RESP18
C1orf198
PDILT
RNASE10
PLAAT4
ZNF430
ATP5MK
GLMP
EVA1B
C1orf53
ANKRD34C
C2orf50
ERG28
FAM234A
MICOS10
PYURF
SELENOM
CBWD5
CRYBG1
POGLUT3
RMC1
MIR184
H2BS1
FMO6P
MIR192
MIRLET7A1
MIR191
HSP90B2P
MIR96
MIRLET7A2
MIRLET7A3
MIR196B
MIR181A2
POTEKP
MIR124-2
MIR181A1
FKBP9P1
IGHV3-23
MIR183

MIR186
MIR187
MIR124-3
MIR224
MIR323A
SLC22A20P
SOX21-AS1
HSP90AB2P
MIR188
RPLP0P6
HNRNPUL2-BSCL2
MIR595
MIR147A
MIR545
MIR563
MIR4487
MIR644A
UGT1A
LRRC52-AS1
ASS1P14
MT1XP1
VDAC1P1
LOC343052
EEF1A1P14
ISCA1P2
RPSAP11
ERDA1
RAC1P1
RPS15P6
TAAR7P
RPL29P32
RPL36AP26
RPL21P47
RACK1P1
LOC105376839
LOC109580097
OXTR
ARSB
FOXL2
UPF3B
MORF4L2
RARB
IDS
LTA4H
RAP1A
NHP2
PPM1G
CCDC91
GALP
ITK

TOP2A
NF2
YES1
DKC1
ARF1
ASNS
HEXB
NAMPT
MCM4
RPS6KA4
XPO1
DCN
DSG2
PKN2
TOP2B
SMARCE1
CYP2J2
ADD1
ADAR
CYP4F2
GALNS
NR1I3
MKI67
MAP4K5
PPM1A
PPM1B
TP53BP1
TMPO
EGR2
ARHGAP1
LMNB2
MCM6
CYP3A7
EIF2B1
ANP32A
EIF4A3
ETF1
FSCN1
POLR2E
RPL27
RPL35A
PDCD6IP
DTYMK
EHD1
BRD7
BANF1
CLTB
PAFAH1B3
LTA
MINK1

POLR2C
NAA10
TLN1
EEF1E1
BCLAF1
ACTR1A
HMGB3
KDSR
EIF3H
MDC1
SSRP1
TERF2
NOP10
ACOT7
AP3S1
AP3D1
MYH13
TTF2
UFM1
DDX39B
EDC4
AASDHPPT
ACIN1
CHMP2A
CSTF3
CPSF6
DAZAP1
DDX23
GGA1
GATAD2A
LARP1
NCBP1
MRPL13
SAP18
SAFB
RSL1D1
RPL32
SRP19
SFXN3
USO1
WTAP
DYNC1LI1
ALYREF
CKMT1A
CD2BP2
API5
IST1
HNRNPUL1
GAR1
GOLM1

NAP1L4
MRPS11
MRPS35
MYCBP
MYO18A
NUDT21
LUC7L
TAF5
THOC1
SUPT16H
THRAP3
SCYL2
RNPS1
VIPAS39
COLGALT1
CRNKL1
COPG1
NAA50
MRPS21
LDHAL6A
RANBP10
PSPC1
SAFB2
NDFIP2
YARS1
DDX19A
CHMP4A
CPSF7
DARS1
SURF6
TMED4
TNRC6C
EXOSC6
INTS5
PHF5A
SARS1
RTCB
PDCL3
TTC4
COPS4
PGBD1
GFUS
HNRNPUL2
HNRNPA1L2
RBM27
SNU13
RTRAF
PBDC1
SHTN1
TERC

POLR1G
PEDS1-UBE2V1
ZNF213-AS1
MORF4L2P1
MORF4L1P1
MORF4L1P3
KCNQ5-DT
MORF4L1P2
KHDC1-AS1
FGFR3
PIK3CD
DPYD
FLT4
BMPR1A
GCK
INPPL1
ITGB3
MERTK
RARA
PTPRF
ACVRL1
BLM
PMS2
POLE
MVK
PIP5K1C
SCNN1B
PLCB3
DIAPH1
FES
CTBP1
CDK8
CYP3A5
ERCC3
ERCC2
GLDC
GRB2
LAMB1
LBR
PYGL
MTAP
TLR8
TEAD1
CYP26B1
ADAM9
ACVR1B
AKR1C2
ATP2B2
AURKC
CSK

KRT5
ILK
EPHA5
CDH3
FAH
LIG1
MAP3K3
MAP3K8
PKLR
PIK3C2B
LYZ
PTPN2
PTGIR
TLR7
RPS6KB2
RRM2
TYMS
TFAP2A
STAT4
RFC1
SMAD2
PCSK1
DAG1
DOCK1
EHMT1
EIF2S3
CAMK4
ABL2
ALDH5A1
APPL1
ARHGEF1
ATF1
CCNH
CPS1
F5
IKBKE
FOLR1
CDC6
EPB41
ERCC1
GFPT1
GZMB
ITPA
HBB
NFS1
MYH2
RAD21
RAC3
POLA1
PHKB

PFKP
MAD1L1
PRKG2
PSPH
PSMB10
SORD
SCNN1G
ROS1
SOX10
PRCP
PRKAB2
PLOD2
PXN
SMARCAD1
SMARCC2
TGFB3
TRRAP
UMPS
DOT1L
EIF2AK4
ACAD8
CBFB
AIP
ALDH3A2
ADCY8
CKM
ARHGAP26
ATP6V1B1
CENPE
CPE
CCNB2
KDM4B
KHK
CDC45
CDC73
CYP2C18
EMD
GPC3
GFER
GMNN
GPD1
GALM
KRT1
NLRP1
NCK1
MMAB
MED12
MAGI2
MAP3K2
MAPKAPK5

IGFALS
IGFBP3
MVD
MYO5B
PRMT7
POLR1D
PEPD
NUP155
PIP4K2A
MTM1
SGPL1
TLK2
SKP2
SLC12A4
SAR1B
SELP
SEC24D
UGT2B7
TRIO
RFC2
SF3B1
ROBO1
PLAA
STAG2
PPP4C
NDRG1
DDX11
DDX41
DHX38
ISCU
DAPK3
GGPS1
HARS2
HAT1
KDM5C
KIF23
KRT16
POFUT1
POLR1A
MSH3
MYO1E
PPAT
PGAM2
PEX14
NUP214
SULT1E1
TACC3
PRPS2
PTPN13
SMS

SELENBP1
SEC63
RNASET2
SERPINB6
SF3B4
STK38
SMARCC1
SLK
VAV2
UPB1
DHTKD1
CALD1
CAPN9
ADD3
CKMT2
CLIC5
CD2AP
CSF2
ARPC3
CDK19
CORO1A
CCNK
CGA
FARSB
FNTA
INPP4A
CTNNA2
DIS3L2
DMGDH
CDA
CYP4B1
DCPS
GTF2H1
GTF2I
GRK4
EFEMP2
DRG1
LAMA5
KRT10
PARD6A
MNAT1
MRPS16
LPP
MAP4K1
MECR
MAN2A1
KPNA3
MYH3
LDHC
PRMT5

RACGAP1
POLE2
POLR2B
PUM1
NUP98
PALLD
NARS2
SULT1A1
PRPF3
SMYD2
SNAP29
UAP1
UBE3B
TP53BP2
TRAF4
TRIM33
SNW1
SERPINB5
SERPINB8
PRIM1
STAM
TERF2IP
SRP54
STT3B
SMAD7
SMARCD2
SFTPD
NFYC
XRN2
TAGLN
DPF2
DIAPH3
DPP9
EED
ADAM8
ALG9
AKR7A2
ALMS1
CHD3
CKS1B
CLASP1
ARFGEF2
ARHGAP4
BTAF1
ARHGEF11
ARID2
ATP6V1H
ATRIP
CUL4A
ANXA3

ANLN
AP2S1
IQGAP2
KRT2
KRT7
KIDINS220
KIF2A
EIF4G3
EIF4H
DGKI
EXOC5
EXOSC2
EXOC7
HLA-F
GMPPA
GTF2B
GTF2F2
HBA1
HLA-E
FAP
FERMT1
FLII
ITSN1
JMJD6
JUNB
DTNBP1
IFNA2
LCP1
OSGEP
NDC80
PAPOLA
NXF1
PMP2
POLR1B
METTL1
MOCOS
ME1
MED23
KRT20
MCAT
LDB3
PPIE
POLR2D
POMP
PUS1
PXDN
PIP4K2B
MTA3
PRKX
PTGR1

SCD5
RPL19
RPS5
SEPHS1
UBA5
UBE2F
UBE4B
TRMT1
TOPBP1
TSG101
TP53I3
TOP3A
SNRPE
STAG1
RNMT
S100A11
SBDS
SEC61A1
RNF7
SERPINB3
SETDB1
RPS29
RPS7
RPL9
PDCD4
PCYT2
STX16
SMARCD3
SFTPA2
PDCD10
ZFYVE9
TRNT1
UROS
XPO5
DYNC1I2
DYRK3
DPH1
DNAJC3
ACTN3
CHD5
CHD8
CLCA2
CLTCL1
CKS2
CKAP5
CNN1
ASF1B
ARPC1A
ATP6V1C1
ATF7

CDK12
CEACAM3
CSTF2T
CCNT1
CTSV
ATP6V1D
ATP6V1F
ATP8A2
AP1M2
ANAPC1
ANAPC10
COG4
EXOC2
ISYNA1
GGA3
GLMN
IL22
KDM4A
EPS15L1
DIS3
D2HGDH
ETNK1
EXOC4
EXOSC8
GFM2
GK2
HSD17B8
EIF4EBP2
GALK2
GLRX3
KDM2A
KDM3A
ITPK1
ICA1
GAST
GTF2F1
NCAPD3
NCAPH
NUP88
POLR2L
MPP1
NFKBIE
MORC2
MED17
METAP1
MSI2
MARCO
LMO7
MAP3K20
LLGL1

LLGL2
MAPK8IP3
MBD2
MTX2
MYH8
MYH1
LIMS1
RBKS
RBM4
RASA2
POLD2
PPL
POLR2J
PUS7
PDLIM1
PFN2
PAPPA
PGK2
PGM2
MYPN
NUP153
NFYB
MED15
SUZ12
TCERG1
TAF2
SLC25A32
SCAP
SCLY
SEC24B
UBA3
UCK2
TP53RK
TNPO1
TRIM2
TRIP13
TRAPPC6B
TOP3B
TRIOBP
SS18
RCOR1
RECQL
RPE
RHOC
PPP2R5A
STRN
SMCHD1
PHF6
VPS33A
WASF3

WBP2
XPOT
UPF2
ZMIZ1
WDR26
DHX36
ABI2
CAPG
CAPN11
CC2D1A
CCDC6
ACTR3B
CHMP5
COG8
CPVL
CPNE1
CWC27
CPSF1
CCT6B
IQGAP3
GINS1
FKBP1B
CDC40
DLX2
DDX50
HACL1
GRK7
OSBP
NUBP1
NAT10
NCAM2
NCBP2
NCAPG
ORC2
OTUD6B
PHIP
PARVB
MRPL15
MED25
MLLT1
KATNA1
LSM2
METTL3
MYH4
LDHAL6B
RAP2A
RAB6B
POLR3K
PPM1F
POLR3F

PITPNA
PIGT
MTMR6
TAF4
SWAP70
PSME4
NUP210
TLN2
SNRPD2
SH3PXD2B
TNNI1
TNPO3
TRA2A
TRIP10
TKFC
SLC9A3R2
RFC3
RPL24
RNPEP
PPP1R12B
TEAD2
SRSF5
VPS45
USP3
VIL1
ZC3H14
ZMYM2
UBR2
USP19
ECE2
DYNC2H1
DTNB
DYNLL2
DYRK4
EDF1
EHD2
BMS1
CCDC22
CAB39
B4GALT3
AGTRAP
ABCA13
AHCYL2
CHAMP1
CHMP1B
CLIC2
CLINT1
CLCC1
COG5
COG7

CTNNBL1
CEP41
ARHGDIG
ASPM
ATAD2
CRIPT
CPSF4
APIP
COLEC10
FNBP1
CSDE1
DMAP1
CDC23
DCTN4
DDX24
DDX46
EIF5A2
GOLGA1
HPS5
GEMIN4
GMIP
GTF2H3
GTF2H2
GAS8
GABPB1
GAPVD1
FKBP14
GNPNAT1
EHD4
EXOC6
DSC1
KPNA6
LGALS9
LAPTM5
NUBP2
NAPRT
NCAPG2
PAPOLG
NPLOC4
PDS5A
PHF20
POLR2G
MRPS12
MRI1
MED14
METTL13
MIB2
KRR1
LRRFIP2
LSM1

LSM3
MYEF2
MYH15
MYO5C
RABEPK
RAB3GAP2
RASAL1
RBM15
RAD54L2
POLR2I
PELO
PFDN5
PIGS
NFRKB
LZTFL1
SURF4
TAF3
TAF7
TARBP1
THOC2
TIMELESS
RPL18A
RPP30
RPRD1B
RPS4X
TSNAX
UBR4
TPD52L1
STAM2
SNRPD1
SRMS
SS18L1
SSR2
STIM2
RNF40
SAP30
RFK
RGN
RDH13
RECQL5
RPL29
RRP7A
PRC1
PPP1R2
PPP1R7
PDCD6
PDCD5
TES
STX12
SMARCA1

SMC2
SLMAP
WDR36
WDR4
TRIP6
ZNF24
TSR1
XPO7
XYLB
VPS25
WDR12
DR1
DNAJB5
DPH2
EBAG9
DNAJC2
BCAS2
BRD8
CCAR1
AMDHD1
ANAPC5
ANAPC7
CHMP7
CHAF1A
COG6
BET1
ARID4A
ARHGAP35
ASCC3
CRCP
CSTF1
CTR9
COPS2
COPS6
CPSF3
DERA
DERL2
AP3M1
CGN
HYAL4
IMP3
ILVBL
DNAH9
DCD
DDX31
DDX39A
EIF5B
ELAVL3
EXOSC5
HSD17B14

GPS1
HSPA4L
HEATR1
ERN2
EVL
FHOD1
GCC2
INTS6
KHDRBS2
IPO11
KCMF1
FOXK2
EXOSC10
DRG2
LCMT2
PAIP1
NLRP5
NSUN5
NSUN6
NANP
NCKAP1L
NCAPH2
PACSIN3
PDXDC1
PES1
NUTF2
NXT1
PAXIP1
MPHOSPH10
MRPL17
NOSIP
MOV10L1
MRPL40
MRPL46
METTL14
MAN2A2
LMAN2L
MED4
MED6
MEMO1
MTMR9
MYH7B
LHPP
RANBP3
RAP2B
POLE4
PPIL3
POLR3H
PPFIA1
PFDN1

PGM5
EXOC8
PIN4
NTAN1
LYRM4
MAGOH
SUPT6H
TAF10
PRPF40A
PSMG1
PTER
NUP205
SMNDC1
TIMM8B
SNRPA1
THOC6
RPL36AL
RUFY2
SIN3B
SAP130
TWF2
UCKL1
TPD52L2
UCK1
UGT3A2
TOE1
TOMM34
SNRPC
SNRPG
SNF8
SNRPF
SRA1
SPIN1
SEC31B
RFT1
RBSN
REG1B
PPP4R1
PPP1R13B
TFAP4
TFIP11
STRN4
SMAP2
SMC1B
REPS2
WDR11
ZNF224
XAB2
TTC5
XPO6

WDR33
DCTN5
EBNA1BP2
DPY30
DNAJB4
EAPP
DHX29
CBLL1
BCCIP
BCL7B
BABAM1
CCDC25
AFMID
AHNAK
ALKBH2
AMDHD2
ANKRD13A
ANKRD17
CLGN
ANAPC13
CRIP1
ARFIP1
ARHGAP44
C12orf57
ARID4B
ARPC5L
COPG2
COPS7A
CPPED1
CPSF2
ATP9A
AP1G2
COG1
IFT80
FLG2
KIN
CENPK
DHRS11
DDX49
DDX56
ERLEC1
GINS2
GNL1
GTF2A1
GSTT2
GTF2A2
GTF3C3
HMCN1
FNBP1L
FAM172A

EP400
GOLGB1
INTS4
INTS8
IPO8
EHD3
HERC4
GZMK
LEO1
PAF1
NCOA5
NPM3
PGP
OSBPL9
MRPS5
MMS19
MRPL32
NLRP4
MNS1
MRPS10
MRPS18C
MED20
MAP3K15
MFAP1
METTL5
RABGAP1
RAP2C
RBM12
RBM26
PNO1
POLR1E
POLR3E
PFDN6
PGAM4
OGFOD1
NUP37
NEMF
PCF11
NAA20
LUC7L3
MTMR12
SULT1C3
SUGT1
TADA3
PRSS16
NUP188
SF3A2
THOC3
THOC5
TDRD3

THUMPD1
TKTL2
SAMSN1
SLC25A33
SARNP
SCOC
SCPEP1
SH3BP1
TXNDC9
TXLNA
UBR7
TMTC3
SNRPB2
REXO4
SMC5
SH2D4A
PLP2
VPS51
VPS72
NDRG3
VARS1
WDHD1
ZDHHC3
YTHDC1
ZFR
UFC1
YTHDF1
YEATS4
EEFSEC
DNTTIP1
BCAP29
ABHD14B
CARHSP1
BCL7A
CCDC47
CCDC80
AHCTF1
CNN3
CHTF18
COG3
CSN2
AQR
ATF7IP
ADSS2
EXOC1
IRF2BP2
FKBP7
KRT77
DDI2
EPDR1

GLYR1
GNL3L
GTF3C1
H2AX
GSTT2B
HABP4
HNRNPLL
EVPL
GOLGA3
KIAA1217
IWS1
LCMT1
PARP16
NCLN
PAN3
MPP7
MPHOSPH6
MRPL22
MRPL38
MOB4
LRWD1
RANBP6
RASGEF1A
POLR3G
PHAX
PGM2L1
NLRP14
SULT1C4
PRPF18
PRPSAP1
PSMG2
QSOX2
THOC7
SNRPD3
SNX12
TBC1D23
TMF1
SAMD9
RPRD1A
RTCA
TSR2
TTI1
TNN
SSNA1
SNUPN
SERPINB13
PURB
PPP2R2D
PPWD1
NFATC2IP

TRMT112
ZPR1
YTHDC2
ZMYM4
VPS18
DCAF13
DDX52
DDX53
DDX51
DOHH
DNAAF5
DNAJA4
DPF3
DTD1
DNAJC21
ACTR5
CAND2
ACTR10
AARSD1
ANAPC4
AAR2
ACTR8
CMTR1
CLVS2
ARFGAP2
BRIX1
BTF3L4
ARAP2
ARL8A
ASCC2
CPNE8
CTTNBP2NL
APMAP
IRF2BP1
IRF2BPL
FBXL6
COMMD7
COMMD8
COMMD9
CDKN2AIP
EXOC6B
GINS4
HOOK3
HRNR
GTF3C5
ERO1A
INTS1
INTS10
INTS9
IFT52

IBA57
EIF1B
LAGE3
NMRAL1
NSMCE1
NSA2
PAK1IP1
MRPL51
MRPL16
MRPL37
MRPL9
NLE1
MRPL41
MRPL4
MED16
MED18
MTRF1L
RBM46
PPIL4
PUS10
PHRF1
PIH1D1
NLRP13
NLRP9
NABP2
MCTS1
MAGOHB
LZIC
MAGI3
SUGP1
TANGO2
SF3B5
SNX8
TM9SF4
SCML2
RRS1
TNKS1BP1
UBLCP1
TRAPPC5
SSU72
SPTBN5
SERPINB12
PPP4R2
PPP1R14B
SMC6
PLIN4
PPP1R11
NHLRC2
UTP14A
UTP15

UTP18
ZFPL1
TATDN1
TRMT10C
WDFY1
WDR74
ZNF593
DPY19L1
DSCC1
AK9
AHNAK2
ANAPC16
CHMP4C
CERT1
BRWD3
ARMC6
C15orf40
CPNE2
COMMD5
KIF4B
DBR1
EML2
GID8
GINS3
GET3
GTF2H2C
CCDC86
CCDC93
ELOC
FRA10AC1
LGSN
KNTC1
NAA40
NR2C2AP
PDRG1
PAXBP1
MRPL50
MRPL54
MRPL55
NOL10
MRPL47
MEPCE
MAK16
MED29
KRT82
RAVER1
RBM34
NACA2
NELFE
NIFK

NUDCD3
LTN1
MAGEB1
MED10
SYAP1
TAGLN3
SFSWAP
TMEM87A
THUMPD3
TMED3
RRP9
SCRN2
UBL7
TTC38
SNRNP27
SBK1
SEH1L
RPF1
RPL39
PDCD11
STRIP1
RWDD1
VPS36
NOC4L
NOL9
NPEPL1
URM1
UTP3
WIZ
ZWILCH
TRMT6
TRMT61A
ZNF830
WDFY2
WDR43
ZNF598
DCUN1D4
DDX60L
EFL1
DRC1
EHBP1L1
CCDC12
AKAP17A
ANKRD13B
ACTR6
CLUH
CLDN17
ASPDH
ATAD3C
COMMD10

COMMD6
CWC22
CGGBP1
ISOC1
FOCAD
INO80E
ERO1B
CDC26
DDA1
H2AZ1
GRWD1
HDDC2
HEATR6
NOMO1
NOMO2
NAXD
NAA35
PAPOLB
MITD1
METTL18
KRI1
MAU2
MEGF6
RABL6
RBM15B
NAF1
NGDN
NUDT13
MZT2B
NAA16
PRPF38B
PRPF40B
SPDL1
SAAL1
UBOX5
SPAG7
RNF113B
SDAD1
SETD4
PCID2
TEX10
PLPBP
ZC3H13
ZFR2
ZMAT2
TMPRSS11E
TCERG1L
UAP1L1
ZFYVE19
YLPM1

DCUN1D5
DPH3
ABRACL
CCDC97
BOLA2
ARMT1
CENPV
C9orf64
ISY1
FAXC
HEATR5A
PNISR
MRPS36
RANBP3L
RBMXL2
PLBD2
LTV1
RRP36
ZC3H18
ZNF512
WDR55
ZNHIT2
DNAAF11
CCDC124
BABAM2
AKR1B15
ANKMY2
CNOT9
BROX
C4orf33
CDV3
ELOA
HMGN5
H3C2
HEATR3
H3C4
FAM98A
KIFBP
KTI12
NAA38
NRDC
LIX1L
POLR1H
NUP210L
NSMCE3
NUDCD2
PRRG1
SAC3D1
SRPRA
RGPD5

RMDN1
RO60
NOP9
UTP20
BOLA2B
CFAP65
CFAP45
C19orf53
IRGQ
FLYWCH2
GDPGP1
H2BC11
H2BC4
H2BC5
H3C3
EIF3CL
INTS11
OTUD6A
NRDE2
PITHD1
PSMG4
RGPD2
RIDA
PPP4R3A
CIAO2A
C7orf26
EIF5AL1
H3C7
H3C10
H3C12
KNOP1
POLR1F
TARS3
SGF29
UBE2NL
STKLD1
SEPTIN10
PUM3
VPS50
UTP11
ZNRD2
CARMIL1
INTS13
PPIAL4A
RGPD6
PPP4R3B
QTRT2
VIRMA
VPS35L
ECPAS

H2AC8
H3C15
H3C11
H3C8
INTS14
MEAK7
MYG1
CZIB
DNAAF10
H2BC6
H2BC7
H2BC8
H3C13
INTS6L
JPT2
H2BC10
PTGES3L-AARSD1
PIP4P2
RTF2
ABITRAM
ETFRF1
RAMAC
SPINDOC
RCC1L
MIDEAS
TDRD15
YJU2
ARMH3
ARPC4-TTLL3
ISY1-RAB43
DLEU2
MIX23
MIR145
CHURC1-FNTB
H2AC19
GTF2H2C_2
PALM2AKAP2
RBM14-RBM4
RNF103-CHMP3
MIR335
GCNT2P1
HMGN1P30
RNU7-51P
H19-ICR
LOC108510657
LOC109610631
LOC110806306
LOC110673974

ADH1C
ATXN2
ATXN8OS
CHCHD2
DNAJC6
EIF4G1
FBXO7
GBA
GIGYF2
GLUD2
HTRA2
LRRK2
MAPT
PARK7
PINK1
PLA2G6
PRKAG2
PRKN
PSAP
SNCA
SYNJ1
TBP
UCHL1
VPS13C
VPS35
PARK10
PARK16
PARK3
PARK21
PARK12

PARK10
PARK3
GATD1
PARK7
PRKN
CUL9
PACRGL
PACRG
ZNF746

ACHE
ADRB3
MAOA
SRC
ADORA1
DDC
COMT
HRH1
MAOB
SIGMAR1
PDE4A
HTR1A
HTR2A
HTR1B
HTR2C
HTR4
ACACB
ADORA2A
DRD1
DRD2
DRD3
DRD4
FKBP1A
GRIN2B
NGFR
CHRM1
CHRM2
MT-ND3
PDE4D
SLC6A4
TH
ADORA2B
UGCG
SLC6A3
GRIA1
GRIA2
CHRNA7
BCR-ABL1
PTGS1
PTGS2
TAAR1
Cav
SNDR
PRKC
T-CaC
NET
GABRG3

GRIA3
GRIA4
CHRM5
CHRNA4-CHRNB2
VAMP1
VAMP2
GSK3B
AKT1
BRAF
MPO
PDE4B
PREP
HTR6
GCH1
GRM5
PDE9A
HTR7
RAF1
APCS
SNCA
TLR9
APH1A
APH1B
NCSTN
PSENEN
PSEN1
LRRK2
TRH
GLUL
GRM4
Malaria ACC1
nAChR
GRIA
NaC
PDE1
5HTR
ADR
NMDAR
OPR
MAO
KCNQ
FAF1
DR
GABAR
NQO

FLI1
HNRNPR
SEC23A
BLVRA
UQCRC1
MAGEA4
F13A1
MORF4L1
VPS4B
PDCD11
ACTA1
CSDE1
STRBP
UAP1
FBP1
MAPK9
FOXM1
S100A6
CTH
ALK
MKI67
NMRK1
CHID1
GOT1
TOP2A
PRKD2
USH1C
HSD17B4
VWF
GTF2I
ANXA3
CYP2E1
KPNB1
SATB2
SEM1
MYO6
NDC80
HMOX1
HBEGF
ESR2
CPSF3
STARD5
YARS1
CD1A
BCAT1
LYZ
NAGK
WARS1
HBB
TST

MB
HK2
IDH1
SKP1
MTRR
PRDX6
PMP2
GSTZ1
TP53
PTPN13
MAN2B1
ETFDH
ACADM
VAV2
PTPRF
METTL16
AR
LGALS3
LGALS9
PSD-95
SSTR2
ABCB1
FGF1
FGF2
HPSE
SLC6A2
CA14
ADORA2A
ADORA2B
ADORA3
PTPN1
SERPINE1
EIF4H
PABPC1
HRAS
MMP13
MMP1
MMP8
SLC29A1
FUCA1
GBA
HK1
HDAC1
MMP3
MMP9
ADAM17
ADK
AKR1B1
CA7
CA4

CA13
CA5A
TOP1
METAP2
PNP
OGA
CHEK1
ABL1
EPHA2
MAPK8
KDR
FGFR1
AURKA
BTK
EGFR
CASP3
GAA
HSPA8
CA3
CA6
MAPK10
DNMT1
ADA
PDE4D
CDK2
MCL1
MAP3K7
PSEN2
ECE1
MGMT
IMPDH1
IMPDH2
AGTR1
TK1
AMD1
CDA
MTAP
TYMP
AHCY
ATIC
ALOX12
GART
BACE1
SLC28A2
HCAR2
CCNA1
CCNA2
PSENEN
NCSTN
APH1A

PSEN1
APH1B
TNF
CD14
LBP
SYN

Ingredient	gene	Net
paeoniflori	FLI1	target
paeoniflori	HNRNPR	target
paeoniflori	SEC23A	target
paeoniflori	BLVRA	target
paeoniflori	UQCRC1	target
paeoniflori	MAGEA4	target
paeoniflori	F13A1	target
paeoniflori	MORF4L1	target
paeoniflori	VPS4B	target
paeoniflori	PDCD11	target
paeoniflori	ACTA1	target
paeoniflori	CSDE1	target
paeoniflori	STRBP	target
paeoniflori	UAP1	target
paeoniflori	FBP1	target
paeoniflori	MAPK9	target
paeoniflori	FOXM1	target
paeoniflori	S100A6	target
paeoniflori	CTH	target
paeoniflori	ALK	target
paeoniflori	MKI67	target
paeoniflori	NMRK1	target
paeoniflori	CHID1	target
paeoniflori	GOT1	target
paeoniflori	TOP2A	target
paeoniflori	PRKD2	target
paeoniflori	USH1C	target
paeoniflori	HSD17B4	target
paeoniflori	VWF	target
paeoniflori	GTF2I	target
paeoniflori	ANXA3	target
paeoniflori	CYP2E1	target
paeoniflori	KPNB1	target
paeoniflori	SATB2	target
paeoniflori	SEM1	target
paeoniflori	MYO6	target
paeoniflori	NDC80	target
paeoniflori	HMOX1	target
paeoniflori	HBEGF	target
paeoniflori	ESR2	target
paeoniflori	CPSF3	target
paeoniflori	STARD5	target
paeoniflori	YARS1	target
paeoniflori	CD1A	target
paeoniflori	BCAT1	target
paeoniflori	LYZ	target

paeoniflori	NAGK	target
paeoniflori	WARS1	target
paeoniflori	HBB	target
paeoniflori	TST	target
paeoniflori	MB	target
paeoniflori	HK2	target
paeoniflori	IDH1	target
paeoniflori	SKP1	target
paeoniflori	MTRR	target
paeoniflori	PRDX6	target
paeoniflori	PMP2	target
paeoniflori	GSTZ1	target
paeoniflori	TP53	target
paeoniflori	PTPN13	target
paeoniflori	MAN2B1	target
paeoniflori	ETFDH	target
paeoniflori	ACADM	target
paeoniflori	VAV2	target
paeoniflori	PTPRF	target
paeoniflori	METTL16	target
paeoniflori	AR	target
paeoniflori	LGALS3	target
paeoniflori	LGALS9	target
paeoniflori	HSP90AA1	target
paeoniflori	SSTR2	target
paeoniflori	ABCB1	target
paeoniflori	FGF1	target
paeoniflori	FGF2	target
paeoniflori	HPSE	target
paeoniflori	SLC6A2	target
paeoniflori	CA14	target
paeoniflori	ADORA2A	target
paeoniflori	ADORA2B	target
paeoniflori	ADORA3	target
paeoniflori	PTPN1	target
paeoniflori	SERPINE1	target
paeoniflori	EIF4H	target
paeoniflori	PABPC1	target
paeoniflori	SYN	target
paeoniflori	MMP13	target
paeoniflori	MMP1	target
paeoniflori	MMP8	target
paeoniflori	SLC29A1	target
paeoniflori	FUCA1	target
paeoniflori	GBA	target
paeoniflori	HK1	target
paeoniflori	HDAC1	target

paeoniflori	MMP3	target
paeoniflori	MMP9	target
paeoniflori	ADAM17	target
paeoniflori	ADK	target
paeoniflori	AKR1B1	target
paeoniflori	CA7	target
paeoniflori	CA4	target
paeoniflori	CA13	target
paeoniflori	CA5A	target
paeoniflori	TOP1	target
paeoniflori	METAP2	target
paeoniflori	PNP	target
paeoniflori	OGA	target
paeoniflori	CHEK1	target
paeoniflori	ABL1	target
paeoniflori	EPHA2	target
paeoniflori	MAPK8	target
paeoniflori	KDR	target
paeoniflori	FGFR1	target
paeoniflori	AURKA	target
paeoniflori	BTK	target
paeoniflori	EGFR	target
paeoniflori	MME	target
paeoniflori	CASP3	target
paeoniflori	GAA	target
paeoniflori	HSPA8	target
paeoniflori	CA3	target
paeoniflori	CA6	target
paeoniflori	MAPK10	target
paeoniflori	DNMT1	target
paeoniflori	ADA	target
paeoniflori	PDE4D	target
paeoniflori	PSD-95	target
paeoniflori	MCL1	target
paeoniflori	MAP3K7	target
paeoniflori	PSEN2	target
paeoniflori	ECE1	target
paeoniflori	MGMT	target
paeoniflori	IMPDH1	target
paeoniflori	IMPDH2	target
paeoniflori	AGTR1	target
paeoniflori	TK1	target
paeoniflori	AMD1	target
paeoniflori	CDA	target
paeoniflori	MTAP	target
paeoniflori	TYMP	target
paeoniflori	AHCY	target

paeoniflori	ATIC	target
paeoniflori	ALOX12	target
paeoniflori	GART	target
paeoniflori	BACE1	target
paeoniflori	SLC28A2	target
paeoniflori	HCAR2	target
paeoniflori	CCNA1	target
paeoniflori	CCNA2	target
paeoniflori	PSENEN	target
paeoniflori	NCSTN	target
paeoniflori	APH1A	target
paeoniflori	PSEN1	target
paeoniflori	APH1B	target
paeoniflori	TNF	target
paeoniflori	CD14	target
paeoniflori	LBP	target

Node	Type
FLI1	gene1
HNRNPR	gene1
SEC23A	gene1
BLVRA	gene1
UQCRC1	gene1
MAGEA4	gene1
F13A1	gene1
MORF4L1	gene1
VPS4B	gene1
PDCD11	gene1
ACTA1	gene1
CSDE1	gene1
STRBP	gene1
UAP1	gene1
FBP1	gene1
MAPK9	gene1
FOXM1	gene1
S100A6	gene1
CTH	gene1
ALK	gene1
MKI67	gene1
NMRK1	gene1
CHID1	gene1
GOT1	gene1
TOP2A	gene1
PRKD2	gene1
USH1C	gene1
HSD17B4	gene1
VWF	gene1
GTF2I	gene1
ANXA3	gene1
CYP2E1	gene1
KPNB1	gene1
SATB2	gene1
SEM1	gene1
MYO6	gene1
NDC80	gene1
HMOX1	gene1
HBEGF	gene1
ESR2	gene1
CPSF3	gene1
STARD5	gene1
YARS1	gene1
CD1A	gene1
BCAT1	gene1
LYZ	gene1
NAGK	gene1
WARS1	gene1
HBB	gene1

TST	gene1
MB	gene1
HK2	gene1
IDH1	gene1
SKP1	gene1
MTRR	gene1
PRDX6	gene1
PMP2	gene1
GSTZ1	gene1
TP53	gene4
PTPN13	gene2
MAN2B1	gene2
ETFDH	gene2
ACADM	gene2
VAV2	gene2
PTPRF	gene2
METTL16	gene2
AR	gene2
LGALS3	gene2
LGALS9	gene2
HSP90AA1	gene2
SSTR2	gene2
ABCB1	gene2
FGF1	gene2
FGF2	gene2
HPSE	gene2
SLC6A2	gene2
CA14	gene2
ADORA2A	gene2
ADORA2B	gene2
ADORA3	gene2
PTPN1	gene2
SERPINE1	gene2
EIF4H	gene2
PABPC1	gene2
SYN	gene2
MMP13	gene2
MMP1	gene2
MMP8	gene2
SLC29A1	gene2
FUCA1	gene2
GBA	gene2
HK1	gene2
HDAC1	gene2
MMP3	gene2
MMP9	gene2
ADAM17	gene2
ADK	gene2
AKR1B1	gene2
CA7	gene2

CA4	gene2
CA13	gene2
CA5A	gene3
TOP1	gene3
METAP2	gene3
PNP	gene3
OGA	gene3
CHEK1	gene3
ABL1	gene3
EPHA2	gene3
MAPK8	gene4
KDR	gene3
FGFR1	gene3
AURKA	gene3
BTK	gene3
EGFR	gene3
MME	gene3
CASP3	gene4
GAA	gene3
HSPA8	gene3
CA3	gene3
CA6	gene3
MAPK10	gene3
DNMT1	gene3
ADA	gene3
PDE4D	gene3
PSD-95	gene3
MCL1	gene3
MAP3K7	gene3
PSEN2	gene3
ECE1	gene3
MGMT	gene3
IMPDH1	gene4
IMPDH2	gene4
AGTR1	gene4
TK1	gene4
AMD1	gene4
CDA	gene4
MTAP	gene4
TYMP	gene4
AHCY	gene4
ATIC	gene4
ALOX12	gene4
GART	gene4
BACE1	gene4
SLC28A2	gene4
HCAR2	gene4
CCNA1	gene4
CCNA2	gene4
PSENEN	gene4

NCSTN	gene4
APH1A	gene4
PSEN1	gene4
APH1B	gene4
TNF	gene4
CD14	gene4
LBP	gene4
paeoniflori	ingredient

name	Betweenne	Closeness	Degree	Eigenvecto	LAC	Network
ABCB1	84.78253	0.445104	15	0.11253	8.266667	9.114719
KDR	778.9372	0.473186	26	0.145537	8.846154	13.88521
TP53	3628.012	0.576923	61	0.292382	12.98361	46.95277
EGFR	1721.395	0.535714	51	0.263939	12.66667	37.16892
AKR1B1	748.5586	0.436047	15	0.07224	4.4	6.569805
MGMT	87.86197	0.438596	15	0.106529	8.133333	8.811688
CASP3	1278.688	0.522648	47	0.256302	13.2766	34.54888
HSP90AA1	2907.347	0.530035	49	0.230923	10.32653	30.08155
TOP1	66.91201	0.432277	16	0.12031	10.125	11.03117
ABL1	104.6497	0.432277	19	0.13263	10.10526	12.32014
SLC29A1	207.5752	0.393701	12	0.034219	3	4.770996
MAPK8	93.84087	0.45045	21	0.142679	10.85714	14.51105
TNF	4169.228	0.541516	50	0.223486	10.08	32.34386
SYN	1141.133	0.520833	43	0.241438	12.88372	29.20056
HSPA8	698.2179	0.457317	25	0.122666	7.28	13.26347
CYP2E1	293.6364	0.426136	10	0.065964	5.2	5.916667
PSD-95	424.0755	0.462963	27	0.169169	12.22222	19.48926
CCNA2	149.7312	0.436047	24	0.161637	12.91667	17.75981
EPHA2	17.29302	0.402145	8	0.065796	4.5	5.25
PTPRF	39.59519	0.357995	4	0.022319	1.5	2
ACTA1	718.9454	0.437318	12	0.085324	5.666667	6.909091
MCL1	64.91758	0.445104	17	0.130977	9.764706	11
PTPN1	104.1723	0.436047	12	0.084013	5.666667	7.151515
AR	27.75176	0.445104	18	0.148972	12.22222	13.01176
IDH1	693.9112	0.445104	18	0.090867	5.111111	7.3312
HDAC1	714.675	0.457317	23	0.146096	9.478261	12.85005
ACADM	421.0463	0.328947	7	0.006598	1.428571	3.5
UQCRC1	0	0.248344	2	3.56E-04	1	2
FUCA1	30.18406	0.301205	3	0.001656	0.666667	1
BCAT1	708.1741	0.424929	12	0.025475	1.666667	2.657576
TST	57.66691	0.326087	5	0.005881	0.8	1
HSD17B4	322.9091	0.382653	5	0.018212	1.6	2.25
ETFDH	19.75242	0.283554	3	0.001157	1.333333	2.5
MORF4L1	3.687179	0.323276	3	0.012299	0.666667	1
VAV2	5.818445	0.364964	5	0.028708	1.6	2
MYO6	298	0.306122	2	0.003933	0	0
ADA	676.3788	0.424929	17	0.035388	5.058824	9.326407
AHCY	398.0666	0.415512	16	0.038176	4.5	7.644048
ATIC	201.1743	0.382653	13	0.019623	4.461538	6.013636
ADK	523.6778	0.390625	16	0.019849	4.5	7.648268
ADORA2B	234.3956	0.394737	8	0.016168	2.25	2.8
SLC28A2	0.622222	0.32538	4	0.005578	2.5	3.333333
IMPDH2	19.58455	0.35461	9	0.010583	6	6.958333
ADORA2A	607.5589	0.390625	6	0.015137	2.666667	3.2
IMPDH1	597.4147	0.344828	7	0.006699	3.428571	4
PNP	316.6163	0.406504	13	0.028186	4.923077	7.121212

ADORA3	113.527	0.397878	7	0.025429	2	2.333333
GART	891.8032	0.433526	21	0.046177	5.142857	11.50871
MTAP	199.2677	0.407609	9	0.022303	4	5.025
CDA	90.70382	0.408719	9	0.032054	3.555556	4.416667
ADAM17	261.2715	0.407609	14	0.065856	5.857143	8.367646
HBEGF	18.77582	0.428571	12	0.095004	8.5	9.272727
MMP8	1.39281	0.38961	7	0.058214	5.142857	6
NCSTN	1.037388	0.309917	7	0.010407	5.714286	6.666667
MMP3	38.98945	0.42735	15	0.101701	9.733333	11.12617
MMP1	34.92304	0.423729	15	0.102708	9.466667	11.41189
PSEN2	94.8948	0.361446	9	0.021885	4.888889	6.196429
PSENEN	1.037388	0.309917	7	0.010407	5.714286	6.666667
APH1A	25.87608	0.319829	8	0.011118	5.25	6.285714
MMP9	340.2694	0.470219	31	0.186199	12.12903	22.35097
PSEN1	553.013	0.422535	14	0.064858	5.714286	8.815659
BACE1	197.9451	0.397878	10	0.043031	5.8	7.55754
PDE4D	3.015037	0.363196	2	0.011991	0	0
SLC6A2	298	0.282486	2	6.98E-04	0	0
ALOX12	2.220513	0.313808	2	0.003777	0	0
APH1B	47.49795	0.320513	8	0.009344	4	4.809524
VWF	274.5482	0.421348	15	0.086122	6.8	8.022727
AGTR1	6.029366	0.386598	6	0.051169	3.666667	4.4
SERPINE1	49.096	0.433526	17	0.117545	10	12.8362
MAPK10	179.4754	0.406504	11	0.067387	5.090909	5.833333
YARS	254.236	0.375	9	0.01397	1.777778	2.583333
AMD1	18.09679	0.351288	6	0.012579	2.333333	2.8
EIF4H	129.8685	0.35461	8	0.015061	2.5	3.357143
LGALS3	425.6396	0.436047	15	0.094864	6	7.664286
MTRR	13.53818	0.34965	5	0.011553	2	2.5
CTH	128.9897	0.353774	7	0.008987	1.142857	1.6
DNMT1	399.0809	0.460123	21	0.138553	9.52381	11.40217
HMOX1	566.6899	0.446429	16	0.093771	6.375	7.454579
GSTZ1	22.07862	0.334076	5	0.008816	2	2.583333
PMP2	0	0.30426	1	0.00332	0	0
UAP1	368.6159	0.331858	7	0.008096	1.428571	2.666667
PRDX6	37.79571	0.364964	4	0.019956	1	1.333333
HK1	383.7472	0.393701	9	0.039135	2.444444	3.916667
HK2	463.55	0.421348	12	0.07615	4.666667	5.723485
ALK	126.9821	0.422535	10	0.074671	4.8	5.333333
PABPC1	472.9239	0.383632	10	0.026337	3	4.603175
FOXM1	45.96921	0.414365	16	0.113892	10.25	11.71338
FGFR1	19.07194	0.418994	12	0.091264	7.166667	8.041667
ANXA3	2.352036	0.382653	4	0.03607	2	2.666667
S100A6	8.049054	0.390625	5	0.034855	2	2.583333
AURKA	97.96639	0.432277	18	0.123774	10.88889	12.7402
FGF2	151.5936	0.445104	25	0.155018	11.28	17.65491
MKI67	138.6896	0.434783	19	0.137848	11.15789	12.96091

TYMP	204.2574	0.426136	12	0.055771	4.166667	4.875
TK1	634.784	0.423729	16	0.06198	4.375	5.293182
CCNA1	9.746242	0.388601	12	0.073344	8.5	9.545455
SKP1	103.2088	0.404313	13	0.071966	5.846154	7.503788
CHEK1	45.91222	0.423729	19	0.13515	12.42105	14.83912
TOP2A	122.0163	0.429799	18	0.114222	9.888889	11.62562
NDC80	1.075146	0.337079	9	0.050555	7.333333	8.25
GOT1	54.59899	0.339367	6	0.00813	1.333333	1.65
BLVRA	3.281997	0.330396	2	0.005482	0	0
BTK	62.57	0.421348	9	0.064431	4	4.5
GTF2I	69.86623	0.35461	6	0.021726	0.333333	0.4
CA13	0	0.213068	2	3.55E-05	1	2
CA3	592	0.269784	3	7.38E-04	0.666667	2
CA7	0	0.213068	2	3.55E-05	1	2
CA14	0	0.205198	1	1.42E-05	0	0
CA4	298	0.257732	2	3.09E-04	0	0
MB	891.2991	0.364964	4	0.015996	0.5	0.666667
CA6	0	0.265957	1	5.31E-04	0	0
LYZ	314.4257	0.361446	3	0.011553	0	0
MMP13	0	0.40107	7	0.061212	6	7
KPNB1	62.82106	0.391645	7	0.043241	4	4.666667
ESR2	32.41101	0.432277	13	0.10485	8.307692	9.566667
MAPK9	31.53445	0.429799	11	0.08337	7.090909	8
FLI1	3.095591	0.345622	4	0.02518	1.5	2
CD14	29.97001	0.364964	6	0.026754	2	2.4
LBP	1.833333	0.362319	6	0.036538	4	4.8
F13A1	3.286032	0.326797	3	0.011869	0.666667	1
FGF1	53.3772	0.421348	13	0.088541	7.230769	8.418398
MAP3K7	69.34262	0.398936	7	0.036131	2.285714	2.7
CD1A	0	0.352113	1	0.010255	0	0
SATB2	0	0.328947	2	0.01448	1	2
CPSF3	0	0.277778	1	0.001211	0	0
CSDE1	4.333333	0.286807	3	0.002577	0.666667	1
METAP2	106.0356	0.364078	4	0.014663	0.5	0.666667
ECE1	3.459367	0.3125	2	0.005367	0	0
HNRNPR	313.6594	0.363196	7	0.026894	3.142857	3.666667
FBP1	73.58889	0.402145	7	0.037165	2.857143	3.366667
GBA	530.3983	0.403226	6	0.027682	1	1.8
MAN2B1	16.56974	0.300601	3	0.001743	0.666667	1
GAA	98.87732	0.33557	5	0.008599	0.4	0.5
WARS	20.45414	0.334821	5	0.006779	1.6	2
PDCD11	22.55338	0.350467	5	0.009918	2	2.5
HBB	122.6357	0.394737	4	0.030381	1.5	2
HCAR2	0	0.352113	1	0.010255	0	0
MAGEA4	2.751675	0.333333	2	0.009557	0	0
NAGK	0	0.289575	2	0.00217	1	2
VPS4B	0	0.29703	1	0.003503	0	0

STRBP	0	0.266904	1	0.001236	0	0
PRKD2	0	0.347222	1	0.010598	0	0
SHFM1	0	0.347222	1	0.010598	0	0
LGALS9	0	0.357995	2	0.014616	1	2
PTPN13	25.61534	0.314465	4	0.005536	0.5	0.833333
METTL16	0	0.298211	1	0.002844	0	0
MGEA5	97.91458	0.40107	4	0.027903	1.5	2
NMRK1	1.733333	0.30303	3	0.002575	1.333333	2
USH1C	0	0.234742	1	1.80E-04	0	0
SEC23A	0	0.249584	1	3.71E-04	0	0
SSTR2	0	0.220588	1	3.19E-05	0	0

Betweenne	Closeness	Degree	Eigenvecto	Informatio	LAC	name	Network	selected
0.4	0.531646	6	0.089912	4.453992	4.333333	ALK	5.2	FALSE
2.995116	0.545455	8	0.10436	5.148054	5	HK2	5.714286	FALSE
15.93885	0.608696	16	0.181224	6.925563	9.75	CCNA2	12.02172	FALSE
18.65235	0.552632	10	0.07969	5.714752	4.6	TK1	5.668651	FALSE
26.99196	0.636364	18	0.192693	7.220809	9.555556	PSD-95	12.91461	FALSE
11.18287	0.552632	8	0.069168	5.148054	3.75	TYMP	4.47619	FALSE
0.953907	0.545455	8	0.09678	5.148054	6.25	CYP2E1	7.142857	FALSE
28.08401	0.6	14	0.13977	6.58454	6.714286	HSPA8	8.732501	FALSE
108.9139	0.736842	27	0.260917	8.172728	10.96296	SYN	21.67939	FALSE
13.39192	0.512195	7	0.048821	4.819485	1.714286	AHCY	2.066667	FALSE
6.98165	0.494118	6	0.033467	4.453992	2	ADA	2.4	FALSE
119.2635	0.711864	25	0.235177	8.001429	10	TNF	19.80521	FALSE
14.78226	0.591549	14	0.162749	6.58454	9	MKI67	10.34751	FALSE
11.40712	0.6	14	0.163122	6.58454	8.857143	MAPK8	10.88919	FALSE
9.690873	0.591549	14	0.164186	6.58454	8.571429	ABL1	9.893162	FALSE
5.436948	0.56	10	0.112148	5.714752	6.2	HMOX1	7.301587	FALSE
27.12983	0.608696	15	0.167024	6.761415	8.266667	DNMT1	9.763015	FALSE
0.333333	0.5	5	0.056487	4.044983	3.6	BACE1	4.5	FALSE
120.0444	0.736842	27	0.257026	8.172728	10.66667	HSP90AA1	21.34901	FALSE
12.14583	0.6	14	0.159801	6.58454	8.857143	FGF2	10.48306	FALSE
3.720736	0.531646	7	0.080255	4.819485	4.285714	PSEN1	5.433333	FALSE
134.1463	0.777778	30	0.281175	8.398936	11.33333	CASP3	25.8493	FALSE
6.974924	0.575342	12	0.14252	6.186193	8.166667	AURKA	9.446465	FALSE
22.6255	0.583333	12	0.134269	6.186193	6.333333	MGMT	7.221465	FALSE
25.47268	0.626866	17	0.187315	7.078313	9.529412	MMP9	12.45194	FALSE
4.667525	0.545455	9	0.101055	5.445027	5.333333	AKR1B1	6.142857	FALSE
11.54176	0.567568	10	0.121954	5.714752	6.8	LGALS3	7.638889	FALSE
196.5929	0.792453	31	0.278764	8.467292	10.58065	EGFR	25.43699	FALSE
258.5557	0.857143	35	0.307603	8.711499	11.6	TP53	32.10312	FALSE
13.32933	0.608696	15	0.171285	6.761415	9.066667	KDR	11.33349	FALSE
8.319291	0.591549	13	0.155487	6.393398	8.307692	ABCB1	9.603355	FALSE
6.170214	0.6	14	0.172948	6.58454	9.714286	HDAC1	10.89598	FALSE
7.544765	0.583333	12	0.13296	6.186193	7.5	TOP2A	8.282828	FALSE
0.2	0.518519	5	0.06918	4.044983	3.6	MAPK10	4.5	FALSE
2.005263	0.512195	6	0.06593	4.453992	3.666667	ADAM17	4.55	FALSE
5.681587	0.567568	10	0.112248	5.714752	5.4	IDH1	6.361905	FALSE
7.350313	0.525	7	0.045088	4.819485	3.142857	CDA	3.733333	FALSE
0.869048	0.506024	6	0.073253	4.453993	4	SKP1	4.8	FALSE
4.457915	0.56	9	0.112673	5.445026	5.555556	PTPN1	6.25	FALSE
15.71857	0.538462	9	0.057907	5.445026	3.333333	GART	4.603571	FALSE
1.353088	0.56	9	0.120502	5.445026	6.888889	ACTA1	7.75	FALSE
1.78279	0.538462	9	0.101545	5.445026	6.666667	VWF	7.5	FALSE
8.199098	0.512195	7	0.036774	4.819485	3.142857	PNP	3.766667	FALSE

shared nan	Subgragh
ALK	140254.2
HK2	188953.2
CCNA2	569793.6
TK1	110211.7
PSD-95	644192
TYMP	83019.47
CYP2E1	162511.6
HSPA8	338939.8
SYN	1181068
AHCY	41364
ADA	19448.13
TNF	959554
MKI67	459539.3
MAPK8	461644.4
ABL1	467685.1
HMOX1	218217.6
DNMT1	483995.9
BACE1	55365.44
HSP90AA1	1146110
FGF2	443048.8
PSEN1	111753.8
CASP3	1371597
AURKA	352413.9
MGMT	312779.6
MMP9	608732.1
AKR1B1	177183.4
LGALS3	258041
EGFR	1348170
TP53	1641539
KDR	509005.3
ABCB1	419443.5
HDAC1	518933.3
TOP2A	306725.9
MAPK10	83033.86
ADAM17	75420.83
IDH1	218596.7
CDA	35295.04
SKP1	93105.76
PTPN1	220255.5
GART	58205.56
ACTA1	251925.2
VWF	178914.9
PNP	23487.25

ONTOLOGY	ID	Description	GeneRatio	BgRatio	pvalue	p.adjust	qvalue	geneID
BP	GO:190165	glycosyl co	15/154	129/18866	1.60E-13	4.51E-10	3.12E-10	FUCA1/GB/
BP	GO:000722	Notch rece	7/154	10/18866	2.47E-13	4.51E-10	3.12E-10	ADAM17/P
BP	GO:000697	response to	23/154	458/18866	3.14E-12	3.81E-09	2.64E-09	MAPK9/CY
BP	GO:000916	nucleoside	9/154	39/18866	2.19E-11	1.99E-08	1.38E-08	ADK/ADA/I
BP	GO:000911	nucleoside	12/154	104/18866	5.15E-11	3.23E-08	2.24E-08	ADK/PNP/A
BP	GO:190165	glycosyl co	9/154	43/18866	5.66E-11	3.23E-08	2.24E-08	ADK/ADA/I
BP	GO:003129	membrane	7/154	18/18866	6.21E-11	3.23E-08	2.24E-08	ADAM17/P
BP	GO:004331	neutrophil	22/154	487/18866	7.55E-11	3.42E-08	2.37E-08	ANXA3/KPN
BP	GO:000228	neutrophil	22/154	490/18866	8.50E-11	3.42E-08	2.37E-08	ANXA3/KPN
BP	GO:000030	response to	16/154	235/18866	9.38E-11	3.42E-08	2.37E-08	MAPK9/CY
BP	GO:000673	one-carbon	8/154	40/18866	1.01E-09	3.35E-07	2.32E-07	CA14/CA7/
BP	GO:003440	nucleobase	11/154	107/18866	1.18E-09	3.59E-07	2.49E-07	ADK/PNP/A
BP	GO:000650	membrane	8/154	42/18866	1.53E-09	4.00E-07	2.77E-07	ADAM17/P
BP	GO:003361	membrane	9/154	61/18866	1.54E-09	4.00E-07	2.77E-07	ADAM17/P
BP	GO:001570	bicarbonate	8/154	43/18866	1.87E-09	4.54E-07	3.14E-07	HBB/CA14/
BP	GO:004801	ephrin rece	10/154	87/18866	2.29E-09	5.20E-07	3.60E-07	VAV2/HRAS
BP	GO:000762	circadian rh	14/154	218/18866	3.15E-09	6.76E-07	4.68E-07	HNRNPR/M
BP	GO:004428	small mole	19/154	452/18866	5.36E-09	1.08E-06	7.51E-07	CTH/GOT1/
BP	GO:006219	cellular res	17/154	360/18866	6.53E-09	1.20E-06	8.31E-07	MAPK9/HM
BP	GO:004298	amyloid pre	8/154	50/18866	6.59E-09	1.20E-06	8.31E-07	CASP3/PSE
BP	GO:000911	ribonucleos	9/154	72/18866	6.98E-09	1.21E-06	8.39E-07	ADK/PNP/A
BP	GO:005043	amyloid-be	8/154	51/18866	7.77E-09	1.29E-06	8.90E-07	CASP3/PSE
BP	GO:004309	cellular me	7/154	34/18866	9.42E-09	1.44E-06	9.94E-07	ADK/PNP/A
BP	GO:000916	purine ribo	6/154	20/18866	9.46E-09	1.44E-06	9.94E-07	ADK/ADA/I
BP	GO:007252	purine-con	19/154	472/18866	1.08E-08	1.57E-06	1.09E-06	FBP1/HSD1
BP	GO:000912	purine nucl	6/154	22/18866	1.80E-08	2.52E-06	1.74E-06	ADK/ADA/I
BP	GO:004612	purine ribo	8/154	58/18866	2.23E-08	2.84E-06	1.96E-06	ADK/PNP/A
BP	GO:001063	positive reg	12/154	176/18866	2.26E-08	2.84E-06	1.96E-06	PRKD2/AN
BP	GO:004245	purine nucl	6/154	23/18866	2.41E-08	2.84E-06	1.96E-06	ADK/ADA/I
BP	GO:004245	ribonucleos	6/154	23/18866	2.41E-08	2.84E-06	1.96E-06	ADK/ADA/I
BP	GO:004612	purine ribo	6/154	23/18866	2.41E-08	2.84E-06	1.96E-06	ADK/ADA/I
BP	GO:004851	rhythmic pr	15/154	305/18866	3.12E-08	3.55E-06	2.46E-06	HNRNPR/M
BP	GO:004227	purine nucl	8/154	61/18866	3.35E-08	3.70E-06	2.56E-06	ADK/PNP/A
BP	GO:190165	glycosyl co	7/154	42/18866	4.47E-08	4.79E-06	3.32E-06	FUCA1/GB/
BP	GO:200123	regulation	17/154	413/18866	4.91E-08	5.10E-06	3.53E-06	MAPK9/CT
BP	GO:000912	nucleoside	7/154	44/18866	6.26E-08	6.34E-06	4.39E-06	ADK/ADA/I
BP	GO:190113	carbohydra	12/154	195/18866	7.05E-08	6.94E-06	4.80E-06	NAGK/FGF
BP	GO:004298	amyloid pre	8/154	71/18866	1.13E-07	1.08E-05	7.50E-06	CASP3/PSE
BP	GO:001059	positive reg	10/154	132/18866	1.30E-07	1.22E-05	8.43E-06	PRKD2/AN
BP	GO:004317	nucleoside	5/154	16/18866	1.38E-07	1.26E-05	8.70E-06	ADK/TK1/C
BP	GO:004866	positive reg	9/154	103/18866	1.66E-07	1.48E-05	1.02E-05	HMOX1/HE
BP	GO:003109	stress-activ	14/154	300/18866	1.75E-07	1.52E-05	1.05E-05	MAPK9/FO
BP	GO:001063	regulation	14/154	301/18866	1.82E-07	1.54E-05	1.07E-05	PRKD2/AN
BP	GO:003459	cellular res	14/154	310/18866	2.61E-07	2.12E-05	1.47E-05	MAPK9/HM
BP	GO:004353	positive reg	8/154	79/18866	2.62E-07	2.12E-05	1.47E-05	PRKD2/HM
BP	GO:006032	cell chemot	14/154	311/18866	2.71E-07	2.15E-05	1.49E-05	PRKD2/HB
BP	GO:000915	ribonucleos	6/154	34/18866	2.99E-07	2.31E-05	1.60E-05	ADK/ADA/I
BP	GO:009013	tissue migr	15/154	374/18866	4.38E-07	3.33E-05	2.30E-05	ACTA1/PRK
BP	GO:000916	purine ribo	6/154	37/18866	5.06E-07	3.76E-05	2.60E-05	ADK/ADA/I

BP	GO:001059	regulation (	12/154	238/18866	6.08E-07	4.43E-05	3.07E-05	PRKD2/ANX
BP	GO:005140	stress-activ	13/154	286/18866	6.61E-07	4.72E-05	3.27E-05	MAPK9/FO
BP	GO:000616	purine nucl	16/154	442/18866	6.97E-07	4.82E-05	3.33E-05	FBP1/HSD1
BP	GO:003420	amyloid-be	6/154	39/18866	7.01E-07	4.82E-05	3.33E-05	CASP3/PSE
BP	GO:190134	regulation (	16/154	444/18866	7.40E-07	4.99E-05	3.45E-05	CTH/PRKD2
BP	GO:000912	purine nucl	6/154	40/18866	8.19E-07	5.42E-05	3.76E-05	ADK/ADA/I
BP	GO:004353	regulation (	10/154	164/18866	9.81E-07	6.38E-05	4.42E-05	PRKD2/HM
BP	GO:007252	purine-con	11/154	208/18866	1.14E-06	7.28E-05	5.04E-05	GSTZ1/ADC
BP	GO:005067	positive reg	11/154	211/18866	1.31E-06	8.24E-05	5.70E-05	PRKD2/HM
BP	GO:003461	cellular res	10/154	170/18866	1.36E-06	8.40E-05	5.82E-05	MAPK9/MN
BP	GO:200102	regulation (	5/154	25/18866	1.58E-06	9.60E-05	6.65E-05	PRKD2/FGF
BP	GO:004340	positive reg	12/154	264/18866	1.81E-06	0.000107	7.44E-05	ALK/FGF1/I
BP	GO:001063	epithelial c	14/154	365/18866	1.83E-06	0.000107	7.44E-05	PRKD2/ANX
BP	GO:000912	nucleoside	7/154	72/18866	1.99E-06	0.000115	7.94E-05	ADK/ADA/I
BP	GO:009013	epithelium	14/154	368/18866	2.01E-06	0.000115	7.94E-05	PRKD2/ANX
BP	GO:007124	cellular res	11/154	221/18866	2.06E-06	0.000115	8.00E-05	FBP1/MAPI
BP	GO:000197	G protein-c	4/154	13/18866	2.88E-06	0.000155	0.000108	ADORA2A/
BP	GO:003558	G protein-c	4/154	13/18866	2.88E-06	0.000155	0.000108	ADORA2A/
BP	GO:007188	leukocyte a	8/154	108/18866	2.90E-06	0.000155	0.000108	TP53/LGAL
BP	GO:015007	neuroinflar	7/154	77/18866	3.14E-06	0.000166	0.000115	ADORA2A/
BP	GO:200123	negative re	11/154	233/18866	3.44E-06	0.000176	0.000122	CTH/HMOX
BP	GO:004353	blood vesse	10/154	189/18866	3.54E-06	0.000176	0.000122	PRKD2/HM
BP	GO:000916	ribonucleos	6/154	51/18866	3.57E-06	0.000176	0.000122	ADK/ADA/I
BP	GO:001605	organic aci	12/154	282/18866	3.58E-06	0.000176	0.000122	CTH/GOT1/
BP	GO:004639	carboxylic a	12/154	282/18866	3.58E-06	0.000176	0.000122	CTH/GOT1/
BP	GO:190401	positive reg	11/154	235/18866	3.73E-06	0.000181	0.000125	CTH/PRKD2
BP	GO:003133	positive reg	14/154	390/18866	3.95E-06	0.000189	0.000131	HNRNPR/M
BP	GO:004354	endothelial	12/154	286/18866	4.14E-06	0.000196	0.000135	PRKD2/ANX
BP	GO:007259	reactive ox	12/154	288/18866	4.44E-06	0.000207	0.000144	FOXM1/HB
BP	GO:005067	epithelial c	15/154	453/18866	4.70E-06	0.000217	0.00015	PRKD2/HM
BP	GO:004340	regulation (	13/154	342/18866	4.79E-06	0.000218	0.000151	ALK/FGF1/I
BP	GO:005109	regulation (	15/154	455/18866	4.96E-06	0.000223	0.000154	MAPK9/CTI
BP	GO:003275	positive reg	6/154	54/18866	5.02E-06	0.000223	0.000154	PRKD2/LGA
BP	GO:007190	positive reg	13/154	345/18866	5.26E-06	0.000231	0.00016	ALK/FGF1/I
BP	GO:004310	purine-con	4/154	15/18866	5.43E-06	0.000233	0.000161	ADK/PNP/A
BP	GO:200102	positive reg	4/154	15/18866	5.43E-06	0.000233	0.000161	PRKD2/FGF
BP	GO:000916	nucleoside	5/154	32/18866	5.72E-06	0.000238	0.000165	PNP/ADA/C
BP	GO:003576	endothelial	5/154	32/18866	5.72E-06	0.000238	0.000165	PRKD2/FGF
BP	GO:004576	regulation (	14/154	403/18866	5.75E-06	0.000238	0.000165	PRKD2/GTF
BP	GO:200037	regulation (	10/154	200/18866	5.85E-06	0.000239	0.000166	FOXM1/HB
BP	GO:000697	response to	7/154	85/18866	6.10E-06	0.000247	0.000171	TP53/ABCB
BP	GO:200010	regulation (	7/154	86/18866	6.60E-06	0.000264	0.000183	TP53/LGAL
BP	GO:004650	S-adenosyl	4/154	16/18866	7.20E-06	0.000285	0.000197	MTRR/MET
BP	GO:004576	positive reg	10/154	208/18866	8.26E-06	0.000324	0.000224	PRKD2/ANX
BP	GO:000725	JNK cascad	10/154	213/18866	1.02E-05	0.000394	0.000273	MAPK9/AD
BP	GO:005072	regulation (	14/154	425/18866	1.05E-05	0.000403	0.000279	CHID1/ADC
BP	GO:001810	peptidyl-ty	13/154	374/18866	1.25E-05	0.000474	0.000328	ALK/HBEGF
BP	GO:004866	regulation (	9/154	173/18866	1.26E-05	0.000474	0.000328	HMOX1/HE
BP	GO:190129	nucleoside	11/154	269/18866	1.34E-05	0.000494	0.000342	NMRK1/GS
BP	GO:001821	peptidyl-ty	13/154	377/18866	1.36E-05	0.000494	0.000342	ALK/HBEGF

BP	GO:004668	response tc	6/154	64/18866	1.36E-05	0.000494	0.000342	MAPK9/GC
BP	GO:007127	cellular res	5/154	38/18866	1.37E-05	0.000494	0.000342	MAPK9/HM
BP	GO:004865	smooth mu	9/154	175/18866	1.38E-05	0.000494	0.000342	HMOX1/HE
BP	GO:001648	protein pro	10/154	223/18866	1.52E-05	0.000536	0.000371	SERPINE1/I
BP	GO:004483	cell cycle G	11/154	273/18866	1.54E-05	0.000539	0.000373	VPS4B/FOX
BP	GO:004873	gland deve	14/154	443/18866	1.67E-05	0.000579	0.000401	HMOX1/HK
BP	GO:000009	sulfur amin	5/154	40/18866	1.77E-05	0.000604	0.000419	CTH/TST/M
BP	GO:190367	positive reg	6/154	67/18866	1.78E-05	0.000604	0.000419	HMOX1/FG
BP	GO:003264	regulation	6/154	68/18866	1.93E-05	0.000652	0.000452	LGALS9/AD
BP	GO:004665	lymphocyte	11/154	283/18866	2.15E-05	0.000718	0.000497	TP53/LGAL
BP	GO:000989	positive reg	14/154	454/18866	2.19E-05	0.000726	0.000502	HNRNPR/M
BP	GO:006104	vascular wc	4/154	21/18866	2.29E-05	0.000752	0.000521	HPSE/SERP
BP	GO:004249	response tc	13/154	397/18866	2.34E-05	0.000761	0.000527	FBP1/CYP2
BP	GO:003294	mononucle	11/154	286/18866	2.37E-05	0.000763	0.000529	TP53/LGAL
BP	GO:005092	positive reg	8/154	144/18866	2.42E-05	0.000773	0.000535	PRKD2/LGA
BP	GO:002154	cerebellum	7/154	105/18866	2.46E-05	0.000778	0.000539	TP53/SSTR
BP	GO:000915	purine ribo	13/154	401/18866	2.60E-05	0.000813	0.000563	FBP1/HSD1
BP	GO:200037	positive reg	7/154	106/18866	2.61E-05	0.000813	0.000563	HBB/TP53/
BP	GO:009719	intrinsic ap	11/154	290/18866	2.69E-05	0.000831	0.000575	HMOX1/TP
BP	GO:004245	ribonucleos	4/154	22/18866	2.79E-05	0.000846	0.000586	PNP/ADA/C
BP	GO:004854	response tc	12/154	346/18866	2.79E-05	0.000846	0.000586	ACTA1/GOT
BP	GO:001051	regulation	6/154	73/18866	2.91E-05	0.000876	0.000607	FGF2/HRAS
BP	GO:003272	positive reg	5/154	45/18866	3.19E-05	0.000952	0.000659	LGALS9/AD
BP	GO:000721	Notch signa	9/154	195/18866	3.26E-05	0.000964	0.000667	GOT1/ADA
BP	GO:003300	muscle cell	10/154	244/18866	3.28E-05	0.000964	0.000667	HMOX1/HE
BP	GO:003110	animal orga	6/154	75/18866	3.40E-05	0.000974	0.000675	ANXA3/HM
BP	GO:003260	chemokine	6/154	75/18866	3.40E-05	0.000974	0.000675	LGALS9/AD
BP	GO:005140	neuron apc	10/154	245/18866	3.40E-05	0.000974	0.000675	HMOX1/TP
BP	GO:000925	ribonucleot	13/154	416/18866	3.80E-05	0.00108	0.000748	FBP1/HSD1
BP	GO:000193	endothelial	9/154	199/18866	3.83E-05	0.00108	0.000748	PRKD2/HM
BP	GO:000193	positive reg	7/154	113/18866	3.95E-05	0.001094	0.000758	PRKD2/HM
BP	GO:003057	collagen ca	5/154	47/18866	3.95E-05	0.001094	0.000758	MMP13/M
BP	GO:005105	positive reg	9/154	200/18866	3.98E-05	0.001094	0.000758	FOXM1/PR
BP	GO:005188	mitochond	4/154	24/18866	3.99E-05	0.001094	0.000758	GOT1/ABL1
BP	GO:007099	neuron dea	12/154	360/18866	4.10E-05	0.001112	0.00077	HMOX1/TP
BP	GO:000166	ameboidal-	14/154	481/18866	4.12E-05	0.001112	0.00077	PRKD2/AN)
BP	GO:002203	metenceph	7/154	114/18866	4.18E-05	0.001118	0.000774	TP53/SSTR
BP	GO:000018	activation c	8/154	156/18866	4.29E-05	0.001134	0.000786	ALK/FGF1/I
BP	GO:190160	alpha-amin	9/154	202/18866	4.30E-05	0.001134	0.000786	CTH/GOT1/
BP	GO:190027	regulation	5/154	48/18866	4.38E-05	0.001148	0.000795	FGF2/HRAS
BP	GO:001931	hexose met	10/154	254/18866	4.61E-05	0.001199	0.00083	FBP1/GOT1
BP	GO:005072	positive reg	8/154	158/18866	4.70E-05	0.001214	0.000841	ADORA2B/
BP	GO:001003	response tc	12/154	366/18866	4.81E-05	0.001233	0.000854	ACTA1/FBP
BP	GO:003465	nucleobase	5/154	49/18866	4.85E-05	0.001234	0.000855	PNP/ADA/C
BP	GO:190122	positive reg	6/154	80/18866	4.90E-05	0.001235	0.000855	LGALS9/MI
BP	GO:005109	positive reg	8/154	159/18866	4.92E-05	0.001235	0.000855	CTH/ALK/P
BP	GO:001969	ribose phos	13/154	427/18866	4.96E-05	0.001238	0.000857	FBP1/HSD1
BP	GO:005085	T cell recep	9/154	206/18866	5.01E-05	0.00124	0.000859	PRKD2/SKP
BP	GO:007066	leukocyte p	11/154	313/18866	5.39E-05	0.001325	0.000918	TP53/LGAL
BP	GO:004275	regulation	7/154	122/18866	6.44E-05	0.001572	0.001088	MAPK9/TO

BP	GO:004814	astrocyte a	4/154	27/18866	6.47E-05	0.001572	0.001088	ADORA2A/
BP	GO:004352	regulation (	9/154	214/18866	6.72E-05	0.001615	0.001118	HMOX1/TP
BP	GO:190121	regulation (	11/154	321/18866	6.75E-05	0.001615	0.001118	HMOX1/TP
BP	GO:000916	nucleotide	10/154	266/18866	6.79E-05	0.001615	0.001118	NMRK1/GS
BP	GO:000906	cellular am	7/154	124/18866	7.14E-05	0.001688	0.001169	CTH/GOT1/
BP	GO:000177	leukocyte h	6/154	86/18866	7.37E-05	0.001731	0.001199	LGALS9/AD
BP	GO:005109	positive reg	10/154	270/18866	7.68E-05	0.001793	0.001242	CTH/ALK/P
BP	GO:000181	positive reg	13/154	447/18866	7.89E-05	0.00183	0.001267	PRKD2/LGA
BP	GO:190547	positive reg	7/154	127/18866	8.30E-05	0.001886	0.001306	TP53/LGAL
BP	GO:000865	pyrimidine-	3/154	11/18866	8.39E-05	0.001886	0.001306	TK1/CDA/T
BP	GO:003576	endothelial	3/154	11/18866	8.39E-05	0.001886	0.001306	FGF1/FGF2
BP	GO:004309	pyrimidine	3/154	11/18866	8.39E-05	0.001886	0.001306	TK1/CDA/T
BP	GO:200054	regulation (	3/154	11/18866	8.39E-05	0.001886	0.001306	FGF1/FGF2
BP	GO:003214	activation c	11/154	331/18866	8.88E-05	0.001966	0.001361	ALK/FGF1/I
BP	GO:006056	epithelial tu	11/154	331/18866	8.88E-05	0.001966	0.001361	PRKD2/AR/
BP	GO:002154	pallium dev	8/154	173/18866	8.90E-05	0.001966	0.001361	ALK/ANXA3
BP	GO:006056	neuroepith	5/154	56/18866	9.27E-05	0.00201	0.001392	USH1C/MY
BP	GO:007022	regulation (	5/154	56/18866	9.27E-05	0.00201	0.001392	TP53/LGAL
BP	GO:190464	response tc	5/154	56/18866	9.27E-05	0.00201	0.001392	MMP13/M
BP	GO:004349	protein kin	10/154	278/18866	9.78E-05	0.002108	0.001459	HBEGF/HSF
BP	GO:006005	angiogenes	4/154	30/18866	9.92E-05	0.002117	0.001466	HPSE/SERP
BP	GO:005067	regulation (	12/154	395/18866	9.94E-05	0.002117	0.001466	PRKD2/HM
BP	GO:005189	positive reg	8/154	178/18866	0.000109	0.002299	0.001592	HBEGF/HSF
BP	GO:004277	signal trans	7/154	133/18866	0.000111	0.002301	0.001593	FOXM1/MY
BP	GO:000600	fructose 6-|	3/154	12/18866	0.000111	0.002301	0.001593	FBP1/HK2/
BP	GO:003576	cell chemot	3/154	12/18866	0.000111	0.002301	0.001593	FGF1/FGF2
BP	GO:190484	regulation (	3/154	12/18866	0.000111	0.002301	0.001593	FGF1/FGF2
BP	GO:000652	cellular am	11/154	340/18866	0.000112	0.002307	0.001598	CTH/GOT1/
BP	GO:005092	regulation (	9/154	229/18866	0.000113	0.002307	0.001598	PRKD2/LGA
BP	GO:003267	regulation (	6/154	93/18866	0.000114	0.002319	0.001606	PRKD2/LGA
BP	GO:004593	negative re	11/154	341/18866	0.000115	0.002336	0.001618	TOP2A/ND
BP	GO:004352	positive reg	5/154	59/18866	0.000119	0.002398	0.001661	TP53/CASP
BP	GO:006190	glial cell act	5/154	60/18866	0.000129	0.002571	0.00178	ADORA2A/
BP	GO:009775	positive reg	5/154	60/18866	0.000129	0.002571	0.00178	HMOX1/AD
BP	GO:000726	Ras protein	11/154	346/18866	0.000131	0.002576	0.001784	FBP1/FOXN
BP	GO:190303	regulation (	8/154	183/18866	0.000132	0.002576	0.001784	HBEGF/PTF
BP	GO:200123	positive reg	8/154	183/18866	0.000132	0.002576	0.001784	MAPK9/TP5
BP	GO:000193	regulation (	8/154	184/18866	0.000137	0.002646	0.001832	PRKD2/HM
BP	GO:003806	NIK/NF-kap	8/154	184/18866	0.000137	0.002646	0.001832	ALK/SKP1/I
BP	GO:190390	positive reg	5/154	61/18866	0.00014	0.002693	0.001865	VPS4B/TOF
BP	GO:190121	positive reg	6/154	97/18866	0.000144	0.002757	0.001909	TP53/ABL1,
BP	GO:003559	purinergic r	4/154	33/18866	0.000145	0.002771	0.001919	ADORA2A/
BP	GO:005160	protein ma	10/154	293/18866	0.00015	0.00285	0.001974	SERPINE1/I
BP	GO:001051	positive reg	5/154	62/18866	0.000151	0.00285	0.001974	FGF2/HRAS
BP	GO:006113	morphoger	8/154	187/18866	0.000153	0.002866	0.001985	AR/FGF1/F
BP	GO:000599	monosacch	10/154	296/18866	0.000163	0.003041	0.002106	FBP1/GOT1
BP	GO:007023	regulation (	4/154	34/18866	0.000164	0.003041	0.002106	TP53/LGAL
BP	GO:003809	Fc receptor	9/154	241/18866	0.000166	0.003064	0.002122	MAPK9/SKI
BP	GO:000301	vascular pr	8/154	190/18866	0.00017	0.003132	0.002169	HMOX1/AE
BP	GO:003529	regulation (	7/154	143/18866	0.000174	0.003173	0.002197	HMOX1/AD

BP	GO:009774	regulation (	7/154	143/18866	0.000174	0.003173	0.002197	HMOX1/AD
BP	GO:003263	interleukin-	6/154	101/18866	0.00018	0.0032	0.002216	PRKD2/LGA
BP	GO:006019	regulation (	6/154	101/18866	0.00018	0.0032	0.002216	FGF2/HRAS
BP	GO:000166	response to	11/154	359/18866	0.000181	0.0032	0.002216	HMOX1/MI
BP	GO:004265	regulation (	3/154	14/18866	0.000182	0.0032	0.002216	AR/FGF2/F(
BP	GO:005066	homocyste	3/154	14/18866	0.000182	0.0032	0.002216	CTH/TST/M
BP	GO:009039	replicative	3/154	14/18866	0.000182	0.0032	0.002216	TP53/SERPI
BP	GO:003515	regulation (	7/154	144/18866	0.000182	0.0032	0.002216	HMOX1/AD
BP	GO:000911	nucleobase	4/154	35/18866	0.000184	0.003214	0.002226	ADA/CDA/T
BP	GO:003272	positive reg	5/154	65/18866	0.000189	0.003277	0.002269	LGALS9/HR
BP	GO:007124	cellular res	8/154	193/18866	0.00019	0.003277	0.002269	FBP1/MAPI
BP	GO:003812	ERBB signa	7/154	145/18866	0.00019	0.003277	0.002269	HBEGF/HSF
BP	GO:007121	cellular res	9/154	246/18866	0.000193	0.003319	0.002299	TP53/SERPI
BP	GO:000863	intrinsic ap	6/154	103/18866	0.0002	0.003419	0.002368	HMOX1/TP
BP	GO:190160	alpha-amin	5/154	66/18866	0.000203	0.003445	0.002386	CTH/GOT1/
BP	GO:190547	regulation (	8/154	195/18866	0.000203	0.003445	0.002386	TP53/AR/L(
BP	GO:005138	response to	7/154	147/18866	0.000207	0.003483	0.002412	GOT1/ANX
BP	GO:005105	regulation (	11/154	365/18866	0.000209	0.003503	0.002426	FOXM1/PR
BP	GO:007037	regulation (	10/154	306/18866	0.000214	0.003567	0.00247	PRKD2/LGA
BP	GO:000616	purine nucl	8/154	197/18866	0.000218	0.003625	0.00251	GSTZ1/ADC
BP	GO:000009	sulfur amin	3/154	15/18866	0.000226	0.003738	0.002589	CTH/TST/M
BP	GO:009005	positive reg	4/154	37/18866	0.000229	0.003768	0.002609	HMOX1/FG
BP	GO:001810	peptidyl-se	10/154	310/18866	0.000237	0.003887	0.002692	MAPK9/PR
BP	GO:005189	regulation (	9/154	253/18866	0.000238	0.003887	0.002692	HBEGF/HSF
BP	GO:003629	response to	11/154	371/18866	0.00024	0.003904	0.002703	HMOX1/MI
BP	GO:006104	regulation (	7/154	151/18866	0.000244	0.003942	0.00273	HBEGF/FGF
BP	GO:000008	G2/M trans	9/154	254/18866	0.000245	0.003948	0.002734	VPS4B/FOX
BP	GO:200123	negative re	6/154	107/18866	0.000246	0.003948	0.002734	HMOX1/AF
BP	GO:000176	morphoger	8/154	201/18866	0.00025	0.003991	0.002764	AR/FGF1/F(
BP	GO:007139	cellular res	4/154	38/18866	0.000254	0.004038	0.002797	ESR2/SSTR
BP	GO:004887	homeostas	9/154	256/18866	0.00026	0.004112	0.002847	HMOX1/MI
BP	GO:190161	organic hyc	9/154	258/18866	0.000275	0.004318	0.002991	GOT1/HSD
BP	GO:004613	pyrimidine	3/154	16/18866	0.000276	0.004318	0.002991	TK1/CDA/T
BP	GO:007173	cellular res	3/154	16/18866	0.000276	0.004318	0.002991	MMP3/CDK
BP	GO:004875	branching r	7/154	155/18866	0.000286	0.004446	0.003079	AR/FGF1/F(
BP	GO:003090	hindbrain d	7/154	156/18866	0.000297	0.004593	0.003181	TP53/SSTR
BP	GO:000756	aging	10/154	319/18866	0.000298	0.004593	0.003181	FOXM1/TP
BP	GO:001657	histone pho	4/154	40/18866	0.00031	0.00475	0.00329	CHEK1/AUI
BP	GO:004574	positive reg	4/154	40/18866	0.00031	0.00475	0.00329	HRAS/FGFF
BP	GO:000627	regulation (	6/154	112/18866	0.000315	0.004801	0.003325	TP53/HRAS
BP	GO:007022	lymphocyte	5/154	73/18866	0.000326	0.004951	0.003429	TP53/LGAL
BP	GO:000989	negative re	10/154	323/18866	0.000329	0.004954	0.00343	FBP1/HMO
BP	GO:003134	positive reg	11/154	385/18866	0.000329	0.004954	0.00343	SKP1/ADOI
BP	GO:003296	collagen m	6/154	113/18866	0.000331	0.004954	0.00343	GOT1/MMI
BP	GO:000009	sulfur amin	3/154	17/18866	0.000333	0.004978	0.003447	CTH/MTRR
BP	GO:005085	antigen rec	10/154	325/18866	0.000345	0.005107	0.003537	PRKD2/SKP
BP	GO:007037	ERK1 and E	10/154	325/18866	0.000345	0.005107	0.003537	PRKD2/LGA
BP	GO:005188	regulation (	5/154	74/18866	0.000348	0.005126	0.00355	GOT1/ADO
BP	GO:000268	regulation (	8/154	212/18866	0.000358	0.005251	0.003636	HMOX1/LG
BP	GO:200123	regulation (	7/154	162/18866	0.000373	0.005462	0.003782	HMOX1/AF

BP	GO:003090	forebrain d	11/154	391/18866	0.000375	0.005466	0.003785	ALK/ANXA3
BP	GO:007037	positive reg	8/154	215/18866	0.000393	0.005687	0.003938	PRKD2/LGA
BP	GO:006019	positive reg	5/154	76/18866	0.000394	0.005687	0.003938	FGF2/HRAS
BP	GO:007121	cellular res	10/154	331/18866	0.000398	0.005712	0.003956	GOT1/TP53
BP	GO:010400	cellular res	10/154	331/18866	0.000398	0.005712	0.003956	GOT1/TP53
BP	GO:003196	response to	7/154	164/18866	0.000402	0.005721	0.003962	GOT1/ANX
BP	GO:004616	alcohol bio	7/154	164/18866	0.000402	0.005721	0.003962	GOT1/KPN
BP	GO:003019	extracellula	11/154	395/18866	0.000409	0.005792	0.004011	VWF/FGF2,
BP	GO:015007	regulation	4/154	43/18866	0.000411	0.005806	0.00402	MMP8/MN
BP	GO:000756	cell aging	6/154	118/18866	0.000417	0.005806	0.00402	FOXM1/TP
BP	GO:004306	extracellula	11/154	396/18866	0.000418	0.005806	0.00402	VWF/FGF2,
BP	GO:007048	response to	11/154	396/18866	0.000418	0.005806	0.00402	HMOX1/M
BP	GO:001820	peptidyl-se	10/154	333/18866	0.000418	0.005806	0.00402	MAPK9/PR
BP	GO:003249	response to	10/154	334/18866	0.000428	0.005921	0.0041	LGALS9/SE
BP	GO:001082	positive reg	6/154	119/18866	0.000436	0.005994	0.004151	HK2/TP53/
BP	GO:190122	regulation	6/154	119/18866	0.000436	0.005994	0.004151	LGALS9/M
BP	GO:004593	positive reg	7/154	168/18866	0.000465	0.006326	0.004381	VPS4B/ND
BP	GO:000315	morphoger	3/154	19/18866	0.00047	0.006326	0.004381	PRKD2/FGF
BP	GO:006115	endothelial	3/154	19/18866	0.00047	0.006326	0.004381	PRKD2/FGF
BP	GO:190217	cellular res	3/154	19/18866	0.00047	0.006326	0.004381	MMP3/CD
BP	GO:001657	histone mo	12/154	468/18866	0.00047	0.006326	0.004381	MORF4L1/
BP	GO:001657	histone dea	5/154	79/18866	0.000471	0.006326	0.004381	MORF4L1/
BP	GO:003809	Fc-epsilon	7/154	169/18866	0.000482	0.006448	0.004465	MAPK9/SK
BP	GO:001400	astrocyte d	4/154	45/18866	0.00049	0.006518	0.004513	ADORA2A/
BP	GO:004542	positive reg	4/154	45/18866	0.00049	0.006518	0.004513	HBB/HSP90
BP	GO:000269	positive reg	8/154	223/18866	0.0005	0.006628	0.00459	HMOX1/CD
BP	GO:000717	epidermal	6/154	123/18866	0.00052	0.006837	0.004735	HBEGF/MM
BP	GO:190437	regulation	6/154	123/18866	0.00052	0.006837	0.004735	AR/LGALS3
BP	GO:001086	positive reg	4/154	46/18866	0.000534	0.006966	0.004824	FGF2/HRAS
BP	GO:190440	positive reg	4/154	46/18866	0.000534	0.006966	0.004824	HBB/HSP90
BP	GO:002261	gland morp	6/154	124/18866	0.000543	0.007042	0.004876	AR/FGF1/E
BP	GO:200102	regulation	8/154	226/18866	0.000547	0.007042	0.004876	FOXM1/GT
BP	GO:000254	chronic infl	3/154	20/18866	0.000549	0.007042	0.004876	ADORA2B/
BP	GO:007173	response to	3/154	20/18866	0.000549	0.007042	0.004876	MMP3/CD
BP	GO:190040	positive reg	3/154	20/18866	0.000549	0.007042	0.004876	MMP3/MC
BP	GO:002261	extracellula	5/154	82/18866	0.000559	0.007116	0.004928	MMP13/M
BP	GO:190331	positive reg	5/154	82/18866	0.000559	0.007116	0.004928	HNRNPR/P
BP	GO:001050	regulation	10/154	347/18866	0.000575	0.007303	0.005057	HMOX1/HK
BP	GO:004330	mast cell d	4/154	47/18866	0.00058	0.007326	0.005073	HMOX1/LG
BP	GO:000724	I-kappaB ki	9/154	286/18866	0.000581	0.007326	0.005073	CTH/HMOX
BP	GO:000759	hemostasis	10/154	348/18866	0.000588	0.007389	0.005117	FLI1/F13A1
BP	GO:004870	astrocyte d	5/154	83/18866	0.000591	0.007389	0.005117	ADORA2A/
BP	GO:000683	neurotrans	8/154	229/18866	0.000596	0.007389	0.005117	SLC6A2/AD
BP	GO:000242	immune re	12/154	481/18866	0.000599	0.007389	0.005117	PRKD2/SKP
BP	GO:000275	immune re	12/154	481/18866	0.000599	0.007389	0.005117	PRKD2/SKP
BP	GO:001656	covalent ch	12/154	481/18866	0.000599	0.007389	0.005117	MORF4L1/
BP	GO:005081	coagulation	10/154	349/18866	0.000602	0.007402	0.005126	F13A1/VW
BP	GO:001624	regulation	7/154	176/18866	0.000613	0.007496	0.005191	HMOX1/TP
BP	GO:009719	extrinsic ap	8/154	230/18866	0.000613	0.007496	0.005191	HMOX1/AR
BP	GO:000865	cellular am	5/154	84/18866	0.000624	0.007602	0.005264	CTH/GOT1/

BP	GO:000227	mast cell a	4/154	48/18866	0.000628	0.007603	0.005265	HMOX1/LG
BP	GO:190403	negative re	4/154	48/18866	0.000628	0.007603	0.005265	HMOX1/SE
BP	GO:000906	aspartate f	3/154	21/18866	0.000637	0.007654	0.005301	GOT1/MTR
BP	GO:001613	glycoside n	3/154	21/18866	0.000637	0.007654	0.005301	FUCA1/GBA
BP	GO:000244	mast cell m	4/154	49/18866	0.00068	0.008092	0.005604	HMOX1/LG
BP	GO:004851	circadian b	4/154	49/18866	0.00068	0.008092	0.005604	TP53/ADOI
BP	GO:007023	T cell apop	4/154	49/18866	0.00068	0.008092	0.005604	TP53/LGAL
BP	GO:000223	response tc	10/154	356/18866	0.000701	0.008315	0.005758	LGALS9/SEI
BP	GO:004426	cellular car	9/154	294/18866	0.000708	0.008369	0.005796	FBP1/GOT1
BP	GO:190470	regulation	5/154	87/18866	0.000732	0.00849	0.00588	HMOX1/FG
BP	GO:199087	vascular as	5/154	87/18866	0.000732	0.00849	0.00588	HMOX1/FG
BP	GO:005190	regulation	3/154	22/18866	0.000733	0.00849	0.00588	GOT1/KDR/
BP	GO:190288	positive reg	3/154	22/18866	0.000733	0.00849	0.00588	MMP3/MC
BP	GO:000762	rhythmic b	4/154	50/18866	0.000734	0.00849	0.00588	TP53/ADOI
BP	GO:001967	NAD metab	4/154	50/18866	0.000734	0.00849	0.00588	NMRK1/HK
BP	GO:190470	positive reg	4/154	50/18866	0.000734	0.00849	0.00588	FGF2/MMF
BP	GO:004677	protein aut	8/154	237/18866	0.000746	0.008597	0.005954	ALK/PRKD2
BP	GO:000110	response tc	6/154	132/18866	0.000755	0.00867	0.006004	AKR1B1/EG
BP	GO:000181	negative re	10/154	360/18866	0.000764	0.008744	0.006055	CHID1/HM
BP	GO:003275	positive reg	5/154	88/18866	0.000772	0.008809	0.0061	LGALS9/AD
BP	GO:007252	purine-con	4/154	51/18866	0.000792	0.009012	0.006241	PNP/ADA/F
BP	GO:190379	regulation	3/154	23/18866	0.000838	0.009506	0.006583	TP53/EGFR
BP	GO:000315	endotheliu	6/154	135/18866	0.000849	0.009599	0.006647	PRKD2/FGF
BP	GO:004205	regulation	5/154	90/18866	0.000854	0.009633	0.006671	HBEGF/MM
BP	GO:004639	carboxylic a	10/154	367/18866	0.000884	0.009938	0.006882	CTH/GOT1/
BP	GO:005189	membrane	5/154	91/18866	0.000898	0.010065	0.00697	GOT1/ADO
BP	GO:001605	organic aci	10/154	368/18866	0.000903	0.010083	0.006982	CTH/GOT1/
BP	GO:000175	organ indu	3/154	24/18866	0.000952	0.010569	0.007319	AR/FGF1/F
BP	GO:004683	carbohydra	3/154	24/18866	0.000952	0.010569	0.007319	NAGK/HK2/
BP	GO:000679	sulfur comp	10/154	371/18866	0.00096	0.010618	0.007353	CTH/HSD17
BP	GO:000657	cellular mo	7/154	190/18866	0.000962	0.010618	0.007353	IDH1/MTRI
BP	GO:003262	interleukin	4/154	54/18866	0.000983	0.010819	0.007492	PRKD2/PNI
BP	GO:000253	production	5/154	93/18866	0.000991	0.010869	0.007527	CHID1/SER
BP	GO:001623	macroauto	9/154	310/18866	0.001028	0.011212	0.007764	VPS4B/HM
BP	GO:009028	regulation	9/154	310/18866	0.001028	0.011212	0.007764	GOT1/PRKI
BP	GO:190403	regulation	5/154	94/18866	0.00104	0.011302	0.007827	HMOX1/SE
BP	GO:000188	liver develc	6/154	141/18866	0.001064	0.01153	0.007985	HMOX1/FG
BP	GO:007092	regulation	3/154	25/18866	0.001075	0.011551	0.007999	TP53/EGFR
BP	GO:007167	positive reg	3/154	25/18866	0.001075	0.011551	0.007999	LGALS3/SEI
BP	GO:190556	positive reg	3/154	25/18866	0.001075	0.011551	0.007999	FGF2/ADAI
BP	GO:000268	positive reg	6/154	142/18866	0.001103	0.011818	0.008184	LGALS3/LG
BP	GO:004209	T cell prolif	7/154	195/18866	0.001119	0.011951	0.008276	TP53/LGAL
BP	GO:004427	sulfur comp	4/154	56/18866	0.001128	0.011973	0.008292	CTH/TST/N
BP	GO:006068	regulation	4/154	56/18866	0.001128	0.011973	0.008292	AR/ABL1/F
BP	GO:000204	sprouting a	7/154	196/18866	0.001153	0.012203	0.00845	HMOX1/FG
BP	GO:004325	regulation	11/154	449/18866	0.00117	0.012354	0.008555	MAPK9/WA
BP	GO:006100	hepaticobil	6/154	144/18866	0.001186	0.012482	0.008644	HMOX1/FG
BP	GO:000647	protein dea	5/154	97/18866	0.001197	0.012527	0.008675	MORF4L1/I
BP	GO:190118	regulation	5/154	97/18866	0.001197	0.012527	0.008675	HBEGF/MM
BP	GO:004274	hydrogen p	4/154	57/18866	0.001205	0.012537	0.008682	HBB/PRDX6

BP	GO:003274	positive reg	3/154	26/18866	0.001208	0.012537	0.008682	PRKD2/PDE
BP	GO:005115	glucose 6-p	3/154	26/18866	0.001208	0.012537	0.008682	HK2/TP53/
BP	GO:004856	embryonic	11/154	451/18866	0.001213	0.012546	0.008688	USH1C/SAT
BP	GO:004801	vascular en	5/154	98/18866	0.001253	0.012924	0.00895	PRKD2/VAV
BP	GO:003133	positive reg	8/154	257/18866	0.001256	0.012924	0.00895	MAPK9/WA
BP	GO:004254	response to	6/154	146/18866	0.001273	0.01306	0.009044	HMOX1/HE
BP	GO:000204	blood vesse	4/154	58/18866	0.001286	0.013122	0.009087	HMOX1/FG
BP	GO:000288	regulation	4/154	58/18866	0.001286	0.013122	0.009087	HMOX1/LG
BP	GO:001605	carbohydra	7/154	200/18866	0.001295	0.013176	0.009125	FBP1/HK2/
BP	GO:005083	defense res	5/154	99/18866	0.001311	0.013302	0.009212	LYZ/ADAM
BP	GO:002153	telencepha	8/154	259/18866	0.00132	0.01335	0.009245	ALK/ANXA3
BP	GO:003109	regeneratio	7/154	201/18866	0.001333	0.013447	0.009312	ANXA3/HM
BP	GO:000965	response to	3/154	27/18866	0.001351	0.013594	0.009414	TP53/AKR1
BP	GO:190367	regulation	6/154	148/18866	0.001365	0.013695	0.009484	HMOX1/FG
BP	GO:005073	regulation	8/154	263/18866	0.001454	0.014527	0.01006	HBEGF/TP5
BP	GO:007149	cellular res	9/154	326/18866	0.001458	0.014527	0.01006	GOT1/HMO
BP	GO:190342	positive reg	4/154	60/18866	0.00146	0.014527	0.01006	HBB/HSP90
BP	GO:000007	regulation	5/154	102/18866	0.001497	0.014807	0.010254	ADAM17/E
BP	GO:190307	regulation	5/154	102/18866	0.001497	0.014807	0.010254	AR/LGALS3
BP	GO:000676	folic acid-c	3/154	28/18866	0.001504	0.014807	0.010254	MTRR/ATIC
BP	GO:004274	circadian sl	3/154	28/18866	0.001504	0.014807	0.010254	ADORA2A/
BP	GO:004557	mast cell ac	4/154	61/18866	0.001552	0.015159	0.010497	HMOX1/LG
BP	GO:009030	positive reg	4/154	61/18866	0.001552	0.015159	0.010497	HBEGF/HPS
BP	GO:190307	positive reg	4/154	61/18866	0.001552	0.015159	0.010497	LGALS3/EP
BP	GO:004578	positive reg	10/154	396/18866	0.001562	0.015215	0.010537	VPS4B/ND
BP	GO:005086	negative re	7/154	207/18866	0.001578	0.015322	0.010611	HMOX1/LG
BP	GO:007122	cellular res	7/154	208/18866	0.001622	0.015707	0.010877	SERPINE1/
BP	GO:003264	regulation	5/154	104/18866	0.001632	0.015724	0.010889	LGALS9/HR
BP	GO:004327	response to	5/154	104/18866	0.001632	0.015724	0.010889	UQCRC1/A
BP	GO:000226	lymphocyte	4/154	62/18866	0.001649	0.015731	0.010894	LGALS9/AB
BP	GO:200035	regulation	4/154	62/18866	0.001649	0.015731	0.010894	SERPINE1/
BP	GO:000820	steroid met	9/154	332/18866	0.001652	0.015731	0.010894	HSD17B4/C
BP	GO:000961	response to	7/154	209/18866	0.001667	0.015731	0.010894	ACTA1/STR
BP	GO:000178	B cell home	3/154	29/18866	0.001668	0.015731	0.010894	ABL1/CASP
BP	GO:003106	regulation	3/154	29/18866	0.001668	0.015731	0.010894	PRKD2/TP5
BP	GO:005082	positive reg	3/154	29/18866	0.001668	0.015731	0.010894	HPSE/SERP
BP	GO:200010	positive reg	3/154	29/18866	0.001668	0.015731	0.010894	TP53/LGAL
BP	GO:000226	myeloid cel	6/154	154/18866	0.001672	0.015731	0.010894	HMOX1/M
BP	GO:000282	positive reg	5/154	105/18866	0.001703	0.01594	0.011038	CD1A/BTK/
BP	GO:004407	regulation	5/154	105/18866	0.001703	0.01594	0.011038	ABCB1/AD
BP	GO:000270	regulation	7/154	211/18866	0.001759	0.016429	0.011377	HMOX1/CD
BP	GO:190402	regulation	5/154	106/18866	0.001775	0.016536	0.011451	ADAM17/E
BP	GO:000626	DNA replica	8/154	273/18866	0.001838	0.016931	0.011725	TP53/HRAS
BP	GO:000717	regulation	3/154	30/18866	0.001842	0.016931	0.011725	HBEGF/AD
BP	GO:190390	regulation	6/154	157/18866	0.001843	0.016931	0.011725	VPS4B/TOP
BP	GO:003033	DNA damag	5/154	107/18866	0.00185	0.016931	0.011725	FOXM1/MY
BP	GO:003560	protein dea	5/154	107/18866	0.00185	0.016931	0.011725	MORF4L1/
BP	GO:004852	positive reg	5/154	107/18866	0.00185	0.016931	0.011725	VPS4B/TOP
BP	GO:005090	neuromusc	5/154	107/18866	0.00185	0.016931	0.011725	USH1C/AD
BP	GO:004617	polyol bios	4/154	64/18866	0.001855	0.016931	0.011725	GOT1/FGF

BP	GO:190382	positive reg	9/154	338/18866	0.001866	0.01699	0.011765	NDC80/TP5
BP	GO:00060C	glucose me	7/154	214/18866	0.001906	0.01731	0.011988	FBP1/GOT1
BP	GO:000758	excretion	4/154	65/18866	0.001964	0.017713	0.012267	HMOX1/AD
BP	GO:00512C	protein ins	4/154	65/18866	0.001964	0.017713	0.012267	TP53/HSP9
BP	GO:003267	regulation	6/154	159/18866	0.001965	0.017713	0.012267	LGALS9/AD
BP	GO:001905	viral life cy	9/154	341/18866	0.001981	0.017816	0.012338	VPS4B/TOF
BP	GO:002176	limbic syste	5/154	109/18866	0.002007	0.017976	0.012448	ALK/ANXA3
BP	GO:005165	maintenan	7/154	216/18866	0.002009	0.017976	0.012448	HK2/SKP1/
BP	GO:004594	positive reg	3/154	31/18866	0.002027	0.018098	0.012533	FGF1/AGTF
BP	GO:004516	cell fate co	8/154	278/18866	0.002058	0.018183	0.012592	SATB2/TP5
BP	GO:000759	blood coag	9/154	343/18866	0.002061	0.018183	0.012592	F13A1/VW
BP	GO:001604	lipid catabo	9/154	343/18866	0.002061	0.018183	0.012592	HSD17B4/I
BP	GO:005079	regulation	7/154	217/18866	0.002062	0.018183	0.012592	VPS4B/TOF
BP	GO:190274	regulation	7/154	217/18866	0.002062	0.018183	0.012592	VPS4B/SKP
BP	GO:004211	T cell activa	11/154	483/18866	0.002082	0.018315	0.012684	TP53/LGAL
BP	GO:000282	positive reg	5/154	110/18866	0.002089	0.018333	0.012696	CD1A/BTK/
BP	GO:004232	negative re	11/154	484/18866	0.002116	0.018523	0.012827	FBP1/FOXN
BP	GO:000007	cell cycle cl	7/154	219/18866	0.002171	0.018936	0.013114	TOP2A/ND
BP	GO:009873	macromole	5/154	111/18866	0.002173	0.018936	0.013114	MORF4L1/I
BP	GO:003812	ERBB2 sign	3/154	32/18866	0.002224	0.019239	0.013323	HBEGF/HSF
BP	GO:00433C	regulation	3/154	32/18866	0.002224	0.019239	0.013323	HMOX1/LG
BP	GO:009742	liver regene	3/154	32/18866	0.002224	0.019239	0.013323	HMOX1/AU
BP	GO:000206	epithelial c	7/154	221/18866	0.002285	0.01972	0.013656	HSD17B4/A
BP	GO:004542	regulation	4/154	68/18866	0.002318	0.019867	0.013758	HBB/HSP90
BP	GO:007257	endothelial	4/154	68/18866	0.002318	0.019867	0.013758	SERPINE1/A
BP	GO:190437	positive reg	4/154	68/18866	0.002318	0.019867	0.013758	LGALS3/EPI
BP	GO:001604	cell growth	11/154	490/18866	0.002327	0.019898	0.01378	FBP1/HBEG
BP	GO:001972	calcium-me	7/154	222/18866	0.002344	0.019942	0.01381	KDR/BTK/E
BP	GO:007121	cellular res	7/154	222/18866	0.002344	0.019942	0.01381	SERPINE1/A
BP	GO:00330C	regulation	3/154	33/18866	0.002432	0.020596	0.014263	HMOX1/LG
BP	GO:004592	negative re	3/154	33/18866	0.002432	0.020596	0.014263	VPS4B/HM
BP	GO:000709	mitotic cell	6/154	166/18866	0.002439	0.02061	0.014273	TOP2A/ND
BP	GO:00326C	interferon-	5/154	115/18866	0.002536	0.021379	0.014805	LGALS9/HR
BP	GO:005122	positive reg	9/154	354/18866	0.002548	0.021431	0.014841	TP53/ADOF
BP	GO:003043	sleep	3/154	34/18866	0.002651	0.022146	0.015337	ADORA2A/
BP	GO:004255	pteridine-c	3/154	34/18866	0.002651	0.022146	0.015337	MTRR/ATIC
BP	GO:200035	negative re	3/154	34/18866	0.002651	0.022146	0.015337	SERPINE1/A
BP	GO:003362	cell adhesi	4/154	71/18866	0.002714	0.02257	0.01563	SERPINE1/A
BP	GO:004573	positive reg	4/154	71/18866	0.002714	0.02257	0.01563	FOXM1/GT
BP	GO:005134	regulation	5/154	117/18866	0.002733	0.02262	0.015664	HSP90AA1/
BP	GO:190401	epithelial c	5/154	117/18866	0.002733	0.02262	0.015664	HMOX1/SE
BP	GO:003263	interleukin-	6/154	170/18866	0.002746	0.022678	0.015704	LGALS9/AD
BP	GO:000229	T cell activa	2/154	10/18866	0.002854	0.022811	0.015797	LGALS3/LG
BP	GO:000267	regulation	2/154	10/18866	0.002854	0.022811	0.015797	ADORA2B/
BP	GO:000288	negative re	2/154	10/18866	0.002854	0.022811	0.015797	HMOX1/LG
BP	GO:000618	IMP biosyn	2/154	10/18866	0.002854	0.022811	0.015797	ATIC/GART
BP	GO:000911	purine nucl	2/154	10/18866	0.002854	0.022811	0.015797	ADA/GART
BP	GO:000939	folic acid-c	2/154	10/18866	0.002854	0.022811	0.015797	ATIC/GART
BP	GO:001613	glycoside c	2/154	10/18866	0.002854	0.022811	0.015797	FUCA1/GB
BP	GO:00519C	positive reg	2/154	10/18866	0.002854	0.022811	0.015797	KDR/ALOX1

BP	GO:007039	response tc	2/154	10/18866	0.002854	0.022811	0.015797	CD14/LBP
BP	GO:007122	cellular res	2/154	10/18866	0.002854	0.022811	0.015797	CD14/LBP
BP	GO:007253	purine-con	2/154	10/18866	0.002854	0.022811	0.015797	SLC29A1/SL
BP	GO:012025	hydrocarbc	2/154	10/18866	0.002854	0.022811	0.015797	CYP2E1/AK
BP	GO:200122	regulation c	2/154	10/18866	0.002854	0.022811	0.015797	ABCB1/CA7
BP	GO:001000	glial cell dif	7/154	230/18866	0.002855	0.022811	0.015797	ADORA2A/
BP	GO:004244	pigment m	4/154	72/18866	0.002856	0.022811	0.015797	BLVRA/HM
BP	GO:000743	salivary gla	3/154	35/18866	0.002883	0.022922	0.015874	FGFR1/EGF
BP	GO:003026	apoptotic n	3/154	35/18866	0.002883	0.022922	0.015874	TOP2A/KPN
BP	GO:001921	regulation c	10/154	431/18866	0.00289	0.022933	0.015881	KPNB1/IDH
BP	GO:005080	positive reg	6/154	172/18866	0.00291	0.023039	0.015954	ADORA2A/
BP	GO:004390	regulation c	7/154	231/18866	0.002924	0.023104	0.015999	VPS4B/TOF
BP	GO:005165	establishm	10/154	432/18866	0.002938	0.023164	0.016041	SEC23A/VP
BP	GO:003210	negative re	10/154	433/18866	0.002987	0.023417	0.016217	CHID1/LGA
BP	GO:000915	purine ribo	6/154	173/18866	0.002995	0.023417	0.016217	GSTZ1/ADK
BP	GO:005196	regulation c	4/154	73/18866	0.003003	0.023417	0.016217	ADORA2A/
BP	GO:007240	signal trans	4/154	73/18866	0.003003	0.023417	0.016217	TP53/CHEK
BP	GO:007242	signal trans	4/154	73/18866	0.003003	0.023417	0.016217	TP53/CHEK
BP	GO:005134	negative re	8/154	296/18866	0.003027	0.02356	0.016315	WARS1/TP5
BP	GO:001046	regulation c	6/154	174/18866	0.003081	0.023868	0.016528	HBEGF/ESR
BP	GO:000275	MyD88-dep	3/154	36/18866	0.003126	0.023868	0.016528	BTK/MAP3I
BP	GO:000600	glucose cat	3/154	36/18866	0.003126	0.023868	0.016528	HK2/TP53/
BP	GO:000621	pyrimidine	3/154	36/18866	0.003126	0.023868	0.016528	TK1/CDA/T
BP	GO:001009	specificatio	3/154	36/18866	0.003126	0.023868	0.016528	AR/FGF1/F
BP	GO:003112	developme	3/154	36/18866	0.003126	0.023868	0.016528	AR/FGF1/F
BP	GO:004249	inner ear a	3/154	36/18866	0.003126	0.023868	0.016528	USH1C/MY
BP	GO:004574	positive reg	3/154	36/18866	0.003126	0.023868	0.016528	HBEGF/MM
BP	GO:190533	positive reg	3/154	36/18866	0.003126	0.023868	0.016528	AR/ABL1/A
BP	GO:007239	signal trans	4/154	74/18866	0.003154	0.023971	0.0166	TP53/CHEK
BP	GO:190303	positive reg	4/154	74/18866	0.003154	0.023971	0.0166	HBEGF/HPS
BP	GO:000206	columnar/c	5/154	121/18866	0.003159	0.023971	0.0166	USH1C/MY
BP	GO:000150	regulation c	7/154	235/18866	0.003215	0.024347	0.01686	SLC6A2/AD
BP	GO:002178	glial cell de	5/154	122/18866	0.003273	0.024731	0.017126	ADORA2A/
BP	GO:004424	cellular lipi	7/154	236/18866	0.003292	0.024814	0.017184	HSD17B4/I
BP	GO:003241	lysosome lc	4/154	75/18866	0.003311	0.024814	0.017184	HMOX1/LG
BP	GO:003803	signal trans	4/154	75/18866	0.003311	0.024814	0.017184	FGFR1/CAS
BP	GO:009719	extrinsic ap	4/154	75/18866	0.003311	0.024814	0.017184	FGFR1/CAS
BP	GO:003032	lung develc	6/154	177/18866	0.003352	0.025065	0.017358	FGF1/FGF2
BP	GO:003287	regulation c	7/154	237/18866	0.003369	0.025135	0.017406	FOXM1/PTI
BP	GO:000692	cellular cor	3/154	37/18866	0.003382	0.025135	0.017406	TOP2A/KPN
BP	GO:004507	positive reg	3/154	37/18866	0.003382	0.025135	0.017406	TOP2A/PAE
BP	GO:000614	regulation c	5/154	123/18866	0.00339	0.025143	0.017412	FBP1/GSTZ
BP	GO:190495	positive reg	9/154	370/18866	0.003415	0.02526	0.017493	TP53/ADOI
BP	GO:009006	positive reg	8/154	302/18866	0.003419	0.02526	0.017493	VPS4B/NDC
BP	GO:000598	disaccharid	2/154	11/18866	0.003469	0.02527	0.0175	FBP1/GAA
BP	GO:000604	UDP-N-ace	2/154	11/18866	0.003469	0.02527	0.0175	UAP1/NAG
BP	GO:000618	GTP biosyn	2/154	11/18866	0.003469	0.02527	0.0175	IMPDH1/IN
BP	GO:003353	fatty acid b	2/154	11/18866	0.003469	0.02527	0.0175	ETFDH/ACA
BP	GO:004358	skin morph	2/154	11/18866	0.003469	0.02527	0.0175	GBA/PSEN1
BP	GO:007180	positive reg	2/154	11/18866	0.003469	0.02527	0.0175	MAPK9/TN

BP	GO:190380	positive reg	2/154	11/18866	0.003469	0.02527	0.0175	TP53/EGFR
BP	GO:190352	regulation	8/154	303/18866	0.003488	0.025358	0.017561	HBEGF/AD(
BP	GO:005110	regulation	5/154	124/18866	0.003509	0.02546	0.017631	HMOX1/MI
BP	GO:000193	negative re	10/154	444/18866	0.003568	0.025837	0.017892	FOXM1/WA
BP	GO:007030	regulation	7/154	240/18866	0.00361	0.026086	0.018064	FOXM1/PTI
BP	GO:000709	centrosom	5/154	125/18866	0.003632	0.026193	0.018139	VPS4B/NDC
BP	GO:000315	regulation	3/154	38/18866	0.00365	0.026218	0.018156	AR/FGF1/F
BP	GO:190118	positive reg	3/154	38/18866	0.00365	0.026218	0.018156	HBEGF/MM
BP	GO:003032	respiratory	6/154	181/18866	0.00374	0.026758	0.01853	FGF1/FGF2
BP	GO:190533	regulation	6/154	181/18866	0.00374	0.026758	0.01853	AR/FGF1/A
BP	GO:006104	negative re	4/154	78/18866	0.003814	0.027234	0.018859	FGF2/SERP
BP	GO:001406	regulation	5/154	127/18866	0.003886	0.027699	0.019182	PTPN13/KD
BP	GO:000743	salivary gla	3/154	39/18866	0.00393	0.027904	0.019324	FGFR1/EGF
BP	GO:001045	regulation	3/154	39/18866	0.00393	0.027904	0.019324	AR/FGF2/F
BP	GO:000680	nitric oxide	4/154	79/18866	0.003992	0.028189	0.019521	HBB/HSP90
BP	GO:007126	cellular res	4/154	79/18866	0.003992	0.028189	0.019521	GOT1/CHEI
BP	GO:004484	cell cycle G	8/154	310/18866	0.004002	0.028189	0.019521	BCAT1/TP5
BP	GO:000269	negative re	6/154	184/18866	0.004052	0.028189	0.019521	HMOX1/LG
BP	GO:004632	regulation	6/154	184/18866	0.004052	0.028189	0.019521	PTPN1/HRA
BP	GO:000646	negative re	7/154	246/18866	0.004131	0.028189	0.019521	WARS1/AD
BP	GO:000182	histamine s	2/154	12/18866	0.004141	0.028189	0.019521	BTK/ADA
BP	GO:000286	positive reg	2/154	12/18866	0.004141	0.028189	0.019521	BTK/TNF
BP	GO:000653	cysteine m	2/154	12/18866	0.004141	0.028189	0.019521	CTH/TST
BP	GO:000908	methionine	2/154	12/18866	0.004141	0.028189	0.019521	MTRR/MTA
BP	GO:000915	purine deo	2/154	12/18866	0.004141	0.028189	0.019521	ADK/ADA
BP	GO:003300	negative re	2/154	12/18866	0.004141	0.028189	0.019521	HMOX1/LG
BP	GO:003369	positive reg	2/154	12/18866	0.004141	0.028189	0.019521	HPSE/ABL1
BP	GO:004330	negative re	2/154	12/18866	0.004141	0.028189	0.019521	HMOX1/LG
BP	GO:005154	positive reg	2/154	12/18866	0.004141	0.028189	0.019521	HBEGF/MM
BP	GO:005179	positive reg	2/154	12/18866	0.004141	0.028189	0.019521	HPSE/TNF
BP	GO:007024	regulation	2/154	12/18866	0.004141	0.028189	0.019521	TP53/ADA
BP	GO:007253	pyrimidine-	2/154	12/18866	0.004141	0.028189	0.019521	SLC29A1/SI
BP	GO:015006	regulation	2/154	12/18866	0.004141	0.028189	0.019521	PRKD2/MA
BP	GO:190396	positive reg	2/154	12/18866	0.004141	0.028189	0.019521	ABCB1/PSE
BP	GO:190418	positive reg	2/154	12/18866	0.004141	0.028189	0.019521	KDR/ALOX1
BP	GO:200010	positive reg	2/154	12/18866	0.004141	0.028189	0.019521	FGFR1/CDK
BP	GO:005080	modulation	10/154	454/18866	0.004172	0.028345	0.019629	ADORA2A/
BP	GO:000906	serine fami	3/154	40/18866	0.004224	0.02859	0.019799	CTH/TST/G
BP	GO:190289	positive reg	3/154	40/18866	0.004224	0.02859	0.019799	TP53/FGF2
BP	GO:009917	regulation	10/154	455/18866	0.004236	0.02859	0.019799	ADORA2A/
BP	GO:190353	positive reg	8/154	313/18866	0.004239	0.02859	0.019799	VPS4B/ADC
BP	GO:000926	ribonucleo	6/154	186/18866	0.00427	0.028748	0.019908	GSTZ1/ADK
BP	GO:002176	hippocamp	4/154	81/18866	0.004365	0.029332	0.020313	ALK/ANXA3
BP	GO:000228	lymphocyte	6/154	187/18866	0.004383	0.029343	0.02032	TP53/LGAL
BP	GO:000821	regulation	6/154	187/18866	0.004383	0.029343	0.02032	HMOX1/HE
BP	GO:000606	alcohol me	9/154	385/18866	0.004424	0.029563	0.020473	GOT1/KPNI
BP	GO:001050	positive reg	5/154	131/18866	0.004433	0.02957	0.020477	HMOX1/HK
BP	GO:004312	positive reg	6/154	188/18866	0.004498	0.029891	0.0207	CTH/HMOX
BP	GO:000604	amino suga	3/154	41/18866	0.00453	0.029891	0.0207	UAP1/NAG
BP	GO:000673	NADH meta	3/154	41/18866	0.00453	0.029891	0.0207	HK2/TP53/

BP	GO:003357	response tc	3/154	41/18866	0.00453	0.029891	0.0207	AR/GBA/M
BP	GO:003808	peptidyl-ty	3/154	41/18866	0.00453	0.029891	0.0207	ABL1/KDR/
BP	GO:007252	pyrimidine-	3/154	41/18866	0.00453	0.029891	0.0207	TK1/CDA/T
BP	GO:000628	regulation	5/154	132/18866	0.004578	0.03015	0.020879	FOXM1/GT
BP	GO:004312	regulation	7/154	252/18866	0.004706	0.03094	0.021426	CTH/HMOX
BP	GO:000271	regulation	4/154	83/18866	0.004761	0.031156	0.021576	HMOX1/BT
BP	GO:004814	regulation	4/154	83/18866	0.004761	0.031156	0.021576	S100A6/TP
BP	GO:007147	cellular res	3/154	42/18866	0.00485	0.031156	0.021576	AKR1B1/CA
BP	GO:007259	maintenan	3/154	42/18866	0.00485	0.031156	0.021576	HK2/SKP1/
BP	GO:001082	regulation	6/154	191/18866	0.004855	0.031156	0.021576	HK2/TP53/
BP	GO:002240	negative re	6/154	191/18866	0.004855	0.031156	0.021576	LGALS3/LG
BP	GO:003223	positive reg	2/154	13/18866	0.004868	0.031156	0.021576	ADORA2A/
BP	GO:003242	regulation	2/154	13/18866	0.004868	0.031156	0.021576	EGFR/AGTF
BP	GO:004263	positive reg	2/154	13/18866	0.004868	0.031156	0.021576	HPSE/TNF
BP	GO:004574	positive reg	2/154	13/18866	0.004868	0.031156	0.021576	HBEGF/AD/
BP	GO:004604	IMP metab	2/154	13/18866	0.004868	0.031156	0.021576	ATIC/GART
BP	GO:004634	amino suga	2/154	13/18866	0.004868	0.031156	0.021576	UAP1/NAG
BP	GO:005148	negative re	2/154	13/18866	0.004868	0.031156	0.021576	GOT1/GTF2
BP	GO:006102	eyelid deve	2/154	13/18866	0.004868	0.031156	0.021576	HDAC1/EGI
BP	GO:006104	regulation	2/154	13/18866	0.004868	0.031156	0.021576	SERPINE1/T
BP	GO:004620	nitric oxide	4/154	84/18866	0.004968	0.031634	0.021907	HBB/HSP90
BP	GO:004814	fibroblast p	4/154	84/18866	0.004968	0.031634	0.021907	S100A6/TP
BP	GO:005088	endocrine p	4/154	84/18866	0.004968	0.031634	0.021907	FGFR1/ECE
BP	GO:004427	sulfur comp	6/154	192/18866	0.004979	0.031644	0.021913	CTH/MTRR/
BP	GO:005085	regulation	3/154	43/18866	0.005183	0.032822	0.022729	PRKD2/LGA
BP	GO:190314	regulation	3/154	43/18866	0.005183	0.032822	0.022729	HK2/TP53/
BP	GO:003102	microtubul	5/154	136/18866	0.005191	0.032822	0.022729	VPS4B/NDC
BP	GO:005123	maintenan	8/154	324/18866	0.005203	0.03284	0.022742	HK2/SKP1/
BP	GO:004639	ribose phos	6/154	194/18866	0.005233	0.032971	0.022833	GSTZ1/ADK
BP	GO:003018	B cell differ	5/154	137/18866	0.005353	0.03362	0.023282	TP53/ADAN
BP	GO:005073	positive reg	6/154	195/18866	0.005363	0.03362	0.023282	HBEGF/TP5
BP	GO:190227	regulation	6/154	195/18866	0.005363	0.03362	0.023282	MKI67/PRK
BP	GO:000270	positive reg	5/154	138/18866	0.005518	0.034173	0.023665	CD1A/ADO
BP	GO:004633	positive reg	5/154	138/18866	0.005518	0.034173	0.023665	PTPN1/HRA
BP	GO:000619	purine nucl	3/154	44/18866	0.005529	0.034173	0.023665	PNP/ADA/F
BP	GO:003300	regulation	3/154	44/18866	0.005529	0.034173	0.023665	HMOX1/LG
BP	GO:004406	regulation	3/154	44/18866	0.005529	0.034173	0.023665	FGFR1/AGT
BP	GO:000194	hair follicle	4/154	87/18866	0.005625	0.034173	0.023665	HPSE/HDAC
BP	GO:000914	purine nucl	4/154	87/18866	0.005625	0.034173	0.023665	ADK/ADA/I
BP	GO:003243	positive reg	4/154	87/18866	0.005625	0.034173	0.023665	MAPK9/GB
BP	GO:009004	regulation	4/154	87/18866	0.005625	0.034173	0.023665	HMOX1/FG
BP	GO:200105	reactive nit	4/154	87/18866	0.005625	0.034173	0.023665	HBB/HSP90
BP	GO:000192	positive reg	2/154	14/18866	0.005649	0.034173	0.023665	ECE1/PSEN
BP	GO:000710	mitotic cen	2/154	14/18866	0.005649	0.034173	0.023665	CHEK1/AUI
BP	GO:003579	platelet-de	2/154	14/18866	0.005649	0.034173	0.023665	PTPN1/ABL
BP	GO:004317	nucleotide	2/154	14/18866	0.005649	0.034173	0.023665	ADK/ADA
BP	GO:004600	negative re	2/154	14/18866	0.005649	0.034173	0.023665	LGALS9/CA
BP	GO:004603	GMP metal	2/154	14/18866	0.005649	0.034173	0.023665	IMPDH1/IN
BP	GO:005154	regulation	2/154	14/18866	0.005649	0.034173	0.023665	HBEGF/MN
BP	GO:007023	positive reg	2/154	14/18866	0.005649	0.034173	0.023665	TP53/LGAL

BP	GO:190107	guanosine-	2/154	14/18866	0.005649	0.034173	0.023665	IMPDH1/IM
BP	GO:190379	negative re	2/154	14/18866	0.005649	0.034173	0.023665	TP53/TNF
BP	GO:190492	positive reg	2/154	14/18866	0.005649	0.034173	0.023665	HK2/GBA
BP	GO:000165	urogenital s	8/154	330/18866	0.005795	0.034999	0.024237	AR/FGF1/F
BP	GO:005081	regulation o	4/154	88/18866	0.005856	0.035104	0.02431	HPSE/SERP
BP	GO:000941	response to	5/154	140/18866	0.00586	0.035104	0.02431	TP53/CHEK
BP	GO:007265	establishme	5/154	140/18866	0.00586	0.035104	0.02431	HK2/TP53/
BP	GO:003509	response to	3/154	45/18866	0.005889	0.035104	0.02431	HMOX1/CA
BP	GO:003531	hair cell dif	3/154	45/18866	0.005889	0.035104	0.02431	USH1C/MY
BP	GO:005120	protein inse	3/154	45/18866	0.005889	0.035104	0.02431	TP53/HSP9
BP	GO:007026	necroptotic	3/154	45/18866	0.005889	0.035104	0.02431	TP53/TNF/
BP	GO:009031	regulation o	3/154	45/18866	0.005889	0.035104	0.02431	PRKD2/TP5
BP	GO:003235	response to	5/154	141/18866	0.006036	0.035923	0.024877	ESR2/SSTR
BP	GO:000862	extrinsic ap	4/154	89/18866	0.006093	0.036084	0.024989	HMOX1/LG
BP	GO:002240	molting cyc	4/154	89/18866	0.006093	0.036084	0.024989	HPSE/HDAC
BP	GO:002240	hair cycle p	4/154	89/18866	0.006093	0.036084	0.024989	HPSE/HDAC
BP	GO:005079	activated T	3/154	46/18866	0.006263	0.037028	0.025642	LGALS9/AB
BP	GO:009877	skin epider	4/154	90/18866	0.006337	0.037406	0.025904	HPSE/HDAC
BP	GO:007233	signal trans	7/154	267/18866	0.006411	0.037418	0.025912	FOXM1/MY
BP	GO:000246	germinal ce	2/154	15/18866	0.006483	0.037418	0.025912	ADAM17/A
BP	GO:000600	fructose me	2/154	15/18866	0.006483	0.037418	0.025912	FBP1/AKR1
BP	GO:000941	response to	2/154	15/18866	0.006483	0.037418	0.025912	AKR1B1/M
BP	GO:001567	oxygen tran	2/154	15/18866	0.006483	0.037418	0.025912	HBB/MB
BP	GO:003434	glial cell ap	2/154	15/18866	0.006483	0.037418	0.025912	TP53/CASP
BP	GO:004255	pteridine-c	2/154	15/18866	0.006483	0.037418	0.025912	ATIC/GART
BP	GO:005086	positive reg	2/154	15/18866	0.006483	0.037418	0.025912	PRKD2/ADA
BP	GO:005129	centrosome	2/154	15/18866	0.006483	0.037418	0.025912	CHEK1/AUI
BP	GO:005160	histamine t	2/154	15/18866	0.006483	0.037418	0.025912	BTK/ADA
BP	GO:006013	prepulse in	2/154	15/18866	0.006483	0.037418	0.025912	ADORA2A/
BP	GO:007147	cellular hyp	2/154	15/18866	0.006483	0.037418	0.025912	AKR1B1/M
BP	GO:007180	regulation o	2/154	15/18866	0.006483	0.037418	0.025912	MAPK9/TN
BP	GO:190492	regulation o	2/154	15/18866	0.006483	0.037418	0.025912	HK2/GBA
BP	GO:003367	negative re	7/154	268/18866	0.006539	0.037679	0.026093	WARS1/AD
BP	GO:004643	organopho	5/154	144/18866	0.006588	0.037839	0.026203	IDH1/PRDX
BP	GO:007058	protein loca	5/154	144/18866	0.006588	0.037839	0.026203	HK2/TP53/
BP	GO:006054	respiratory	6/154	204/18866	0.006648	0.037839	0.026203	FGF1/FGF2
BP	GO:000708	mitotic met	3/154	47/18866	0.00665	0.037839	0.026203	VPS4B/KPN
BP	GO:004330	regulation o	3/154	47/18866	0.00665	0.037839	0.026203	HMOX1/LG
BP	GO:004663	positive reg	3/154	47/18866	0.00665	0.037839	0.026203	LGALS9/PN
BP	GO:004667	response to	3/154	47/18866	0.00665	0.037839	0.026203	TP53/HSP9
BP	GO:006098	endocrine h	3/154	47/18866	0.00665	0.037839	0.026203	FGFR1/AGT
BP	GO:190198	regulation o	10/154	486/18866	0.00666	0.037839	0.026203	VPS4B/ND(
BP	GO:003313	regulation o	5/154	145/18866	0.006779	0.038458	0.026632	PRKD2/HSF
BP	GO:005104	positive reg	8/154	340/18866	0.006894	0.038986	0.026998	VPS4B/ADC
BP	GO:199077	protein loca	8/154	340/18866	0.006894	0.038986	0.026998	SEC23A/AR
BP	GO:000683	mitochond	7/154	271/18866	0.006934	0.039093	0.027072	TST/HK2/T
BP	GO:003025	lipid modifi	7/154	271/18866	0.006934	0.039093	0.027072	HSD17B4/C
BP	GO:004663	alpha-beta	5/154	146/18866	0.006974	0.03926	0.027188	LGALS9/AD
BP	GO:003362	regulation o	3/154	48/18866	0.007052	0.039512	0.027362	SERPINE1/E
BP	GO:190027	regulation o	3/154	48/18866	0.007052	0.039512	0.027362	ADORA2A/

BP	GO:200123	regulation	3/154	48/18866	0.007052	0.039512	0.027362	FGFR1/MCI
BP	GO:006109	regulation	4/154	93/18866	0.007107	0.039697	0.02749	HBEGF/PTF
BP	GO:190303	negative re	4/154	93/18866	0.007107	0.039697	0.02749	FGF2/SERP
BP	GO:000604	UDP-N-ace	2/154	16/18866	0.00737	0.040704	0.028188	UAP1/NAG
BP	GO:000610	2-oxoglutai	2/154	16/18866	0.00737	0.040704	0.028188	GOT1/IDH1
BP	GO:000620	pyrimidine	2/154	16/18866	0.00737	0.040704	0.028188	CDA/TYMP
BP	GO:000655	methionine	2/154	16/18866	0.00737	0.040704	0.028188	MTRR/MTA
BP	GO:005144	negative re	2/154	16/18866	0.00737	0.040704	0.028188	ABL1/PSEN
BP	GO:006168	chaperone-	2/154	16/18866	0.00737	0.040704	0.028188	HSP90AA1/
BP	GO:001066	regulation	4/154	94/18866	0.007376	0.040704	0.028188	HMOX1/TP
BP	GO:190126	carbohydra	4/154	94/18866	0.007376	0.040704	0.028188	SLC29A1/A
BP	GO:000325	regulation	3/154	49/18866	0.007467	0.040835	0.028279	GOT1/KDR/
BP	GO:003266	regulation	3/154	49/18866	0.007467	0.040835	0.028279	PRKD2/PDE
BP	GO:003519	production	3/154	49/18866	0.007467	0.040835	0.028279	TP53/EGFR
BP	GO:004205	negative re	3/154	49/18866	0.007467	0.040835	0.028279	HBEGF/EGI
BP	GO:004239	cellular mo	3/154	49/18866	0.007467	0.040835	0.028279	ACADM/AT
BP	GO:009015	establishm	3/154	49/18866	0.007467	0.040835	0.028279	TP53/HSP9
BP	GO:003105	regulation	5/154	149/18866	0.007584	0.041348	0.028633	PRKD2/TP5
BP	GO:190336	positive reg	5/154	149/18866	0.007584	0.041348	0.028633	MAPK9/HS
BP	GO:000307	regulation	4/154	95/18866	0.007653	0.041598	0.028807	AR/PDE4D/
BP	GO:009719	execution p	4/154	95/18866	0.007653	0.041598	0.028807	TOP2A/KPN
BP	GO:000155	regulation	9/154	420/18866	0.007684	0.041704	0.028881	FBP1/HBEG
BP	GO:004339	regulation	6/154	211/18866	0.007793	0.042233	0.029247	PTPRF/MM
BP	GO:000304	regulation	3/154	50/18866	0.007897	0.042421	0.029377	PDE4D/ECE
BP	GO:000906	aspartate fa	3/154	50/18866	0.007897	0.042421	0.029377	GOT1/MTR
BP	GO:006101	positive reg	3/154	50/18866	0.007897	0.042421	0.029377	HNRNPR/M
BP	GO:009730	programme	3/154	50/18866	0.007897	0.042421	0.029377	TP53/TNF/
BP	GO:010102	vascular en	3/154	50/18866	0.007897	0.042421	0.029377	FGF2/ADAN
BP	GO:190556	regulation	3/154	50/18866	0.007897	0.042421	0.029377	FGF2/ADAN
BP	GO:000269	positive reg	4/154	96/18866	0.007936	0.042502	0.029433	LGALS9/SEI
BP	GO:003524	synaptic tra	4/154	96/18866	0.007936	0.042502	0.029433	ADORA2A/
BP	GO:004210	B cell prolif	4/154	97/18866	0.008225	0.043336	0.030011	ABL1/BTK/
BP	GO:004364	dicarboxyli	4/154	97/18866	0.008225	0.043336	0.030011	GOT1/IDH1
BP	GO:004663	regulation	4/154	97/18866	0.008225	0.043336	0.030011	LGALS9/AD
BP	GO:000609	pyruvate m	5/154	152/18866	0.008229	0.043336	0.030011	FBP1/HK2/
BP	GO:004358	skin develo	9/154	425/18866	0.008269	0.043336	0.030011	HPSE/GBA/
BP	GO:000626	DNA ligatio	2/154	17/18866	0.008308	0.043336	0.030011	TOP2A/MG
BP	GO:000697	DNA damag	2/154	17/18866	0.008308	0.043336	0.030011	FOXM1/TP
BP	GO:001937	glycolipid c	2/154	17/18866	0.008308	0.043336	0.030011	FUCA1/GBA
BP	GO:003088	negative re	2/154	17/18866	0.008308	0.043336	0.030011	BTK/CASP3
BP	GO:003106	positive reg	2/154	17/18866	0.008308	0.043336	0.030011	PRKD2/TP5
BP	GO:003268	negative re	2/154	17/18866	0.008308	0.043336	0.030011	LGALS9/EPI
BP	GO:005196	regulation	2/154	17/18866	0.008308	0.043336	0.030011	GBA/TYMP
BP	GO:007024	thymocyte	2/154	17/18866	0.008308	0.043336	0.030011	TP53/ADA
BP	GO:190320	positive reg	2/154	17/18866	0.008308	0.043336	0.030011	MMP3/MC
BP	GO:200126	regulation	2/154	17/18866	0.008308	0.043336	0.030011	LGALS9/MI
BP	GO:003527	exocrine sy	3/154	51/18866	0.008341	0.043336	0.030011	FGFR1/EGF
BP	GO:004814	positive reg	3/154	51/18866	0.008341	0.043336	0.030011	S100A6/EG
BP	GO:005088	neuromusc	3/154	51/18866	0.008341	0.043336	0.030011	USH1C/ABI
BP	GO:007167	regulation	3/154	51/18866	0.008341	0.043336	0.030011	LGALS3/SEI

BP	GO:190289	regulation (	3/154	51/18866	0.008341	0.043336	0.030011	TP53/FGF2,
BP	GO:190358	regulation (	3/154	51/18866	0.008341	0.043336	0.030011	HMOX1/FG
BP	GO:000282	regulation (	5/154	153/18866	0.008453	0.04379	0.030325	CD1A/BTK/
BP	GO:004583	positive reg	5/154	153/18866	0.008453	0.04379	0.030325	VAV2/FGF1
BP	GO:001065	muscle cell	4/154	98/18866	0.008522	0.044025	0.030488	HMOX1/TP
BP	GO:200124	negative re	4/154	98/18866	0.008522	0.044025	0.030488	PTPN1/HD/
BP	GO:000270	regulation (	5/154	154/18866	0.00868	0.044652	0.030922	CD1A/LGAL
BP	GO:001406	phosphatid	5/154	154/18866	0.00868	0.044652	0.030922	PTPN13/KD
BP	GO:005125	negative re	5/154	154/18866	0.00868	0.044652	0.030922	LGALS3/LG.
BP	GO:000182	kidney dev	7/154	283/18866	0.008696	0.044669	0.030934	FGF1/FGF2
BP	GO:000018	activation c	3/154	52/18866	0.0088	0.045076	0.031215	EGFR/MAP
BP	GO:005099	regulation (	3/154	52/18866	0.0088	0.045076	0.031215	HSP90AA1/
BP	GO:003304	regulation (	8/154	356/18866	0.008972	0.045892	0.03178	MKI67/TOF
BP	GO:003009	myeloid cel	9/154	431/18866	0.009016	0.046054	0.031893	FLI1/MB/LC
BP	GO:004506	regulation (	4/154	100/18866	0.009136	0.046603	0.032273	TOP2A/PAE
BP	GO:005067	regulation (	6/154	219/18866	0.009267	0.046636	0.032296	LGALS3/LG.
BP	GO:000931	oligosacchа	3/154	53/18866	0.009273	0.046636	0.032296	FBP1/MAN
BP	GO:006161	pri-miRNA	3/154	53/18866	0.009273	0.046636	0.032296	TP53/FGF2,
BP	GO:000674	NADPH reg	2/154	18/18866	0.009296	0.046636	0.032296	IDH1/TP53
BP	GO:000920	deoxyribon	2/154	18/18866	0.009296	0.046636	0.032296	ADK/ADA
BP	GO:003531	wound hea	2/154	18/18866	0.009296	0.046636	0.032296	HBEGF/AD/
BP	GO:004277	DNA damag	2/154	18/18866	0.009296	0.046636	0.032296	FOXM1/TP!
BP	GO:004518	regulation (	2/154	18/18866	0.009296	0.046636	0.032296	ADORA2A/
BP	GO:004611	nucleobase	2/154	18/18866	0.009296	0.046636	0.032296	ADA/GART
BP	GO:007023	positive reg	2/154	18/18866	0.009296	0.046636	0.032296	TP53/LGAL
BP	GO:015007	positive reg	2/154	18/18866	0.009296	0.046636	0.032296	MMP8/TNF
BP	GO:190022	regulation (	2/154	18/18866	0.009296	0.046636	0.032296	HDAC1/TNI
BP	GO:000008	G1/S transi	7/154	287/18866	0.00935	0.046842	0.032439	BCAT1/TP5
BP	GO:000170	cell fate sp	4/154	101/18866	0.009454	0.047296	0.032753	AR/FGF2/F
BP	GO:003294	regulation (	6/154	221/18866	0.009665	0.048283	0.033436	LGALS3/LG.
BP	GO:000696	cellular def	3/154	54/18866	0.00976	0.04843	0.033538	ADORA2A/
BP	GO:003105	dsRNA proc	3/154	54/18866	0.00976	0.04843	0.033538	TP53/EGFR
BP	GO:005143	regulation (	3/154	54/18866	0.00976	0.04843	0.033538	SKP1/ABL1,
BP	GO:007091	production	3/154	54/18866	0.00976	0.04843	0.033538	TP53/EGFR
BP	GO:190118	negative re	3/154	54/18866	0.00976	0.04843	0.033538	HBEGF/EGI
BP	GO:200006	positive reg	4/154	102/18866	0.009779	0.048455	0.033555	MAPK9/GB
BP	GO:001715	regulation (	6/154	222/18866	0.009868	0.04883	0.033815	VPS4B/HM
BP	GO:190210	positive reg	5/154	159/18866	0.009882	0.048833	0.033817	LGALS9/PN
BP	GO:002240	regulation (	9/154	439/18866	0.01009	0.049761	0.03446	LGALS3/LG.
BP	GO:000204	cell migrati	4/154	103/18866	0.010111	0.049761	0.03446	HMOX1/FG
BP	GO:009886	cellular oxi	4/154	103/18866	0.010111	0.049761	0.03446	HBB/PRDX(
BP	GO:007200	renal syste	7/154	292/18866	0.010219	0.050224	0.034781	FGF1/FGF2
BP	GO:005135	positive reg	3/154	55/18866	0.010263	0.050254	0.034801	ABL1/AGTF
BP	GO:000604	N-acetylglu	2/154	19/18866	0.010335	0.050254	0.034801	NAGK/OGA
BP	GO:001074	positive reg	2/154	19/18866	0.010335	0.050254	0.034801	MAPK9/AG
BP	GO:003202	response tc	2/154	19/18866	0.010335	0.050254	0.034801	FBP1/CD14
BP	GO:004665	tetrahydro	2/154	19/18866	0.010335	0.050254	0.034801	ATIC/GART
BP	GO:005154	keratinocyt	2/154	19/18866	0.010335	0.050254	0.034801	HBEGF/MN
BP	GO:007180	podosome	2/154	19/18866	0.010335	0.050254	0.034801	MAPK9/TN
BP	GO:200025	positive reg	2/154	19/18866	0.010335	0.050254	0.034801	HRAS/ABL1

BP	GO:003157	DNA integr	5/154	161/18866	0.010393	0.050468	0.03495	TOP2A/TP5
BP	GO:000236	cytokine pr	4/154	104/18866	0.01045	0.05061	0.035047	HMOX1/BT
BP	GO:190160	alpha-amin	4/154	104/18866	0.01045	0.05061	0.035047	GOT1/MTR
BP	GO:004573	positive reg	6/154	225/18866	0.010496	0.050767	0.035156	MAPK9/HS
BP	GO:005109	regulation	8/154	367/18866	0.010651	0.051331	0.035547	HMOX1/PT
BP	GO:190265	secondary	5/154	162/18866	0.010655	0.051331	0.035547	KPNB1/IDH
BP	GO:190330	regulation	5/154	162/18866	0.010655	0.051331	0.035547	HMOX1/LG
BP	GO:000697	DNA damag	3/154	56/18866	0.01078	0.051737	0.035828	TP53/AURK
BP	GO:001932	hexose cata	3/154	56/18866	0.01078	0.051737	0.035828	HK2/TP53/
BP	GO:004562	positive reg	4/154	105/18866	0.010796	0.051737	0.035828	LGALS9/PN
BP	GO:200102	positive reg	4/154	105/18866	0.010796	0.051737	0.035828	FOXM1/GT
BP	GO:000708	regulation	5/154	163/18866	0.010922	0.05227	0.036198	VPS4B/MKI
BP	GO:004586	positive reg	8/154	370/18866	0.011147	0.052876	0.036617	MAPK9/LGA
BP	GO:000270	positive reg	4/154	106/18866	0.01115	0.052876	0.036617	CD1A/BTK/
BP	GO:001939	fatty acid o	4/154	106/18866	0.01115	0.052876	0.036617	HSD17B4/E
BP	GO:004663	alpha-beta	4/154	106/18866	0.01115	0.052876	0.036617	LGALS9/PN
BP	GO:007115	regulation	4/154	106/18866	0.01115	0.052876	0.036617	FOXM1/TP5
BP	GO:007186	cellular res	4/154	106/18866	0.01115	0.052876	0.036617	ALK/ABL1/I
BP	GO:007187	cellular res	4/154	106/18866	0.01115	0.052876	0.036617	ALK/ABL1/I
BP	GO:000931	response to	9/154	447/18866	0.011259	0.053322	0.036926	TP53/HRAS
BP	GO:007243	signal trans	3/154	57/18866	0.011312	0.053402	0.036981	TP53/AURK
BP	GO:190240	intracellula	3/154	57/18866	0.011312	0.053402	0.036981	TP53/AURK
BP	GO:190199	regulation	9/154	448/18866	0.011412	0.053402	0.036981	VPS4B/NDC
BP	GO:000290	regulation	2/154	20/18866	0.011422	0.053402	0.036981	BTK/ADA
BP	GO:000614	purine nucl	2/154	20/18866	0.011422	0.053402	0.036981	ADA/GART
BP	GO:000634	DNA methy	2/154	20/18866	0.011422	0.053402	0.036981	HDAC1/DN
BP	GO:000725	I-kappaB pl	2/154	20/18866	0.011422	0.053402	0.036981	MAP3K7/TI
BP	GO:001566	gas transpo	2/154	20/18866	0.011422	0.053402	0.036981	HBB/MB
BP	GO:006096	negative re	2/154	20/18866	0.011422	0.053402	0.036981	TP53/TNF
BP	GO:190200	positive reg	2/154	20/18866	0.011422	0.053402	0.036981	CASP3/TNF
BP	GO:190342	regulation	4/154	107/18866	0.011511	0.053749	0.037221	HBB/HSP90
BP	GO:000940	response to	5/154	166/18866	0.01175	0.054721	0.037894	HMOX1/HS
BP	GO:200124	regulation	5/154	166/18866	0.01175	0.054721	0.037894	TP53/PTPN
BP	GO:001065	negative re	3/154	58/18866	0.011859	0.055088	0.038149	HMOX1/DN
BP	GO:190379	positive reg	3/154	58/18866	0.011859	0.055088	0.038149	ABCB1/ADC
BP	GO:004477	mitotic DNA	4/154	108/18866	0.01188	0.055116	0.038168	TOP2A/TP5
BP	GO:000716	negative re	7/154	301/18866	0.011928	0.055267	0.038273	LGALS3/LGA
BP	GO:003059	leukocyte c	6/154	232/18866	0.012072	0.055867	0.038688	LGALS3/LGA
BP	GO:000228	T cell activa	4/154	109/18866	0.012256	0.05643	0.039078	TP53/LGAL
BP	GO:000693	smooth mu	4/154	109/18866	0.012256	0.05643	0.039078	SSTR2/ADC
BP	GO:003313	positive reg	4/154	109/18866	0.012256	0.05643	0.039078	PRKD2/HSP
BP	GO:200027	regulation	4/154	109/18866	0.012256	0.05643	0.039078	PRKD2/TP5
BP	GO:000281	regulation	5/154	168/18866	0.012324	0.056673	0.039246	CD1A/BTK/
BP	GO:003166	lipopolysac	3/154	59/18866	0.01242	0.056685	0.039255	TNF/CD14/
BP	GO:004660	regulation	3/154	59/18866	0.01242	0.056685	0.039255	VPS4B/CHE
BP	GO:006011	inner ear re	3/154	59/18866	0.01242	0.056685	0.039255	USH1C/MY
BP	GO:007241	signal trans	3/154	59/18866	0.01242	0.056685	0.039255	TP53/AURK
BP	GO:190240	signal trans	3/154	59/18866	0.01242	0.056685	0.039255	TP53/AURK
BP	GO:190240	signal trans	3/154	59/18866	0.01242	0.056685	0.039255	TP53/AURK
BP	GO:000705	cell cycle ar	6/154	234/18866	0.012552	0.056743	0.039295	FOXM1/TP5

BP	GO:001089	positive reg	2/154	21/18866	0.012558	0.056743	0.039295	FGF1/TNF
BP	GO:003581	regulation (	2/154	21/18866	0.012558	0.056743	0.039295	ADORA2A/
BP	GO:005179	regulation (	2/154	21/18866	0.012558	0.056743	0.039295	HPSE/TNF
BP	GO:005195	positive reg	2/154	21/18866	0.012558	0.056743	0.039295	ADORA2A/
BP	GO:009031	positive reg	2/154	21/18866	0.012558	0.056743	0.039295	PRKD2/TP5
BP	GO:009878	response to	2/154	21/18866	0.012558	0.056743	0.039295	HK2/GBA
BP	GO:190359	positive reg	2/154	21/18866	0.012558	0.056743	0.039295	HK2/GBA
BP	GO:005110	negative re	5/154	169/18866	0.012619	0.056761	0.039308	HMOX1/PT
BP	GO:000914	nucleoside	4/154	110/18866	0.01264	0.056761	0.039308	ADK/ADA/I
BP	GO:007186	response to	4/154	110/18866	0.01264	0.056761	0.039308	ALK/ABL1/I
BP	GO:007186	response to	4/154	110/18866	0.01264	0.056761	0.039308	ALK/ABL1/I
BP	GO:190180	positive reg	4/154	110/18866	0.01264	0.056761	0.039308	MAPK9/GB
BP	GO:001993	second-me	9/154	456/18866	0.012692	0.056855	0.039372	ADORA2A/
BP	GO:006201	regulation (	9/154	456/18866	0.012692	0.056855	0.039372	FBP1/KPNE
BP	GO:003287	positive reg	5/154	170/18866	0.012918	0.057797	0.040025	PTPN1/HRA
BP	GO:001922	regulation (	3/154	60/18866	0.012997	0.057876	0.04008	EGFR/ECE1
BP	GO:005099	regulation (	3/154	60/18866	0.012997	0.057876	0.04008	IDH1/HCAF
BP	GO:006109	positive reg	3/154	60/18866	0.012997	0.057876	0.04008	HBEGF/PTF
BP	GO:003444	lipid oxidat	4/154	111/18866	0.013031	0.057876	0.04008	HSD17B4/E
BP	GO:004230	molting cyc	4/154	111/18866	0.013031	0.057876	0.04008	HPSE/HDA(
BP	GO:004263	hair cycle	4/154	111/18866	0.013031	0.057876	0.04008	HPSE/HDA(
BP	GO:004206	gliogenesis	7/154	307/18866	0.013176	0.05845	0.040477	ADORA2A/
BP	GO:000690	phagocytos	8/154	382/18866	0.013303	0.058941	0.040817	ANXA3/VA\
BP	GO:000224	hematopoi	5/154	172/18866	0.013531	0.059629	0.041293	TOP2A/TP5
BP	GO:005071	positive reg	5/154	172/18866	0.013531	0.059629	0.041293	ADORA2A/
BP	GO:007030	positive reg	5/154	172/18866	0.013531	0.059629	0.041293	PTPN1/HRA
BP	GO:000602	glycosamin	3/154	61/18866	0.013589	0.059629	0.041293	FGF2/HPSE
BP	GO:003301	tetrapyrrol	3/154	61/18866	0.013589	0.059629	0.041293	BLVRA/HM
BP	GO:005131	metaphase	3/154	61/18866	0.013589	0.059629	0.041293	VPS4B/KPN
BP	GO:009034	regulation (	3/154	61/18866	0.013589	0.059629	0.041293	FOXM1/TP!
BP	GO:190204	regulation (	3/154	61/18866	0.013589	0.059629	0.041293	HMOX1/LG
BP	GO:003580	regulation (	2/154	22/18866	0.01374	0.059858	0.041452	ADORA2A/
BP	GO:004235	vitamin D n	2/154	22/18866	0.01374	0.059858	0.041452	FGFR1/TNF
BP	GO:004274	regulation (	2/154	22/18866	0.01374	0.059858	0.041452	ADORA2A/
BP	GO:004598	negative re	2/154	22/18866	0.01374	0.059858	0.041452	FBP1/CDA
BP	GO:004605	cAMP meta	2/154	22/18866	0.01374	0.059858	0.041452	EPHA2/PDE
BP	GO:005080	circadian sl	2/154	22/18866	0.01374	0.059858	0.041452	ADORA2A/
BP	GO:005095	sensory pe	5/154	173/18866	0.013844	0.06024	0.041716	USH1C/MY
BP	GO:004603	ATP metab	7/154	311/18866	0.014059	0.061075	0.042295	UQCRC1/FE
BP	GO:007066	regulation (	6/154	240/18866	0.01407	0.061075	0.042295	LGALS3/LG.
BP	GO:003088	regulation (	3/154	62/18866	0.014196	0.061551	0.042625	BTK/CASP3
BP	GO:004320	response to	4/154	114/18866	0.014251	0.06164	0.042686	EGFR/CASP
BP	GO:199074	cellular det	4/154	114/18866	0.014251	0.06164	0.042686	HBB/PRDX(
BP	GO:000726	neurotrans	5/154	175/18866	0.014486	0.062509	0.043288	ADORA2A/
BP	GO:009964	signal relea	5/154	175/18866	0.014486	0.062509	0.043288	ADORA2A/
BP	GO:000609	glycolytic p	4/154	115/18866	0.014673	0.063241	0.043795	FBP1/HK2/
BP	GO:003157	mitotic G1	3/154	63/18866	0.014819	0.063318	0.043848	TP53/AURK
BP	GO:004663	positive reg	3/154	63/18866	0.014819	0.063318	0.043848	LGALS9/PN
BP	GO:000000	mitochondi	2/154	23/18866	0.014969	0.063318	0.043848	TP53/TYMF
BP	GO:000191	regulation (	2/154	23/18866	0.014969	0.063318	0.043848	ECE1/PSEN

BP	GO:000922	nucleotide-	2/154	23/18866	0.014969	0.063318	0.043848	UAP1/NAG
BP	GO:003128	positive reg	2/154	23/18866	0.014969	0.063318	0.043848	ADORA2B/
BP	GO:003581	renal sodiu	2/154	23/18866	0.014969	0.063318	0.043848	ADORA2A/
BP	GO:004202	protein refo	2/154	23/18866	0.014969	0.063318	0.043848	HSP90AA1/
BP	GO:004358	tongue dev	2/154	23/18866	0.014969	0.063318	0.043848	HDAC1/EGI
BP	GO:004613	pyrimidine	2/154	23/18866	0.014969	0.063318	0.043848	CDA/TYMP
BP	GO:004802	positive reg	2/154	23/18866	0.014969	0.063318	0.043848	PRDX6/HSP
BP	GO:006014	negative re	2/154	23/18866	0.014969	0.063318	0.043848	TP53/TNF
BP	GO:006044	branching i	2/154	23/18866	0.014969	0.063318	0.043848	FGFR1/TNF
BP	GO:006096	negative re	2/154	23/18866	0.014969	0.063318	0.043848	TP53/TNF
BP	GO:190358	positive reg	2/154	23/18866	0.014969	0.063318	0.043848	HMOX1/AG
BP	GO:200081	regulation	2/154	23/18866	0.014969	0.063318	0.043848	EPHA2/TNF
BP	GO:000675	ATP genera	4/154	116/18866	0.015103	0.063811	0.044189	FBP1/HK2/
BP	GO:000269	regulation	9/154	470/18866	0.015185	0.064084	0.044379	HMOX1/CD
BP	GO:000243	inflammato	3/154	64/18866	0.015456	0.064703	0.044807	BTK/AHCY/
BP	GO:003250	maintenan	3/154	64/18866	0.015456	0.064703	0.044807	HK2/SKP1/
BP	GO:004478	G1 DNA da	3/154	64/18866	0.015456	0.064703	0.044807	TP53/AURK
BP	GO:004481	mitotic G1/	3/154	64/18866	0.015456	0.064703	0.044807	TP53/AURK
BP	GO:004663	regulation	3/154	64/18866	0.015456	0.064703	0.044807	LGALS9/PN
BP	GO:006013	maternal p	3/154	64/18866	0.015456	0.064703	0.044807	AR/LGALS9
BP	GO:007026	necrotic ce	3/154	64/18866	0.015456	0.064703	0.044807	TP53/TNF/
BP	GO:003166	response to	9/154	473/18866	0.015764	0.065914	0.045646	HMOX1/TP
BP	GO:000941	response to	7/154	319/18866	0.015951	0.066465	0.046027	TP53/META
BP	GO:000705	mitotic spir	4/154	118/18866	0.015987	0.066465	0.046027	VPS4B/KPN
BP	GO:000854	fibroblast g	4/154	118/18866	0.015987	0.066465	0.046027	PRKD2/FGF
BP	GO:002198	cerebral co	4/154	118/18866	0.015987	0.066465	0.046027	EGFR/ATIC/
BP	GO:005086	negative re	4/154	118/18866	0.015987	0.066465	0.046027	LGALS3/LG
BP	GO:001057	vascular en	3/154	65/18866	0.016109	0.066769	0.046238	HPSE/ADOI
BP	GO:004864	animal orga	3/154	65/18866	0.016109	0.066769	0.046238	AR/FGF1/F
BP	GO:190179	regulation	5/154	180/18866	0.016177	0.066769	0.046238	TP53/HDAC
BP	GO:000271	negative re	2/154	24/18866	0.016243	0.066769	0.046238	HMOX1/TN
BP	GO:004406	regulation	2/154	24/18866	0.016243	0.066769	0.046238	ADORA2A/
BP	GO:005099	negative re	2/154	24/18866	0.016243	0.066769	0.046238	HCAR2/TNI
BP	GO:006044	branching i	2/154	24/18866	0.016243	0.066769	0.046238	AR/EPHA2
BP	GO:190299	positive reg	2/154	24/18866	0.016243	0.066769	0.046238	CASP3/TNF
BP	GO:190330	negative re	2/154	24/18866	0.016243	0.066769	0.046238	HMOX1/LG
BP	GO:200063	positive reg	2/154	24/18866	0.016243	0.066769	0.046238	TP53/EGFR
BP	GO:004828	organelle fi	9/154	476/18866	0.016358	0.067166	0.046513	VPS4B/MKI
BP	GO:004561	regulation	5/154	181/18866	0.01653	0.067797	0.04695	LGALS9/PN
BP	GO:000854	epidermis	9/154	477/18866	0.01656	0.067842	0.046981	USH1C/MY
BP	GO:190165	cellular res	8/154	398/18866	0.016635	0.068072	0.04714	GOT1/TP53
BP	GO:000914	purine nucl	3/154	66/18866	0.016777	0.068271	0.047278	ADK/IMPDI
BP	GO:003276	regulation	3/154	66/18866	0.016777	0.068271	0.047278	HSP90AA1/
BP	GO:004249	mechanore	3/154	66/18866	0.016777	0.068271	0.047278	USH1C/MY
BP	GO:004636	monosacch	3/154	66/18866	0.016777	0.068271	0.047278	HK2/TP53/
BP	GO:200037	negative re	3/154	66/18866	0.016777	0.068271	0.047278	HK2/TP53/
BP	GO:009028	negative re	5/154	182/18866	0.016889	0.068649	0.04754	TP53/FGF2
BP	GO:001607	synaptic ve	4/154	121/18866	0.017372	0.070364	0.048728	ADORA2A/
BP	GO:190054	regulation	4/154	121/18866	0.017372	0.070364	0.048728	FBP1/GSTZ
BP	GO:000602	aminoglyca	3/154	67/18866	0.017461	0.070364	0.048728	FGF2/HPSE

BP	GO:000916	nucleotide	3/154	67/18866	0.017461	0.070364	0.048728	PNP/ADA/F
BP	GO:005091	positive ch	3/154	67/18866	0.017461	0.070364	0.048728	LGALS3/FG
BP	GO:001587	norepineph	2/154	25/18866	0.017562	0.070364	0.048728	SLC6A2/AD
BP	GO:001940	alditol meta	2/154	25/18866	0.017562	0.070364	0.048728	GOT1/AKR1
BP	GO:002241	circadian sl	2/154	25/18866	0.017562	0.070364	0.048728	ADORA2A/
BP	GO:004603	GTP metab	2/154	25/18866	0.017562	0.070364	0.048728	IMPDH1/IM
BP	GO:005085	positive reg	2/154	25/18866	0.017562	0.070364	0.048728	PRKD2/ADA
BP	GO:005189	positive reg	2/154	25/18866	0.017562	0.070364	0.048728	ABL1/KDR
BP	GO:006014	positive reg	2/154	25/18866	0.017562	0.070364	0.048728	TP53/EGFR
BP	GO:006057	morphoger	2/154	25/18866	0.017562	0.070364	0.048728	AR/EGFR
BP	GO:003021	T cell differ	6/154	253/18866	0.01779	0.071121	0.049252	TP53/LGAL
BP	GO:003166	cellular res	6/154	253/18866	0.01779	0.071121	0.049252	HMOX1/TP
BP	GO:009723	cellular res	4/154	122/18866	0.01785	0.071282	0.049363	HBB/PRDX6
BP	GO:200004	regulation	5/154	185/18866	0.017995	0.071783	0.04971	TP53/ADAM
CC	GO:003198	vesicle lum	20/154	328/19559	1.45E-12	4.71E-10	3.80E-10	F13A1/VW
CC	GO:006020	cytoplasmi	19/154	326/19559	1.20E-11	1.95E-09	1.58E-09	F13A1/VW
CC	GO:003477	secretory g	17/154	322/19559	7.12E-10	7.71E-08	6.22E-08	F13A1/VW
CC	GO:010100	ficolin-1-ric	10/154	124/19559	5.17E-08	2.56E-06	2.07E-06	KPNB1/HBI
CC	GO:190481	ficolin-1-ric	10/154	124/19559	5.17E-08	2.56E-06	2.07E-06	KPNB1/HBI
CC	GO:004512	membrane	15/154	329/19559	5.31E-08	2.56E-06	2.07E-06	HMOX1/CD
CC	GO:009885	membrane	15/154	330/19559	5.52E-08	2.56E-06	2.07E-06	HMOX1/CD
CC	GO:009858	membrane	15/154	343/19559	9.14E-08	3.71E-06	3.00E-06	HMOX1/CD
CC	GO:000577	vacuolar lu	11/154	173/19559	1.27E-07	4.58E-06	3.70E-06	CHID1/LYZ/
CC	GO:004320	lysosomal l	8/154	96/19559	9.08E-07	2.95E-05	2.38E-05	CHID1/MAI
CC	GO:190472	tertiary gra	6/154	55/19559	4.56E-06	0.000135	0.000109	LYZ/HBB/ID
CC	GO:000576	primary lys	8/154	155/19559	3.19E-05	0.000797	0.000642	LYZ/PRDX6
CC	GO:004258	azurophil g	8/154	155/19559	3.19E-05	0.000797	0.000642	LYZ/PRDX6
CC	GO:007082	tertiary gra	8/154	164/19559	4.76E-05	0.001106	0.000892	LYZ/HBB/ID
CC	GO:009905	integral cor	5/154	74/19559	0.000295	0.00639	0.005154	ADORA2A/
CC	GO:009888	intrinsic co	5/154	83/19559	0.000502	0.009685	0.007811	ADORA2A/
CC	GO:004320	myelin shea	4/154	47/19559	0.000507	0.009685	0.007811	PMP2/HSP9
CC	GO:003557	azurophil g	5/154	91/19559	0.000765	0.013813	0.01114	LYZ/PRDX6
CC	GO:003558	specific gra	4/154	62/19559	0.001446	0.024197	0.019514	KPNB1/LYZ
CC	GO:004273	presynaptic	6/154	156/19559	0.001489	0.024197	0.019514	SLC6A2/AD
CC	GO:000577	vacuolar m	10/154	427/19559	0.002088	0.032309	0.026056	MYO6/MAI
CC	GO:004517	apical part	10/154	433/19559	0.00231	0.034122	0.027519	USH1C/MY
CC	GO:003013	coated vesi	8/154	298/19559	0.002535	0.035816	0.028885	SEC23A/VV
CC	GO:000576	lysosomal r	9/154	375/19559	0.002946	0.038294	0.030883	MYO6/HPS
CC	GO:009885	lytic vacuol	9/154	375/19559	0.002946	0.038294	0.030883	MYO6/HPS
CC	GO:003525	ciliary rootl	2/154	11/19559	0.003233	0.040417	0.032595	PSEN2/PSE
CC	GO:003013	clathrin-coa	6/154	194/19559	0.004404	0.052568	0.042395	VWF/MYO6
CC	GO:001658	Sin3 compl	2/154	13/19559	0.004538	0.052568	0.042395	MORF4L1/I
CC	GO:000030	cyclin-depe	3/154	43/19559	0.004691	0.052568	0.042395	CDK2/CCN/
CC	GO:190255	serine/thre	4/154	89/19559	0.005375	0.058231	0.046962	CDK2/MAP
MF	GO:000408	carbonate	7/154	15/18352	1.55E-11	7.43E-09	6.17E-09	CA14/CA7/
MF	GO:001683	hydro-lyase	8/154	59/18352	3.16E-08	7.56E-06	6.27E-06	HSD17B4/C
MF	GO:001683	carbon-oxy	8/154	74/18352	1.93E-07	3.08E-05	2.56E-05	HSD17B4/C
MF	GO:001682	lyase activi	11/154	189/18352	5.78E-07	6.90E-05	5.73E-05	CTH/GOT1/
MF	GO:000455	hydrolase a	7/154	96/18352	1.63E-05	0.001559	0.001294	LYZ/MAN2I
MF	GO:000417	endopeptic	14/154	440/18352	2.10E-05	0.00167	0.001387	MMP13/M

MF	GO:000422	metalloend	7/154	108/18352	3.51E-05	0.002396	0.001989	MMP13/M
MF	GO:000419	aspartic-tyl	4/154	25/18352	5.26E-05	0.003142	0.002608	CASP3/PSE
MF	GO:00700C	aspartic-tyl	4/154	26/18352	6.17E-05	0.003183	0.002643	CASP3/PSE
MF	GO:007172	lipopeptide	3/154	10/18352	6.66E-05	0.003183	0.002643	CD1A/CD1
MF	GO:001679	hydrolase a	7/154	133/18352	0.000131	0.005712	0.004742	LYZ/MAN2I
MF	GO:000471	protein tyr	7/154	135/18352	0.000144	0.005746	0.004771	ALK/ABL1/I
MF	GO:000471	transmemk	5/154	61/18352	0.000159	0.00584	0.004849	ALK/EPHA2
MF	GO:007085	growth fact	7/154	141/18352	0.000189	0.006305	0.005235	HBEGF/VA\
MF	GO:000823	metallopep	8/154	189/18352	0.000198	0.006305	0.005235	MMP13/M
MF	GO:004802	monosacch	5/154	69/18352	0.000284	0.008494	0.007052	FBP1/HK2/
MF	GO:003517	histone kin	3/154	17/18352	0.000361	0.010163	0.008437	CHEK1/AUI
MF	GO:003024	carbohydra	9/154	267/18352	0.000431	0.011455	0.00951	UAP1/FBP1
MF	GO:004529	cadherin bi	10/154	332/18352	0.000505	0.012159	0.010095	IDH1/PRDX
MF	GO:000532	neurotrans	3/154	19/18352	0.000509	0.012159	0.010095	SLC6A2/SL(
MF	GO:001919	transmemk	5/154	80/18352	0.000565	0.012858	0.010675	ALK/EPHA2
MF	GO:00470C	MAP kinase	3/154	20/18352	0.000595	0.012925	0.01073	MAPK9/MA
MF	GO:00192C	carbohydra	3/154	21/18352	0.00069	0.014335	0.011901	NAGK/HK2,
MF	GO:005111	ATPase bin	5/154	88/18352	0.000872	0.017377	0.014427	AR/EGFR/P
MF	GO:000108	RNA polym	4/154	56/18352	0.001248	0.023859	0.019808	FBP1/TP53,
MF	GO:000517	integrin bir	6/154	144/18352	0.001364	0.025076	0.020819	VWF/FGF1,
MF	GO:004282	histone dea	5/154	115/18352	0.002854	0.050521	0.041943	TOP2A/TP5
MF	GO:001681	hydrolase a	3/154	35/18352	0.003115	0.05269	0.043744	ADA/CDA/
MF	GO:001982	oxygen bin	3/154	36/18352	0.003377	0.05269	0.043744	CYP2E1/HB
MF	GO:001661	oxidoreduc	5/154	120/18352	0.003427	0.05269	0.043744	HSD17B4/I
MF	GO:003162	ubiquitin pı	8/154	297/18352	0.003648	0.05269	0.043744	UQCRC1/PI
MF	GO:000553	glucose bin	2/154	11/18352	0.003661	0.05269	0.043744	HK2/HK1
MF	GO:00168C	hydrolase a	2/154	11/18352	0.003661	0.05269	0.043744	AHCY/ALO)
MF	GO:000467	protein ser	10/154	435/18352	0.003748	0.05269	0.043744	MAPK9/PR
MF	GO:003107	heat shock	5/154	127/18352	0.004364	0.0596	0.049481	CYP2E1/KP

Count
15
7
23
9
12
9
7
22
22
16
8
11
8
9
8
10
14
19
17
8
9
8
7
6
19
6
8
12
6
6
6
15
8
7
17
7
12
8
10
5
9
14
14
14
8
14
6
15
6

12
13
16
6
16
6
10
11
11
10
5
12
14
7
14
11
4
4
8
7
11
10
6
12
12
11
14
12
12
15
13
15
6
13
4
4
5
5
14
10
7
7
4
10
10
14
13
9
11
13

6
5
9
10
11
14
5
6
6
11
14
4
13
11
8
7
13
7
11
4
12
6
5
9
10
6
6
10
13
9
7
5
9
4
12
14
7
8
9
5
10
8
12
5
6
8
13
9
11
7

4
9
11
10
7
6
10
13
7
3
3
3
3
11
11
8
5
5
5
10
4
12
8
7
3
3
3
11
9
6
11
5
5
5
11
8
8
8
8
5
6
4
10
5
8
10
4
9
8
7

7
6
6
11
3
3
3
7
4
5
8
7
9
6
5
8
7
11
10
8
3
4
10
9
11
7
9
6
8
4
9
9
3
3
7
7
10
4
4
6
5
10
11
6
3
10
10
5
8
7

11
8
5
10
10
7
7
11
4
6
11
11
10
10
6
6
7
3
3
3
12
5
7
4
4
8
6
6
4
4
6
8
3
3
3
5
5
10
4
9
10
5
8
12
12
12
10
7
8
5

4
4
3
3
4
4
4
10
9
5
5
3
3
4
4
4
8
6
10
5
4
3
6
5
10
5
10
3
3
10
7
4
5
9
9
5
6
3
3
3
6
7
4
4
7
11
6
5
5
4

3
3
11
5
8
6
4
4
7
5
8
7
3
6
8
9
4
5
5
3
3
4
4
4
10
7
7
5
5
4
4
9
7
3
3
3
3
6
5
5
7
5
8
3
6
5
5
5
5
4

9
7
4
4
6
9
5
7
3
8
9
9
7
7
11
5
11
7
5
3
3
3
7
4
4
4
11
7
7
3
3
6
5
9
3
3
3
4
4
5
5
6
2
2
2
2
2
2
2
2

2
2
2
2
2
7
4
3
3
10
6
7
10
10
6
4
4
4
8
6
3
3
3
3
3
3
3
3
4
4
5
7
5
7
4
4
4
6
7
3
3
5
9
8
2
2
2
2
2
2

2
8
5
10
7
5
3
3
6
6
4
5
3
3
4
4
8
6
6
7
2
2
2
2
2
2
2
2
2
2
2
2
2
2
2
2
10
3
3
10
8
6
4
6
6
9
5
6
3
3

3
3
3
5
7
4
4
3
3
6
6
2
2
2
2
2
2
2
2
2
4
4
4
6
3
3
5
8
6
5
6
6
5
5
3
3
3
4
4
4
4
4
2
2
2
2
2
2
2
2

2
2
2
8
4
5
5
3
3
3
3
3
5
4
4
4
3
4
7
2
2
2
2
2
2
2
2
2
2
2
2
2
7
5
5
6
3
3
3
3
3
10
5
8
8
7
7
5
3
3

3
4
4
2
2
2
2
2
2
4
4
3
3
3
3
3
3
5
5
4
4
9
6
3
3
3
3
3
3
4
4
4
4
4
5
9
2
2
2
2
2
2
2
2
2
2
3
3
3
3

3
3
5
5
4
4
5
5
5
7
3
3
8
9
4
6
3
3
2
2
2
2
2
2
2
2
2
7
4
6
3
3
3
3
3
4
6
5
9
4
4
7
3
2
2
2
2
2
2
2

5
4
4
6
8
5
5
3
3
4
4
5
8
4
4
4
4
4
4
9
3
3
9
2
2
2
2
2
2
2
4
5
5
3
3
4
7
6
4
4
4
4
5
3
3
3
3
3
3
6

2
2
2
2
2
2
2
5
4
4
4
4
9
9
5
3
3
3
4
4
4
7
8
5
5
5
3
3
3
3
3
2
2
2
2
2
2
5
7
6
3
4
4
5
5
4
3
3
2
2

2
2
2
2
2
2
2
2
2
2
2
2
4
9
3
3
3
3
3
3
3
9
7
4
4
4
4
3
3
5
2
2
2
2
2
2
2
9
5
9
8
3
3
3
3
3
5
4
4
3

3
3
2
2
2
2
2
2
2
2
6
6
4
5
20
19
17
10
10
15
15
15
11
8
6
8
8
8
5
5
4
5
4
6
10
10
8
9
9
2
6
2
3
4
7
8
8
11
7
14

7
4
4
3
7
7
5
7
8
5
3
9
10
3
5
3
3
5
4
6
5
3
3
5
8
2
2
10
5

Supplemental Material 6

ID	Descriptior	GeneRatio	BgRatio	pvalue	p.adjust	qvalue	geneID	Count
hsa00910	Nitrogen m	7/126	17/8098	3.19E-09	8.11E-07	5.07E-07	CA14/CA7/	7
hsa05417	Lipid and at	16/126	215/8098	1.88E-07	1.63E-05	1.02E-05	MAPK9/TP5	16
hsa04657	IL-17 signal	11/126	94/8098	1.93E-07	1.63E-05	1.02E-05	MAPK9/HS	11
hsa00270	Cysteine ar	8/126	50/8098	8.53E-07	5.42E-05	3.39E-05	CTH/GOT1/	8
hsa05219	Bladder car	7/126	41/8098	2.71E-06	0.000133	8.30E-05	HBEGF/TP5	7
hsa04330	Notch signa	8/126	59/8098	3.13E-06	0.000133	8.30E-05	HDAC1/AD	8
hsa05161	Hepatitis B	12/126	162/8098	7.42E-06	0.000269	0.000168	MAPK9/TP5	12
hsa04010	MAPK signa	16/126	294/8098	1.17E-05	0.000372	0.000233	MAPK9/TP5	16
hsa05169	Epstein-Bar	13/126	202/8098	1.41E-05	0.000398	0.000249	MAPK9/SEI	13
hsa05215	Prostate ca	9/126	97/8098	1.81E-05	0.000454	0.000284	TP53/AR/H	9
hsa01522	Endocrine r	9/126	98/8098	1.97E-05	0.000454	0.000284	MAPK9/HB	9
hsa05418	Fluid shear	10/126	139/8098	5.69E-05	0.001185	0.000741	MAPK9/HM	10
hsa05171	Coronaviru	13/126	232/8098	6.06E-05	0.001185	0.000741	F13A1/MA	13
hsa04936	Alcoholic li	10/126	142/8098	6.82E-05	0.0012	0.000751	MAPK9/CY	10
hsa04930	Type II diab	6/126	46/8098	7.09E-05	0.0012	0.000751	MAPK9/HK	6
hsa05205	Proteoglyca	12/126	205/8098	7.77E-05	0.001231	0.00077	HBEGF/TP5	12
hsa04664	Fc epsilon R	7/126	68/8098	8.24E-05	0.001231	0.00077	MAPK9/VA	7
hsa05120	Epithelial c	7/126	70/8098	9.93E-05	0.001328	0.000831	MAPK9/HB	7
hsa05230	Central car	7/126	70/8098	9.93E-05	0.001328	0.000831	HK2/IDH1/	7
hsa04933	AGE-RAGE	8/126	100/8098	0.000154	0.001962	0.001228	MAPK9/SEI	8
hsa04914	Progestero	8/126	102/8098	0.000177	0.002145	0.001343	MAPK9/HS	8
hsa04210	Apoptosis	9/126	136/8098	0.000255	0.002944	0.001842	MAPK9/TP5	9
hsa04910	Insulin sign	9/126	137/8098	0.000269	0.002975	0.001861	FBP1/MAP	9
hsa05010	Alzheimer d	16/126	384/8098	0.000284	0.003008	0.001882	UQCRC1/M	16
hsa04012	ErbB signal	7/126	85/8098	0.000337	0.003301	0.002066	MAPK9/HB	7
hsa04668	TNF signali	8/126	112/8098	0.000338	0.003301	0.002066	MAPK9/MM	8
hsa05210	Colorectal c	7/126	86/8098	0.000362	0.003409	0.002134	MAPK9/TP5	7
hsa04015	Rap1 signal	11/126	210/8098	0.00041	0.003718	0.002326	PRKD2/VAV	11
hsa04722	Neurotroph	8/126	119/8098	0.000509	0.004458	0.00279	MAPK9/TP5	8
hsa05208	Chemical ca	11/126	223/8098	0.00068	0.005755	0.003601	UQCRC1/M	11
hsa04110	Cell cycle	8/126	126/8098	0.000745	0.006101	0.003818	SKP1/TP53/	8
hsa05218	Melanoma	6/126	72/8098	0.00085	0.006691	0.004187	TP53/FGF1/	6
hsa04926	Relaxin sigr	8/126	129/8098	0.000869	0.006691	0.004187	MAPK9/HR	8
hsa00230	Purine met	8/126	130/8098	0.000914	0.006831	0.004275	ADK/PNP/A	8
hsa04014	Ras signalir	11/126	232/8098	0.000943	0.006842	0.004281	MAPK9/FG	11
hsa05133	Pertussis	6/126	76/8098	0.001131	0.007605	0.004759	MAPK9/MA	6
hsa04620	Toll-like rec	7/126	104/8098	0.001138	0.007605	0.004759	MAPK9/MA	7
hsa04660	T cell recep	7/126	104/8098	0.001138	0.007605	0.004759	MAPK9/VA	7
hsa00052	Galactose r	4/126	31/8098	0.00127	0.008064	0.005046	HK2/HK1/A	4
hsa01523	Antifolate r	4/126	31/8098	0.00127	0.008064	0.005046	ATIC/ALOX	4
hsa05162	Measles	8/126	139/8098	0.001411	0.008471	0.005301	MAPK9/TP5	8
hsa04931	Insulin resis	7/126	108/8098	0.001419	0.008471	0.005301	MAPK9/PTI	7
hsa04215	Apoptosis -	4/126	32/8098	0.001434	0.008471	0.005301	MAPK9/MA	4
hsa00983	Drug metal	6/126	80/8098	0.001478	0.008533	0.00534	CYP2E1/IM	6
hsa05131	Shigellosis	11/126	247/8098	0.001566	0.008839	0.005531	MAPK9/HK	11
hsa00051	Fructose ar	4/126	33/8098	0.001612	0.008903	0.005571	FBP1/HK2/	4
hsa05132	Salmonella	11/126	249/8098	0.00167	0.009025	0.005648	MAPK9/MY	11
hsa05145	Toxoplasm	7/126	112/8098	0.001752	0.009269	0.0058	MAPK9/MA	7
hsa05166	Human T-c	10/126	222/8098	0.002352	0.012193	0.00763	MAPK9/TP5	10

hsa04071	Sphingolipi	7/126	119/8098	0.002477	0.012583	0.007874	MAPK9/TP!	7
hsa00511	Other glyca	3/126	18/8098	0.00253	0.0126	0.007885	MAN2B1/F	3
hsa04218	Cellular ser	8/126	156/8098	0.002918	0.014236	0.008908	FOXM1/TP!	8
hsa01210	2-Oxocarbo	3/126	19/8098	0.00297	0.014236	0.008908	GOT1/BCAT	3
hsa04912	GnRH signa	6/126	93/8098	0.003183	0.014973	0.00937	MAPK9/HB	6
hsa04622	RIG-I-like re	5/126	70/8098	0.004547	0.020625	0.012907	MAPK9/MA	5
hsa04917	Prolactin si	5/126	70/8098	0.004547	0.020625	0.012907	MAPK9/ESI	5
hsa04520	Adherens ju	5/126	71/8098	0.004832	0.021532	0.013474	PTPRF/PTP	5
hsa04137	Mitophagy	5/126	72/8098	0.005129	0.022462	0.014056	MAPK9/TP!	5
hsa04115	p53 signalir	5/126	73/8098	0.005439	0.023416	0.014653	TP53/SERP	5
hsa04915	Estrogen si	7/126	138/8098	0.005624	0.023718	0.014842	HBEGF/ESR	7
hsa05170	Human imr	9/126	212/8098	0.005696	0.023718	0.014842	MAPK9/SKI	9
hsa05212	Pancreatic	5/126	76/8098	0.006448	0.026416	0.016531	MAPK9/TP!	5
hsa00520	Amino suga	4/126	49/8098	0.006897	0.027807	0.017401	UAP1/NAG	4
hsa05224	Breast canc	7/126	147/8098	0.007882	0.03128	0.019574	ESR2/TP53/	7
hsa05226	Gastric can	7/126	149/8098	0.008463	0.033071	0.020695	TP53/ABCB	7
hsa05206	MicroRNAs	11/126	310/8098	0.008758	0.033648	0.021056	HMOX1/TP	11
hsa04151	PI3K-Akt sig	12/126	354/8098	0.008876	0.033648	0.021056	VWF/TP53/	12
hsa05202	Transcriptio	8/126	192/8098	0.010017	0.037416	0.023414	FLI1/TP53/	8
hsa04932	Non-alcoho	7/126	155/8098	0.010398	0.038276	0.023952	UQCRC1/M	7
hsa00240	Pyrimidine	4/126	56/8098	0.011003	0.039924	0.024983	PNP/TK1/C	4
hsa05130	Pathogenic	8/126	197/8098	0.011585	0.041253	0.025815	MAPK9/MY	8
hsa05134	Legionellos	4/126	57/8098	0.011694	0.041253	0.025815	CASP3/HSP	4
hsa04510	Focal adhes	8/126	201/8098	0.012965	0.04511	0.028229	MAPK9/VW	8
hsa05165	Human pap	11/126	331/8098	0.013853	0.047549	0.029755	VWF/TP53/	11
hsa05203	Viral carcin	8/126	204/8098	0.014076	0.047671	0.029832	TP53/HRAS	8
hsa04380	Osteoclast	6/126	128/8098	0.014614	0.048668	0.030456	MAPK9/MA	6
hsa04310	Wnt signali	7/126	166/8098	0.014754	0.048668	0.030456	MAPK9/SKI	7
hsa04068	FoxO signal	6/126	131/8098	0.016223	0.052828	0.033058	MAPK9/HR	6
hsa04141	Protein pro	7/126	171/8098	0.017113	0.055021	0.034431	SEC23A/MA	7
hsa00500	Starch and	3/126	36/8098	0.018072	0.056795	0.035541	HK2/HK1/G	3
hsa05231	Choline me	5/126	98/8098	0.018112	0.056795	0.035541	MAPK9/HR	5
hsa05135	Yersinia inf	6/126	137/8098	0.019799	0.061329	0.038378	MAPK9/VA	6
hsa05221	Acute myel	4/126	67/8098	0.020162	0.061701	0.038611	HRAS/CCNA	4
hsa04024	cAMP signa	8/126	219/8098	0.020701	0.062595	0.03917	MAPK9/VA	8
hsa05142	Chagas dise	5/126	102/8098	0.021158	0.063226	0.039565	MAPK9/SEI	5
hsa05152	Tuberculos	7/126	180/8098	0.022013	0.064846	0.040579	MAPK9/MA	7
hsa04920	Adipocytok	4/126	69/8098	0.022211	0.064846	0.040579	MAPK9/MA	4
hsa04064	NF-kappa B	5/126	104/8098	0.0228	0.06507	0.040719	BTK/MAP3I	5
hsa04625	C-type lecti	5/126	104/8098	0.0228	0.06507	0.040719	MAPK9/HR	5
hsa04928	Parathyroic	5/126	106/8098	0.024522	0.069207	0.043308	HBEGF/MM	5
hsa05223	Non-small (	4/126	72/8098	0.025516	0.07122	0.044568	ALK/TP53/I	4
hsa04066	HIF-1 signa	5/126	109/8098	0.027259	0.075259	0.047096	HMOX1/HK	5
hsa01230	Biosynthes	4/126	75/8098	0.029102	0.079482	0.049738	CTH/GOT1/	4
